# From RNA sequence to its three-dimensional structure: geometrical structure, stability and dynamics of selected fragments of SARS-CoV-2 RNA

**DOI:** 10.1093/nargab/lqae062

**Published:** 2024-06-04

**Authors:** Leonid Gorb, Ivan Voiteshenko, Vasyl Hurmach, Margarita Zarudnaya, Alex Nyporko, Tetiana Shyryna, Maksym Platonov, Szczepan Roszak, Bakhtiyor Rasulev

**Affiliations:** Department of Molecular and Quantum Biophysics, Institute of Molecular Biology and Genetics, National Academy of Sciences of Ukraine, 150, Akademika Zabolotnoho Str., Kyiv 03143, Ukraine; Department of Molecular and Quantum Biophysics, Institute of Molecular Biology and Genetics, National Academy of Sciences of Ukraine, 150, Akademika Zabolotnoho Str., Kyiv 03143, Ukraine; Taras Shevchenko National University of Kyiv, 60 Volodymyrska Street, Kyiv01033, Ukraine; Department of Molecular and Quantum Biophysics, Institute of Molecular Biology and Genetics, National Academy of Sciences of Ukraine, 150, Akademika Zabolotnoho Str., Kyiv 03143, Ukraine; Department of Molecular and Quantum Biophysics, Institute of Molecular Biology and Genetics, National Academy of Sciences of Ukraine, 150, Akademika Zabolotnoho Str., Kyiv 03143, Ukraine; Taras Shevchenko National University of Kyiv, 60 Volodymyrska Street, Kyiv01033, Ukraine; Department of Molecular and Quantum Biophysics, Institute of Molecular Biology and Genetics, National Academy of Sciences of Ukraine, 150, Akademika Zabolotnoho Str., Kyiv 03143, Ukraine; Department of Molecular and Quantum Biophysics, Institute of Molecular Biology and Genetics, National Academy of Sciences of Ukraine, 150, Akademika Zabolotnoho Str., Kyiv 03143, Ukraine; Faculty of Chemistry, University of Wrocław, 50-370Wrocław, Poland; Department of Coatings and Polymer Materials, North Dakota State University, NDSU Department 2760, PO Box 6050, Fargo, ND 58108, USA

## Abstract

In this computational study, we explore the folding of a particular sequence using various computational tools to produce two-dimensional structures, which are then transformed into three-dimensional structures. We then study the geometry, energetics and dynamics of these structures using full electron quantum-chemical and classical molecular dynamics calculations. Our study focuses on the SARS-CoV-2 RNA fragment GGaGGaGGuguugcaGG and its various structures, including a G-quadruplex and five different hairpins. We examine the impact of two types of counterions (K^+^ and Na^+^) and flanking nucleotides on their geometrical characteristics, relative stability and dynamic properties. Our results show that the G-quadruplex structure is the most stable among the constructed hairpins. We confirm its topological stability through molecular dynamics simulations. Furthermore, we observe that the nucleotide loop consisting of seven nucleotides is the most flexible part of the RNA fragment. Additionally, we find that RNA networks of intermolecular hydrogen bonds are highly sensitive to the surrounding environment. Our findings reveal the loss of 79 old hydrogen bonds and the formation of 91 new ones in the case when the G-quadruplex containing flanking nucleotides is additionally stabilized by Na^+^ counterions.

## Graphical Abstract

**Graphical Abstract:**
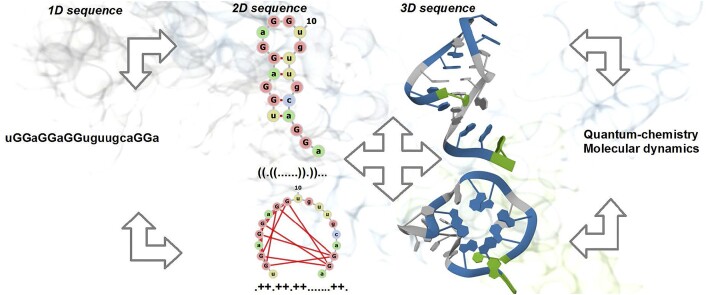

## Introduction

It is currently known that G-rich sequences of nucleic acids can form four-stranded structures called G-quadruplexes. These fragments are completely different from usual DNA or RNA double helixes (1). Recent research indicates the presence of G-quadruplexes in key regions of the human genome such as telomeres (2), promoters of cancer genes (3,4), hot spots of mutations and some non-coding DNAs (5,6). It is expected that >350 000 possible G-quadruplexes are present in the human genome (7). Sometimes the presence G-quadruplexes is associated with various human diseases and cancer (8), and they are potential targets for drug development (9–12). G-quadruplexes are also present in RNA molecules. They are especially enriched in the untranslated regions of mRNAs and introns of pre-mRNAs. Furthermore, RNA G-quadruplexes were found in non-coding RNAs (ncRNAs) such as telomeric RNAs, long ncRNAs and microRNAs [see reviews (11,13)]. While <2000 studies devoted to different aspects of quadruplex chemistry and biology were published in 2022–023 (https://clarivate.com/products/scientific-and-academic-research/research-discovery-and-workflow-solutions/webofscience-platform/web-of-science-core-collection/), an in-depth structural resolution and dynamic studies are needed in this area (14).

Although the human population's rate of infection by severe acute respiratory syndrome coronaviurs 2 (SARS-CoV-2) has significantly decreased (see, for instance, https://www.worldometers.info/coronavirus/), studies of the structural resolution and dynamics of the regulatory elements in its genome remain of permanent interest. Studying G-quadruplexes located in a SARS-CoV-2 genome will accelerate the design of relevant compounds that selectively interact with them.

To date, ∼50 putative G-quadruplex-forming sequences have been identified in the positive RNA strand of the SARS-CoV-2 genome. Thirty-seven such sequences were reported in a review by Zhai *et al*. (15), and a further 10  in the work of Bezzi *et al*. (16). Multiple experimental techniques have confirmed the formation of eight of them (see Table 1).

**Table 1.** G-quadruplexes in RNA of SARS-CoV-2

Position	Gene	Sequence	Methods
353	Nsp 1	GGagacuccguGGaGGaGG	CD (21) fluorescence (21)
644	Nsp 1	GGuaauaaaGGagcuGGuGG	CD (14,21,22), fluorescence (14,21,22), NMR (17), TDS (14)
1574	Nsp 2	GGuguuguuGGagaaGGuuccgaaGG	CD (22), fluorescence (22)
3467	Nsp 3	GGaGGaGGuguugcaGG	CD (14,22–24), electrophoresis (22), fluorescence (14,22), NMR (14,22–24), TDS (14,24)
13 385	Nsp 10	GGuauguGGaaaGGuuauGG	CD (21,22,25–27), electrophoresis (27), fluorescence (21,22,25–27,29,30), UV melting (25)
24 215	S	GGuuGGaccuuuGGugcaGG	CD (21), fluorescence (21)
24 268	S	GGcuuauaGGuuuaauGGuauuGG	CD (21,25), fluorescence (21,25), UV melting (25)
25 197	S	GGccauGGuacauuuGGcuaGG	CD (21), fluorescence (21)
28 903	N	GGcuGGcaauGGcGG	CD (21–23,27–30), electrophoresis (27), fluorescence (21,22,27), FRET (27, 28), NMR (23,27)

CD, circular dichroism spectroscopy; FRET, Förster resonance energy transfer; NMR, nuclear magnetic resonance; spectra; TDS, thermal differential spectra; UV melting, ultraviolet melting.

In addition to experimental investigations, an area of computational research expands the experimentally obtained information. In particular, there is a group of computational programs and web services that can perform folding of an RNA fragment into the secondary (two-dimensional) structure of RNA (17–22). The outcome of those investigations is the different possible variants of an RNA secondary structure and the relative difference of Gibbs free energy between them. However, due to the different parametrization of the methods that estimate those differences, the results obtained when applying those tools to the same RNA fragment do not always correspond to one another. Examples from the authors' own experience are given in the Appendix of the Supplementary data.

A further group of tools are quantum-chemical and classical molecular dynamics (MD) computer software that predict and investigate the three-dimensional structure and dynamics of RNA fragments. It should also be highlighted that some of the information obtained in this way is unique because, currently, it is not possible to study this experimentally. In addition, three-dimensional structures could be verified by the application of experimental techniques such as X-rays and nuclear magnetic resonance (NMR) (7).

Recently, such a combination of quantum-chemical and classical MD investigations has been applied to the SAR-CoV-2 genome position 28 903 (31) (herein it is called RG1). However, only the structure of the corresponding G-quadruplex, which was resolved from the three experimentally observed PDB structures, has been studied. Also, the QM0/MM scheme was applied in the case of quantum-chemical calculations. This does not allow accurate inclusion of the electronic and spatial influence of the loop's G-quadruplex structure. The resolving structure and dynamics of other sequences presented in Table 1 are unknown.

Herein, we present the computational protocol, which includes folding the sequence of interest by different tools that generate two-dimensional structures, converting them into three-dimensional structures and further investigation at the level of full electron quantum-chemical and classical MD calculations. Such combinations lead to the application of quite time-demanding computational techniques to three-dimensional fragments of RNA to obtain the details of their geometry, energetics and dynamics. The drawback of the proposed strategy is still the impossibility of applying it to real-size fragments of RNA at an accurate quantum-chemical level due to the current limitations of modern supercomputers.

**Scheme 1.**
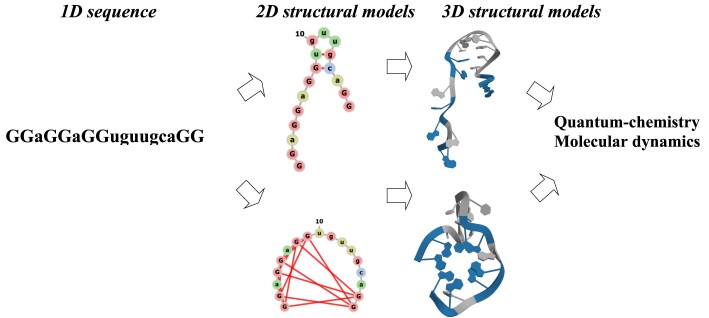
Schematic overview of this research.

The described protocol can be illustrated by Scheme 1.

The protocol presented in Scheme 1 has been applied to convert the sequence of genomic SAR-CoV-2 RNA at position 3467 (see Table 1) to three-dimensional structures such as G-quadruplexes and five different hairpins to study the influence of two types of counterions (K^+^ and Na^+^) and flanking nucleotides on the geometrical characteristics, relative stability and dynamic properties.

## Materials and methods

**Figure 1.**
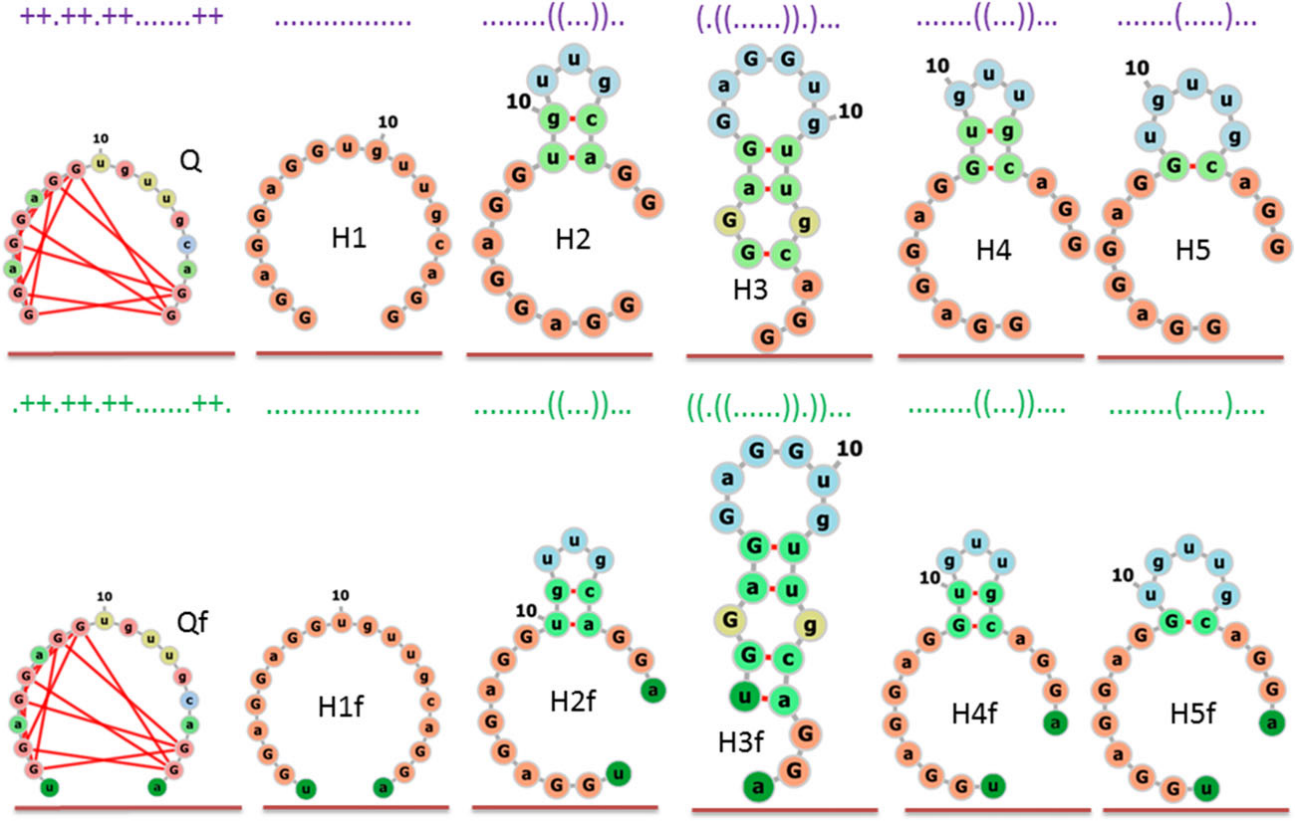
Dot–bracket notations and two-dimensional structures used in this study.

The dot–bracket notations and two-dimensional structures corresponding to those notations are presented in Figure 1.

To represent these, the graphical style of ViennaRNA (22) has been used. The two-dimensional structure of Q was obtained manually. The presence of flanking nucleotide (deep green, second line) is indicated in Figure 1 by the presence of the letter f. To build two-dimensional models of the H1, H2 and H3 hairpins, the options of RNAcomposer (CentroidFold, ContextFold, CONTRAfold, IPKnot, RNAFold and RNA structure) were used with the default parameters. Duplicate conformer structures were discarded. The two structures of the H4 and H5 hairpins were obtained by the UNAFold program (19).

The three-dimensional structures are obtained using the web services 3D-NuS (32) and RNAComposer (33). In the case of the Qf structure, flanking nucleotides were added manually using the VMD graphical interface (36), taking into account the typical values of interatomic bonds, valence and dihedral angles between the atoms. In this case, the investigated RNA fragment was represented by the sequence: uGGaGGaGGuguugcaGGa having flanking nucleotides.

Quantum-chemical calculations were performed using the Gaussian 16 software at the level of electron density functional theory, including the dispersion interactions. The exchange and correlation functional B97-D3 (34) and the 6-31G (d,p) basic set (35) were used. Both of them are well-known computational tools, allowing us to reach a compromise between the time and resources needed to assume quality calculations for such large systems. We also used them for the computational investigations of systems such as d(A)5·d(T)5 and d(G)5·d(C)5 (36). However, given the large size of the system (the smallest RNA fragment had 559 atoms and 6589 basis functions), soft convergence criteria [opt = Loose and IOp(1/18 = 100 000)] reducing the number of redundant internals were used.

The large size of the system also did not allow us to consider parameters such as zero-point vibrational energy, entropy and temperature. Thus, all energy estimates are obtained on the basis of the total energy of the systems. The influence of the water environment was taken into account in the conductor-like polarizable continuum model (CPCM) approximation (dielectric continuum with dielectric constant ϵ = 78.3) (37,38). The presence of counterions that compensate for the charge of the RNA backbone in the CPCM approximation is taken into account automatically through interaction with a positively charged molecular cavity (39). However, we also considered the RNA fragments when a single K^+^ or Na^+^ counterion was inserted into a quadruplex structure, or when they were presented explicitly as interacting with RNA backbone counterions. In the latter case, the location has been chosen randomly.

The analysis of electron density distribution has been performed with the application of the AIMALL package (40) at the B97-D3/6-31G(d,p) level of theory. The presence of a critical bond point (BCP) of (3, –1) type and a positive Laplacian value in this BCP (Δρb > 0) were considered as criteria for the formation of the hydrogen bond The energies of hydrogen bonds are calculated based on the empirical method of Espinoza–Molins–Lecomte (EML): *E*_AH···B_= 0.5·*V*(*r*), where *V*(*r*) is the value of the local potential energy at the critical point of (3, −1) type (41).

The MD simulation was performed to estimate the time stability of the obtained RNA structures. The calculations were done with Gromacs 2021 software (42) using force field Charmm36 (43). In the case of the RNA G-quadruplex, K^+^ was inserted within the quadruplex structure.

Each system was placed into the center of a periodic triclinic box with the next filling by SPC/E water molecules. A minimum 1.5 nm distance was maintained between the nearest atom of the RNA and the edge of the simulation box so that the RNA could fully immerse in water and rotate freely. Before conducting any evaluations, each MD trajectory was subjected to a rotation and translation fit to a reference RNA structure. This step was carried out to eliminate translational and rotational motions of the RNA molecules, allowing subsequent analyses to concentrate on internal molecular motions and interaction (44). To neutralize the system and mimic the cellular environment (pH   7), Na^+^ and Cl^−^ ions were added to bring the ionic concentration to 150 mM. In this process, the solvent molecules are randomly replaced by monoatomic ions. The obtained systems were energy minimized via the steepest descent algorithm (the maximum number of steps was 50 000) and equilibrated in two stages.

The stage of NVT equilibration was performed at 100 ps using a V-rescale thermostat, and the second NPT equilibration of 100 ps with the same thermostat and a Berendsen barostat, appropriately. After that, the MD simulations were launched within 100 ns. All calculations were done at a temperature of 293.16 K and constant atmospheric pressure.

Free energies for ensembles of RNA conformations obtained from MD calculations were calculated using the standard energy operand of the Gromacs software. The reference temperature for free energy calculation corresponded to the temperature during productive MD calculation.

Principal component analysis (PCA) has been utilized to elucidate high-amplitude concerted motions within fragments of genomic RNA of SAR-CoV-2 (45,46). The simulations were executed utilizing eigenvectors derived from the mass-weighted covariance matrix of RNA atomic fluctuations. The build-in gromacs function ‘gmx covar’ was used to generate the covariance matrix; the MD representative RNA structure was used as a reference to evaluate rotation fit during the MD trajectory. Then, to define the dimensionality of the essential subspace, eigenvectors and eigenvalues were calculated. Notably, most of the movements (∼90%) can be characterized using <10 eigenvectors, which reveal the significant coordinated motions occurring within an atomic system. Cosine content is used as a measure of principal components (47–49). If the cosine content is close to 1, it indicates significant movement within the RNA molecule and renders it unsuitable for PCA. However, most snapshots captured during MD simulation exhibit cosine values close to 0.2, with some approaching 0.5, making them suitable for PCA. So, according to the above, by using ‘gmx analyse’, utility FEL (free energy landscape) was constructed utilizing cosine contents < 0.2 of the first two projection eigenvectors (defined as PC1 and PC2, respectively). The most prevalent and energetically favorable structures extracted from the FEL’s minimum energy basins were then utilized for subsequent analyses.

Three-dimensional structures were visualized by the Mol* (50) and PyMol (51) molecular graphic programs.

## Results and Discussion

**Figure 2.**
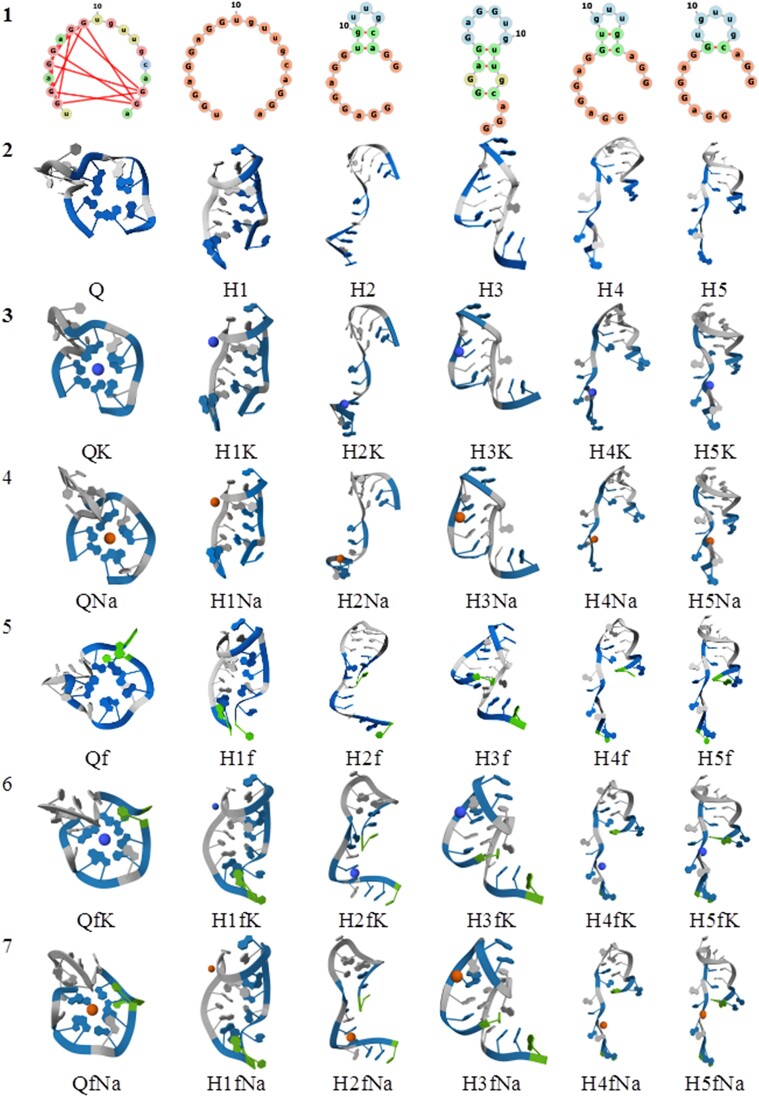
Two- and three-dimensional structures of the investigated RNA fragments (K^+^, filled purple circle; Na^+^, filled red circle; flanking nucleotide, green backbone).

The first line of Figure 2 shows the two-dimensional structures of the quadruplex and five computationally generated hairpins (see Materials and methods). As we have already mentioned, those structures were converted into three-dimensional structures, and their total energy was minimized quantum-chemically. The geometrical structures obtained in this way are collected in lines 2–8 of Figure 2.

Visual analysis of the data presented in Figure 2 mostly indicates a topological similarity between two-dimensional and three-dimensional structures. The most pronounced difference between a quantum-chemically optimized structure and its two-dimensional counterpart seems to be in the case of the structures belonging to family H1. This was expected because hydrogen bonds between nucleotides in H1 are completely disturbed. Also, the three-dimensional geometries presented in Figure 2 do not indicate dramatic changes due to the interaction, neither do they explicitly consider K^+^ and Na^+^ counterions or flanking nucleotides.

To obtain more information on the differences in geometrical structures, we have analyzed two integral structural characteristics, root mean square deviation (RMSD) and molecular cavity volume [the value always used as one of the parameters of a CPCM (37) approximation]. Both parameters are directly related to the change of geometry. The data presented in Table 2 demonstrate a rather insignificant difference in geometrical parameters of the structures denoted in Figure 2 as Q, H1, H3, H4, H5, and the structures denoted as QK, H1K, H3K, H4K, H5K and - QNa, H1Na, H3Na, H4Na, H5Na. K^+^-containing structures (the most frequent counterion presenting between quadruplex planes) demonstrate the smallest deviation between G-tetrads in the quadruplex.

**Table 2.** RMSD in atomic position (Å) of structures presented in Figure 2 as RMSD value

Q	H1	H2	H3	H4	H5
0.00	0.00	0.00	0.00	0.00	0.00
QK	H1K	H2K	H3K	H4K	H5K
1.44	0.25	0.59	0.47	0.30	1.28
QNa	H1Na	H2Na	H3Na	H4Na	H5Na
2.47	2.80	2.76	1.26	0.97	1.17
Qf	H1f	H2f	H3f	H4f	H5f
2.68	4.00	17.32	3.61	2.97	2.64
QfK	H1fK	H2fK	H3fK	H4fK	H5fK
6.59	3.95	17.20	4.23	2.94	2.66
QfNa	H1fNa	H2fNa	H3fNa	H4fNa	H5fNa
6.08	4.00	17.21	5.24	2.86	2.71

It is quite expected that flanking nucleotides will be able, at least to some extent, to deform the structure. The data presented in Figure 2 suggest that the deformation is generally greater than that caused by the interaction with counterions. The greatest is the deformation of the structure H2. The scale of the changes follows from the data presented in Figure 2, where the superposition of related structures has been performed. It is clear to see that SAR-CoV-2 RNA fragments have mostly changed the geometry in the area of flanking nucleotides.

The following notations are used: Q, H1, H2, H3, H4 and H5 denote the structure of the quadruplex and hairpins which do not contain flanking nucleotides and counterions; Qf, H1f, H2f, H3f, H4f and H5f denote the structure of the quadruplex and hairpins which contain flanking nucleotides; QK, H1K, H2K, H3K, H4K, H5K or - QNa, H1Na, H2Na, H3Na, H4Na and H5Na denote the structure of the quadruplex and hairpins with explicit inclusion of K^+^ or Na^+^ counterions; QfK, H1fK, H2fK, H3fK, H4fK, H5fK or -QfNa, H1fNa, H2fNa, H3fNa, H4fNa and H5fNa denote the structure of the quadruplex and hairpins which contain both flanking nucleotides and explicit inclusion of K^+^ or Na^+^ counterions.

Supplementary Table S1 lists the data on the volume of the CPCM molecular cavities. The conclusions derived from the analysis of these values are similar to the data presented in Table 2.

**Figure 3.**
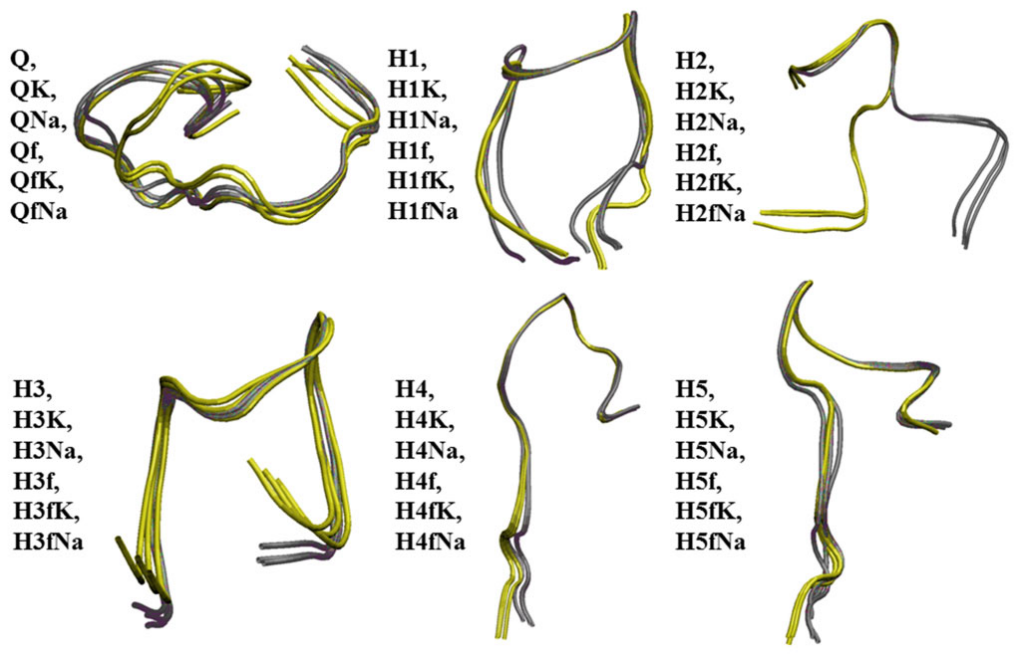
Superposition of the structures presented in Figure 1. The structures with flanking nucleotides are depicted in yellow.

Also, we would like to mention that we did not carefully analyze characteristics of the geometry such as a change of interatomic distances, valence and torsion angles, which are traditionally used [see, for example, such an analysis in (52) when one of the first quantum-chemical modelings of G-quadruplexes was performed]. Supplementary Table S3 contains a full set of Cartesian coordinates for all structures presented in Figure 3.

**Table 3.** Number of hydrogen bonds which disappeared^a^ and corresponding total hydrogen bond energies (kcal/mol)

Notation	*Q*	*QNa*	*QNa*	*Qf*	*QfK*	*QfNa*
**Q**		18^b^	18^b^	24^b^	70^b^	68^b^
		22.96^c^	23.38^c^	61.14^c^	184.20^c^	173.51^c^
**QK**	11^b^		+6^b^	+25^b^	+67^b^	+66^b^
	14.40^c^		8.49^c^	68.00^c^	189.41^c^	178.99^c^
**QNa**	13^b^	8^b^		26^b^	71^b^	69^b^
	17.43^c^	10.31^c^		68.64^c^	194.89^c^	134.26^c^
**Qf**	49^b^	57^b^	56^b^		80^b^	79^b^
	130.04^c^	147.62^c^	145.72^c^		239.32^c^	229.30^c^
**Qf**	108^b^	112^b^	114^b^	93^b^		7^b^
	282.96^c^	314.50^c^	317.12^c^	268.79^c^		6.50^c^
**QfNa**	105^b^	110^b^	111^b^	91^b^	6^b^	
	278.97^c^	311.06^c^	311.34^c^	264.05^c^	5.15^c^	

^a^The loss of the number of hydrogen bonds goes in the direction of structures designated from bold to italic.

^b^Number of hydrogen bonds which disappeared.

^c^Total energy of hydrogen bonds which disappeared.

One may conclude that the presence of flanking nucleotides or explicitly considered K^+^ and Na^+^ counterions does not meaningfully change the geometry of the species considered but makes those changes locally. For instance, the results presented in (53) illustrate the exclusion of one water molecule from the G4 cavity during the penetration of K^+^ ions into the G-quadruplex. However, the absence of profound changes in the structural parameters does not mean the absence of the possible impact of flanking nucleotides and counterions on relative stability.

**Figure 4.**
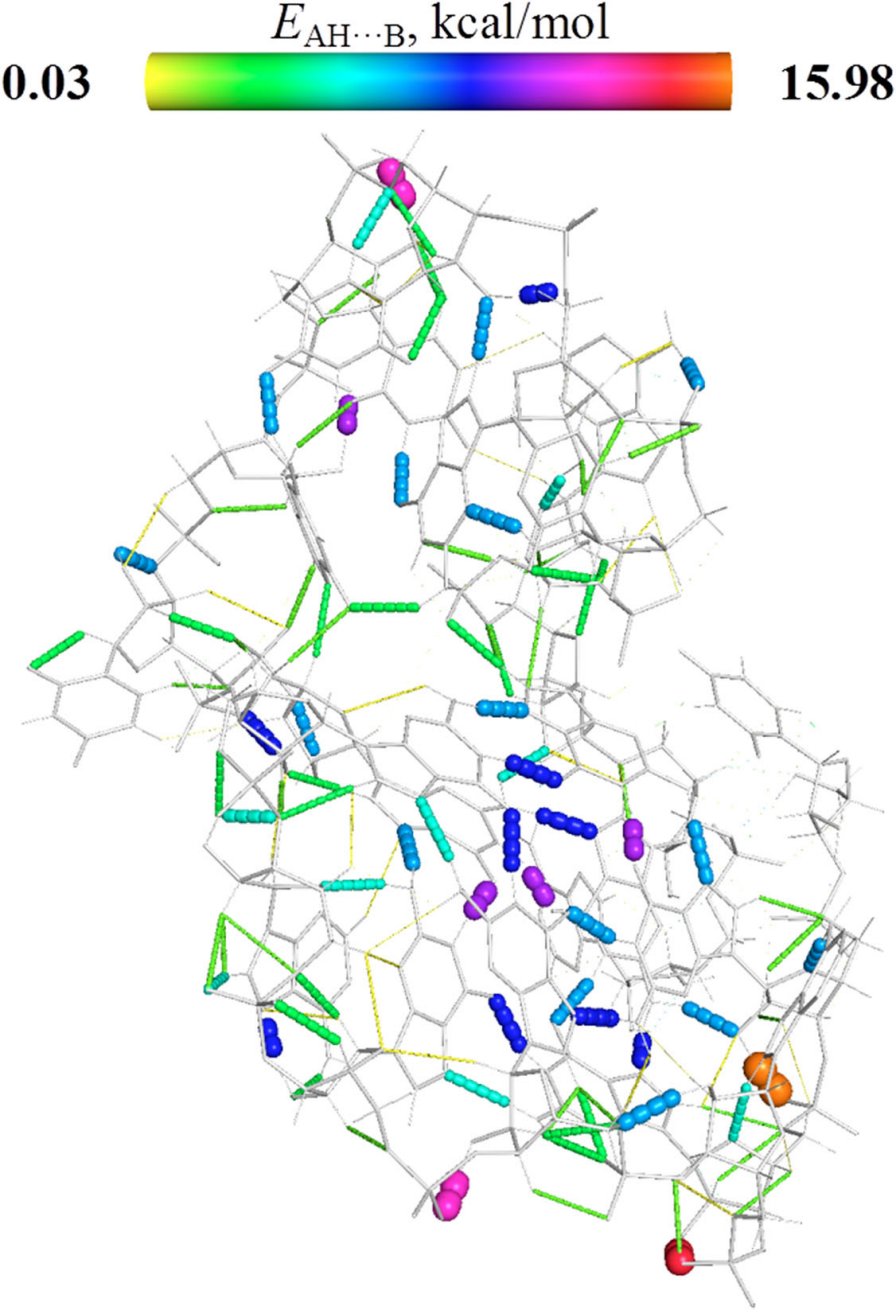
Network of hydrogen bonds. QfK, the fragment most populated by hydrogen bonds.

The ability to form inter- and intramolecular hydrogen bonds is another structural factor which contributes to the dynamics and stability of DNA and RNA strands (54,55), To study this phenomenon, we applied AIM theory and obtained rather surprising results. The Excel file collecting the AIM characteristics of H-bonds found is presented in the Supplementary data. Supplementary Table S2 collects the extraction of the data from those Excel files which are necessary for our analysis. As follows from the data presented in Supplementary Table S2, the RNA fragment most populated by hydrogen bonds is QfK. This structure as well as others is stabilized by a distinct network of H-bonds of different types (NH···O, OH···N, NH···N, CH···O, CH···N, CH···C, CH···Hx) which are presented in Figure 4.

We found that the investigated fragments of RNA accumulate from 322.11 to 679.50 kcal/mol of  intramolecular energy of hydrogen bonds. This amount of energy is much greater than similar estimates performed in (56); however, these authors used more accurate formulas for their estimations. In addition, the RNA fragments we investigated are much larger. Also, the data presented in Supplementary Table S2 suggest that the ratio of intermolecular to intramolecular hydrogen bonds is much greater in G-quadruplexes than in hairpins. This is probably due to their very different geometrical structures.

The total picture of new hydrogen bond formation in the case the RNA fragments most populated by hydrogen bonds (Q, QK, QNa, Qf, QfK and QfNa) is described by the data collected in Table 3.

**Figure 5.**
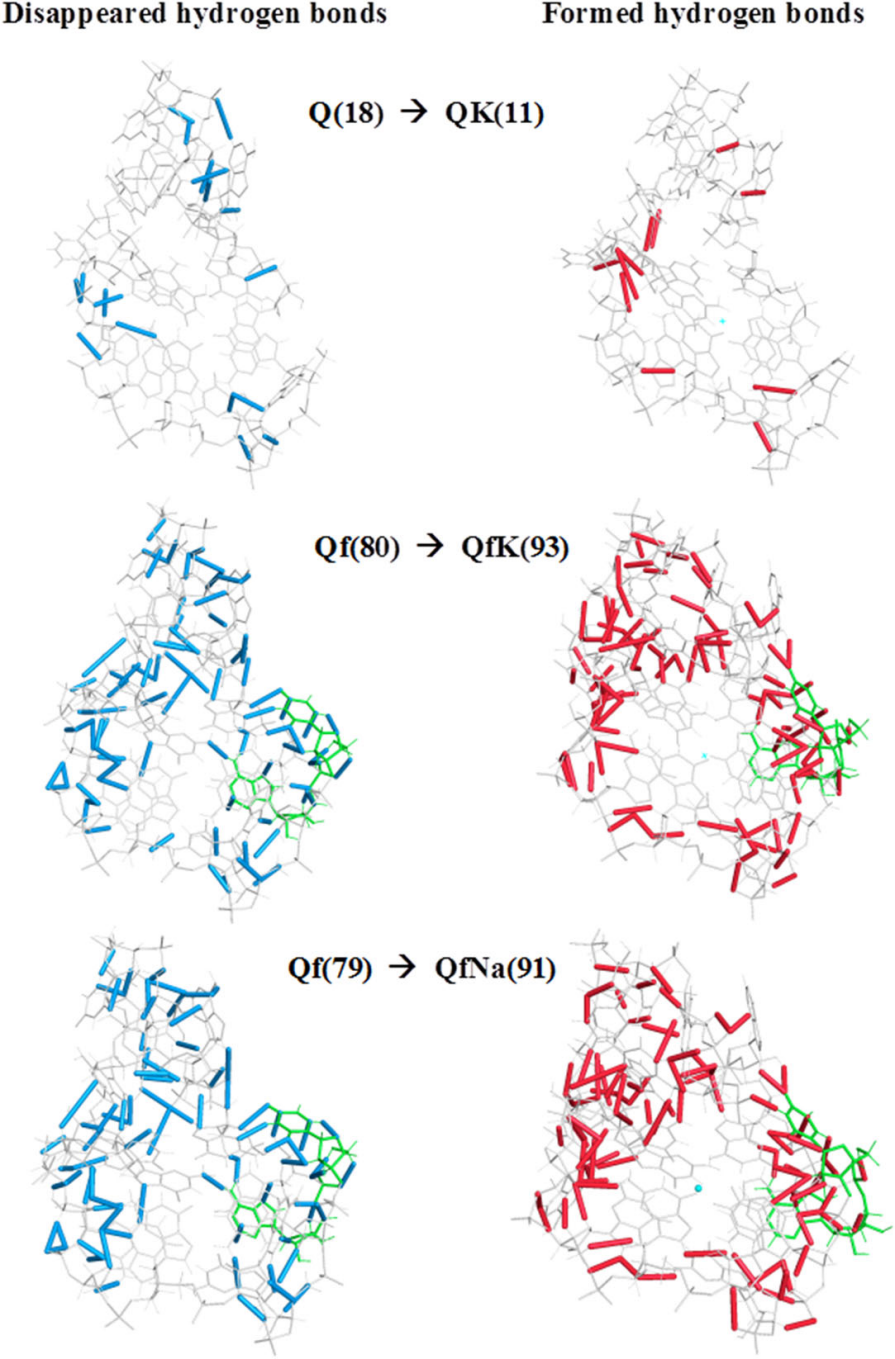
Graphical visualization of some of the transformations presented in Table 3 (the number of hydrogen bonds which are formed or disappear is shown in parentheses). The flanking nucleotides are drawn in green.

These data can shed light on the physical mechanism of forming the above-mentioned distinct networks in each of the SARS-CoV-2 RNA fragments considered. For instance, stabilizing the Q fragment by K^+^ and Na^+^ counterions results in losing of seven and five H-bonds, respectively. At the same time, Q itself loses 18 bonds in both cases, QK itself forms 11 and QNa forms 13. In contrast, when flanking nucleotides are included, stabilization of Qf by K^+^ and Na^+^ counterions increases the total number of bonds by 13 and 12, respectively (see Table 3). At the same time, Qf loses 80 and 79 bonds, QfK forms 93 and QfNa forms 91 (see Supplementary Table S2 for details). The graphical visualization of selected transformations is presented in Figure 5.

Another example describing the transformation of the network of hydrogen bonds due to the addition of flanking nucleotides is presented in Supplementary Figure S1. Adding flanked nucleotides to Q results in 25 new hydrogen bonds that accumulate 68.90 kcal/mol of intramolecular hydrogen energy (see Supplementary Table S2). The additional images in Supplementary Figure S1 suggest that the appearance of 25 new hydrogen bonds is, in fact, the result of the disappearance of 24 and the appearance of 49 new ones.

As far as we know, we are the first to observe the transformation of RNA fragments in such detail. We investigated the influence of factors such as minor extensions to the RNA structure by adding flanking nucleotides and the effect of stabilizing counterions. Through computational evidence, we found that stabilizing RNA’s shape, the distinct networks of hydrogen bonds are significantly sensitive to the environment surrounding the RNA fragment. However, we are aware that this technique needs to be extended in the future by more accurate calculations of energetic values, including MD, and to be supported by experimental data.

**Figure 6.**
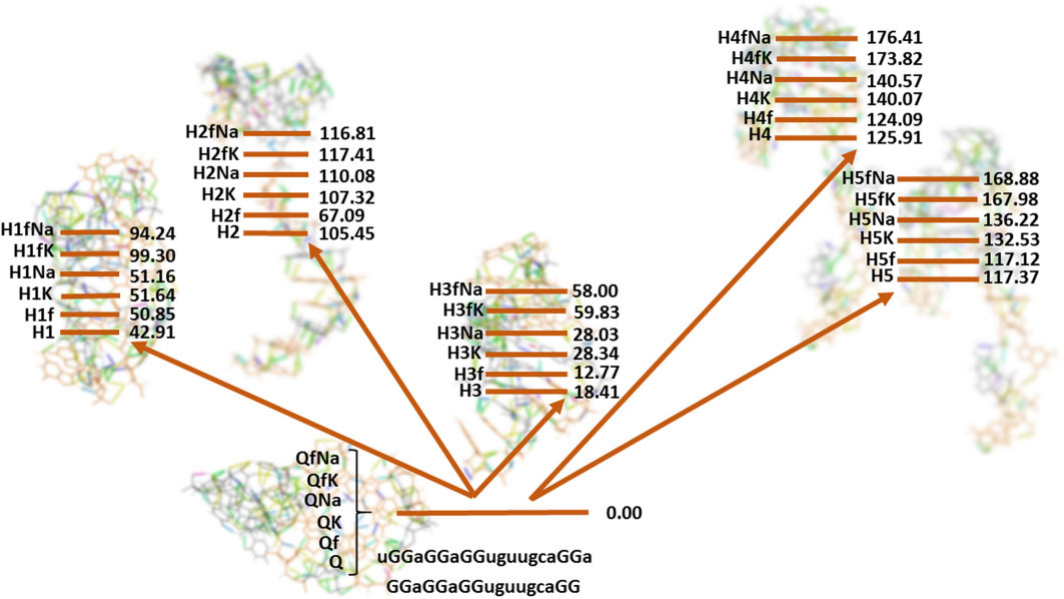
Difference in the total energy (kcal/mol) for the RNA fragments presented in Figure 1.

The relative energy differences considered in the investigation of RNA fragments are presented in Figure 6. We would like to remind readers that all those estimates are based just on the differences in total energy (neither zero-point energy nor contributions from entropy and temperature were considered). In principle, the presented data are in line with the trends already found in the literature (11,57,58). We observe the energetic preference of the RNA fragments, including the quadruplex (Q, QK, QNa, Qf, QfK and QfNa). The most energetically favorable to corresponding quadruplexes is the hairpin H3. As expected, it has the largest number of Watson–Crick and Hoogsteen-type base pairings. Differences in the energy between quadruplexes and some hairpins may seem large. Support for such differences follows from the data of relative Gibbs free energies obtained from molecular dynamics simulations of G-quadruplexes and hairpins (see the data in Table 4). Also unexpected is virtually the same stabilization of hairpins by K^+^ and Na^+^ counterions. However, as mentioned below, an inaccurate estimation of energetic differences could be considered as the explanation for that. Also, at least two more reasons to have such a picture exist: these are the random choice of counterion position in hairpins; and the specific influence of the loops in G-quadruplex structure, which this investigation has not studied in detail.

**Table 4.** Relative Gibbs free energy differences (kcal/mol, T = 293.16 K) obtained from MD simulations

QK	H1	H2	H3	H4	H5
0.0	68.5	65.6	38.7	80.9	95.1

Also, we would like to mention the existence of the correlation between energy differences presented in Figure 5 and the total amount of hydrogen bond energy (see Supplementary Table S2) in each considered species. Such a correlation has negative dependence and correlation coefficients in the range of –0.78 to –0.87 (see Supplementary Figure S2).

The energetic difference between the altered versions of quadruplexes formed from the considered RNA sequence and the possible structures of hairpins completely corresponds to experimental observations. Currently, those observations suggest no evidence of the transition of this particular G-quadruplex to any other structures such as hairpins. Nevertheless, the transitions between quadruplexes and hairpins are very well known in the RNA world, including the quadruplexes formed by other RNA sequences presented in Table 1 [see, for example (16,26)]. This is why we included the MD study of the stability and flexibility of quadruplexes and hairpins presented by lines 3 and QfK from lines 6 of Figure 1. They include K^+^ as the typical RNA counterion stabilizing the structure.

**Figure 7.**
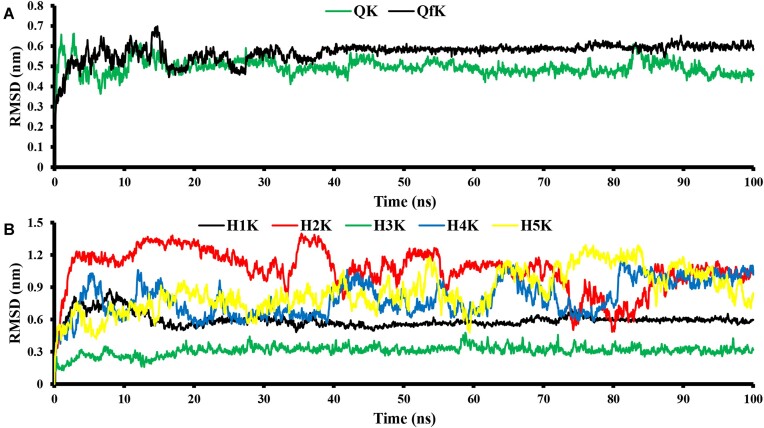
RMSD trajectories of RNA quadruplexes (**A**) and hairpins (**B**).

**Figure 8.**
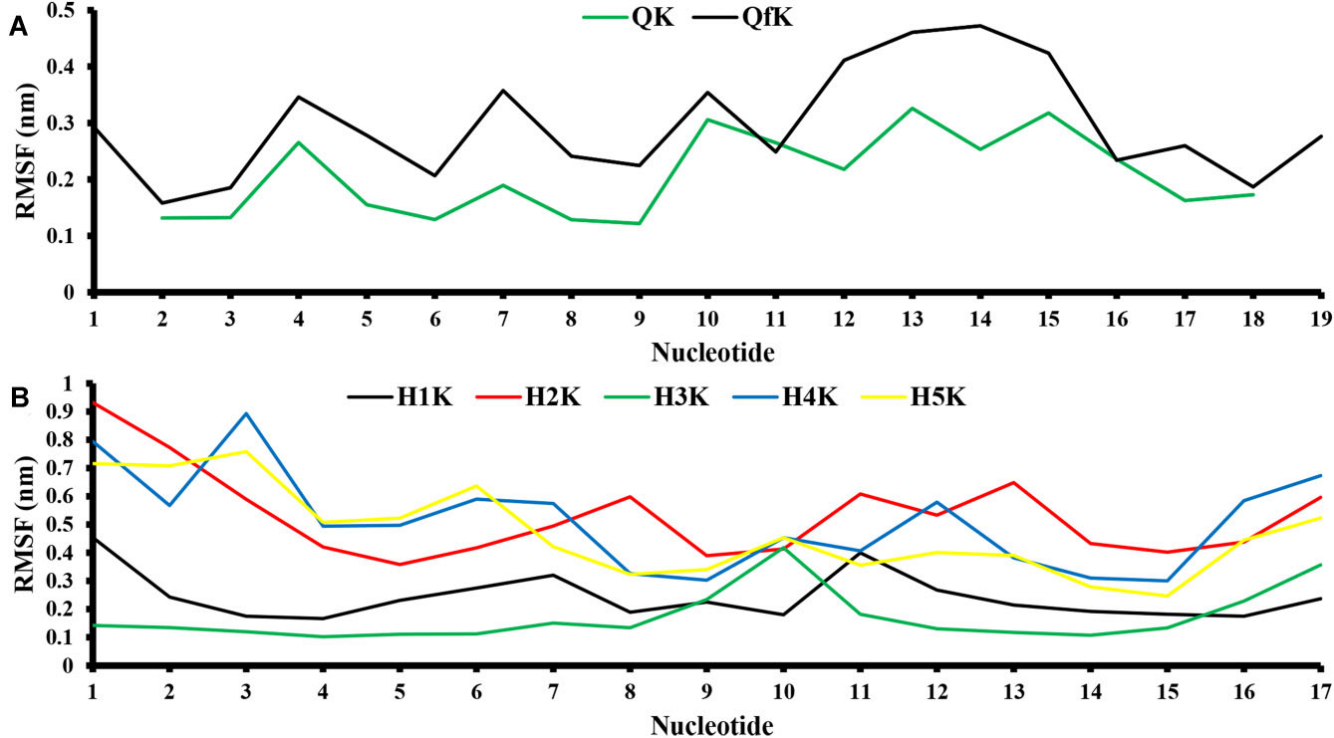
RMSF of the RNA quadruplexes (**A**) and hairpins (**B**).

The MD simulations for QK and QfK (see Figure 7A) suggest that the investigated G-quadruplexes attain equilibrium after ∼15–20 ns of the simulation. The movement of the considered G-quadruplexes remains within the 0.4–0.7 nm range throughout the simulation. However, topologically, the QK and QfK structures maintain the G-quadruplex-like configurations and remain stable. At the same time, the structure of QfK looks more flexible than that of QKs. The root mean square fluctuation (RMSF) profiles presented in Figure 7A enabled identification of the more flexible regions of RNA quadruplexes. The RMSF profiles exhibit similarity, especially for nucleotides 1–9. Also, in the case of nucleotides 11–18, we observe a similar trend in flexibility, but the specific flexibility pattern differs. This disparity may arise from the non-uniform binding of potassium ions to the quadruplex due to varying side chain lengths (see Figures 7A and 8A). To make sure that the obtained results are not force field dependent, similar calculations have been performed at the AMBER20 force field level (see Supplementary Figures S3 and S4 and comments on them).

One should also note that RNA fragments containing G-quadruplexes do not exist alone in the cell but are part of larger RNA molecules. The question of whether the presence of additional nucleotides in the RNA chain stabilizes the quadruplex structure, or vice versa, is still open. To preliminarily verify the stability of the G-quadruplex structure as part of a ‘long’ RNA, an average Gibbs free energy value for the QK and QfK fragments was obtained from the appropriate molecular ensembles. RNA chain extension appeared to decrease the free energy of the fragment containing a G-quadruplex from –2397 kcal/mol, typical for the isolated quadruplex in solution, to –2462 kcal/mol. Thus, we believe that it is correct to assert (or at least confidently assume) that G-quadruplex 3467 in the composition of a long RNA molecule will be even more stable than in the free state under cellular conditions. According to RMSF data (Figure 7A), we can assume that the observed free energy decrease has an entropy nature (increased flexibility of the segment between nucleotides 11 and 16) [see also (59) which is focused on the folding of an RNA G-quadruplex driven by conformational entropy and (60) focused on structural dynamics of RNA G-enriched hairpins].

The same analysis was applied to all RNA structures exhibiting a hairpin conformation. In all cases, the hairpin structures remain stable throughout the MD simulation. According to data presented in Figure 7B, H2K (RMSD ranging from 0.75 to 1.5 nm) and H5K (0.5–1.25 nm) hairpins can be categorized as less flexible than H3K (RMSD ranging from 0.25 to 0.5 nm) and H1K (RMSD ranging from 0.5 to 0.75 nm) (see Figure 8B). This observation is unsurprising since structures such as ‘H2K’, ‘H5K’ and ‘H4K’ exhibit incomplete involvement of all nucleotides in forming the RNA hairpin. Consequently, RNA regions moving in a disorderly fashion in these cases significantly destabilize the system. However, it is noteworthy that in all these less flexible cases, the hairpin structure remains relatively stable throughout the entire MD simulation, indicating significant stability within such structures. Therefore, we can confidently assert that structures ‘H3K’ and ‘H1K’ are substantially more stable than all other RNA structures with a hairpin conformation. This is evident from the RMSD chart (Figure 7B) and is supported by the fact that all nucleotides actively participate in hairpin formation in the case of these two structures.

**Figure 9.**
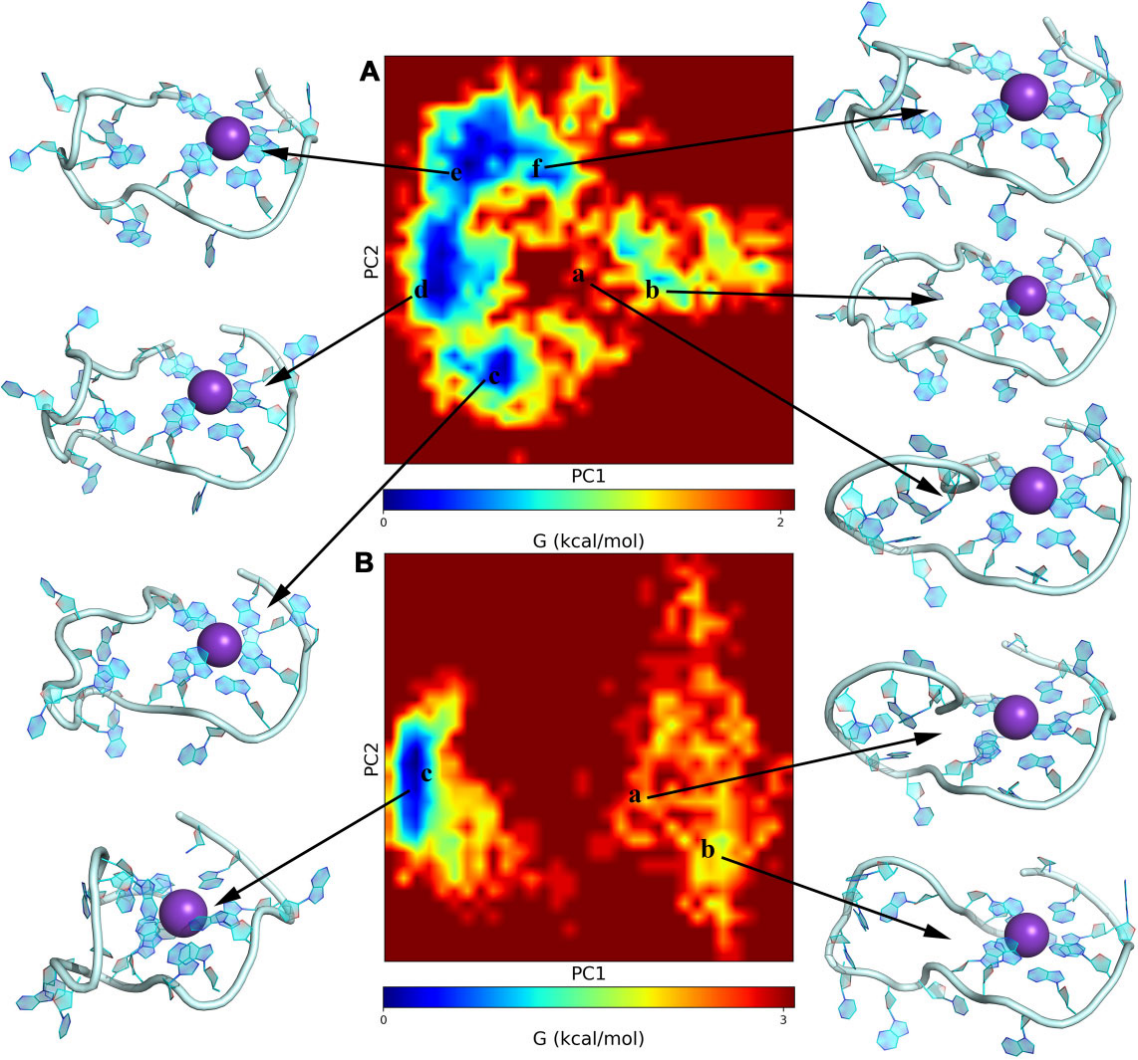
Free energy landscape of QK (**A**) and QfK (**B**) conformational space. A: a - initial conformation; B: a - initial conformation.

One may obtain new details on the flexibility and relative stability of considered RNA fragments by performing a PCA. The free energy landscape of QK and QfK conformational space is depicted in Figure 9. In the case of QK, Figure 9A shows six distinct Gibbs free energy clusters with representative structures (a, b, c, d, e and f). All those structures are slightly different in the shape of the nucleotide loop, keeping the quadruplex configuration stable. The configurations c and d are the most populated. However, since the relative Gibbs free energy of all configurations lies in the interval of 2 kcal/mol, it is natural to assume that the presented in Figure 9A picture simply reflects a motion of the loop with large amplitude.

The conformational space of QfK is limited by just three configurations (see Figure 9B). The obtained data support the conclusion formulated in the previous paragraph regarding the stability of the G-quadruplex configuration and its dominant role during the loop's motion. According to RMSD analysis (see Figure 7), we expect that RNA fragment QfK is more flexible. Therefore, we assume that such flexibility is due to the movements of atoms within the same configuration.

The Gibbs free energy landscape and corresponding representative structures for hairpins H1K–H5K are presented in Supplementary Figure S5. Since none of them is an energy-dominating structure according to quantum-chemical predictions (see Figure 6), we do not place these data in the main text of the paper. We just briefly mention that they exhibit fewer representative structures than QK. This is not surprising because, among the considered structures, the hairpin structures have to be more rigid due to the formation of Watson–Crick and Hoogsteen-like base pairing.

## Conclusions

The presented computational protocol allows for analyzing the static and dynamic properties of the RNA fragments *in silico*. It starts by converting a sequence representation of RNA structure to two- and three-dimensional structures. Obtained in this way, three-dimensional structures are the subject of routine quantum-chemical and classical MD investigations. We assume that such a procedure analyzing the relative stability of three-dimensional RNA fragments is more informative and trustworthy than that which is based on two-dimensional structures [see, for example (61 ,62)].

The application of the above-mentioned protocol to the SARS-CoV-2 RNA fragment GGaGGaGGuguugcaGG led us to the following basic conclusions:

1. The structure of the G-quadruplex has been found to be the most stable fragment compared with the structures of hairpins considered. The pattern of stability does not depend on the presence of flanking nucleotides or on the explicit presence of K^+^ and Na^+^ counterions. The topological stability of all considered SARS-CoV-2 RNA fragments has been confirmed at the MD level. The loop consisting of seven nucleotides is the most flexible structural part of the RNA fragment. Its motion results in the formation of Gibbs free energy clusters with a shape close to that of structures d and f (Figure 8).
2. Intramolecular hydrogen bonds, namely their strength, number, and total hydrogen bond energy, contribute significantly to the total stability of the studied RNA fragments. We found that stabilizing the shape of RNA networks of hydrogen bonds is significantly sensitive to the environment surrounding the RNA fragment. For example, the transition of Qf to QfNa results in the loss of 79 old hydrogen bonds and the formation of 91 new ones.
3. The structural analysis presented above reveals that all considered species are highly flexible. Such complexity makes it difficult to identify a definitive structural target for initiating structure-based design. We presume that the complexity in the motion of nucleotide loops could present significant challenges in the effective design of specific binding agents.

## Supplementary Material

lqae062_Supplemental_Files

## Data Availability

The authors confirm that the data supporting the findings of this study are available within the article and its supplementary materials.
