# Supplementary material for: From RNA sequence to its three-dimensional structure: geometrical structure, stability and dynamics of selected fragments of SARS-CoV-2 RNA
Source: NAR Genom Bioinform. 2024 Jun 4;6(2):lqae062. doi: 10.1093/nargab/lqae062 (PMC11148665; doi:10.1093/nargab/lqae062)
Supplement: lqae062_Supplemental_Files [file lqae062_supplemental_files.zip › LGorb_Suppl Mat_Revised.docx]

**Supplementary data**

**From RNA Sequence to its Three-Dimensional Structure:**

**Geometrical Structure, Stability and Dynamics of Selected
Fragments of RNA SARS-CoV-2**

Leonid Gorb^a^, Ivan Voiteshenko^a,b^, Vasyl Hurmach^a^, Margarita Zarudnaya^a^, Alex. Nyporko^b^, Tetiana Shyryna^a^, Maksim Platonov^a^, Szczepan. Roszak^c^, Bakhtiyor Rasulev^d^

^a^Department of Molecular and Quantum Biophysics, Institute of Molecular Biology and Genetics, National Academy of Sciences of Ukraine, 150, Akademika Zabolotnoho Str., Kyiv, Ukraine, 03143

^b^Taras Shevchenko National University of Kyiv, 60 Volodymyrska Street, Kyiv, Ukraine, 01033

^c^Faculty of Chemistry, University of Wrocław, 50-370 Wrocław, Poland

^d^Department of Coatings and Polymer Materials, North Dakota State University, NDSU Dept. 2760, PO Box 6050, Fargo, ND 58108, USA

**Appendix**

Optimal Domain Structures for RNA fragment: ggaagaagcuaaaaagguaaaaccaacagugguuguuaaugcagccaauguuuaccuuaaacauggaggagguguugcaggagccuuaaauaaggcuacuaacaaugccaugcaaguugaaucugaugauuacauagcuacuaauggaccacuuaaagugggugguaguuguguuuuaagcggacacaaucuugcuaaacacugucuucauguugucggcccaaauguuaacaaaggugaagacauucaacuucuuaagagugcuuaugaaaauuuuaaucagcacgaaguucuacuugcaccauuauuaucagcugguauuuuuggugcugacccuauacauucuuuaagaguuuguguagauacuguucgcacaaaugucuacuuagcugucuuug .

| **RNAfold 2.6.3 (ViennaRNA Web Services)** | **Mfold web server** | **UNAFold** | **RNAstructure (RNAComposer)** |
| --- | --- | --- | --- |
| 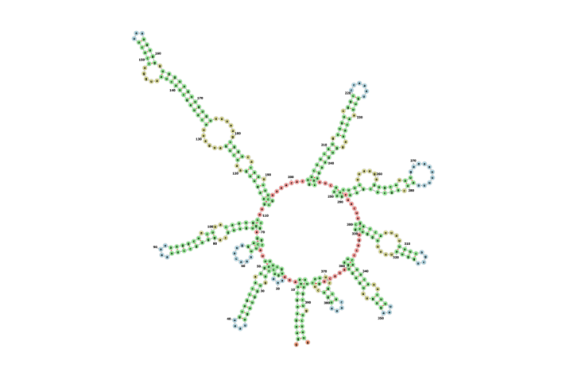 | 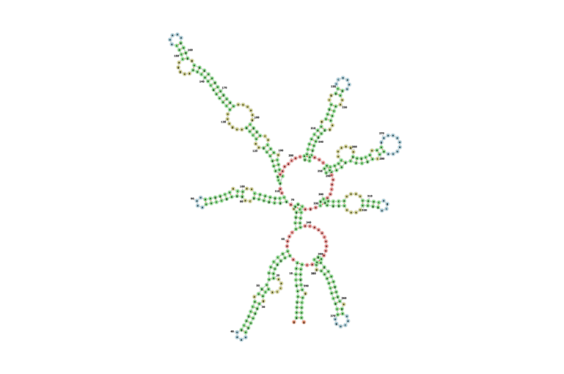 | 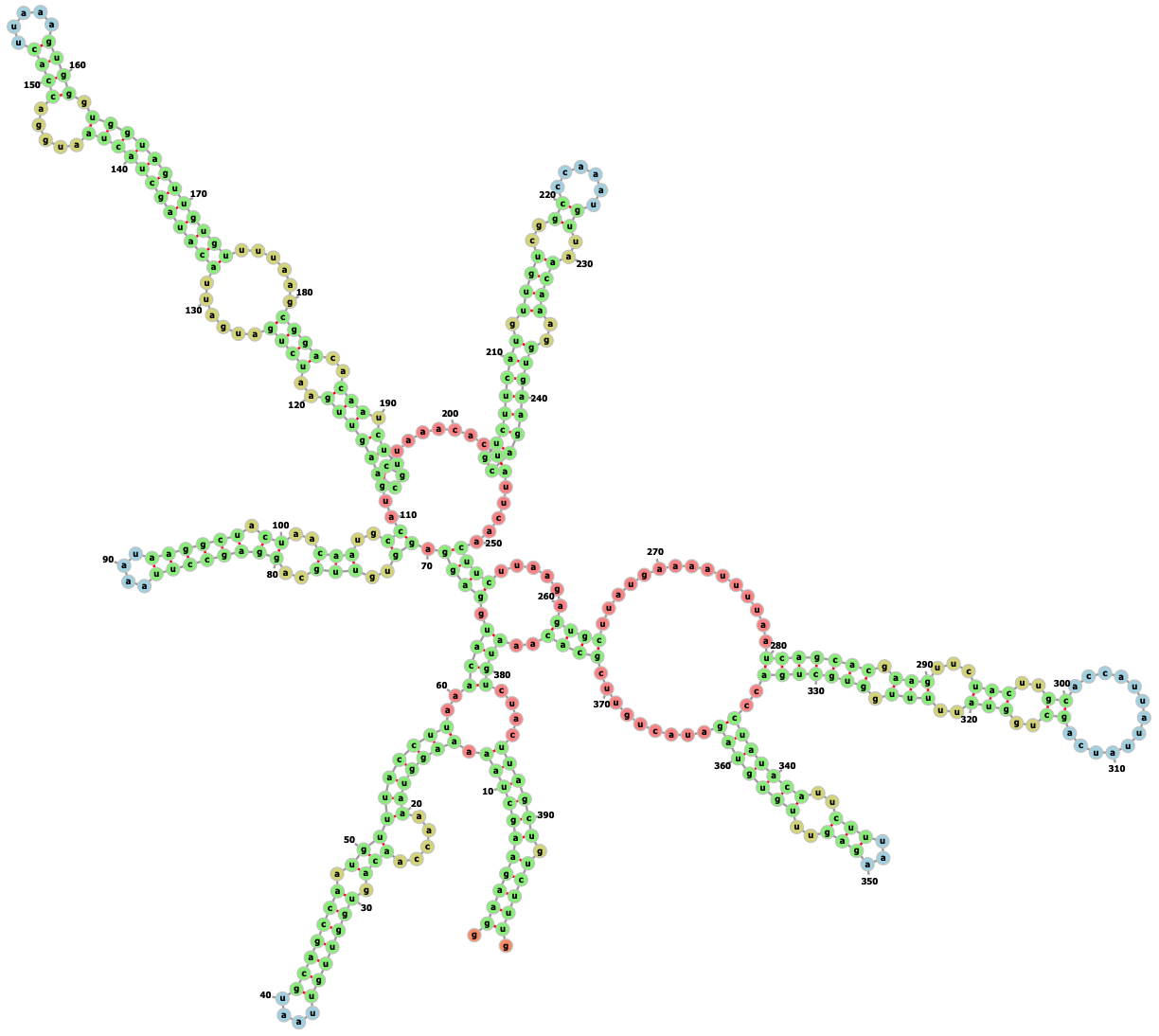 | 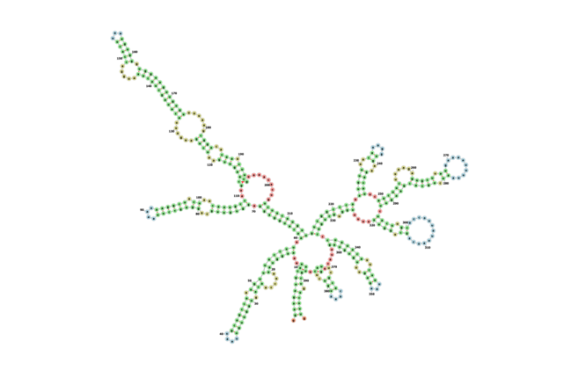 |

The domain sequence was selected in accordance with the genomic RNA model presented by Huston et al [HustonNC, Wan H, Strine MS, de Cesaris Araujo Tavares R, Wilen CB, Pyle AM.Comprehensive invivo secondary structure of the SARS-CoV-2 genome revealsnovel regulatory motifs and mechanisms. Mol Cell. 2021 Feb 4;81(3):584-598.e5.doi: 10.1016/j.molcel.2020.12.041. PMID: 33444546] and confirmed experimentally. From the Figure one can see that the secondary structure created by three different software are different.

**Figures**

| 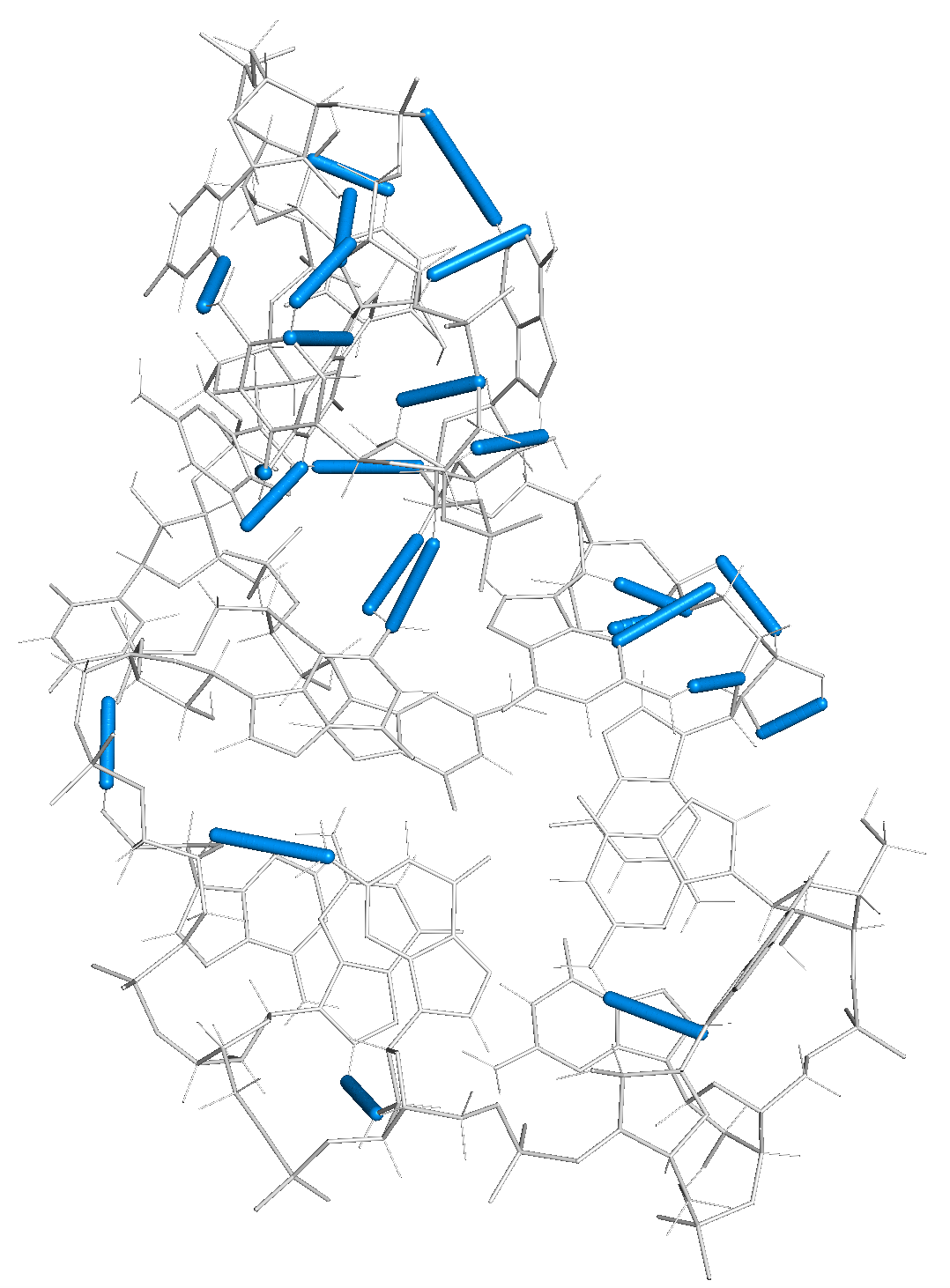  **Q(24) 🡪 Qf(49)** | 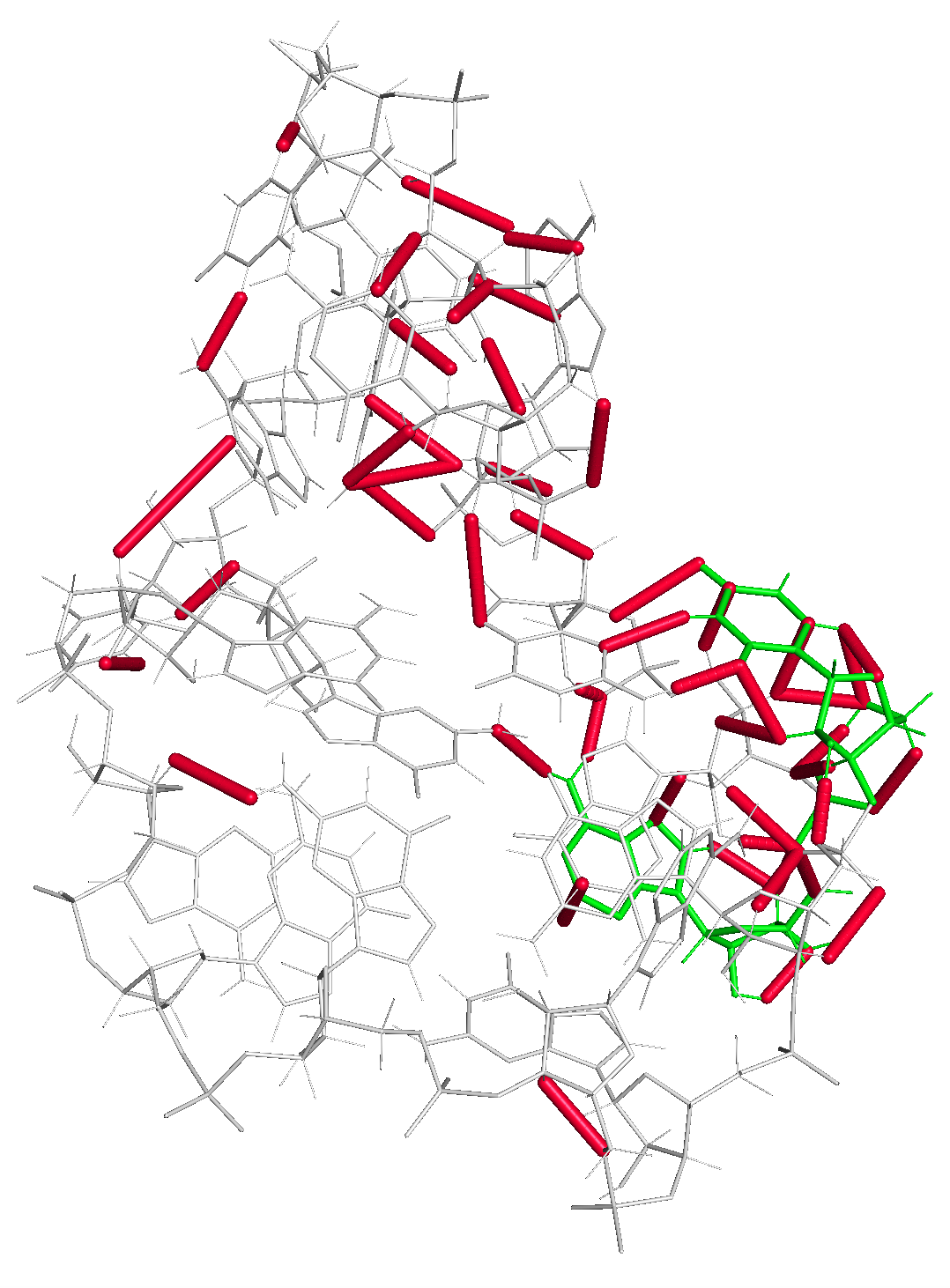 |
| --- | --- |

Figure 1S Formation of distinct net of hydrogen bonds in case of Q to Qf transition.Flanking nucleoides are represented by green color.

| 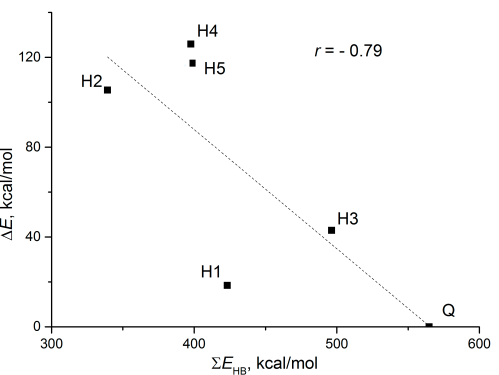 | 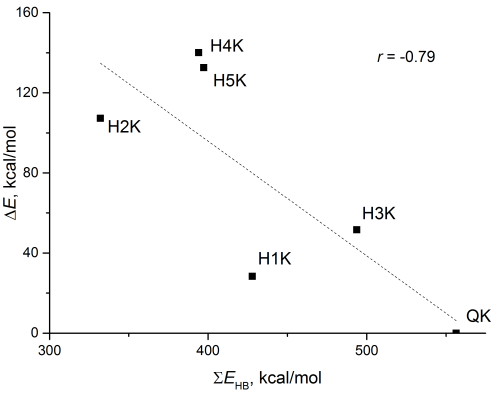 | 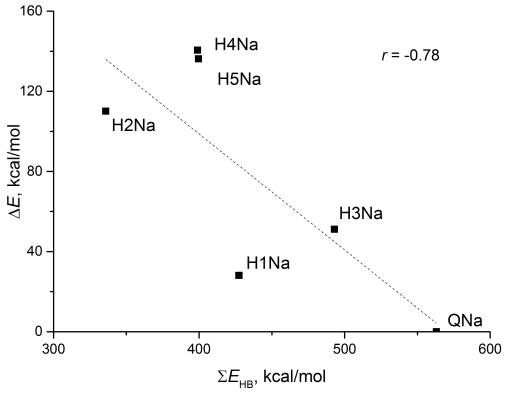 |
| --- | --- | --- |
|  |  |  |
| 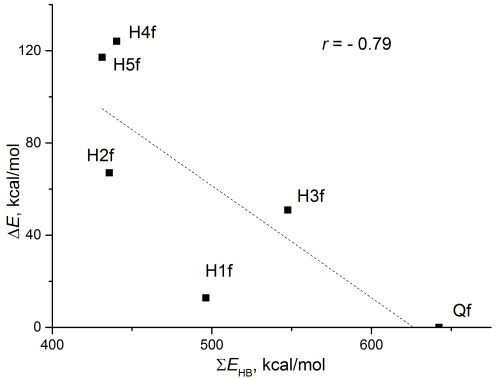 | 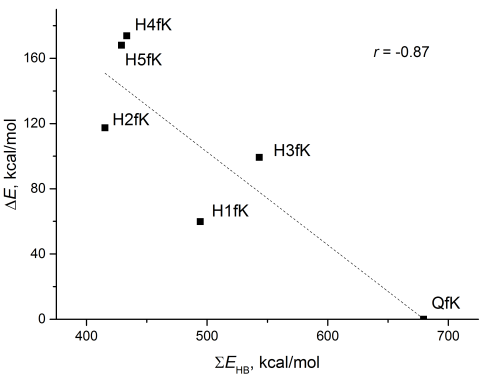 | 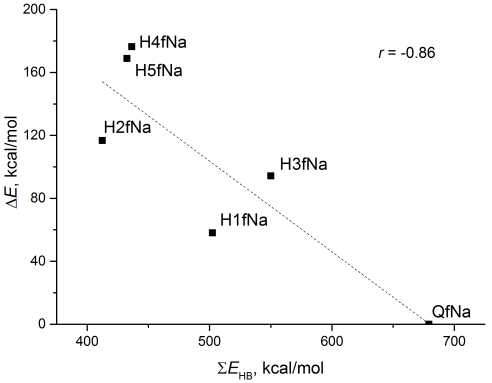 |
|  |  |  |

Figure 2S.Some correlation dependences were found in this investigation.

| 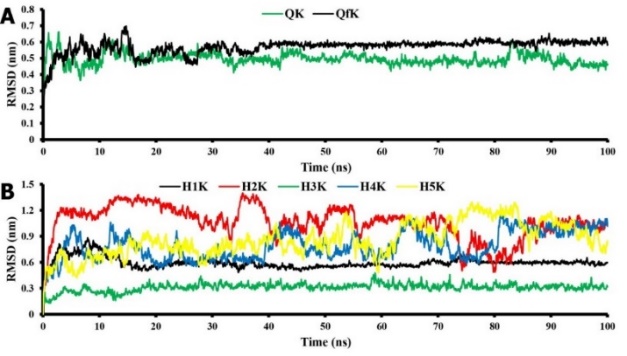 | 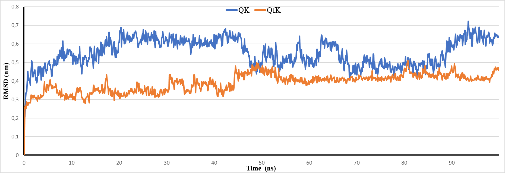  **D**  **C** 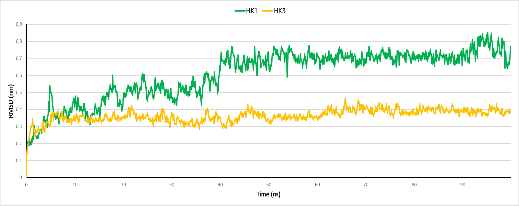 **B** |
| --- | --- |

Figure 3S. RMSD trajectories of RNA quadruplexes (A) and hairpins (B) were calculated within the Charmm36 force field; quadruplexes (C) and hairpins (D) were calculated within the AMBER20 force field.

| 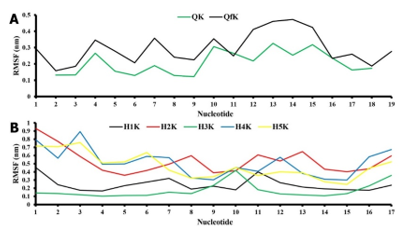 | 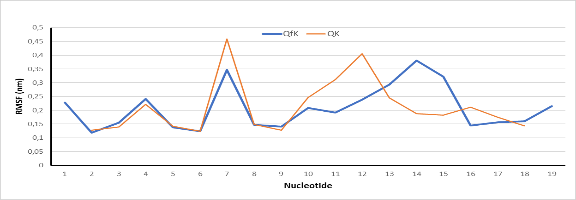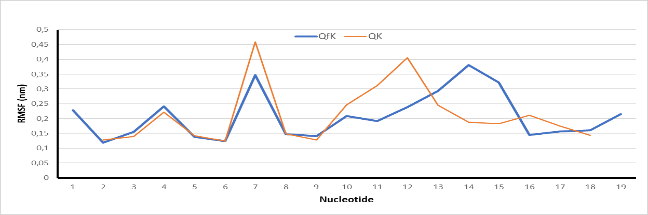  **D**  **C** |
| --- | --- |

Figure 4S. RMSF of RNA quadruplexes (A) and hairpins (B) calculated within Charmm36 force field; quadruplexes (C) and hairpins (D) calculated within AMBER20 force field.

Comments to Figure 4S and 5S.

All structure variants investigated using two force fields tend to stabilise over time, as evidenced by corresponding plateaus in the RMSD plots (Fig. 6, 4S) and appropriate trend lines parallel to the abscissa axis. All mobility plots (profiles) for individual nucleotides in the case of structures containing G-quadruplexes have 4 indicative areas of low mobility corresponding to guanines directly involved in the quadruplex formation (Fig. 7A, 5S). The high similarity of mobility profiles calculated from Charmm and Amber trajectories for the hairpin-containing structures of H1K and H3K is also noteworthy (Fig. 7B, 5S).


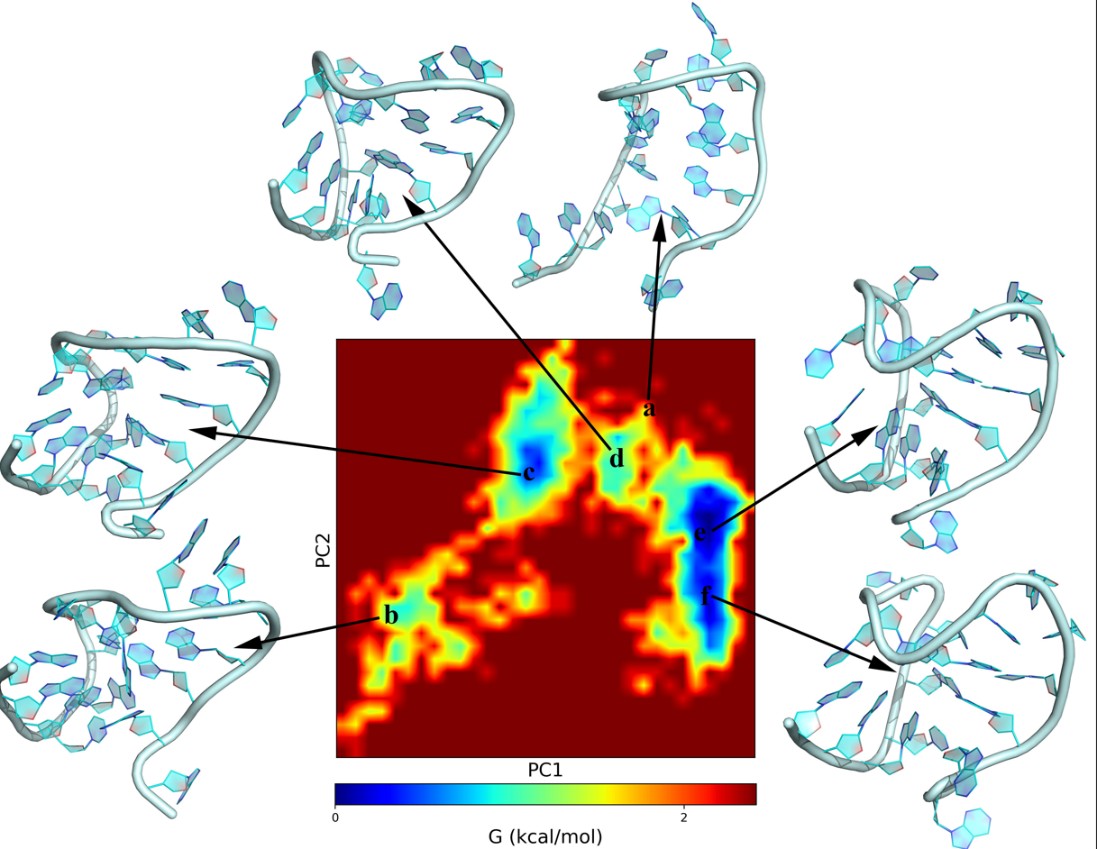


**H1K**

| **H2K**  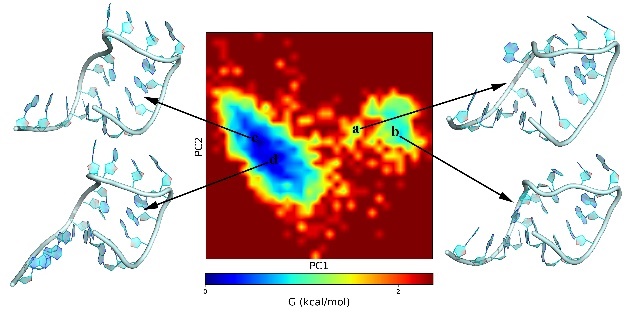 |
| --- |
|  |


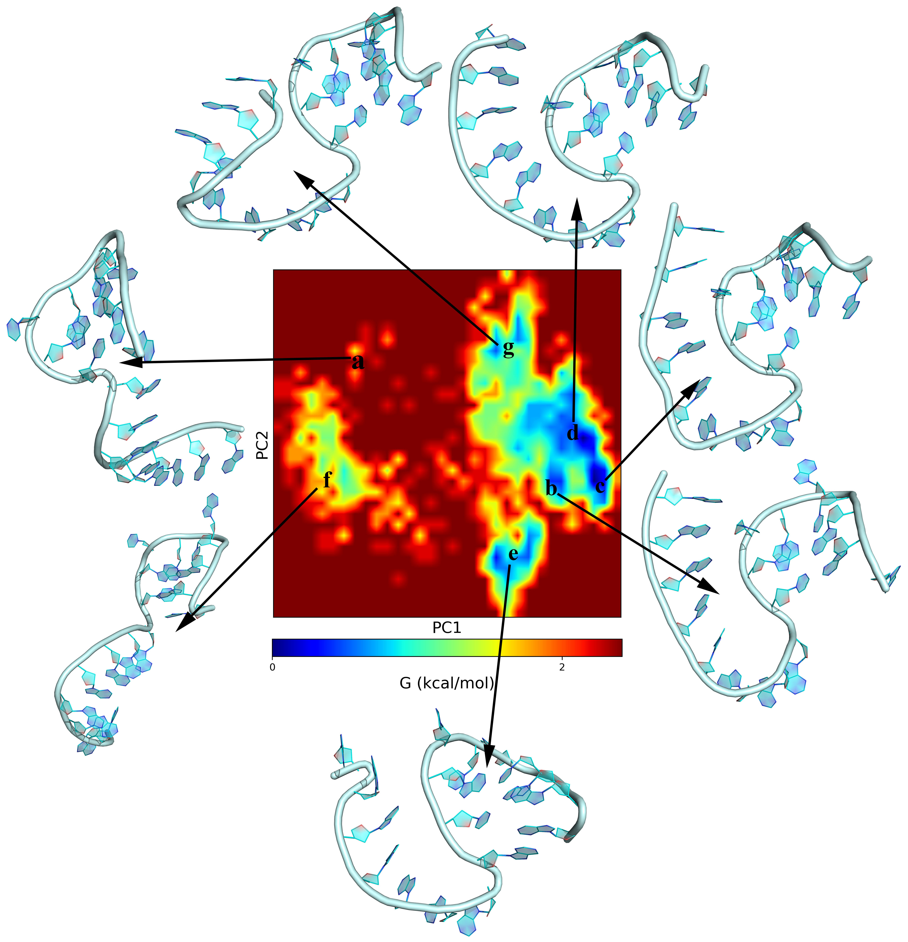


**H3K**


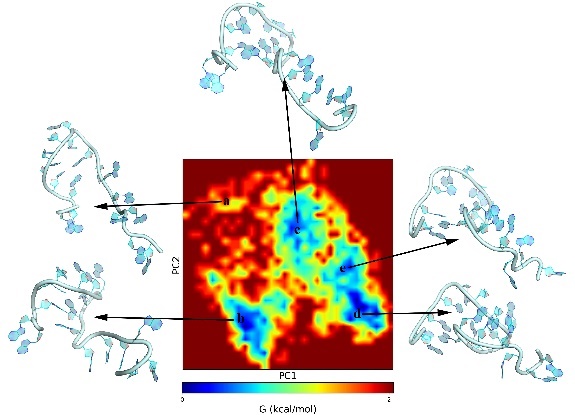


**H4K**


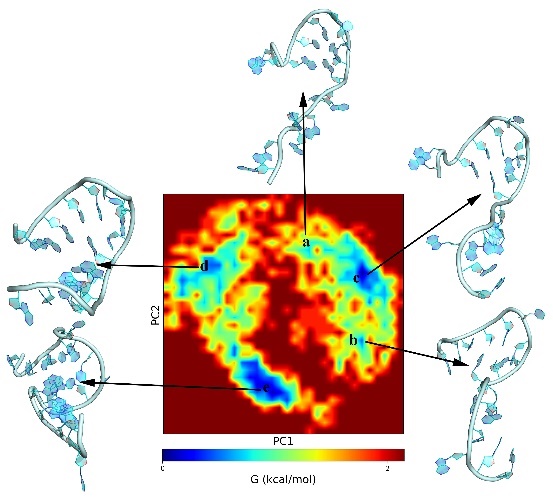


**H5K**

Figure. 5S. Free energy landscape of QK (A)and QfK (B) conformational space a – always initial conformation.

**Tables**

Table 1S The volume of the CPCM molecular cavities

| Volume (Ang**3) | Q | H1 | H2 | H3 | H4 | H5 |
| --- | --- | --- | --- | --- | --- | --- |
| . | 5639.416 | 5654.709 | 5777.296 | 5679.288 | 5795.083 | 5767.489 |
| K^+^ | 5664.102 | 5677.478 | 5789.070 | 5700.133 | 5830.990 | 5798.727 |
| Na^+^ | 5631.261 | 5657.583 | 5771.872 | 5687.398 | 5806.119 | 5779.138 |
| f | 6270.042 | 6307.779 | 6370.410 | 6285.545 | 6449.813 | 6416.144 |
| fK^+^ | 6240.693 | 6329.619 | 6402.517 | 6289.092 | 6468.051 | 6440.820 |
| fNa^+^ | 6221.823 | 6311.120 | 6394.025 | 6274.832 | 6450.323 | 6417.885 |

Table 2S The AIM characteristics of selected hydrogen bonds

| Name | Σ*_E_*_AH···B_, kcal/mol | Num*_E_*_AH···B_ | Avg*_E_*_AH···B_ | Σ*_E_*_AH···B_, kcal/mol | Num*_E_*_AH···B_ | Avg*_E_*_AH···B_ | Σ*_E_*_AH···B_, kcal/mol | Num*_E_*_AH···B_ | Avg*_E_*_AH···B_ |
| --- | --- | --- | --- | --- | --- | --- | --- | --- | --- |
| uGGaGGaGGuguugcaGGa |  | | | K+ | | | Na+ | | |
| Qf | 642.36 | 173 | 3.71 | 679.50 | 186 | 3.65 | 679.21 | 185 | 3.67 |
| O2*, O3*, O4*, O5* | 344.92 | 81 | 4.26 | 367.93 | 88 | 4.18 | 360.10 | 86 | 4.19 |
| O1P, O2P | 154.70 | 34 | 4.55 | 186.10 | 38 | 4.90 | 184.95 | 38 | 4.87 |
| intra nucleotide | 152.73 | 48 | 3.18 | 141.51 | 46 | 3.08 | 142.99 | 47 | 3.04 |
| between nucleotides | 489.63 | 125 | 3.92 | 537.99 | 140 | 3.84 | 536.22 | 138 | 3.89 |
| H4f | 440.31 | 133 | 3.31 | 433.48 | 130 | 3.33 | 436.47 | 136 | 3.21 |
| O2*, O3*, O4*, O5* | 271.46 | 65 | 4.18 | 270.30 | 64 | 4.22 | 268.00 | 65 | 4.12 |
| O1P, O2P | 97.73 | 24 | 4.07 | 104.93 | 25 | 4.20 | 96.81 | 24 | 4.03 |
| intra nucleotide | 174.44 | 50 | 3.49 | 161.05 | 48 | 3.36 | 171.70 | 51 | 3.37 |
| between nucleotides | 265.82 | 83 | 3.20 | 272.43 | 82 | 3.32 | 264.75 | 85 | 3.11 |
| H5f | 431.25 | 141 | 3.06 | 429.13 | 140 | 3.07 | 432.52 | 139 | 3.11 |
| O2*, O3*, O4*, O5* | 264.56 | 65 | 4.07 | 262.28 | 64 | 4.10 | 260.46 | 63 | 4.13 |
| O1P, O2P | 118.13 | 29 | 4.07 | 116.57 | 28 | 4.16 | 116.41 | 27 | 4.31 |
| intra nucleotide | 170.04 | 47 | 3.62 | 171.92 | 47 | 3.66 | 171.67 | 46 | 3.73 |
| between nucleotides | 261.21 | 94 | 2.78 | 257.21 | 93 | 2.77 | 260.85 | 93 | 2.80 |
| H1f | 547.56 | 165 | 3.32 | 543.22 | 163 | 3.33 | 550.04 | 166 | 3.31 |
| O2*, O3*, O4*, O5* | 263.25 | 66 | 3.99 | 262.50 | 65 | 4.04 | 261.84 | 67 | 3.91 |
| O1P, O2P | 112.37 | 25 | 4.49 | 109.32 | 24 | 4.56 | 114.87 | 25 | 4.59 |
| intra nucleotide | 167.64 | 43 | 3.90 | 163.60 | 42 | 3.90 | 170.96 | 44 | 3.89 |
| between nucleotides | 379.92 | 122 | 3.11 | 379.82 | 121 | 3.14 | 379.07 | 122 | 3.11 |
| H2f | 435.80 | 141 | 3.09 | 415.37 | 142 | 2.93 | 412.41 | 139 | 2.97 |
| O2*, O3*, O4*, O5* | 315.04 | 80 | 3.94 | 302.71 | 81 | 3.74 | 301.73 | 80 | 3.77 |
| O1P, O2P | 31.53 | 10 | 3.15 | 30.87 | 9 | 3.43 | 31.05 | 9 | 3.45 |
| intra nucleotide | 163.92 | 39 | 4.20 | 164.67 | 42 | 3.92 | 164.34 | 41 | 4.01 |
| between nucleotides | 271.89 | 102 | 2.67 | 250.70 | 100 | 2.51 | 248.06 | 98 | 2.53 |
| H3f | 496.31 | 161 | 3.08 | 494.34 | 160 | 3.09 | 502.37 | 158 | 3.18 |
| O2*, O3*, O4*, O5* | 255.85 | 70 | 3.65 | 254.23 | 71 | 3.58 | 260.93 | 68 | 3.84 |
| O1P, O2P | 78.14 | 19 | 4.11 | 78.83 | 20 | 3.94 | 85.26 | 20 | 4.26 |
| intra nucleotide | 174.59 | 47 | 3.71 | 165.87 | 44 | 3.77 | 168.74 | 43 | 3.92 |
| between nucleotides | 321.72 | 114 | 2.82 | 328.47 | 116 | 2.83 | 333.63 | 115 | 2.90 |
| GGaGGaGGuguugcaGG |  | | | K+ | | | Na+ | | |
| Q | 564.74 | 148 | 3.82 | 556.40 | 141 | 3.95 | 563.07 | 143 | 3.94 |
| O2*, O3*, O4*, O5* | 301.26 | 70 | 4.30 | 299.42 | 65 | 4.61 | 295.92 | 66 | 4.48 |
| O1P, O2P | 134.53 | 27 | 4.98 | 138.90 | 28 | 4.96 | 141.19 | 29 | 4.87 |
| intra nucleotide | 141.01 | 42 | 3.36 | 141.14 | 41 | 3.44 | 142.85 | 42 | 3.40 |
| between nucleotides | 423.73 | 106 | 4.00 | 415.27 | 100 | 4.15 | 420.23 | 101 | 4.16 |
| H4 | 397.74 | 128 | 3.11 | 394.03 | 129 | 3.05 | 399.06 | 129 | 3.09 |
| O2*, O3*, O4*, O5* | 234.87 | 61 | 3.85 | 232.88 | 61 | 3.82 | 235.40 | 61 | 3.86 |
| O1P, O2P | 70.24 | 19 | 3.70 | 68.23 | 19 | 3.59 | 67.99 | 18 | 3.78 |
| intra nucleotide | 174.91 | 48 | 3.64 | 172.01 | 48 | 3.58 | 171.82 | 48 | 3.58 |
| between nucleotides | 222.82 | 80 | 2.79 | 222.02 | 81 | 2.74 | 227.24 | 81 | 2.81 |
| H5 | 399.22 | 129 | 3.09 | 397.26 | 130 | 3.06 | 399.71 | 130 | 3.07 |
| O2*, O3*, O4*, O5* | 231.69 | 59 | 3.93 | 228.86 | 60 | 3.81 | 229.12 | 59 | 3.88 |
| O1P, O2P | 94.93 | 25 | 3.80 | 93.49 | 25 | 3.74 | 92.56 | 25 | 3.70 |
| intra nucleotide | 168.45 | 44 | 3.83 | 178.75 | 47 | 3.80 | 178.02 | 48 | 3.71 |
| between nucleotides | 230.77 | 85 | 2.71 | 218.51 | 83 | 2.63 | 221.69 | 82 | 2.70 |
| H1 | 496.36 | 157 | 3.16 | 493.35 | 156 | 3.16 | 492.96 | 157 | 3.14 |
| O2*, O3*, O4*, O5* | 217.48 | 54 | 4.03 | 214.61 | 53 | 4.05 | 213.48 | 54 | 3.95 |
| O1P, O2P | 117.48 | 29 | 4.05 | 118.11 | 29 | 4.07 | 117.86 | 29 | 4.06 |
| intra nucleotide | 155.80 | 43 | 3.62 | 155.44 | 43 | 3.61 | 152.29 | 42 | 3.63 |
| between nucleotides | 340.57 | 114 | 2.99 | 337.91 | 113 | 2.99 | 340.66 | 115 | 2.96 |
| H2 | 339.22 | 105 | 3.23 | 332.11 | 103 | 3.22 | 335.89 | 105 | 3.20 |
| O2*, O3*, O4*, O5* | 241.24 | 62 | 3.89 | 233.26 | 60 | 3.89 | 234.88 | 60 | 3.91 |
| O1P, O2P | 23.05 | 7 | 3.29 | 22.27 | 7 | 3.18 | 22.52 | 7 | 3.22 |
| intra nucleotide | 161.81 | 37 | 4.37 | 160.36 | 38 | 4.22 | 161.87 | 38 | 4.26 |
| between nucleotides | 177.41 | 68 | 2.61 | 171.75 | 65 | 2.64 | 174.02 | 67 | 2.60 |
| H3 | 423.31 | 130 | 3.26 | 427.81 | 133 | 3.22 | 427.40 | 133 | 3.21 |
| O2*, O3*, O4*, O5* | 230.18 | 60 | 3.84 | 233.56 | 62 | 3.77 | 235.11 | 62 | 3.79 |
| O1P, O2P | 51.12 | 10 | 5.11 | 54.39 | 11 | 4.94 | 54.42 | 13 | 4.19 |
| intra nucleotide | 165.03 | 42 | 3.93 | 156.44 | 40 | 3.91 | 154.89 | 39 | 3.97 |
| between nucleotides | 258.28 | 88 | 2.93 | 271.37 | 93 | 2.92 | 272.51 | 94 | 2.90 |

Table 3S Cartesian coordinates

| **uGGaGGaGGuguugcaGGa** | | | |
| --- | --- | --- | --- |
| **Name** | **f** | **fK** | **fNa** |
| **Q** | 1 4.479521000 -10.999497000 2.195523000  8 4.037033000 -11.672931000 1.638929000  6 5.032371000 -12.284742000 0.824288000  1 4.623608000 -13.247922000 0.487973000  1 5.951192000 -12.480312000 1.400328000  6 5.414524000 -11.456291000 -0.402334000  1 6.154987000 -12.017025000 -0.992731000  8 4.246777000 -11.255409000 -1.249925000  6 4.045018000 -9.902848000 -1.549846000  1 3.903879000 -9.775752000 -2.630572000  7 2.760842000 -9.427638000 -0.911320000  6 1.907481000 -10.291015000 -0.269283000  1 2.315132000 -11.281262000 -0.105643000  6 2.375560000 -8.123519000 -1.211902000  8 3.088222000 -7.383729000 -1.902738000  7 1.157419000 -7.729742000 -0.698862000  1 0.931690000 -6.732556000 -0.789115000  6 0.210835000 -8.547614000 -0.042922000  8 -0.873188000 -8.073787000 0.319443000  6 0.668766000 -9.908407000 0.152470000  1 0.017553000 -10.606274000 0.667563000  6 5.293160000 -9.109653000 -1.107420000  1 5.020152000 -8.164253000 -0.622834000  8 6.055598000 -8.902487000 -2.283602000  1 6.901913000 -8.508241000 -1.976276000  6 5.994093000 -10.064279000 -0.095203000  1 5.762033000 -9.740523000 0.926063000  8 7.416975000 -10.152475000 -0.263513000  8 7.802232000 -8.130056000 1.345569000  15 8.437359000 -8.926812000 0.234439000  8 9.815624000 -9.535348000 0.303664000  8 8.356332000 -7.913345000 -1.136098000  6 9.399694000 -7.957547000 -2.128431000  1 8.912515000 -8.000002000 -3.112383000  1 10.018418000 -8.853416000 -1.993126000  6 10.293755000 -6.722244000 -2.087596000  1 11.028838000 -6.819114000 -2.899344000  8 9.507208000 -5.524198000 -2.335061000  6 9.548303000 -4.644134000 -1.196697000  1 10.230243000 -3.805979000 -1.376879000  7 8.218227000 -4.072397000 -1.056927000  6 7.887532000 -2.723521000 -1.179435000  7 8.731015000 -1.676112000 -1.152529000  6 8.125402000 -0.503620000 -1.388753000  7 8.857065000 0.624320000 -1.450792000  1 9.849184000 0.535327000 -1.286791000  1 8.458683000 1.553330000 -1.690966000  7 6.760625000 -0.381403000 -1.592325000  1 6.328868000 0.544752000 -1.777633000  6 5.849777000 -1.459212000 -1.622244000  8 4.649929000 -1.239887000 -1.893105000  6 6.493968000 -2.705724000 -1.359576000  7 5.982910000 -4.000443000 -1.357752000  6 7.025281000 -4.789168000 -1.174035000  1 7.017913000 -5.871311000 -1.134896000  6 10.040859000 -5.531485000 -0.034550000  1 9.203074000 -6.161667000 0.300799000  8 10.551497000 -4.780127000 1.040143000  1 11.478587000 -5.116044000 1.261135000  6 11.037611000 -6.444805000 -0.775657000  1 11.270456000 -7.359561000 -0.218198000  8 12.233357000 -5.736824000 -1.165747000  15 13.493234000 -5.457497000 -0.108775000  8 12.991390000 -5.621139000 1.324388000  8 14.719838000 -6.185098000 -0.594580000  8 13.655105000 -3.817463000 -0.335045000  6 13.962395000 -3.354975000 -1.665027000  1 13.088085000 -3.499369000 -2.315545000  1 14.809398000 -3.923674000 -2.077827000  6 14.326350000 -1.877839000 -1.620774000  1 14.631377000 -1.563590000 -2.626912000  8 13.147834000 -1.111765000 -1.252751000  6 13.372714000 -0.362244000 -0.062349000  1 13.540889000 0.698098000 -0.285397000  7 12.168998000 -0.429784000 0.746421000  6 11.257055000 0.600227000 0.953609000  7 11.355243000 1.869980000 0.511858000  6 10.283941000 2.620495000 0.824151000  7 10.273000000 3.922132000 0.470021000  1 10.935048000 4.197388000 -0.254480000  1 9.478028000 4.534900000 0.692172000  7 9.188812000 2.130494000 1.519443000  1 8.299189000 2.670745000 1.525067000  6 9.051140000 0.797379000 1.975228000  8 7.998816000 0.423202000 2.534714000  6 10.212592000 0.019232000 1.686416000  7 10.460960000 -1.329127000 1.900267000  6 11.620599000 -1.574640000 1.319451000  1 12.094264000 -2.544338000 1.232121000  6 14.620924000 -0.989550000 0.613265000  1 14.291659000 -1.880124000 1.164245000  8 15.291408000 -0.151022000 1.510057000  1 15.557642000 0.644158000 0.959033000  6 15.429369000 -1.529670000 -0.600716000  1 15.979063000 -2.431288000 -0.308714000  8 16.448028000 -0.659175000 -1.125321000  15 16.300499000 0.960052000 -1.487006000  8 15.794989000 1.712147000 -0.256809000  8 17.585739000 1.343269000 -2.170872000  8 15.025752000 0.981588000 -2.552476000  6 15.289677000 1.148715000 -3.951884000  1 15.848277000 0.290417000 -4.354547000  1 15.889309000 2.053938000 -4.115979000  6 13.976960000 1.298381000 -4.701430000  1 14.194091000 1.729268000 -5.695811000  8 13.330703000 0.022384000 -4.858826000  6 11.983672000 0.294261000 -5.261996000  1 11.899389000 0.392316000 -6.355460000  7 11.182814000 -0.851562000 -4.887876000  6 10.377963000 -1.604846000 -5.734552000  7 10.068403000 -1.362975000 -7.023096000  6 9.244215000 -2.314500000 -7.507030000  1 8.948999000 -2.195443000 -8.552899000  7 8.729138000 -3.393295000 -6.885189000  6 9.062542000 -3.608251000 -5.581174000  7 8.529823000 -4.668547000 -4.936448000  1 8.870981000 -4.937947000 -4.011646000  1 7.995477000 -5.333320000 -5.479173000  6 9.938904000 -2.687599000 -4.948517000  7 10.470889000 -2.616940000 -3.664665000  6 11.197246000 -1.510800000 -3.661997000  1 11.779010000 -1.137618000 -2.825969000  6 11.574830000 1.659872000 -4.635798000  1 10.813306000 1.517586000 -3.851254000  8 11.091150000 2.477116000 -5.686414000  1 10.978089000 3.372907000 -5.298390000  6 12.914534000 2.165241000 -4.014214000  1 12.914606000 1.968811000 -2.937627000  8 13.223525000 3.536256000 -4.269618000  15 12.480066000 4.744948000 -3.403082000  8 13.362345000 5.961314000 -3.507595000  8 11.965336000 4.191207000 -2.087695000  8 11.099743000 4.954830000 -4.361460000  6 10.998253000 6.074397000 -5.265483000  1 10.515086000 5.693846000 -6.174743000  1 11.995386000 6.455672000 -5.520360000  6 10.144399000 7.218300000 -4.709478000  1 10.070757000 7.978422000 -5.501456000  8 8.799861000 6.762276000 -4.407390000  6 8.611549000 6.711914000 -2.983261000  1 7.884843000 7.470535000 -2.670154000  7 8.038416000 5.415602000 -2.630944000  6 6.683484000 5.092947000 -2.687786000  7 5.659392000 5.923211000 -2.978296000  6 4.465723000 5.316423000 -2.941314000  7 3.351176000 6.022874000 -3.263077000  1 3.501914000 7.021496000 -3.319086000  1 2.415386000 5.731649000 -2.943397000  7 4.299431000 3.977390000 -2.652092000  1 3.338774000 3.586304000 -2.603408000  6 5.348780000 3.074602000 -2.367569000  8 5.104219000 1.872116000 -2.140376000  6 6.619615000 3.722651000 -2.396955000  7 7.895433000 3.209387000 -2.192539000  6 8.718152000 4.232279000 -2.350980000  1 9.798594000 4.179444000 -2.288840000  6 10.000597000 6.975601000 -2.356759000  1 10.561594000 6.036418000 -2.382341000  8 9.956629000 7.455874000 -1.038926000  1 10.590777000 8.227384000 -0.984584000  6 10.647201000 7.876052000 -3.421572000  1 11.739419000 7.878309000 -3.342661000  8 10.125571000 9.222523000 -3.423891000  15 10.579517000 10.309773000 -2.240583000  8 11.499532000 9.609205000 -1.244537000  8 10.976343000 11.592690000 -2.925678000  8 9.144103000 10.502044000 -1.422955000  6 7.973826000 10.839837000 -2.181751000  1 7.922070000 10.221990000 -3.089325000  1 8.003224000 11.899928000 -2.481892000  6 6.719382000 10.595590000 -1.358953000  1 5.871777000 10.968564000 -1.947880000  8 6.549350000 9.165024000 -1.143193000  6 6.362015000 8.926081000 0.256758000  1 5.294540000 8.970855000 0.499309000  7 6.789725000 7.585926000 0.589622000  6 5.930052000 6.497375000 0.516012000  7 4.633740000 6.552238000 0.178371000  6 4.021991000 5.364743000 0.260468000  7 2.719016000 5.268294000 -0.085299000  1 2.272424000 6.182327000 -0.147638000  1 2.134433000 4.559971000 0.389966000  7 4.680676000 4.209362000 0.634840000  1 4.144760000 3.314641000 0.663212000  6 6.045015000 4.118843000 0.979135000  8 6.537379000 3.014771000 1.303696000  6 6.692122000 5.389894000 0.905607000  7 7.997946000 5.781744000 1.208576000  6 8.020927000 7.090628000 1.008573000  1 8.882737000 7.728230000 1.128376000  6 7.106092000 10.069749000 0.958623000  1 8.187203000 9.921824000 0.839063000  8 6.839297000 10.202826000 2.335316000  1 5.852287000 10.210888000 2.477569000  6 6.724601000 11.260566000 0.035606000  1 7.476224000 12.057385000 0.090199000  8 5.480807000 11.844883000 0.472107000  15 4.025519000 11.889693000 -0.342516000  8 4.164955000 12.319529000 -1.787084000  8 3.102217000 12.614399000 0.616661000  8 3.650307000 10.267058000 -0.268862000  6 3.215877000 9.489836000 -1.383277000  1 3.890852000 8.628581000 -1.461656000  1 3.264978000 10.079611000 -2.309610000  6 1.792742000 8.985698000 -1.180228000  1 1.536193000 8.370560000 -2.051461000  8 1.659653000 8.119032000 -0.021633000  6 1.153344000 8.842414000 1.102788000  1 0.346508000 8.248660000 1.545001000  7 2.206852000 8.949817000 2.133345000  6 2.801845000 7.812738000 2.689115000  7 2.307606000 6.566927000 2.745221000  6 3.201534000 5.706276000 3.271684000  1 2.865093000 4.671458000 3.353523000  7 4.469552000 5.943953000 3.666966000  6 4.938679000 7.215573000 3.590188000  7 6.240155000 7.468613000 3.927960000  1 6.645375000 8.311219000 3.520805000  1 6.846414000 6.655718000 3.908765000  6 4.057787000 8.232690000 3.159723000  7 4.215449000 9.604400000 2.952139000  6 3.108259000 9.987641000 2.333284000  1 2.921411000 11.002896000 2.000276000  6 0.633768000 10.212324000 0.573745000  1 1.303537000 11.026782000 0.863457000  8 -0.663423000 10.533254000 1.040624000  1 -1.289149000 10.152848000 0.393246000  6 0.721258000 10.054817000 -0.971561000  1 0.943328000 11.007171000 -1.464081000  8 -0.517137000 9.522378000 -1.454085000  15 -1.625276000 10.496121000 -2.278330000  8 -1.977306000 9.803635000 -3.571785000  8 -1.148907000 11.927777000 -2.215689000  8 -2.865216000 10.319619000 -1.162447000  6 -4.047012000 9.570478000 -1.485067000  1 -4.159337000 9.492700000 -2.573681000  1 -4.897939000 10.133966000 -1.075430000  6 -4.080393000 8.175825000 -0.871721000  1 -5.127032000 7.831060000 -0.892360000  8 -3.279675000 7.248990000 -1.660426000  6 -2.650044000 6.349143000 -0.754252000  1 -3.313656000 5.525634000 -0.444908000  7 -1.510923000 5.733346000 -1.405227000  6 -1.169399000 4.391943000 -1.306449000  7 -1.891452000 3.434570000 -0.700429000  6 -1.285363000 2.238953000 -0.699946000  7 -1.900085000 1.202300000 -0.091269000  1 -2.659134000 1.466415000 0.524972000  1 -1.445251000 0.283197000 0.062681000  7 -0.070837000 2.011264000 -1.319399000  1 0.387310000 1.080718000 -1.239349000  6 0.705849000 2.996110000 -1.978451000  8 1.797225000 2.678276000 -2.498172000  6 0.072759000 4.277560000 -1.950558000  7 0.483181000 5.517527000 -2.440226000  6 -0.474686000 6.363513000 -2.092101000  1 -0.510803000 7.426442000 -2.295396000  6 -2.287807000 7.249801000 0.444999000  1 -1.495659000 7.919863000 0.089514000  8 -1.804058000 6.587496000 1.588166000  1 -2.562228000 6.231716000 2.110873000  6 -3.584565000 8.076087000 0.592206000  1 -3.396423000 9.051814000 1.051748000  8 -4.593181000 7.372185000 1.328837000  15 -4.633654000 7.438597000 3.010190000  8 -3.671071000 8.479414000 3.522085000  8 -6.091811000 7.373590000 3.388138000  8 -3.898825000 5.941500000 3.315757000  6 -4.703564000 4.789734000 2.945946000  1 -4.828373000 4.760245000 1.857712000  1 -5.683265000 4.869182000 3.427137000  6 -4.074154000 3.500633000 3.415870000  1 -4.827579000 2.710990000 3.301738000  8 -2.925036000 3.163530000 2.573029000  6 -1.949582000 2.543114000 3.391957000  1 -2.135273000 1.466220000 3.543367000  7 -0.669894000 2.655127000 2.715748000  6 0.279767000 1.652087000 2.564508000  7 0.223508000 0.395422000 3.055864000  6 1.304583000 -0.329587000 2.737481000  7 1.440718000 -1.604599000 3.181646000  1 0.592249000 -2.073420000 3.482798000  1 2.063223000 -2.235760000 2.637941000  7 2.359198000 0.161246000 1.995200000  1 3.237201000 -0.395519000 1.896478000  6 2.437722000 1.451188000 1.447493000  8 3.435403000 1.777070000 0.767342000  6 1.295448000 2.233699000 1.787056000  7 0.975468000 3.544208000 1.463094000  6 -0.196828000 3.772495000 2.030012000  1 -0.768262000 4.688529000 1.945936000  6 -2.047818000 3.298490000 4.742003000  1 -1.562745000 4.276434000 4.612629000  8 -1.451657000 2.637568000 5.836071000  1 -2.166124000 2.071887000 6.188682000  6 -3.572184000 3.503477000 4.874537000  1 -3.817392000 4.434937000 5.392473000  8 -4.080167000 2.370840000 5.610891000  15 -5.643732000 2.487479000 6.221396000  8 -6.204640000 1.086231000 6.301271000  8 -6.342835000 3.628514000 5.512099000  8 -5.257359000 3.019811000 7.759571000  6 -5.958047000 2.483777000 8.889748000  1 -5.260048000 2.540114000 9.736543000  1 -6.231535000 1.433533000 8.720957000  6 -7.222403000 3.261777000 9.253844000  1 -7.609280000 2.830893000 10.190069000  8 -6.871403000 4.651883000 9.484043000  6 -7.730766000 5.514461000 8.750954000  1 -8.490846000 5.972161000 9.395714000  7 -6.925548000 6.631175000 8.234600000  6 -5.916632000 6.381327000 7.322238000  1 -5.836330000 5.347172000 6.993242000  6 -7.144720000 7.914387000 8.761318000  8 -8.013112000 8.175459000 9.594631000  7 -6.290075000 8.878170000 8.248584000  1 -6.435277000 9.815855000 8.610664000  6 -5.243338000 8.723423000 7.309036000  8 -4.571405000 9.704599000 6.972660000  6 -5.095177000 7.356585000 6.847950000  1 -4.338324000 7.138389000 6.102526000  6 -8.405629000 4.671909000 7.641688000  1 -7.802616000 4.677192000 6.728116000  8 -9.710246000 5.125749000 7.321583000  1 -10.278487000 4.656034000 7.965314000  6 -8.376746000 3.246790000 8.231610000  1 -8.232167000 2.489562000 7.455234000  8 -9.638785000 3.047030000 8.897464000  15 -10.252459000 1.473888000 8.844301000  8 -9.149243000 0.499253000 9.175139000  8 -11.566098000 1.542268000 9.583411000  8 -10.502976000 1.317511000 7.198471000  6 -11.586769000 2.053477000 6.607309000  1 -11.344537000 3.126570000 6.584582000  1 -12.500918000 1.909365000 7.201402000  6 -11.832254000 1.573346000 5.180976000  1 -12.826534000 1.924509000 4.863456000  8 -10.828870000 2.141325000 4.291100000  6 -10.270596000 1.072923000 3.507597000  1 -10.869304000 0.874880000 2.608809000  7 -8.951211000 1.471460000 3.050011000  6 -8.716888000 2.236036000 1.917889000  7 -9.654220000 2.775774000 1.106438000  6 -9.138872000 3.415705000 0.063419000  7 -9.973431000 3.941744000 -0.886247000  1 -10.967453000 3.934619000 -0.621381000  1 -9.656701000 4.753559000 -1.402982000  7 -7.778286000 3.529649000 -0.152342000  1 -7.444272000 3.973094000 -1.003255000  6 -6.757072000 2.966208000 0.661364000  8 -5.563165000 3.057907000 0.309237000  6 -7.318104000 2.309910000 1.798979000  7 -6.705645000 1.592155000 2.818149000  6 -7.697656000 1.120139000 3.549913000  1 -7.569368000 0.560224000 4.467002000  6 -10.320305000 -0.138274000 4.468238000  1 -9.647732000 0.088908000 5.310028000  8 -9.982291000 -1.381887000 3.921791000  1 -10.813033000 -1.907363000 3.763417000  6 -11.756863000 0.044206000 5.008146000  1 -11.923120000 -0.482730000 5.955273000  8 -12.729909000 -0.362697000 4.021658000  15 -13.358308000 -1.910103000 4.190403000  8 -12.238075000 -2.907421000 3.929965000  8 -14.184411000 -1.979174000 5.454383000  8 -14.317308000 -1.948836000 2.836175000  6 -15.298171000 -0.927022000 2.609028000  1 -15.008456000 -0.007567000 3.133331000  1 -16.283030000 -1.245705000 2.985271000  6 -15.379367000 -0.601273000 1.103534000  1 -15.866323000 0.378678000 1.001990000  8 -14.042750000 -0.483454000 0.572890000  6 -13.726155000 -1.606828000 -0.223823000  1 -13.550696000 -1.269866000 -1.254794000  7 -12.491138000 -2.297478000 0.237818000  6 -12.371714000 -3.655707000 0.035920000  1 -13.223041000 -4.135842000 -0.437875000  6 -11.383668000 -1.536431000 0.660097000  8 -11.373248000 -0.308831000 0.661398000  7 -10.310448000 -2.294996000 1.090691000  1 -9.473154000 -1.729222000 1.281432000  6 -10.119482000 -3.672034000 0.947223000  8 -9.051963000 -4.207997000 1.287800000  6 -11.256216000 -4.357832000 0.369786000  1 -11.193105000 -5.424552000 0.183704000  6 -14.942208000 -2.561231000 -0.150280000  1 -14.816076000 -3.175518000 0.759122000  8 -15.054073000 -3.362017000 -1.303428000  1 -16.031304000 -3.438880000 -1.512509000  6 -16.090911000 -1.594933000 0.172760000  1 -16.940471000 -2.102357000 0.645089000  8 -16.535245000 -0.844046000 -0.980211000  15 -17.462056000 -1.573600000 -2.161418000  8 -17.632102000 -3.047643000 -1.792950000  8 -18.647214000 -0.702895000 -2.484125000  8 -16.328677000 -1.471690000 -3.387296000  6 -16.733234000 -1.236616000 -4.741801000  1 -17.301931000 -0.298973000 -4.811099000  1 -17.372064000 -2.057438000 -5.105743000  6 -15.498896000 -1.149118000 -5.640745000  1 -15.853949000 -1.043675000 -6.675339000  8 -14.705325000 0.035798000 -5.331684000  6 -13.456453000 -0.340710000 -4.751093000  1 -12.621699000 -0.155404000 -5.436499000  7 -13.213133000 0.530911000 -3.585096000  6 -14.229158000 0.722735000 -2.671201000  1 -15.106816000 0.103983000 -2.827782000  6 -12.026103000 1.280953000 -3.523174000  8 -11.133011000 1.227796000 -4.364810000  7 -11.941010000 2.100909000 -2.403856000  1 -11.057372000 2.603829000 -2.304887000  6 -12.939478000 2.380619000 -1.455673000  8 -12.745239000 3.224406000 -0.560217000  6 -14.145136000 1.612440000 -1.647256000  1 -14.961656000 1.722011000 -0.945249000  6 -13.573152000 -1.855699000 -4.425988000  1 -14.099020000 -1.983851000 -3.473356000  8 -12.356945000 -2.553093000 -4.318543000  1 -11.930168000 -2.613446000 -5.206725000  6 -14.515664000 -2.322606000 -5.552158000  1 -14.990397000 -3.284854000 -5.329044000  8 -13.825138000 -2.377493000 -6.815202000  15 -12.966964000 -3.736927000 -7.301446000  8 -13.326285000 -4.922368000 -6.444702000  8 -13.015397000 -3.736402000 -8.807998000  8 -11.405218000 -3.254067000 -6.813195000  6 -10.752968000 -2.290668000 -7.663197000  1 -11.401957000 -1.413585000 -7.805985000  1 -10.558248000 -2.736740000 -8.648069000  6 -9.436276000 -1.834001000 -7.066433000  1 -8.966702000 -1.150116000 -7.793573000  8 -9.700551000 -1.101869000 -5.841056000  6 -8.525413000 -1.214412000 -5.059129000  1 -7.733840000 -0.523222000 -5.390820000  7 -8.816631000 -0.924497000 -3.672485000  6 -7.928484000 -0.412360000 -2.746368000  7 -6.761638000 0.229341000 -3.007913000  6 -6.117533000 0.629144000 -1.909665000  7 -4.929258000 1.284519000 -1.998522000  1 -4.658769000 1.550977000 -2.937968000  1 -4.731712000 1.964022000 -1.264331000  7 -6.572530000 0.341971000 -0.641100000  1 -5.973445000 0.557873000 0.152659000  6 -7.766446000 -0.363243000 -0.314022000  8 -8.005602000 -0.622198000 0.874272000  6 -8.505267000 -0.686185000 -1.493321000  7 -9.707626000 -1.362331000 -1.647942000  6 -9.864709000 -1.496569000 -2.947928000  1 -10.723255000 -1.935879000 -3.443086000  6 -8.096144000 -2.685082000 -5.238471000  1 -8.821419000 -3.288585000 -4.669367000  8 -6.773785000 -2.941273000 -4.844145000  1 -6.713898000 -2.958218000 -3.867114000  6 -8.371091000 -2.926268000 -6.739091000  1 -8.746555000 -3.935298000 -6.924303000  8 -7.220390000 -2.681995000 -7.544858000  15 -6.470741000 -3.903440000 -8.411546000  8 -7.332591000 -5.153978000 -8.344038000  8 -6.077900000 -3.293496000 -9.737902000  8 -5.134565000 -4.188236000 -7.476070000  6 -3.806564000 -3.799368000 -7.855624000  1 -3.708967000 -3.775540000 -8.949244000  1 -3.144688000 -4.570561000 -7.441636000  6 -3.347171000 -2.463942000 -7.277730000  1 -2.245601000 -2.473411000 -7.258804000  8 -3.772167000 -1.371761000 -8.144469000  6 -4.128736000 -0.263940000 -7.333459000  1 -3.276916000 0.386734000 -7.095425000  7 -5.091100000 0.567132000 -8.070593000  6 -6.069587000 -0.033427000 -8.808346000  1 -6.000118000 -1.112995000 -8.917020000  6 -5.042015000 1.994629000 -7.910149000  8 -4.102851000 2.504855000 -7.280795000  7 -6.056399000 2.728451000 -8.466732000  6 -7.023296000 2.123638000 -9.165468000  7 -7.983372000 2.920775000 -9.714040000  1 -8.846952000 2.507575000 -10.037478000  1 -8.026122000 3.879305000 -9.392363000  6 -7.059253000 0.709404000 -9.390627000  1 -7.829388000 0.236616000 -9.993082000  6 -4.724513000 -0.888015000 -6.062357000  1 -5.718874000 -1.268261000 -6.324900000  8 -4.775858000 0.052612000 -5.010263000  1 -5.587208000 -0.081583000 -4.470104000  6 -3.837978000 -2.136841000 -5.845487000  1 -4.437200000 -2.949471000 -5.431703000  8 -2.730529000 -1.831116000 -4.985141000  15 -2.159815000 -3.090892000 -4.046740000  8 -2.130809000 -4.385775000 -4.830299000  8 -0.947803000 -2.533127000 -3.338486000  8 -3.436362000 -3.292552000 -2.974558000  6 -3.921907000 -2.144137000 -2.267672000  1 -4.326902000 -1.416581000 -2.982258000  1 -3.112010000 -1.653615000 -1.704114000  6 -5.008603000 -2.548119000 -1.280955000  1 -5.375834000 -1.643904000 -0.793259000  8 -6.138027000 -3.183742000 -1.970014000  6 -6.226912000 -4.570070000 -1.571874000  1 -7.055965000 -4.696489000 -0.864572000  7 -6.554979000 -5.359829000 -2.738714000  6 -7.861398000 -5.535983000 -3.180592000  7 -8.992629000 -5.228513000 -2.522785000  6 -10.079683000 -5.498483000 -3.272190000  1 -11.043166000 -5.292838000 -2.800844000  7 -10.131968000 -5.947138000 -4.540552000  6 -8.966243000 -6.197911000 -5.197177000  7 -9.022881000 -6.552190000 -6.513178000  1 -8.254221000 -6.213398000 -7.113426000  1 -9.943127000 -6.407747000 -6.918431000  6 -7.749091000 -6.063370000 -4.481097000  7 -6.408938000 -6.224738000 -4.829759000  6 -5.732875000 -5.771393000 -3.788452000  1 -4.654149000 -5.682703000 -3.730708000  6 -4.887184000 -4.887051000 -0.897182000  1 -4.111315000 -4.992827000 -1.667969000  8 -4.969753000 -6.036599000 -0.076232000  1 -4.077265000 -6.133433000 0.304080000  6 -4.618839000 -3.550125000 -0.192677000  1 -3.578959000 -3.456995000 0.119595000  8 -5.523188000 -3.439360000 0.918801000  15 -5.081378000 -2.547342000 2.260377000  8 -4.344256000 -1.292578000 1.833955000  8 -6.270770000 -2.542125000 3.185329000  8 -3.886537000 -3.540097000 2.903467000  6 -4.186163000 -4.929871000 3.084680000  1 -4.960513000 -5.244269000 2.374360000  1 -4.544264000 -5.120815000 4.108783000  6 -2.930140000 -5.763880000 2.815755000  1 -3.204877000 -6.827830000 2.816563000  8 -2.427211000 -5.424516000 1.485464000  6 -1.131661000 -4.826837000 1.609835000  1 -0.354253000 -5.549623000 1.335893000  7 -0.956538000 -3.675878000 0.732812000  6 0.173443000 -3.465165000 -0.060883000  7 1.070063000 -4.390502000 -0.471874000  6 2.149365000 -3.836999000 -1.059150000  7 3.155595000 -4.606623000 -1.508712000  1 3.071445000 -5.614657000 -1.385423000  1 4.124050000 -4.251209000 -1.559324000  7 2.279122000 -2.480136000 -1.276542000  1 3.187554000 -2.117804000 -1.627878000  6 1.309600000 -1.509564000 -0.979600000  8 1.501050000 -0.314031000 -1.288883000  6 0.196094000 -2.083860000 -0.302732000  7 -0.889039000 -1.462747000 0.294639000  6 -1.544148000 -2.417077000 0.927262000  1 -2.444202000 -2.265019000 1.512509000  6 -1.016776000 -4.410094000 3.088004000  1 -1.638048000 -3.512550000 3.224781000  8 0.316121000 -4.162730000 3.449650000  1 0.450950000 -4.588921000 4.345844000  6 -1.760752000 -5.543902000 3.793285000  1 -2.109167000 -5.260580000 4.793967000  8 -0.987896000 -6.762161000 3.862080000  15 0.257650000 -6.918439000 4.970646000  8 0.485573000 -5.563996000 5.654000000  8 0.027330000 -8.168599000 5.775080000  8 1.561293000 -7.090179000 3.963692000  6 1.567235000 -8.220116000 3.074570000  1 0.900253000 -8.012747000 2.232617000  1 1.201891000 -9.117651000 3.596625000  6 2.977195000 -8.506938000 2.581697000  1 2.943774000 -9.449399000 2.024701000  8 3.448269000 -7.479922000 1.659872000  6 4.567687000 -6.794213000 2.210180000  1 5.502898000 -7.066168000 1.701864000  7 4.358844000 -5.355527000 2.019532000  6 5.340864000 -4.371543000 2.033729000  7 6.669969000 -4.547742000 2.171897000  6 7.346366000 -3.397889000 2.281378000  7 8.666093000 -3.416410000 2.564211000  1 9.163795000 -4.260138000 2.277066000  1 9.234270000 -2.570947000 2.371902000  7 6.743843000 -2.153766000 2.184533000  1 7.313369000 -1.296093000 2.290296000  6 5.366169000 -1.936422000 2.012008000  8 4.928676000 -0.765379000 1.943933000  6 4.637796000 -3.159135000 1.949200000  7 3.268918000 -3.390261000 1.912842000  6 3.131981000 -4.705150000 1.974940000  1 2.196039000 -5.240103000 2.004153000  6 4.634375000 -7.189833000 3.700439000  1 3.934440000 -6.541464000 4.249136000  8 5.941419000 -7.058855000 4.191458000  1 6.146263000 -7.828114000 4.761308000  1 3.560350000 -8.852529000 4.667434000  8 5.001267000 -9.602182000 3.363654000  6 4.017413000 -8.599474000 3.704306000  15 5.611891000 -10.592481000 4.586408000  8 6.496906000 -11.575709000 3.865984000  8 4.494186000 -10.964698000 5.526922000  8 6.558875000 -9.469548000 5.421354000  6 7.991699000 -9.517226000 5.277996000  1 8.285085000 -9.157281000 4.281123000  1 8.348240000 -10.549570000 5.392288000  6 8.603559000 -8.629517000 6.351243000  1 9.697703000 -8.767625000 6.345405000  8 8.300507000 -7.249836000 6.050454000  6 7.879176000 -6.547605000 7.223333000  1 8.465587000 -5.626400000 7.310711000  7 6.491034000 -6.113270000 7.089199000  6 6.129522000 -4.917778000 6.476560000  7 6.945567000 -3.926910000 6.075091000  6 6.244742000 -2.927016000 5.501683000  1 6.834668000 -2.083984000 5.135725000  7 4.918298000 -2.828662000 5.303143000  6 4.118519000 -3.852311000 5.716497000  7 2.786944000 -3.745538000 5.521315000  1 2.166848000 -4.545792000 5.661286000  1 2.466978000 -2.997350000 4.914224000  6 4.729853000 -4.965533000 6.353769000  7 4.232821000 -6.168328000 6.855544000  6 5.307218000 -6.827189000 7.259947000  1 5.299781000 -7.818574000 7.695826000  6 8.149730000 -7.477198000 8.417345000  1 7.418036000 -7.333858000 9.228028000  8 9.476512000 -7.219792000 8.864787000  1 9.835415000 -8.099930000 9.088337000  1 7.033012000 -9.230336000 7.735023000  6 8.073166000 -8.879652000 7.782245000  8 8.872004000 -9.762627000 8.566941000  1 9.019890000 -10.577004000 8.062444000 | 1 7.729873000 -9.738385000 -1.335144000  8 7.191591000 -9.659588000 -2.172495000  6 8.119639000 -9.326586000 -3.196511000  1 7.831898000 -9.849552000 -4.122064000  1 9.137561000 -9.653825000 -2.928836000  6 8.185759000 -7.827088000 -3.493156000  1 9.036255000 -7.623952000 -4.165082000  8 6.965548000 -7.405917000 -4.145072000  6 6.550226000 -6.131355000 -3.679760000  1 6.489707000 -5.406678000 -4.498493000  7 5.160288000 -6.267808000 -3.178210000  6 4.779680000 -7.409795000 -2.511726000  1 5.586350000 -8.099634000 -2.263415000  6 4.219509000 -5.306376000 -3.599564000  8 4.550535000 -4.261864000 -4.162339000  7 2.898654000 -5.653458000 -3.367308000  1 2.200428000 -4.972980000 -3.717536000  6 2.429081000 -6.807667000 -2.720356000  8 1.209582000 -7.024034000 -2.625878000  6 3.475021000 -7.683890000 -2.236114000  1 3.192920000 -8.592198000 -1.714394000  6 7.596617000 -5.630888000 -2.655776000  1 7.121018000 -5.144855000 -1.793927000  8 8.441241000 -4.729022000 -3.360936000  1 9.206815000 -4.555139000 -2.766384000  6 8.323673000 -6.940764000 -2.251171000  1 7.801235000 -7.390544000 -1.397756000  8 9.723329000 -6.840378000 -1.970144000  8 9.193771000 -5.686042000 0.310630000  15 10.293489000 -5.937110000 -0.696544000  8 11.655037000 -6.473667000 -0.339213000  8 10.462431000 -4.459183000 -1.510131000  6 11.728582000 -4.015335000 -2.025437000  1 11.596974000 -3.797405000 -3.094189000  1 12.489251000 -4.797276000 -1.907415000  6 12.162081000 -2.741050000 -1.301136000  1 13.048229000 -2.333288000 -1.806283000  8 11.106737000 -1.747714000 -1.390211000  6 10.456997000 -1.574390000 -0.115831000  1 10.658503000 -0.560207000 0.234910000  7 9.018751000 -1.666004000 -0.288026000  6 8.224157000 -0.585675000 -0.662452000  7 8.633628000 0.672999000 -0.879216000  6 7.642329000 1.494339000 -1.255173000  7 7.923963000 2.768656000 -1.565528000  1 8.877734000 3.066381000 -1.417102000  1 7.204114000 3.464431000 -1.831867000  7 6.319683000 1.085316000 -1.357783000  1 5.577000000 1.776751000 -1.566873000  6 5.870981000 -0.242882000 -1.184724000  8 4.653776000 -0.524229000 -1.319257000  6 6.934954000 -1.114316000 -0.822137000  7 6.944601000 -2.483162000 -0.579018000  6 8.191537000 -2.788905000 -0.268886000  1 8.546261000 -3.781840000 -0.011620000  6 11.069547000 -2.640825000 0.829697000  1 10.511382000 -3.571812000 0.692934000  8 11.023797000 -2.352652000 2.203867000  1 11.680001000 -1.605103000 2.398073000  6 12.457217000 -2.910033000 0.197600000  1 12.774932000 -3.934550000 0.423330000  8 13.569866000 -2.077292000 0.586969000  15 13.611405000 -0.547493000 1.238880000  8 12.833885000 -0.537753000 2.557972000  8 15.053344000 -0.120452000 1.170545000  8 12.653807000 0.365311000 0.262496000  6 13.036308000 0.792227000 -1.056335000  1 12.340190000 0.340936000 -1.770576000  1 14.063202000 0.476383000 -1.289826000  6 12.955659000 2.309555000 -1.127110000  1 13.205182000 2.621187000 -2.146127000  8 11.600270000 2.761382000 -0.853173000  6 11.530751000 3.503770000 0.361424000  1 11.212057000 4.527220000 0.138638000  7 10.485588000 2.951371000 1.229577000  6 9.199953000 3.473945000 1.330896000  7 8.783427000 4.660662000 0.861412000  6 7.477780000 4.888829000 1.081001000  7 6.950254000 6.072339000 0.740715000  1 7.555958000 6.728977000 0.265918000  1 5.968779000 6.347335000 0.926995000  7 6.638361000 3.950002000 1.665640000  1 5.618628000 4.133153000 1.724357000  6 7.054017000 2.704333000 2.183484000  8 6.209624000 1.915172000 2.677411000  6 8.458327000 2.512800000 2.036611000  7 9.272889000 1.442724000 2.387885000  6 10.465071000 1.723125000 1.890237000  1 11.341149000 1.091645000 1.970899000  6 12.943762000 3.518875000 1.002897000  1 12.986502000 2.761140000 1.795784000  8 13.270850000 4.755900000 1.586653000  1 13.178222000 5.401489000 0.833503000  6 13.889543000 3.028329000 -0.141136000  1 14.621189000 2.324623000 0.269992000  8 14.702024000 4.044795000 -0.759661000  15 14.153816000 5.405571000 -1.528621000  8 12.941539000 5.961878000 -0.781959000  8 15.358799000 6.274966000 -1.784697000  8 13.644729000 4.714383000 -2.970766000  6 13.613209000 5.564125000 -4.131789000  1 14.352269000 5.190956000 -4.855749000  1 13.890345000 6.594463000 -3.872236000  6 12.238630000 5.595595000 -4.781860000  1 12.247489000 6.374181000 -5.562829000  8 11.940825000 4.322164000 -5.401098000  6 10.557166000 4.029961000 -5.295860000  1 10.101268000 3.942141000 -6.292778000  7 10.433615000 2.713345000 -4.644786000  6 9.292715000 1.921069000 -4.593280000  7 8.034635000 2.243870000 -4.954125000  6 7.200969000 1.198150000 -4.787236000  1 6.156830000 1.379099000 -5.053218000  7 7.469909000 -0.044376000 -4.333062000  6 8.747589000 -0.337983000 -3.973559000  7 9.050798000 -1.593466000 -3.554910000  1 9.867051000 -1.701946000 -2.953014000  1 8.280313000 -2.227194000 -3.370770000  6 9.733148000 0.675329000 -4.101777000  7 11.106325000 0.688126000 -3.881345000  6 11.483871000 1.912161000 -4.220649000  1 12.492431000 2.299852000 -4.201496000  6 9.872989000 5.189045000 -4.537985000  1 9.132817000 4.811255000 -3.816786000  8 9.281181000 6.018669000 -5.524216000  1 8.950624000 6.806778000 -5.037464000  6 11.071692000 5.876599000 -3.831637000  1 11.252270000 5.414796000 -2.854784000  8 10.972165000 7.299213000 -3.706259000  15 9.843138000 7.999864000 -2.705290000  8 10.313433000 9.405427000 -2.432075000  8 9.422651000 7.017848000 -1.634578000  8 8.531905000 8.047800000 -3.785728000  6 8.044212000 9.285655000 -4.330010000  1 7.753508000 9.073317000 -5.367106000  1 8.836033000 10.046180000 -4.327759000  6 6.819468000 9.831261000 -3.588485000  1 6.461417000 10.701065000 -4.158902000  8 5.750284000 8.842883000 -3.562793000  6 5.509850000 8.421790000 -2.213740000  1 4.561901000 8.825507000 -1.836097000  7 5.382680000 6.969805000 -2.173753000  6 4.235382000 6.231996000 -1.898065000  7 2.993838000 6.702602000 -1.669944000  6 2.107792000 5.739202000 -1.382083000  7 0.812814000 6.067327000 -1.158185000  1 0.633447000 7.038944000 -0.941823000  1 0.112020000 5.383915000 -0.837152000  7 2.436478000 4.395764000 -1.338287000  1 1.728182000 3.695847000 -1.035631000  6 3.717608000 3.870734000 -1.600214000  8 3.925510000 2.634346000 -1.520054000  6 4.655239000 4.890476000 -1.920418000  7 6.010099000 4.804050000 -2.223198000  6 6.414705000 6.053233000 -2.365569000  1 7.430887000 6.372006000 -2.556515000  6 6.717270000 8.941196000 -1.402159000  1 7.558393000 8.274815000 -1.625488000  8 6.517565000 8.961658000 -0.012219000  1 6.726091000 9.890536000 0.317085000  6 7.031935000 10.254639000 -2.127848000  1 8.053042000 10.599987000 -1.930132000  8 6.075240000 11.308676000 -1.887844000  15 5.942417000 12.082305000 -0.417995000  8 6.913550000 11.459653000 0.581131000  8 5.919363000 13.566434000 -0.673214000  8 4.429621000 11.545712000 0.043594000  6 3.367408000 11.819027000 -0.887699000  1 3.561648000 11.289657000 -1.831766000  1 3.320446000 12.898374000 -1.100327000  6 2.013371000 11.383635000 -0.359323000  1 1.285077000 11.735567000 -1.099072000  8 1.913567000 9.932300000 -0.262536000  6 1.716125000 9.550257000 1.105649000  1 0.669117000 9.276610000 1.279416000  7 2.503230000 8.361350000 1.377415000  6 2.019597000 7.115293000 1.759868000  7 0.755441000 6.802500000 2.095777000  6 0.589811000 5.499080000 2.381747000  7 -0.607449000 5.042095000 2.763045000  1 -1.365269000 5.713576000 2.887090000  1 -0.765939000 4.052305000 3.028368000  7 1.625644000 4.572664000 2.299977000  1 1.455499000 3.583106000 2.547136000  6 2.957583000 4.880338000 1.968079000  8 3.817350000 3.965987000 1.907895000  6 3.140924000 6.266226000 1.714372000  7 4.273552000 6.970175000 1.320848000  6 3.862857000 8.210154000 1.122856000  1 4.482637000 9.019339000 0.758345000  6 2.103563000 10.789347000 1.950203000  1 3.193967000 10.844054000 2.044230000  8 1.555630000 10.787707000 3.251137000  1 0.609267000 10.994528000 3.141292000  6 1.617850000 11.916360000 1.021204000  1 2.060727000 12.896249000 1.240833000  8 0.195205000 11.936980000 1.216226000  15 -0.884122000 12.872353000 0.323164000  8 -0.151677000 13.522263000 -0.828577000  8 -1.729460000 13.630374000 1.314254000  8 -1.882647000 11.637672000 -0.218901000  6 -1.371555000 10.724547000 -1.198605000  1 -0.279607000 10.674901000 -1.165295000  1 -1.671605000 11.047606000 -2.207928000  6 -1.867293000 9.304555000 -0.961660000  1 -1.432902000 8.674500000 -1.751223000  8 -1.369740000 8.814439000 0.319860000  6 -2.462269000 8.527542000 1.181735000  1 -2.625038000 7.445029000 1.269138000  7 -2.182102000 8.993575000 2.529718000  6 -2.328149000 8.246318000 3.685592000  7 -2.693298000 6.956385000 3.801945000  6 -2.798961000 6.580752000 5.095467000  1 -3.132058000 5.557952000 5.267383000  7 -2.570685000 7.318013000 6.199285000  6 -2.178359000 8.610301000 6.048073000  7 -1.896702000 9.336346000 7.160130000  1 -1.813196000 10.340079000 7.074062000  1 -2.200447000 8.966466000 8.051598000  6 -2.054466000 9.138699000 4.739626000  7 -1.758111000 10.408770000 4.248680000  6 -1.862345000 10.283878000 2.931382000  1 -1.721616000 11.073640000 2.207660000  6 -3.697086000 9.225350000 0.562502000  1 -3.624932000 10.294902000 0.800279000  8 -4.928933000 8.763928000 1.052477000  1 -5.136935000 7.902757000 0.619124000  6 -3.382180000 9.075401000 -0.935565000  1 -3.947455000 9.767349000 -1.569001000  8 -3.584829000 7.716362000 -1.372929000  15 -5.064776000 7.225885000 -1.958302000  8 -4.813499000 6.086490000 -2.912743000  8 -5.928329000 8.413616000 -2.311741000  8 -5.571745000 6.588309000 -0.461782000  6 -6.819515000 5.878724000 -0.478340000  1 -7.025861000 5.523429000 -1.497791000  1 -7.641536000 6.538051000 -0.160758000  6 -6.746193000 4.659351000 0.427584000  1 -7.681081000 4.097522000 0.295448000  8 -5.640807000 3.835134000 -0.024123000  6 -4.761825000 3.548770000 1.050519000  1 -4.944226000 2.548798000 1.469249000  7 -3.393666000 3.531865000 0.557486000  6 -2.552244000 2.431750000 0.529532000  7 -2.842342000 1.175737000 0.923757000  6 -1.802012000 0.324969000 0.826697000  7 -1.974425000 -0.961716000 1.157264000  1 -2.802356000 -1.195038000 1.728280000  1 -1.192935000 -1.635847000 1.237091000  7 -0.563035000 0.717547000 0.359036000  1 0.227320000 0.043955000 0.408815000  6 -0.235602000 2.009875000 -0.092751000  8 0.925645000 2.241795000 -0.502773000  6 -1.341436000 2.907048000 -0.003878000  7 -1.451276000 4.254964000 -0.338303000  6 -2.682532000 4.597879000 0.000998000  1 -3.141700000 5.566393000 -0.155195000  6 -5.036492000 4.612437000 2.139841000  1 -4.475171000 5.526626000 1.911268000  8 -4.647337000 4.206982000 3.430309000  1 -5.308498000 3.568981000 3.781933000  6 -6.542385000 4.890092000 1.936891000  1 -6.824092000 5.895173000 2.270583000  8 -7.346390000 3.898305000 2.597599000  15 -7.767339000 4.015987000 4.217692000  8 -7.270981000 5.334116000 4.779539000  8 -9.191858000 3.536714000 4.322052000  8 -6.726524000 2.835747000 4.824943000  6 -7.095419000 1.475351000 4.488186000  1 -7.215031000 1.385537000 3.400955000  1 -8.043384000 1.212979000 4.968637000  6 -6.049935000 0.489917000 4.955200000  1 -6.444594000 -0.511993000 4.754851000  8 -4.826894000 0.682120000 4.191796000  6 -3.750877000 0.267192000 5.023928000  1 -3.642018000 -0.827118000 5.056604000  7 -2.529451000 0.815029000 4.470110000  6 -1.291941000 0.204606000 4.276164000  7 -0.938249000 -1.077389000 4.519649000  6 0.348855000 -1.330707000 4.234545000  7 0.873535000 -2.560153000 4.479287000  1 0.195193000 -3.301503000 4.599748000  1 1.759468000 -2.848635000 4.040274000  7 1.224065000 -0.377966000 3.747877000  1 2.229826000 -0.615247000 3.619666000  6 0.886861000 0.958034000 3.476943000  8 1.740634000 1.764848000 3.036503000  6 -0.476041000 1.229791000 3.765490000  7 -1.183343000 2.414197000 3.640890000  6 -2.400309000 2.139136000 4.069377000  1 -3.235252000 2.827224000 4.054094000  6 -4.140161000 0.852655000 6.407651000  1 -3.992231000 1.939214000 6.370810000  8 -3.413138000 0.341386000 7.503616000  1 -3.922098000 -0.447154000 7.776723000  6 -5.651189000 0.540204000 6.445655000  1 -6.219239000 1.281262000 7.010937000  8 -5.755455000 -0.752932000 7.078712000  15 -7.270779000 -1.312785000 7.511407000  8 -7.080656000 -2.718999000 8.024177000  8 -8.257408000 -0.981683000 6.412909000  8 -7.501581000 -0.257385000 8.799326000  6 -8.579980000 -0.556684000 9.701083000  1 -8.169728000 -0.576070000 10.721393000  1 -9.018924000 -1.541904000 9.489739000  6 -9.699803000 0.472379000 9.624634000  1 -10.507217000 0.139311000 10.292238000  8 -9.253934000 1.770732000 10.121444000  6 -9.328182000 2.766805000 9.138292000  1 -9.904711000 3.625610000 9.501339000  7 -7.948209000 3.276222000 8.827638000  6 -6.808652000 2.657650000 9.268873000  1 -6.974763000 1.858204000 9.980077000  6 -7.896535000 4.360530000 7.939418000  8 -8.907862000 4.993550000 7.631659000  7 -6.639480000 4.640791000 7.429681000  1 -6.679792000 5.153675000 6.529346000  6 -5.436989000 3.990313000 7.757504000  8 -4.385065000 4.268245000 7.157936000  6 -5.570903000 3.009499000 8.816026000  1 -4.687871000 2.472551000 9.145181000  6 -9.997029000 2.177465000 7.857183000  1 -9.286475000 2.235474000 7.022649000  8 -11.184428000 2.860052000 7.501507000  1 -11.890316000 2.206634000 7.700817000  6 -10.277185000 0.683438000 8.216725000  1 -9.797856000 0.024240000 7.486457000  8 -11.703278000 0.489481000 8.216733000  15 -12.284833000 -0.964046000 7.572674000  8 -11.538398000 -2.118481000 8.194526000  8 -13.786013000 -0.815765000 7.616742000  8 -11.722719000 -0.859931000 6.004752000  6 -12.130177000 0.259745000 5.206702000  1 -11.583956000 1.164154000 5.513514000  1 -13.207181000 0.449417000 5.336946000  6 -11.865197000 -0.009491000 3.732756000  1 -12.303001000 0.822711000 3.160131000  8 -10.431798000 -0.055219000 3.468418000  6 -10.163206000 -1.248894000 2.712236000  1 -10.298471000 -1.073951000 1.636847000  7 -8.789455000 -1.656469000 2.892068000  6 -7.716548000 -1.112714000 2.202238000  7 -7.769511000 -0.121337000 1.290853000  6 -6.565119000 0.215043000 0.820080000  7 -6.489585000 1.101485000 -0.208059000  1 -7.281421000 1.744621000 -0.289320000  1 -5.602443000 1.411949000 -0.608621000  7 -5.390023000 -0.315303000 1.305484000  1 -4.504381000 0.173655000 1.079644000  6 -5.304452000 -1.383095000 2.227865000  8 -4.192320000 -1.846916000 2.576450000  6 -6.590834000 -1.822520000 2.652287000  7 -6.956169000 -2.794097000 3.585857000  6 -8.263319000 -2.661496000 3.706341000  1 -8.898479000 -3.253111000 4.351216000  6 -11.207768000 -2.249155000 3.235199000  1 -10.999894000 -2.413746000 4.304257000  8 -11.246910000 -3.476736000 2.557083000  1 -12.096244000 -3.523569000 2.011675000  6 -12.444129000 -1.331382000 3.183129000  1 -13.290545000 -1.705398000 3.772753000  8 -12.825830000 -1.059712000 1.822542000  15 -13.860496000 -2.050422000 0.968902000  8 -13.501149000 -3.506388000 1.252228000  8 -15.282710000 -1.569200000 1.113861000  8 -13.262278000 -1.710991000 -0.543484000  6 -13.073267000 -0.330633000 -0.897195000  1 -12.373435000 0.133389000 -0.189888000  1 -14.028657000 0.216828000 -0.852298000  6 -12.488986000 -0.198021000 -2.296154000  1 -12.303627000 0.869245000 -2.460245000  8 -11.202161000 -0.856666000 -2.406336000  6 -11.336682000 -2.111910000 -3.025075000  1 -10.604128000 -2.192190000 -3.836421000  7 -11.066599000 -3.262211000 -2.081066000  6 -11.570818000 -4.492142000 -2.443422000  1 -12.011068000 -4.528974000 -3.437762000  6 -10.329272000 -3.117107000 -0.895026000  8 -9.666629000 -2.127681000 -0.591938000  7 -10.404866000 -4.223218000 -0.055541000  1 -10.134000000 -4.030867000 0.911114000  6 -10.993845000 -5.472962000 -0.297572000  8 -11.016311000 -6.343306000 0.584091000  6 -11.531885000 -5.588812000 -1.638146000  1 -11.945572000 -6.536352000 -1.966772000  6 -12.803783000 -2.209930000 -3.543962000  1 -13.385129000 -2.775461000 -2.795861000  8 -12.861173000 -2.811959000 -4.817004000  1 -13.682925000 -2.442392000 -5.249619000  6 -13.311318000 -0.753282000 -3.455552000  1 -14.389938000 -0.720011000 -3.261584000  8 -12.985835000 0.011264000 -4.630670000  15 -14.056466000 -0.049237000 -5.922245000  8 -14.882517000 -1.323818000 -5.772393000  8 -14.690769000 1.307894000 -6.094609000  8 -13.005167000 -0.366862000 -7.158577000  6 -12.354803000 0.662560000 -7.920135000  1 -12.604568000 1.659238000 -7.533935000  1 -12.726158000 0.590543000 -8.953547000  6 -10.836916000 0.466512000 -7.942440000  1 -10.437941000 0.886378000 -8.875308000  8 -10.194096000 1.201240000 -6.842152000  6 -9.482465000 0.289131000 -6.026052000  1 -8.421796000 0.228282000 -6.308878000  7 -9.492408000 0.796442000 -4.634588000  6 -10.374208000 1.757874000 -4.217907000  1 -11.138476000 2.012196000 -4.942190000  6 -8.433565000 0.374421000 -3.815042000  8 -7.581205000 -0.429101000 -4.183047000  7 -8.411162000 0.954183000 -2.564264000  1 -7.624397000 0.667886000 -1.977320000  6 -9.240182000 1.967853000 -2.071196000  8 -9.038214000 2.447187000 -0.938363000  6 -10.277420000 2.363025000 -2.996618000  1 -10.980110000 3.134633000 -2.701734000  6 -10.171035000 -1.072599000 -6.252485000  1 -11.146043000 -1.057629000 -5.756852000  8 -9.468949000 -2.196433000 -5.798316000  1 -8.540821000 -2.122198000 -6.109656000  6 -10.394820000 -0.996720000 -7.777341000  1 -11.145073000 -1.715505000 -8.118636000  8 -9.145610000 -1.194172000 -8.442338000  15 -8.897247000 -2.541948000 -9.423945000  8 -9.813042000 -3.673817000 -9.022549000  8 -8.809622000 -2.046457000 -10.852340000  8 -7.364841000 -2.948500000 -8.880499000  6 -6.427189000 -1.873284000 -8.755243000  1 -6.903654000 -0.912876000 -8.978283000  1 -5.603016000 -2.014044000 -9.472679000  6 -5.818467000 -1.819037000 -7.345698000  1 -5.578989000 -0.770625000 -7.111274000  8 -6.759058000 -2.303153000 -6.355540000  6 -5.970337000 -2.633287000 -5.220545000  1 -5.635329000 -1.735066000 -4.686468000  7 -6.751172000 -3.419965000 -4.291559000  6 -6.453276000 -3.526979000 -2.947191000  7 -5.531668000 -2.794347000 -2.273245000  6 -5.327568000 -3.235961000 -1.026821000  7 -4.377478000 -2.659975000 -0.265061000  1 -3.775160000 -1.952822000 -0.667155000  1 -4.135639000 -3.040247000 0.641146000  7 -6.023611000 -4.286434000 -0.469186000  1 -5.580755000 -4.738376000 0.351322000  6 -7.013797000 -5.073438000 -1.137127000  8 -7.522360000 -6.043453000 -0.562780000  6 -7.235122000 -4.593827000 -2.475831000  7 -7.993325000 -5.139904000 -3.511022000  6 -7.668179000 -4.432794000 -4.579123000  1 -8.069629000 -4.550624000 -5.577435000  6 -4.783105000 -3.413809000 -5.821407000  1 -5.170629000 -4.403793000 -6.107377000  8 -3.658626000 -3.498257000 -4.991617000  1 -3.776370000 -4.182051000 -4.296971000  6 -4.503360000 -2.612784000 -7.114455000  1 -4.265381000 -3.269273000 -7.953968000  8 -3.438427000 -1.690358000 -6.907324000  15 -2.301502000 -1.394665000 -8.094113000  8 -2.359087000 -2.511408000 -9.122883000  8 -2.412093000 0.058561000 -8.495885000  8 -0.923969000 -1.658652000 -7.208189000  6 -0.208842000 -0.598429000 -6.556754000  1 -0.226891000 0.311462000 -7.171993000  1 0.824617000 -0.959657000 -6.477936000  6 -0.686912000 -0.257345000 -5.142320000  1 0.184160000 0.109223000 -4.573224000  8 -1.666761000 0.817257000 -5.186952000  6 -2.535115000 0.661114000 -4.074714000  1 -2.144731000 1.117624000 -3.153715000  7 -3.804507000 1.342348000 -4.385205000  6 -4.258090000 1.361774000 -5.674060000  1 -3.603070000 0.913896000 -6.418551000  6 -4.604035000 1.848119000 -3.307166000  8 -4.192230000 1.718241000 -2.139435000  7 -5.784883000 2.454604000 -3.623482000  6 -6.203112000 2.500576000 -4.893093000  7 -7.374875000 3.145614000 -5.136130000  1 -7.834630000 3.061956000 -6.031455000  1 -7.933766000 3.444178000 -4.347760000  6 -5.463087000 1.931778000 -5.979539000  1 -5.822175000 1.963540000 -7.003796000  6 -2.701623000 -0.858886000 -3.937782000  1 -3.363464000 -1.169172000 -4.748936000  8 -3.225612000 -1.237384000 -2.684556000  1 -4.055007000 -1.756198000 -2.823934000  6 -1.307074000 -1.420796000 -4.329972000  1 -1.437493000 -2.313069000 -4.942976000  8 -0.473218000 -1.702709000 -3.197993000  15 0.365440000 -3.141735000 -3.122803000  8 0.925880000 -3.594949000 -4.465329000  8 1.283849000 -2.976143000 -1.943103000  8 -0.844156000 -4.236111000 -2.823568000  6 -1.827349000 -3.946953000 -1.820402000  1 -2.360741000 -3.036888000 -2.102453000  1 -1.354909000 -3.783204000 -0.840335000  6 -2.810993000 -5.094754000 -1.692377000  1 -3.588553000 -4.787395000 -1.000842000  8 -3.462121000 -5.419985000 -2.970032000  6 -2.925221000 -6.655747000 -3.485693000  1 -3.712439000 -7.419510000 -3.478359000  7 -2.562177000 -6.463664000 -4.882815000  6 -3.483670000 -6.583655000 -5.919537000  7 -4.709772000 -7.135784000 -5.868187000  6 -5.333800000 -7.047892000 -7.060663000  1 -6.329861000 -7.494012000 -7.109049000  7 -4.908809000 -6.455886000 -8.193843000  6 -3.678279000 -5.872931000 -8.200772000  7 -3.274719000 -5.217830000 -9.329300000  1 -2.703784000 -4.370138000 -9.195572000  1 -4.028368000 -5.083134000 -9.996632000  6 -2.879435000 -5.969912000 -7.034224000  7 -1.616034000 -5.484601000 -6.700990000  6 -1.470156000 -5.774646000 -5.417944000  1 -0.621810000 -5.483666000 -4.810309000  6 -1.770329000 -7.023080000 -2.540646000  1 -0.895997000 -6.428362000 -2.816042000  8 -1.451998000 -8.402964000 -2.554435000  1 -0.480943000 -8.416934000 -2.477736000  6 -2.258885000 -6.428014000 -1.215390000  1 -1.451938000 -6.337197000 -0.481026000  8 -3.341537000 -7.229737000 -0.688859000  15 -3.848401000 -6.978221000 0.881409000  8 -4.057807000 -5.495777000 1.181920000  8 -4.930936000 -7.997843000 1.121141000  8 -2.460021000 -7.374399000 1.721507000  6 -1.904402000 -8.666034000 1.421216000  1 -2.098612000 -8.917387000 0.370567000  1 -2.359407000 -9.440716000 2.058153000  6 -0.393382000 -8.663039000 1.618803000  1 -0.013328000 -9.613360000 1.218595000  8 0.204960000 -7.581192000 0.851495000  6 0.794890000 -6.641190000 1.753106000  1 1.883992000 -6.749048000 1.735985000  7 0.550902000 -5.264226000 1.361740000  6 1.574889000 -4.459570000 0.879495000  7 2.742558000 -4.904591000 0.403522000  6 3.555741000 -3.918659000 0.010920000  7 4.738500000 -4.240798000 -0.554193000  1 5.012228000 -5.196887000 -0.335851000  1 5.512013000 -3.546239000 -0.577699000  7 3.232720000 -2.580396000 0.138644000  1 3.845369000 -1.883986000 -0.318220000  6 1.982530000 -2.092735000 0.560259000  8 1.767738000 -0.854835000 0.559134000  6 1.114169000 -3.144128000 0.981785000  7 -0.190686000 -3.139099000 1.460051000  6 -0.502592000 -4.406271000 1.689720000  1 -1.466800000 -4.774084000 2.019519000  6 0.248029000 -6.976178000 3.152436000  1 -0.801570000 -6.647470000 3.179520000  8 0.935851000 -6.341664000 4.199327000  1 1.564991000 -6.999467000 4.613291000  6 0.147832000 -8.510252000 3.055333000  1 -0.522726000 -8.931092000 3.815569000  8 1.377532000 -9.273080000 3.051894000  15 2.772157000 -9.056712000 3.909892000  8 2.492358000 -8.227810000 5.167277000  8 3.438737000 -10.402215000 3.972998000  8 3.616003000 -8.015394000 2.926085000  6 4.007366000 -8.497293000 1.623782000  1 3.283443000 -8.145980000 0.876721000  1 4.036782000 -9.594822000 1.613836000  6 5.394820000 -7.983640000 1.286692000  1 5.697164000 -8.424586000 0.328011000  8 5.388769000 -6.539860000 1.119074000  6 6.147468000 -5.898125000 2.139063000  1 7.043880000 -5.429866000 1.715048000  7 5.366949000 -4.809527000 2.735441000  6 5.832863000 -3.512198000 2.886018000  7 7.101530000 -3.099704000 2.719009000  6 7.250287000 -1.775362000 2.881985000  7 8.473595000 -1.230643000 2.872857000  1 9.270212000 -1.824083000 2.624373000  1 8.637960000 -0.210703000 2.795331000  7 6.173911000 -0.928269000 3.110428000  1 6.320364000 0.095601000 3.070906000  6 4.852872000 -1.346859000 3.360674000  8 3.967987000 -0.480127000 3.579520000  6 4.714437000 -2.762287000 3.295760000  7 3.600442000 -3.590672000 3.449217000  6 4.022981000 -4.801231000 3.114488000  1 3.434234000 -5.707532000 3.121464000  6 6.549197000 -6.982510000 3.157466000  1 5.791842000 -7.014193000 3.948405000  8 7.809246000 -6.716674000 3.725333000  1 8.440558000 -7.364677000 3.358872000  1 6.161672000 -9.137749000 2.985049000  8 7.733874000 -8.516597000 1.754437000  6 6.452973000 -8.290158000 2.358159000  15 8.098906000 -10.076307000 1.270375000  8 8.824030000 -9.918591000 -0.060998000  8 6.903166000 -10.974642000 1.439286000  8 9.194490000 -10.510764000 2.438834000  6 10.471899000 -9.855310000 2.478984000  1 10.476072000 -8.937347000 1.875922000  1 11.236363000 -10.528810000 2.061158000  6 10.846729000 -9.515746000 3.913234000  1 11.907749000 -9.212094000 3.934885000  8 10.015169000 -8.434548000 4.381945000  6 9.872574000 -8.507453000 5.815999000  1 10.371369000 -7.651898000 6.286391000  7 8.467229000 -8.388983000 6.161911000  6 7.849773000 -7.190049000 6.508863000  7 8.442706000 -6.026143000 6.822688000  6 7.529191000 -5.047918000 6.981591000  1 7.928811000 -4.064144000 7.240948000  7 6.191115000 -5.106164000 6.845452000  6 5.620089000 -6.306267000 6.540680000  7 4.278758000 -6.351374000 6.366289000  1 3.815155000 -7.154297000 5.922252000  1 3.813206000 -5.456013000 6.279884000  6 6.468578000 -7.437481000 6.409490000  7 6.233759000 -8.750000000 5.999645000  6 7.437673000 -9.270798000 5.841717000  1 7.642374000 -10.280973000 5.506487000  6 10.545997000 -9.822501000 6.263829000  1 9.959820000 -10.332845000 7.044020000  8 11.854698000 -9.501971000 6.726199000  1 12.410003000 -10.251456000 6.439352000  1 9.678573000 -11.139261000 4.749252000  6 10.631401000 -10.630666000 4.955287000  8 11.702549000 -11.559728000 5.076925000  1 11.878918000 -11.945562000 4.205362000  19 3.606377000 1.312448000 1.071666000 | 1 7.617352000 -9.749874000 -1.293517000  8 7.063003000 -9.649036000 -2.117566000  6 7.981094000 -9.338819000 -3.158611000  1 7.682550000 -9.877155000 -4.072051000  1 8.999949000 -9.664859000 -2.894348000  6 8.048786000 -7.845197000 -3.483268000  1 8.898642000 -7.655820000 -4.159978000  8 6.828831000 -7.435134000 -4.141519000  6 6.417118000 -6.149913000 -3.704046000  1 6.373171000 -5.438363000 -4.535611000  7 5.016518000 -6.272536000 -3.225657000  6 4.609548000 -7.416964000 -2.578436000  1 5.406971000 -8.100513000 -2.288387000  6 4.092409000 -5.309723000 -3.679090000  8 4.444751000 -4.257902000 -4.214289000  7 2.763172000 -5.662473000 -3.510137000  1 2.078531000 -4.977632000 -3.875970000  6 2.269022000 -6.832602000 -2.911783000  8 1.048858000 -7.065045000 -2.901500000  6 3.294003000 -7.700729000 -2.370933000  1 2.991144000 -8.611027000 -1.864591000  6 7.456924000 -5.634466000 -2.679234000  1 6.974045000 -5.145874000 -1.823040000  8 8.295138000 -4.731913000 -3.389983000  1 9.053949000 -4.539726000 -2.792735000  6 8.190515000 -6.936524000 -2.258375000  1 7.673414000 -7.372936000 -1.394693000  8 9.591765000 -6.831916000 -1.987590000  8 9.085973000 -5.636630000 0.278539000  15 10.174533000 -5.909012000 -0.734698000  8 11.538394000 -6.443646000 -0.383624000  8 10.335546000 -4.441917000 -1.566406000  6 11.583359000 -4.039665000 -2.156184000  1 11.399730000 -3.834291000 -3.219765000  1 12.329965000 -4.838721000 -2.066574000  6 12.085961000 -2.766383000 -1.477153000  1 12.951800000 -2.387608000 -2.037011000  8 11.050235000 -1.749633000 -1.523431000  6 10.481731000 -1.541241000 -0.215890000  1 10.733157000 -0.529992000 0.109087000  7 9.034951000 -1.589450000 -0.296769000  6 8.250067000 -0.475383000 -0.578583000  7 8.685383000 0.763421000 -0.841461000  6 7.695027000 1.623452000 -1.120680000  7 7.994783000 2.870660000 -1.507508000  1 8.972811000 3.123698000 -1.489823000  1 7.269764000 3.580175000 -1.719365000  7 6.352946000 1.277967000 -1.046696000  1 5.610009000 1.977237000 -1.235058000  6 5.876346000 -0.019432000 -0.795714000  8 4.636422000 -0.240761000 -0.753446000  6 6.930163000 -0.949571000 -0.598963000  7 6.911551000 -2.320896000 -0.375038000  6 8.171619000 -2.680679000 -0.207154000  1 8.510521000 -3.691930000 -0.005202000  6 11.116278000 -2.612662000 0.708354000  1 10.531308000 -3.531610000 0.604799000  8 11.140736000 -2.315337000 2.080777000  1 11.826058000 -1.587546000 2.241661000  6 12.462439000 -2.917432000 0.005943000  1 12.775446000 -3.943131000 0.232241000  8 13.610100000 -2.094569000 0.308373000  15 13.723078000 -0.572895000 0.968904000  8 13.033238000 -0.556741000 2.335507000  8 15.165013000 -0.171749000 0.807972000  8 12.721727000 0.365390000 0.062010000  6 13.019888000 0.792516000 -1.277854000  1 12.273470000 0.351650000 -1.946405000  1 14.025363000 0.466213000 -1.580030000  6 12.951169000 2.311097000 -1.335462000  1 13.145814000 2.628366000 -2.364686000  8 11.618864000 2.776285000 -0.982347000  6 11.624282000 3.497024000 0.248565000  1 11.306491000 4.527710000 0.060179000  7 10.619267000 2.942855000 1.161558000  6 9.343541000 3.475998000 1.320345000  7 8.929327000 4.684849000 0.908775000  6 7.626957000 4.908292000 1.149459000  7 7.106981000 6.113665000 0.880237000  1 7.695935000 6.770562000 0.385167000  1 6.111637000 6.360793000 1.037649000  7 6.786494000 3.947800000 1.696721000  1 5.761276000 4.104384000 1.720169000  6 7.206251000 2.687530000 2.177758000  8 6.368208000 1.888261000 2.656843000  6 8.610439000 2.499826000 2.011833000  7 9.421436000 1.408768000 2.303143000  6 10.601895000 1.691970000 1.780099000  1 11.474898000 1.052902000 1.818419000  6 13.070421000 3.486887000 0.809523000  1 13.150205000 2.715034000 1.585663000  8 13.442367000 4.711023000 1.394309000  1 13.311602000 5.370263000 0.658998000  6 13.945460000 3.009957000 -0.395068000  1 14.695987000 2.296321000 -0.038685000  8 14.725438000 4.033568000 -1.042742000  15 14.140641000 5.406105000 -1.762852000  8 12.984274000 5.966919000 -0.934935000  8 15.334929000 6.267375000 -2.088308000  8 13.532942000 4.733587000 -3.174961000  6 13.472854000 5.579815000 -4.336923000  1 14.174253000 5.190072000 -5.089157000  1 13.780977000 6.604731000 -4.091677000  6 12.073743000 5.637745000 -4.930078000  1 12.064258000 6.419694000 -5.707489000  8 11.726063000 4.373446000 -5.542968000  6 10.344935000 4.101594000 -5.371965000  1 9.843736000 4.014418000 -6.346923000  7 10.232553000 2.790676000 -4.705751000  6 9.078090000 2.028150000 -4.570422000  7 7.804572000 2.387026000 -4.827978000  6 6.956596000 1.368603000 -4.584190000  1 5.898929000 1.582406000 -4.755869000  7 7.226024000 0.118359000 -4.151245000  6 8.521021000 -0.215203000 -3.907492000  7 8.821047000 -1.483548000 -3.524681000  1 9.686360000 -1.626796000 -3.004554000  1 8.048105000 -2.087069000 -3.267460000  6 9.521274000 0.769547000 -4.115321000  7 10.906645000 0.745767000 -3.997031000  6 11.290077000 1.960707000 -4.361091000  1 12.307214000 2.322433000 -4.412458000  6 9.713871000 5.273550000 -4.589809000  1 8.992881000 4.912314000 -3.841804000  8 9.103223000 6.118007000 -5.551943000  1 8.799225000 6.904588000 -5.046201000  6 10.953183000 5.934273000 -3.930923000  1 11.163746000 5.463341000 -2.964545000  8 10.884940000 7.357423000 -3.792231000  15 9.812843000 8.057783000 -2.731261000  8 10.306168000 9.457062000 -2.467511000  8 9.443885000 7.066960000 -1.648925000  8 8.448433000 8.121717000 -3.740510000  6 7.942496000 9.367758000 -4.248928000  1 7.601574000 9.167759000 -5.273125000  1 8.737416000 10.124447000 -4.276942000  6 6.756539000 9.909941000 -3.444673000  1 6.375376000 10.787024000 -3.988432000  8 5.686417000 8.925318000 -3.378617000  6 5.512083000 8.485237000 -2.024758000  1 4.581307000 8.882217000 -1.601411000  7 5.390077000 7.032009000 -1.994974000  6 4.236931000 6.288580000 -1.756066000  7 2.988495000 6.752657000 -1.554930000  6 2.096388000 5.783998000 -1.306139000  7 0.795307000 6.106481000 -1.111890000  1 0.614387000 7.069240000 -0.859500000  1 0.092969000 5.411985000 -0.815885000  7 2.425625000 4.440448000 -1.272833000  1 1.709965000 3.730512000 -1.018127000  6 3.717063000 3.925314000 -1.485711000  8 3.931329000 2.689659000 -1.402019000  6 4.659823000 4.948372000 -1.775968000  7 6.022438000 4.868048000 -2.042628000  6 6.428070000 6.118572000 -2.168229000  1 7.451009000 6.433785000 -2.333683000  6 6.755765000 8.998838000 -1.264129000  1 7.586947000 8.338043000 -1.534171000  8 6.626585000 9.004454000 0.134460000  1 6.845858000 9.931039000 0.464711000  6 7.039963000 10.318457000 -1.991579000  1 8.071644000 10.655859000 -1.839708000  8 6.104853000 11.377423000 -1.697667000  15 6.029163000 12.126286000 -0.211254000  8 7.035761000 11.483841000 0.739055000  8 6.002259000 13.614517000 -0.440991000  8 4.533026000 11.585998000 0.297640000  6 3.442656000 11.885648000 -0.592418000  1 3.620649000 11.403510000 -1.564394000  1 3.374355000 12.972824000 -0.753036000  6 2.109383000 11.403325000 -0.054378000  1 1.361148000 11.774995000 -0.764075000  8 2.038399000 9.948097000 -0.021895000  6 1.853295000 9.501547000 1.328791000  1 0.809047000 9.212213000 1.495885000  7 2.648792000 8.306941000 1.544164000  6 2.161537000 7.038033000 1.834924000  7 0.892872000 6.715139000 2.135751000  6 0.711973000 5.394594000 2.309342000  7 -0.500280000 4.925852000 2.618939000  1 -1.226632000 5.606888000 2.844135000  1 -0.660639000 3.931594000 2.870563000  7 1.739230000 4.467331000 2.163699000  1 1.550144000 3.460797000 2.306684000  6 3.082832000 4.783157000 1.880204000  8 3.932509000 3.867215000 1.758905000  6 3.278486000 6.187766000 1.740758000  7 4.414047000 6.917072000 1.404182000  6 4.008728000 8.169689000 1.288458000  1 4.634066000 8.998288000 0.981901000  6 2.235836000 10.704330000 2.225712000  1 3.326886000 10.766586000 2.310094000  8 1.703859000 10.636871000 3.531133000  1 0.751442000 10.828629000 3.444330000  6 1.728778000 11.867793000 1.354953000  1 2.165368000 12.840791000 1.614161000  8 0.308601000 11.865203000 1.565941000  15 -0.779556000 12.852459000 0.744955000  8 -0.060964000 13.571723000 -0.373602000  8 -1.614675000 13.547022000 1.790712000  8 -1.789105000 11.655987000 0.141590000  6 -1.292574000 10.786613000 -0.883694000  1 -0.200798000 10.723044000 -0.857288000  1 -1.593214000 11.161611000 -1.874665000  6 -1.801652000 9.361921000 -0.710523000  1 -1.382121000 8.766423000 -1.534219000  8 -1.296595000 8.806126000 0.540120000  6 -2.383016000 8.514123000 1.410913000  1 -2.560166000 7.431729000 1.468180000  7 -2.072313000 8.932115000 2.768099000  6 -2.082304000 8.115110000 3.885764000  7 -2.391628000 6.808122000 3.957451000  6 -2.356280000 6.354215000 5.228407000  1 -2.629139000 5.308589000 5.365465000  7 -2.047703000 7.033026000 6.349237000  6 -1.721512000 8.348196000 6.241866000  7 -1.360379000 9.019665000 7.364567000  1 -1.316679000 10.028971000 7.333185000  1 -1.556018000 8.585162000 8.256860000  6 -1.744368000 8.955774000 4.962626000  7 -1.539233000 10.262735000 4.523880000  6 -1.757767000 10.208897000 3.215859000  1 -1.708798000 11.046643000 2.535292000  6 -3.617067000 9.243821000 0.828913000  1 -3.537370000 10.304064000 1.102043000  8 -4.847903000 8.772599000 1.313179000  1 -5.069327000 7.933108000 0.845243000  6 -3.317926000 9.144437000 -0.677422000  1 -3.884461000 9.862188000 -1.280338000  8 -3.533994000 7.802798000 -1.160787000  15 -5.018402000 7.339720000 -1.757616000  8 -4.777314000 6.225243000 -2.743723000  8 -5.873840000 8.543367000 -2.075139000  8 -5.526291000 6.663527000 -0.279021000  6 -6.779797000 5.964726000 -0.307084000  1 -7.000373000 5.646544000 -1.335720000  1 -7.592373000 6.619784000 0.042182000  6 -6.707054000 4.715357000 0.556581000  1 -7.653172000 4.172070000 0.427328000  8 -5.625352000 3.887632000 0.056073000  6 -4.721115000 3.566321000 1.100560000  1 -4.906226000 2.559714000 1.501356000  7 -3.367520000 3.541449000 0.569612000  6 -2.549971000 2.427060000 0.481719000  7 -2.849358000 1.168244000 0.857129000  6 -1.831739000 0.300467000 0.700565000  7 -2.015816000 -0.991883000 1.003726000  1 -2.815305000 -1.223832000 1.617139000  1 -1.239031000 -1.674976000 1.047239000  7 -0.603713000 0.682272000 0.199296000  1 0.179576000 -0.001585000 0.231217000  6 -0.262070000 1.980320000 -0.217139000  8 0.899554000 2.205542000 -0.638516000  6 -1.349048000 2.893090000 -0.083017000  7 -1.445450000 4.250987000 -0.378799000  6 -2.656204000 4.607794000 0.013947000  1 -3.098482000 5.590313000 -0.095945000  6 -4.954993000 4.607584000 2.221006000  1 -4.397057000 5.523756000 1.991617000  8 -4.529575000 4.176198000 3.491760000  1 -5.179913000 3.528730000 3.846770000  6 -6.464725000 4.897124000 2.066369000  1 -6.734240000 5.891543000 2.439594000  8 -7.255525000 3.886425000 2.714692000  15 -7.638581000 3.955681000 4.346069000  8 -7.139704000 5.262743000 4.931652000  8 -9.056210000 3.461433000 4.471734000  8 -6.574254000 2.769545000 4.897403000  6 -6.940831000 1.414864000 4.535975000  1 -7.072604000 1.347503000 3.448541000  1 -7.881289000 1.137756000 5.022373000  6 -5.884270000 0.426663000 4.970377000  1 -6.279140000 -0.573918000 4.762882000  8 -4.676445000 0.633444000 4.188834000  6 -3.585254000 0.203732000 4.993617000  1 -3.481156000 -0.891516000 5.012482000  7 -2.375801000 0.750001000 4.412061000  6 -1.157858000 0.126987000 4.143444000  7 -0.800085000 -1.154358000 4.375738000  6 0.465625000 -1.420536000 4.010616000  7 0.999327000 -2.644143000 4.246171000  1 0.335053000 -3.381249000 4.443416000  1 1.876205000 -2.936623000 3.785649000  7 1.307657000 -0.484586000 3.439682000  1 2.303304000 -0.718007000 3.235515000  6 0.954310000 0.839634000 3.151528000  8 1.763167000 1.617286000 2.584720000  6 -0.368528000 1.139642000 3.568203000  7 -1.074776000 2.326613000 3.478775000  6 -2.262408000 2.068920000 3.990586000  1 -3.089520000 2.766221000 4.024415000  6 -3.946876000 0.772649000 6.391875000  1 -3.800213000 1.859950000 6.367021000  8 -3.201225000 0.246697000 7.468242000  1 -3.705900000 -0.544860000 7.740361000  6 -5.458361000 0.459975000 6.453908000  1 -6.014862000 1.195269000 7.038175000  8 -5.554044000 -0.840707000 7.072506000  15 -7.064718000 -1.407681000 7.514145000  8 -6.872093000 -2.830060000 7.979282000  8 -8.068615000 -1.038485000 6.443297000  8 -7.268077000 -0.392575000 8.838219000  6 -8.315919000 -0.726222000 9.763702000  1 -7.877238000 -0.753714000 10.771912000  1 -8.740418000 -1.716739000 9.547804000  6 -9.455791000 0.283327000 9.734912000  1 -10.241270000 -0.077607000 10.414068000  8 -9.021752000 1.579297000 10.248624000  6 -9.125522000 2.591284000 9.284538000  1 -9.703912000 3.437272000 9.673726000  7 -7.757557000 3.120823000 8.955164000  6 -6.602924000 2.505773000 9.361242000  1 -6.746259000 1.694399000 10.063701000  6 -7.734948000 4.220214000 8.084307000  8 -8.758721000 4.847886000 7.807414000  7 -6.491571000 4.521923000 7.554863000  1 -6.553454000 5.050639000 6.664388000  6 -5.276004000 3.876488000 7.844017000  8 -4.241765000 4.177451000 7.225900000  6 -5.378339000 2.875673000 8.887433000  1 -4.483656000 2.341848000 9.189214000  6 -9.812804000 2.016116000 8.006833000  1 -9.118909000 2.096021000 7.160334000  8 -11.014395000 2.690870000 7.684735000  1 -11.709310000 2.028354000 7.892215000  6 -10.068516000 0.512940000 8.345051000  1 -9.597773000 -0.125314000 7.591156000  8 -11.491637000 0.300050000 8.374073000  15 -12.068435000 -1.141876000 7.699320000  8 -11.283038000 -2.303827000 8.256026000  8 -13.569718000 -1.022151000 7.795250000  8 -11.558114000 -0.976461000 6.119003000  6 -12.000059000 0.165161000 5.372069000  1 -11.453300000 1.063890000 5.693774000  1 -13.074510000 0.339853000 5.539922000  6 -11.776460000 -0.049048000 3.882008000  1 -12.224110000 0.807604000 3.354844000  8 -10.350779000 -0.091401000 3.577669000  6 -10.106736000 -1.262999000 2.779500000  1 -10.262048000 -1.053177000 1.713117000  7 -8.732993000 -1.684589000 2.918449000  6 -7.672420000 -1.126773000 2.220960000  7 -7.741215000 -0.113630000 1.334384000  6 -6.544948000 0.229843000 0.848629000  7 -6.484653000 1.144065000 -0.156654000  1 -7.270903000 1.797809000 -0.201250000  1 -5.603215000 1.455770000 -0.569143000  7 -5.362559000 -0.314303000 1.298120000  1 -4.481524000 0.180209000 1.061972000  6 -5.260857000 -1.401308000 2.196256000  8 -4.144844000 -1.868723000 2.523560000  6 -6.540548000 -1.848653000 2.633374000  7 -6.889721000 -2.840550000 3.551273000  6 -8.193850000 -2.707888000 3.700566000  1 -8.818020000 -3.313257000 4.343626000  6 -11.147332000 -2.272669000 3.290891000  1 -10.919693000 -2.473966000 4.349664000  8 -11.206720000 -3.476859000 2.573743000  1 -12.068261000 -3.502423000 2.046088000  6 -12.377267000 -1.344844000 3.294914000  1 -13.215869000 -1.733993000 3.885972000  8 -12.782757000 -1.017196000 1.953030000  15 -13.834919000 -1.967957000 1.075099000  8 -13.503283000 -3.437189000 1.320968000  8 -15.247989000 -1.464483000 1.232813000  8 -13.232908000 -1.602423000 -0.430174000  6 -13.029702000 -0.216028000 -0.751308000  1 -12.308367000 0.218156000 -0.046404000  1 -13.975540000 0.343830000 -0.671041000  6 -12.473809000 -0.051192000 -2.158579000  1 -12.279278000 1.018197000 -2.296153000  8 -11.197372000 -0.720570000 -2.316223000  6 -11.360829000 -1.956359000 -2.966645000  1 -10.647993000 -2.022164000 -3.796647000  7 -11.084342000 -3.135627000 -2.060973000  6 -11.611117000 -4.349130000 -2.446225000  1 -12.067205000 -4.354503000 -3.434095000  6 -10.327010000 -3.030831000 -0.883329000  8 -9.643957000 -2.059696000 -0.567125000  7 -10.409038000 -4.155117000 -0.068691000  1 -10.126119000 -3.989218000 0.899486000  6 -11.020310000 -5.389650000 -0.332525000  8 -11.047944000 -6.279341000 0.529397000  6 -11.575641000 -5.465931000 -1.668945000  1 -12.006987000 -6.399452000 -2.014537000  6 -12.840466000 -2.023092000 -3.454282000  1 -13.411679000 -2.601190000 -2.707917000  8 -12.934163000 -2.590836000 -4.740685000  1 -13.758204000 -2.197030000 -5.146926000  6 -13.326902000 -0.563322000 -3.315952000  1 -14.400985000 -0.521104000 -3.099748000  8 -13.013610000 0.228970000 -4.476183000  15 -14.106695000 0.217277000 -5.750166000  8 -14.950275000 -1.047780000 -5.617420000  8 -14.722870000 1.587713000 -5.878154000  8 -13.081608000 -0.084892000 -7.012664000  6 -12.439529000 0.953502000 -7.769163000  1 -12.671373000 1.944539000 -7.358033000  1 -12.835274000 0.905006000 -8.794920000  6 -10.924214000 0.745705000 -7.831559000  1 -10.544330000 1.183589000 -8.764050000  8 -10.248412000 1.450624000 -6.731675000  6 -9.535815000 0.513912000 -5.944538000  1 -8.479617000 0.448589000 -6.242229000  7 -9.523365000 0.991269000 -4.542823000  6 -10.393658000 1.948991000 -4.094738000  1 -11.162784000 2.226313000 -4.805216000  6 -8.459008000 0.542370000 -3.744926000  8 -7.615285000 -0.257265000 -4.140708000  7 -8.421271000 1.090931000 -2.480771000  1 -7.634093000 0.781114000 -1.905978000  6 -9.240173000 2.095760000 -1.954029000  8 -9.028081000 2.541494000 -0.809238000  6 -10.280970000 2.523631000 -2.860342000  1 -10.975215000 3.292698000 -2.539889000  6 -10.241826000 -0.835879000 -6.189699000  1 -11.208517000 -0.821630000 -5.677999000  8 -9.544786000 -1.976175000 -5.771045000  1 -8.620750000 -1.903962000 -6.094671000  6 -10.489324000 -0.723646000 -7.708654000  1 -11.248648000 -1.430381000 -8.055386000  8 -9.251248000 -0.910385000 -8.397728000  15 -9.020728000 -2.233361000 -9.415836000  8 -9.924586000 -3.376832000 -9.019950000  8 -8.967035000 -1.705558000 -10.834332000  8 -7.474794000 -2.648393000 -8.918705000  6 -6.540123000 -1.575523000 -8.756848000  1 -7.024312000 -0.607700000 -8.924395000  1 -5.725934000 -1.676875000 -9.492296000  6 -5.912031000 -1.588976000 -7.356446000  1 -5.647835000 -0.555559000 -7.085471000  8 -6.853219000 -2.091266000 -6.376399000  6 -6.063437000 -2.482424000 -5.262388000  1 -5.709062000 -1.613393000 -4.693743000  7 -6.854104000 -3.292751000 -4.362845000  6 -6.559516000 -3.452567000 -3.022794000  7 -5.635050000 -2.750497000 -2.319792000  6 -5.437541000 -3.240517000 -1.091133000  7 -4.493724000 -2.696681000 -0.296075000  1 -3.866251000 -1.998927000 -0.676735000  1 -4.214967000 -3.169404000 0.555141000  7 -6.140717000 -4.308299000 -0.576766000  1 -5.709625000 -4.780778000 0.236346000  6 -7.125808000 -5.070725000 -1.277935000  8 -7.633166000 -6.064895000 -0.745254000  6 -7.343576000 -4.536898000 -2.596255000  7 -8.097865000 -5.042591000 -3.654360000  6 -7.769380000 -4.294405000 -4.692774000  1 -8.167585000 -4.372839000 -5.696326000  6 -4.895127000 -3.260609000 -5.902964000  1 -5.301627000 -4.233186000 -6.220164000  8 -3.766197000 -3.394531000 -5.085369000  1 -3.891976000 -4.104056000 -4.418511000  6 -4.612058000 -2.418962000 -7.169933000  1 -4.398569000 -3.049914000 -8.035519000  8 -3.524553000 -1.528790000 -6.941668000  15 -2.389533000 -1.217341000 -8.126341000  8 -2.466926000 -2.305631000 -9.183952000  8 -2.481019000 0.247272000 -8.489980000  8 -1.013838000 -1.524939000 -7.252573000  6 -0.281616000 -0.495935000 -6.570956000  1 -0.282961000 0.431149000 -7.160356000  1 0.745233000 -0.876977000 -6.501110000  6 -0.756293000 -0.186131000 -5.148187000  1 0.119425000 0.151402000 -4.568223000  8 -1.719325000 0.903375000 -5.165695000  6 -2.588411000 0.735113000 -4.056089000  1 -2.189692000 1.163799000 -3.125444000  7 -3.845637000 1.445942000 -4.349869000  6 -4.299215000 1.504584000 -5.637549000  1 -3.653418000 1.061425000 -6.393055000  6 -4.631550000 1.949070000 -3.260678000  8 -4.220270000 1.787066000 -2.096954000  7 -5.798898000 2.588048000 -3.561840000  6 -6.221401000 2.663270000 -4.828607000  7 -7.385437000 3.327859000 -5.054026000  1 -7.847840000 3.272836000 -5.950070000  1 -7.935917000 3.620789000 -4.257727000  6 -5.493288000 2.104700000 -5.928446000  1 -5.853395000 2.165303000 -6.951054000  6 -2.778611000 -0.785299000 -3.952004000  1 -3.449617000 -1.067091000 -4.766123000  8 -3.303135000 -1.180771000 -2.703143000  1 -4.137789000 -1.687947000 -2.847570000  6 -1.394818000 -1.361616000 -4.368361000  1 -1.548099000 -2.230177000 -5.009566000  8 -0.555668000 -1.699149000 -3.254403000  15 0.253095000 -3.158962000 -3.247735000  8 0.799109000 -3.564851000 -4.610795000  8 1.181360000 -3.073044000 -2.066655000  8 -0.985628000 -4.232415000 -2.990854000  6 -1.962239000 -3.952469000 -1.978539000  1 -2.492522000 -3.035264000 -2.242266000  1 -1.483893000 -3.805286000 -0.999062000  6 -2.947749000 -5.098135000 -1.860985000  1 -3.718218000 -4.802560000 -1.156822000  8 -3.612366000 -5.398658000 -3.136875000  6 -3.082682000 -6.624853000 -3.681547000  1 -3.873931000 -7.384456000 -3.688918000  7 -2.723408000 -6.400954000 -5.075375000  6 -3.657917000 -6.462148000 -6.105772000  7 -4.896120000 -6.987020000 -6.059853000  6 -5.530076000 -6.843172000 -7.241622000  1 -6.536893000 -7.263963000 -7.294465000  7 -5.102683000 -6.222255000 -8.358214000  6 -3.859012000 -5.667534000 -8.358461000  7 -3.452040000 -4.983675000 -9.468238000  1 -2.860211000 -4.153996000 -9.312861000  1 -4.208838000 -4.810169000 -10.122934000  6 -3.050932000 -5.823192000 -7.204513000  7 -1.772850000 -5.379833000 -6.868129000  6 -1.620859000 -5.718664000 -5.597978000  1 -0.758799000 -5.471431000 -4.990738000  6 -1.924595000 -7.018798000 -2.750596000  1 -1.046721000 -6.428802000 -3.024520000  8 -1.620176000 -8.401195000 -2.787729000  1 -0.648194000 -8.425903000 -2.725320000  6 -2.391710000 -6.438709000 -1.410893000  1 -1.570538000 -6.357746000 -0.691190000  8 -3.465945000 -7.242704000 -0.870712000  15 -3.923292000 -7.008429000 0.717886000  8 -4.130079000 -5.529351000 1.037409000  8 -4.994940000 -8.032851000 0.984129000  8 -2.507287000 -7.406349000 1.509463000  6 -1.956316000 -8.691471000 1.173176000  1 -2.181643000 -8.927022000 0.125090000  1 -2.390068000 -9.477484000 1.810948000  6 -0.439770000 -8.688389000 1.323987000  1 -0.071438000 -9.623584000 0.879121000  8 0.132288000 -7.578484000 0.578094000  6 0.767856000 -6.682608000 1.493467000  1 1.854815000 -6.807729000 1.448740000  7 0.537790000 -5.292806000 1.149403000  6 1.571352000 -4.470001000 0.724453000  7 2.764249000 -4.892434000 0.293257000  6 3.585061000 -3.889372000 -0.034936000  7 4.814576000 -4.184132000 -0.508596000  1 5.101533000 -5.129418000 -0.259734000  1 5.560705000 -3.459605000 -0.465110000  7 3.224295000 -2.560013000 0.067010000  1 3.883655000 -1.821300000 -0.237638000  6 1.951131000 -2.098479000 0.427950000  8 1.709890000 -0.863271000 0.398029000  6 1.086574000 -3.161691000 0.820432000  7 -0.236001000 -3.180462000 1.243950000  6 -0.538964000 -4.454559000 1.447398000  1 -1.507707000 -4.840532000 1.742406000  6 0.254884000 -7.056887000 2.894928000  1 -0.791346000 -6.720872000 2.963908000  8 0.987662000 -6.463720000 3.937251000  1 1.562274000 -7.162807000 4.359602000  6 0.151744000 -8.585947000 2.744409000  1 -0.481979000 -9.044614000 3.514351000  8 1.392430000 -9.323175000 2.648740000  15 2.749892000 -9.194850000 3.582017000  8 2.409347000 -8.480753000 4.894008000  8 3.412806000 -10.543592000 3.552668000  8 3.631947000 -8.071932000 2.728739000  6 4.097798000 -8.457268000 1.417489000  1 3.440328000 -8.016664000 0.656617000  1 4.093688000 -9.550496000 1.316375000  6 5.521558000 -7.973153000 1.216096000  1 5.893593000 -8.392476000 0.271686000  8 5.571514000 -6.526056000 1.095561000  6 6.273785000 -5.938603000 2.190101000  1 7.216850000 -5.499224000 1.844501000  7 5.482633000 -4.834049000 2.734872000  6 5.938455000 -3.526555000 2.839131000  7 7.217228000 -3.122057000 2.753662000  6 7.352712000 -1.788624000 2.838393000  7 8.578530000 -1.243679000 2.887585000  1 9.377838000 -1.840047000 2.658790000  1 8.746525000 -0.230822000 2.748047000  7 6.265971000 -0.931157000 2.919150000  1 6.419918000 0.093526000 2.855269000  6 4.924124000 -1.336122000 3.045547000  8 4.017266000 -0.467343000 3.080901000  6 4.791722000 -2.754150000 3.094727000  7 3.666991000 -3.574444000 3.205763000  6 4.110223000 -4.803581000 2.990433000  1 3.513333000 -5.706229000 2.992144000  6 6.554450000 -7.062012000 3.208843000  1 5.746506000 -7.081767000 3.949416000  8 7.783806000 -6.865960000 3.869838000  1 8.420417000 -7.500312000 3.486044000  1 6.087770000 -9.194258000 2.944137000  8 7.788184000 -8.619352000 1.878667000  6 6.467798000 -8.347124000 2.368103000  15 8.092525000 -10.159884000 1.296137000  8 8.761306000 -9.955384000 -0.058875000  8 6.884836000 -11.040424000 1.472889000  8 9.226284000 -10.672516000 2.391890000  6 10.498009000 -10.007596000 2.455352000  1 10.493357000 -9.066873000 1.888756000  1 11.266018000 -10.659524000 2.010632000  6 10.870616000 -9.726977000 3.903348000  1 11.927585000 -9.411588000 3.940596000  8 10.027807000 -8.676511000 4.421953000  6 9.819883000 -8.855356000 5.835571000  1 10.254333000 -8.015678000 6.391132000  7 8.393796000 -8.816811000 6.115217000  6 7.733428000 -7.710783000 6.642566000  7 8.285052000 -6.617841000 7.197211000  6 7.339670000 -5.709057000 7.511629000  1 7.703751000 -4.787919000 7.974525000  7 6.009367000 -5.767386000 7.318140000  6 5.481417000 -6.892696000 6.755354000  7 4.149215000 -6.926030000 6.526583000  1 3.710301000 -7.626989000 5.915672000  1 3.657570000 -6.047580000 6.638485000  6 6.364299000 -7.958711000 6.436334000  7 6.179661000 -9.180138000 5.789354000  6 7.402089000 -9.645260000 5.596828000  1 7.647434000 -10.567063000 5.081675000  6 10.528013000 -10.167818000 6.229684000  1 9.940895000 -10.737280000 6.967046000  8 11.814400000 -9.827904000 6.738692000  1 12.403152000 -10.538011000 6.419734000  1 9.741858000 -11.432385000 4.631688000  6 10.670343000 -10.900425000 4.883767000  8 11.771940000 -11.797259000 4.979568000  1 11.987838000 -12.118317000 4.090767000  11 3.132623000 1.183191000 0.633060000 |
| **H1** | 1 -16.950427000 -1.667221000 4.349053000  8 -17.352911000 -1.105636000 3.635314000  6 -17.224246000 -1.778693000 2.390617000  1 -17.180225000 -1.008320000 1.604261000  1 -18.101207000 -2.419894000 2.169325000  6 -15.962135000 -2.618451000 2.270854000  1 -15.170933000 -2.153997000 2.866032000  8 -16.181795000 -3.967424000 2.794260000  6 -15.325263000 -4.874502000 2.133409000  1 -14.644257000 -5.368688000 2.836256000  7 -16.146867000 -5.958117000 1.510223000  6 -17.489502000 -5.805256000 1.268536000  1 -17.899440000 -4.862609000 1.617022000  6 -15.454136000 -7.118714000 1.140738000  8 -14.245329000 -7.261128000 1.329683000  7 -16.252476000 -8.081289000 0.543713000  1 -15.769279000 -8.930515000 0.265934000  6 -17.636123000 -8.015366000 0.247699000  8 -18.196177000 -8.966661000 -0.307199000  6 -18.242501000 -6.766270000 0.663901000  1 -19.302832000 -6.616062000 0.492153000  6 -14.538579000 -4.092394000 1.067081000  1 -14.392608000 -4.666173000 0.144220000  8 -13.291902000 -3.721524000 1.635491000  1 -12.844949000 -3.122315000 0.989705000  6 -15.436758000 -2.853785000 0.856420000  1 -16.261234000 -3.103814000 0.169542000  8 -14.712470000 -1.713985000 0.421108000  15 -14.666116000 -1.443807000 -1.251302000  8 -15.840813000 -0.563575000 -1.612614000  8 -14.404228000 -2.761173000 -1.948552000  8 -13.313626000 -0.471137000 -1.242423000  6 -12.061059000 -0.985561000 -1.723501000  1 -11.603273000 -0.199758000 -2.341930000  1 -12.226446000 -1.878569000 -2.339547000  6 -11.078146000 -1.316843000 -0.616337000  1 -10.993304000 -0.493269000 0.106135000  8 -11.507717000 -2.522656000 0.075193000  6 -10.371875000 -3.268688000 0.554531000  1 -10.355247000 -3.239320000 1.649732000  7 -10.501726000 -4.670127000 0.188944000  6 -10.266240000 -5.238864000 -1.055291000  7 -10.108290000 -4.563226000 -2.209134000  6 -9.686551000 -5.325378000 -3.219493000  7 -9.427274000 -4.753469000 -4.422129000  1 -9.286724000 -3.736489000 -4.381142000  1 -8.808983000 -5.305272000 -5.029958000  7 -9.505143000 -6.687490000 -3.093010000  1 -8.989373000 -7.144106000 -3.857475000  6 -9.728037000 -7.459327000 -1.912172000  8 -9.496298000 -8.676515000 -1.916568000  6 -10.176118000 -6.620188000 -0.827444000  7 -10.379192000 -6.909763000 0.525461000  6 -10.565180000 -5.733779000 1.094004000  1 -10.760918000 -5.546363000 2.144137000  6 -9.114458000 -2.577888000 -0.020009000  1 -8.396309000 -3.317714000 -0.393818000  8 -8.552750000 -1.820100000 1.046534000  1 -7.794631000 -1.287985000 0.700033000  6 -9.717910000 -1.749723000 -1.172761000  1 -9.915199000 -2.462955000 -1.979197000  8 -8.932755000 -0.668718000 -1.672523000  15 -8.075746000 -0.978591000 -3.111283000  8 -8.912332000 -1.851102000 -4.027646000  8 -7.542336000 0.368794000 -3.517724000  8 -6.803562000 -1.849342000 -2.488962000  6 -6.824096000 -3.289478000 -2.576770000  1 -7.845221000 -3.656630000 -2.697884000  1 -6.422193000 -3.665598000 -1.631656000  6 -5.982530000 -3.765135000 -3.753866000  1 -6.417235000 -3.408594000 -4.690493000  8 -4.646931000 -3.193262000 -3.710254000  6 -3.756394000 -4.073567000 -3.045285000  1 -2.853656000 -4.177693000 -3.657340000  7 -3.282039000 -3.428093000 -1.793972000  6 -3.929394000 -3.214621000 -0.580916000  7 -4.984444000 -3.896494000 -0.070764000  6 -5.449935000 -3.390355000 1.067193000  7 -6.520529000 -3.970274000 1.707105000  1 -6.925354000 -4.723388000 1.159468000  1 -7.234473000 -3.304390000 2.012362000  7 -4.897683000 -2.292772000 1.684973000  1 -5.233055000 -2.008683000 2.619429000  6 -3.812573000 -1.540750000 1.192660000  8 -3.471668000 -0.490799000 1.790214000  6 -3.280722000 -2.101505000 -0.005533000  7 -2.256864000 -1.638433000 -0.823619000  6 -2.294807000 -2.437007000 -1.871250000  1 -1.678836000 -2.367351000 -2.758721000  6 -4.472441000 -5.450032000 -2.889323000  1 -4.781276000 -5.585904000 -1.846921000  8 -3.664073000 -6.550192000 -3.227108000  1 -3.421822000 -6.483867000 -4.178847000  6 -5.753837000 -5.277830000 -3.765942000  1 -6.605104000 -5.838456000 -3.362990000  8 -5.464238000 -5.708596000 -5.104757000  15 -6.392245000 -6.864075000 -5.862146000  8 -5.648488000 -8.168583000 -6.000092000  8 -7.796522000 -6.854802000 -5.262347000  8 -6.496787000 -6.077498000 -7.325067000  6 -6.086465000 -6.739117000 -8.528858000  1 -6.936640000 -6.734052000 -9.225599000  1 -5.804167000 -7.778531000 -8.316793000  6 -4.896362000 -6.055846000 -9.174888000  1 -4.596024000 -6.658049000 -10.048000000  8 -5.251441000 -4.719807000 -9.638062000  6 -4.165675000 -3.833898000 -9.423407000  1 -3.969322000 -3.247511000 -10.329618000  7 -4.507638000 -2.899461000 -8.329084000  6 -3.683877000 -1.891137000 -7.850742000  7 -2.530654000 -1.436965000 -8.383975000  6 -1.995818000 -0.468002000 -7.620762000  1 -1.094743000 0.012255000 -8.007735000  7 -2.412917000 -0.021671000 -6.419320000  6 -3.525535000 -0.571720000 -5.858349000  7 -3.832882000 -0.260460000 -4.569528000  1 -4.442427000 -0.935162000 -4.111223000  1 -3.011601000 -0.004221000 -4.019012000  6 -4.293343000 -1.456544000 -6.654666000  7 -5.481119000 -2.141765000 -6.432150000  6 -5.569058000 -2.998556000 -7.438503000  1 -6.340527000 -3.744077000 -7.577006000  6 -2.958509000 -4.688483000 -9.008219000  1 -2.259020000 -4.137046000 -8.369519000  8 -2.315751000 -5.170280000 -10.186038000  1 -2.070742000 -6.090075000 -9.976243000  6 -3.673219000 -5.840710000 -8.276353000  1 -3.975188000 -5.533272000 -7.268742000  8 -2.891593000 -7.035750000 -8.226331000  15 -1.714413000 -7.134505000 -7.020999000  8 -1.140516000 -8.522136000 -7.135340000  8 -2.240782000 -6.544358000 -5.726811000  8 -0.647947000 -5.985029000 -7.562229000  6 0.367718000 -6.286377000 -8.527612000  1 0.217281000 -5.618817000 -9.386443000  1 0.297783000 -7.330265000 -8.863998000  6 1.742451000 -6.035675000 -7.917660000  1 2.504516000 -6.202841000 -8.692560000  8 1.843786000 -4.656884000 -7.477147000  6 1.867895000 -4.597346000 -6.049961000  1 2.850078000 -4.247572000 -5.707816000  7 0.900411000 -3.615004000 -5.571167000  6 1.199307000 -2.330268000 -5.145201000  7 2.408257000 -1.732428000 -5.167073000  6 2.401917000 -0.520040000 -4.614811000  7 3.532246000 0.232455000 -4.630663000  1 4.385700000 -0.282417000 -4.804916000  1 3.595007000 1.033512000 -4.002389000  7 1.268542000 0.056672000 -4.059548000  1 1.381643000 0.920133000 -3.526810000  6 -0.027782000 -0.516936000 -4.078291000  8 -0.997502000 0.083442000 -3.557502000  6 -0.024358000 -1.792901000 -4.705659000  7 -1.056279000 -2.710267000 -4.883509000  6 -0.479014000 -3.779531000 -5.399470000  1 -0.991030000 -4.696031000 -5.669235000  6 1.591332000 -6.025003000 -5.513760000  1 0.504196000 -6.165551000 -5.436063000  8 2.200647000 -6.242004000 -4.262353000  1 2.665081000 -7.133621000 -4.293575000  6 2.069697000 -6.899124000 -6.692952000  1 1.543048000 -7.861715000 -6.730122000  8 3.496324000 -7.080542000 -6.649373000  15 4.001747000 -8.511476000 -5.944300000  8 3.406935000 -8.566948000 -4.534944000  8 3.802068000 -9.661334000 -6.898991000  8 5.612395000 -8.158392000 -5.894466000  6 6.218289000 -7.702912000 -4.671167000  1 7.058295000 -8.380892000 -4.457904000  1 5.506389000 -7.745544000 -3.839497000  6 6.794968000 -6.303447000 -4.777678000  1 7.318429000 -6.176519000 -5.739822000  8 5.769804000 -5.298551000 -4.650349000  6 6.358944000 -4.118414000 -4.130304000  1 6.709193000 -3.443268000 -4.928833000  7 5.302163000 -3.422156000 -3.386125000  6 5.405721000 -2.153405000 -2.843744000  7 6.450350000 -1.303801000 -2.992648000  6 6.277249000 -0.145360000 -2.357562000  7 7.269687000 0.778770000 -2.363449000  1 8.038384000 0.571484000 -2.987376000  1 7.068552000 1.768788000 -2.207698000  7 5.132939000 0.149551000 -1.640001000  1 5.090086000 1.022458000 -1.124358000  6 3.984899000 -0.695854000 -1.486418000  8 3.011712000 -0.307496000 -0.830334000  6 4.189943000 -1.939962000 -2.169340000  7 3.348481000 -3.037536000 -2.319146000  6 4.039643000 -3.904383000 -3.047922000  1 3.688432000 -4.870682000 -3.390511000  6 7.586294000 -4.531049000 -3.266190000  1 7.325777000 -4.453855000 -2.198679000  8 8.691789000 -3.712359000 -3.593989000  1 9.459101000 -4.149212000 -3.160255000  6 7.798221000 -6.038644000 -3.644360000  1 7.575222000 -6.666515000 -2.773174000  8 9.098070000 -6.326179000 -4.155224000  15 10.297862000 -6.877420000 -3.135792000  8 11.525857000 -7.028880000 -4.002714000  8 9.802352000 -7.997529000 -2.248871000  8 10.373694000 -5.449896000 -2.239300000  6 11.327847000 -5.385638000 -1.162840000  1 11.391357000 -6.347507000 -0.642611000  1 10.953677000 -4.641590000 -0.452764000  6 12.710736000 -5.005712000 -1.677817000  1 13.058498000 -5.763333000 -2.384410000  8 12.637572000 -3.745387000 -2.419371000  6 13.124948000 -2.717019000 -1.598935000  1 13.507611000 -1.920417000 -2.246660000  7 12.014797000 -2.115235000 -0.791008000  6 11.987573000 -1.673855000 0.530995000  7 12.851548000 -1.935046000 1.535104000  6 12.510889000 -1.301675000 2.664857000  1 13.147580000 -1.494107000 3.523088000  7 11.490050000 -0.446506000 2.880542000  6 10.643545000 -0.168588000 1.847346000  7 9.737035000 0.813805000 1.989832000  1 8.930584000 0.918051000 1.370093000  1 9.644462000 1.282272000 2.879635000  6 10.833022000 -0.876259000 0.632134000  7 10.148149000 -0.845668000 -0.576642000  6 10.876751000 -1.594687000 -1.392542000  1 10.631703000 -1.822882000 -2.422739000  6 14.248271000 -3.332919000 -0.755160000  1 14.413972000 -2.813114000 0.194764000  8 15.416978000 -3.317313000 -1.570391000  1 15.783511000 -4.218690000 -1.469271000  6 13.739719000 -4.788066000 -0.540067000  1 13.268513000 -4.897579000 0.445616000  8 14.893175000 -5.632268000 -0.640341000  15 14.825180000 -7.249819000 -0.174234000  8 16.211500000 -7.772079000 -0.461431000  8 13.565320000 -7.891793000 -0.701306000  8 14.577633000 -7.088120000 1.464389000  6 15.520020000 -6.360739000 2.258375000  1 15.524953000 -5.294309000 1.981599000  1 16.537844000 -6.758443000 2.128019000  6 15.057488000 -6.522661000 3.703629000  1 15.037786000 -7.589883000 3.967644000  8 15.988057000 -5.848275000 4.595673000  6 15.284646000 -4.970947000 5.462816000  1 15.731555000 -5.040771000 6.462506000  7 15.448183000 -3.575211000 4.971633000  6 14.758467000 -2.420522000 5.334500000  7 13.612710000 -2.341336000 6.060672000  6 13.146355000 -1.092212000 6.172576000  7 11.991605000 -0.830934000 6.829373000  1 11.321321000 -1.588164000 7.033559000  1 11.544021000 0.057250000 6.630177000  7 13.811792000 0.003660000 5.657698000  1 13.372622000 0.923628000 5.792989000  6 15.000290000 -0.017312000 4.879225000  8 15.473792000 1.038819000 4.436058000  6 15.471215000 -1.368687000 4.721167000  7 16.565873000 -1.849606000 4.011812000  6 16.526265000 -3.157544000 4.190572000  1 17.223606000 -3.881114000 3.789396000  6 13.823144000 -5.412845000 5.445734000  1 13.144297000 -4.593955000 5.699389000  8 13.679844000 -6.509232000 6.344560000  1 13.026135000 -7.085357000 5.902935000  6 13.677634000 -5.909171000 3.991783000  1 13.484403000 -5.072071000 3.308643000  8 12.652056000 -6.896613000 3.897402000  15 11.157197000 -6.375934000 3.309149000  8 11.362420000 -5.697655000 1.969772000  8 10.225764000 -7.540236000 3.513060000  8 10.776303000 -5.229212000 4.452910000  6 10.979466000 -3.829301000 4.187667000  1 11.467603000 -3.687210000 3.218171000  1 11.623896000 -3.425400000 4.978959000  6 9.668387000 -3.063650000 4.206597000  1 9.898525000 -2.015661000 4.428091000  8 9.030845000 -3.101301000 2.888260000  6 7.723864000 -3.629100000 3.006096000  1 6.959408000 -2.839108000 3.061936000  7 7.423719000 -4.426687000 1.823262000  6 6.186303000 -4.537962000 1.219306000  7 5.111496000 -3.736100000 1.437430000  6 4.060365000 -4.024793000 0.663854000  7 2.930331000 -3.272059000 0.735930000  1 3.018000000 -2.428878000 1.316666000  1 2.467566000 -3.121567000 -0.160373000  7 4.067213000 -5.093886000 -0.201278000  1 3.244181000 -5.244714000 -0.775910000  6 5.191604000 -5.910199000 -0.543093000  8 5.084736000 -6.759901000 -1.435474000  6 6.320581000 -5.571964000 0.278766000  7 7.613321000 -6.083532000 0.298265000  6 8.247430000 -5.386794000 1.228180000  1 9.296800000 -5.479478000 1.500787000  6 7.718799000 -4.465163000 4.310870000  1 8.194533000 -5.432409000 4.106818000  8 6.432403000 -4.707354000 4.833969000  1 6.162013000 -3.871808000 5.258019000  6 8.647867000 -3.630949000 5.205047000  1 9.129441000 -4.219274000 5.989566000  8 7.820359000 -2.611379000 5.788348000  15 8.461764000 -1.698689000 7.030629000  8 7.321046000 -1.385908000 7.971168000  8 9.733933000 -2.356410000 7.538383000  8 8.962555000 -0.326851000 6.236229000  6 8.343633000 0.203232000 5.054998000  1 8.349761000 -0.537405000 4.244854000  1 8.993732000 1.037321000 4.770106000  6 6.941968000 0.776426000 5.189942000  1 6.808166000 1.455173000 4.337704000  8 5.943066000 -0.264788000 5.115777000  6 4.794445000 0.207902000 5.818643000  1 4.178562000 0.874906000 5.202208000  7 3.974011000 -0.966115000 6.126141000  6 4.579668000 -2.086970000 6.653448000  1 5.622266000 -1.949266000 6.943694000  6 2.608319000 -0.944684000 5.795706000  8 2.007871000 0.060795000 5.425805000  7 1.985467000 -2.177004000 5.946368000  1 0.999045000 -2.188321000 5.705148000  6 2.526787000 -3.388917000 6.433811000  8 1.817726000 -4.398517000 6.509926000  6 3.919988000 -3.267803000 6.816817000  1 4.424404000 -4.129961000 7.241016000  6 5.358874000 0.967928000 7.043078000  1 5.619310000 0.243443000 7.822003000  8 4.477823000 1.934544000 7.580159000  1 4.702783000 2.757013000 7.097455000  6 6.664052000 1.587322000 6.468667000  1 7.486426000 1.538515000 7.185777000  8 6.407725000 2.950041000 6.108837000  15 7.160279000 4.202056000 6.953734000  8 8.151951000 3.617243000 7.934347000  8 6.068575000 5.166882000 7.343663000  8 7.961256000 4.928369000 5.681927000  6 9.163186000 4.333927000 5.170991000  1 9.306579000 3.315006000 5.551938000  1 10.025225000 4.935135000 5.499747000  6 9.142047000 4.325503000 3.645475000  1 10.167070000 4.179767000 3.277498000  8 8.319108000 3.221179000 3.166414000  6 7.353444000 3.709934000 2.225207000  1 7.733437000 3.629020000 1.195626000  7 6.156001000 2.888418000 2.320444000  6 6.027634000 1.581511000 1.856336000  7 6.940865000 0.881811000 1.134750000  6 6.597176000 -0.404018000 0.969841000  7 7.426504000 -1.257576000 0.332603000  1 8.267787000 -0.912912000 -0.145187000  1 7.075469000 -2.156140000 0.034044000  7 5.427272000 -0.934111000 1.455991000  1 5.302877000 -1.969221000 1.435654000  6 4.405457000 -0.229832000 2.127520000  8 3.372483000 -0.829640000 2.477782000  6 4.774697000 1.144286000 2.323243000  7 4.145401000 2.145872000 3.055573000  6 4.992033000 3.155353000 3.049231000  1 4.829248000 4.105427000 3.536262000  6 7.134652000 5.185567000 2.630439000  1 6.549502000 5.186050000 3.559412000  8 6.478895000 5.979665000 1.673751000  1 7.133671000 6.528405000 1.166845000  6 8.555304000 5.613764000 3.048302000  1 8.525367000 6.423755000 3.782912000  8 9.320606000 6.037247000 1.908939000  15 9.252141000 7.695293000 1.585332000  8 8.168158000 7.887861000 0.533705000  8 9.238119000 8.464070000 2.885283000  8 10.703707000 7.856766000 0.807012000  6 11.862510000 7.192747000 1.334334000  1 11.562365000 6.439179000 2.070100000  1 12.524736000 7.921054000 1.823136000  6 12.586979000 6.520095000 0.156719000  1 13.046570000 7.276203000 -0.489278000  8 13.668503000 5.672405000 0.649633000  6 13.260126000 4.325542000 0.679629000  1 14.006514000 3.691971000 0.189373000  7 13.157955000 3.851005000 2.103895000  6 13.204948000 4.689836000 3.183153000  1 13.517212000 5.703791000 2.957257000  6 12.912347000 2.477041000 2.257146000  8 13.023568000 1.687726000 1.322590000  7 12.507560000 2.109231000 3.529345000  1 12.180100000 1.124997000 3.595906000  6 12.510505000 2.902336000 4.676832000  8 12.207719000 2.400898000 5.783778000  6 12.904123000 4.272148000 4.449820000  1 12.971620000 4.950952000 5.292531000  6 11.863625000 4.208233000 -0.028770000  1 11.094318000 4.036075000 0.735345000  8 11.774304000 3.187967000 -0.994686000  1 11.952524000 3.655650000 -1.838231000  6 11.671599000 5.613829000 -0.656611000  1 10.641823000 5.962774000 -0.586325000  8 12.092066000 5.545250000 -2.026851000  15 10.976671000 6.069835000 -3.204796000  8 11.762284000 6.121535000 -4.487089000  8 10.207228000 7.230869000 -2.627245000  8 10.009046000 4.713402000 -3.325360000  6 8.966666000 4.464135000 -2.373035000  1 8.940823000 5.228125000 -1.586406000  1 9.161037000 3.489240000 -1.904204000  6 7.618276000 4.437568000 -3.089894000  1 7.730923000 3.975681000 -4.084066000  8 6.714249000 3.642005000 -2.275419000  6 5.378578000 3.905296000 -2.730217000  1 5.073624000 3.192725000 -3.502819000  7 4.482841000 3.722457000 -1.592997000  6 4.681945000 4.498691000 -0.463158000  1 5.645438000 4.993810000 -0.397513000  6 3.294580000 3.003698000 -1.776652000  8 3.058761000 2.370381000 -2.817378000  7 2.421008000 3.043003000 -0.720806000  1 1.466986000 2.716428000 -0.991874000  6 2.479444000 3.922584000 0.372811000  8 1.501727000 4.049425000 1.124833000  6 3.746051000 4.614170000 0.508837000  1 3.930690000 5.244667000 1.369113000  6 5.375976000 5.333825000 -3.329385000  1 4.731246000 5.998512000 -2.733909000  8 4.922057000 5.231891000 -4.663057000  1 4.945132000 6.143801000 -5.024952000  6 6.866989000 5.771995000 -3.208726000  1 7.002028000 6.343410000 -2.280040000  8 7.319994000 6.527901000 -4.320484000  15 6.958310000 8.165620000 -4.348422000  8 6.760919000 8.700600000 -2.949558000  8 7.901780000 8.770366000 -5.355978000  8 5.428662000 8.045281000 -5.087728000  6 4.343042000 8.764380000 -4.485251000  1 4.601802000 9.819614000 -4.313865000  1 4.101053000 8.327864000 -3.504837000  6 3.147720000 8.708265000 -5.421722000  1 3.361433000 9.281439000 -6.336316000  8 2.884942000 7.331742000 -5.799326000  6 1.496163000 7.041103000 -5.688479000  1 1.152828000 6.489031000 -6.570961000  7 1.273859000 6.154884000 -4.536519000  6 0.488127000 5.012601000 -4.522853000  7 -0.269003000 4.556111000 -5.542965000  6 -0.865930000 3.390891000 -5.260749000  7 -1.663289000 2.794120000 -6.177833000  1 -1.600581000 3.176024000 -7.112582000  1 -1.899577000 1.788899000 -6.105903000  7 -0.740861000 2.762038000 -4.042232000  1 -1.080868000 1.792431000 -3.907521000  6 0.016476000 3.221084000 -2.951810000  8 0.042848000 2.515425000 -1.912621000  6 0.679119000 4.449862000 -3.244734000  7 1.555065000 5.227755000 -2.493450000  6 1.889784000 6.222986000 -3.289138000  1 2.573879000 7.018847000 -3.035307000  6 0.777120000 8.384695000 -5.538564000  1 -0.168639000 8.294481000 -4.994908000  8 0.569587000 8.936673000 -6.838111000  1 0.733133000 9.891915000 -6.722659000  6 1.835534000 9.215795000 -4.790105000  1 1.809658000 9.000493000 -3.714261000  8 1.655698000 10.613194000 -5.005435000  15 0.960547000 11.490836000 -3.722582000  8 0.944125000 12.915777000 -4.210791000  8 1.585472000 11.033690000 -2.428640000  8 -0.588347000 10.856409000 -3.706795000  6 -1.486387000 11.262313000 -4.750179000  1 -1.004238000 11.158956000 -5.733167000  1 -1.777653000 12.314764000 -4.618748000  6 -2.735953000 10.402655000 -4.725903000  1 -3.420909000 10.776165000 -5.506008000  8 -2.375317000 9.030968000 -5.021044000  6 -3.237142000 8.131574000 -4.328708000  1 -3.731654000 7.450393000 -5.025695000  7 -2.462221000 7.271728000 -3.410627000  6 -1.474911000 7.797101000 -2.623244000  1 -1.151816000 8.806491000 -2.867970000  6 -2.933075000 5.926854000 -3.213603000  8 -3.835242000 5.497975000 -3.954533000  7 -2.358381000 5.193086000 -2.221641000  6 -1.419214000 5.727804000 -1.430507000  7 -0.953397000 4.965764000 -0.411751000  1 -0.038975000 5.156560000 -0.017142000  1 -1.193618000 3.980520000 -0.445769000  6 -0.921058000 7.062935000 -1.615472000  1 -0.119691000 7.466197000 -1.005155000  6 -4.258192000 8.978153000 -3.557577000  1 -4.496061000 8.522718000 -2.591286000  8 -5.426204000 9.119811000 -4.355208000  1 -6.027761000 9.631234000 -3.775831000  6 -3.513502000 10.325074000 -3.405411000  1 -2.825022000 10.284423000 -2.552633000  8 -4.382377000 11.450104000 -3.272909000  15 -5.185752000 11.647174000 -1.816864000  8 -4.325176000 11.181831000 -0.663723000  8 -5.796795000 13.025052000 -1.883379000  8 -6.405345000 10.495749000 -2.087892000  6 -6.904213000 9.760287000 -0.951813000  1 -7.735999000 10.311294000 -0.484125000  1 -6.101858000 9.630481000 -0.213386000  6 -7.426807000 8.408000000 -1.385624000  1 -8.117548000 8.516908000 -2.238465000  8 -6.322656000 7.551526000 -1.753580000  6 -6.711999000 6.187817000 -1.601645000  1 -6.856547000 5.693939000 -2.571729000  7 -5.633653000 5.476103000 -0.928544000  6 -5.287280000 4.153620000 -1.150519000  7 -5.770049000 3.313615000 -2.083071000  6 -5.175537000 2.107461000 -2.015654000  1 -5.534267000 1.362259000 -2.726109000  7 -4.222189000 1.688090000 -1.150879000  6 -3.773458000 2.555909000 -0.207516000  7 -2.790765000 2.146645000 0.652734000  1 -2.779206000 2.653369000 1.533081000  1 -2.728228000 1.133874000 0.776736000  6 -4.320671000 3.859030000 -0.169179000  7 -4.092959000 4.960254000 0.650217000  6 -4.893195000 5.898849000 0.169331000  1 -4.982864000 6.911271000 0.544142000  6 -8.018715000 6.168063000 -0.785647000  1 -7.933987000 5.454319000 0.040533000  8 -9.092452000 5.837090000 -1.652359000  1 -9.878254000 5.902698000 -1.072869000  6 -8.127625000 7.622059000 -0.265725000  1 -7.564259000 7.718287000 0.672902000  8 -9.477207000 8.018079000 -0.086006000  15 -9.958124000 8.591316000 1.426169000  8 -10.161676000 10.082754000 1.299053000  8 -9.080401000 7.986468000 2.505316000  8 -11.487200000 7.916620000 1.467514000  6 -11.708425000 6.715932000 2.218917000  1 -12.693904000 6.803450000 2.700539000  1 -10.945572000 6.613227000 3.001252000  6 -11.738693000 5.468295000 1.350665000  1 -12.500804000 5.567870000 0.564327000  8 -10.454013000 5.264011000 0.709272000  6 -10.026458000 3.914793000 0.830178000  1 -9.710876000 3.538038000 -0.150157000  7 -8.839179000 3.870863000 1.703471000  6 -7.864078000 2.894146000 1.636796000  7 -7.880007000 1.822903000 0.806006000  6 -6.774900000 1.096460000 0.894114000  7 -6.639144000 -0.009545000 0.071330000  1 -7.301133000 0.033718000 -0.707674000  1 -5.690818000 -0.169743000 -0.263169000  7 -5.764627000 1.349186000 1.786197000  1 -4.951546000 0.717002000 1.826285000  6 -5.657431000 2.509486000 2.616654000  8 -4.631273000 2.688326000 3.286373000  6 -6.844589000 3.315573000 2.507700000  7 -7.170905000 4.542104000 3.076031000  6 -8.360524000 4.849840000 2.579123000  1 -8.878754000 5.794199000 2.719107000  6 -11.219751000 3.113594000 1.364939000  1 -10.908350000 2.259137000 1.977534000  8 -11.995738000 2.686184000 0.246428000  1 -12.920769000 2.848535000 0.514569000  6 -11.997723000 4.184866000 2.159144000  1 -11.598337000 4.277027000 3.178123000  8 -13.396127000 3.908809000 2.188044000  15 -14.043076000 3.293028000 3.634453000  8 -13.441310000 4.042546000 4.796274000  8 -15.519560000 3.168213000 3.365548000  8 -13.317388000 1.793781000 3.666905000  6 -13.746775000 0.790540000 2.736905000  1 -13.678273000 1.157960000 1.704045000  1 -14.788104000 0.498886000 2.936116000  6 -12.863358000 -0.427900000 2.886321000  1 -13.241762000 -1.194286000 2.199406000  8 -11.495193000 -0.113762000 2.524113000  6 -10.591767000 -0.772721000 3.401951000  1 -9.832480000 -1.307597000 2.823998000  7 -9.859376000 0.246631000 4.178206000  6 -8.523074000 0.155930000 4.508295000  7 -7.733466000 -0.930585000 4.319928000  6 -6.488683000 -0.732713000 4.717562000  7 -5.573436000 -1.760775000 4.535572000  1 -6.012043000 -2.674376000 4.622000000  1 -4.717399000 -1.703568000 5.078591000  7 -6.040781000 0.449322000 5.250139000  1 -5.066778000 0.548469000 5.519433000  6 -6.841294000 1.609425000 5.523605000  8 -6.328666000 2.588052000 6.078650000  6 -8.190638000 1.392017000 5.087526000  7 -9.308125000 2.219780000 5.136528000  6 -10.283933000 1.509793000 4.586043000  1 -11.307034000 1.832573000 4.430559000  6 -11.424444000 -1.739169000 4.278729000  1 -10.975837000 -1.863178000 5.268954000  8 -11.506201000 -3.024943000 3.674398000  1 -12.358452000 -3.128679000 3.201423000  6 -12.771561000 -0.999108000 4.309196000  1 -12.736661000 -0.184316000 5.041629000  8 -13.889094000 -1.844183000 4.553964000  15 -14.760406000 -1.690868000 5.972416000  8 -14.654843000 -0.292062000 6.519647000  8 -16.082801000 -2.380045000 5.687254000  8 -13.856821000 -2.641187000 6.998401000  6 -13.596908000 -4.007225000 6.643399000  1 -13.369486000 -4.089476000 5.572010000  1 -14.476053000 -4.631449000 6.867277000  6 -12.400235000 -4.502055000 7.440121000  1 -12.315195000 -5.593608000 7.312442000  8 -11.198338000 -3.874535000 6.927610000  6 -10.336844000 -3.494006000 7.988255000  1 -9.321590000 -3.873429000 7.818412000  7 -10.216006000 -2.031429000 8.020604000  6 -9.032967000 -1.339127000 8.229593000  7 -7.805182000 -1.845506000 8.447759000  6 -6.895490000 -0.861690000 8.594505000  1 -5.870223000 -1.188477000 8.782958000  7 -7.073630000 0.472320000 8.530717000  6 -8.320247000 0.948063000 8.277896000  7 -8.497896000 2.301353000 8.180075000  1 -9.298158000 2.563784000 7.609667000  1 -7.651536000 2.782976000 7.879754000  6 -9.383076000 0.023617000 8.141751000  7 -10.742767000 0.169674000 7.880246000  6 -11.205954000 -1.071413000 7.819778000  1 -12.228652000 -1.359884000 7.603958000  6 -10.920314000 -4.093580000 9.280947000  1 -10.705654000 -3.458690000 10.154921000  8 -10.380466000 -5.401421000 9.434926000  1 -11.121687000 -5.937066000 9.775830000  1 -12.903366000 -3.203074000 9.117141000  6 -12.420211000 -4.175660000 8.950563000  8 -13.000369000 -5.188812000 9.768111000  1 -13.884145000 -5.392554000 9.425948000 | 1 -16.986412000 -1.515582000 4.344358000  8 -17.377521000 -0.948998000 3.628287000  6 -17.258664000 -1.627693000 2.385608000  1 -17.201450000 -0.860253000 1.597283000  1 -18.145576000 -2.255347000 2.165404000  6 -16.009903000 -2.487531000 2.269372000  1 -15.212420000 -2.034213000 2.864674000  8 -16.251412000 -3.831646000 2.795358000  6 -15.405819000 -4.752835000 2.139417000  1 -14.733824000 -5.254713000 2.845398000  7 -16.241175000 -5.825786000 1.516673000  6 -17.578492000 -5.649748000 1.261245000  1 -17.975344000 -4.699420000 1.603986000  6 -15.564947000 -6.998313000 1.154263000  8 -14.360279000 -7.160731000 1.353238000  7 -16.374412000 -7.948038000 0.551586000  1 -15.903260000 -8.805586000 0.278736000  6 -17.753728000 -7.858387000 0.241842000  8 -18.324751000 -8.800421000 -0.317644000  6 -18.342296000 -6.598383000 0.650704000  1 -19.398082000 -6.429787000 0.468266000  6 -14.605920000 -3.985248000 1.072346000  1 -14.466563000 -4.563423000 0.151325000  8 -13.354717000 -3.629924000 1.640973000  1 -12.896343000 -3.043349000 0.991608000  6 -15.486573000 -2.735042000 0.856267000  1 -16.314481000 -2.976599000 0.170327000  8 -14.747226000 -1.606767000 0.416543000  15 -14.682423000 -1.357527000 -1.258726000  8 -15.844657000 -0.469755000 -1.641377000  8 -14.428018000 -2.686041000 -1.937380000  8 -13.319593000 -0.399494000 -1.248327000  6 -12.071033000 -0.929616000 -1.722273000  1 -11.601119000 -0.150866000 -2.340509000  1 -12.244018000 -1.822208000 -2.336773000  6 -11.097125000 -1.269628000 -0.609805000  1 -11.002893000 -0.443575000 0.108835000  8 -11.546305000 -2.465473000 0.086025000  6 -10.422475000 -3.223545000 0.575428000  1 -10.409184000 -3.185786000 1.670358000  7 -10.571160000 -4.625445000 0.220543000  6 -10.337646000 -5.207019000 -1.017924000  7 -10.162461000 -4.542057000 -2.175396000  6 -9.747734000 -5.318078000 -3.178015000  7 -9.475224000 -4.759383000 -4.383899000  1 -9.323185000 -3.743801000 -4.350793000  1 -8.860829000 -5.323802000 -4.983972000  7 -9.587747000 -6.681817000 -3.040348000  1 -9.073858000 -7.151635000 -3.797786000  6 -9.829368000 -7.441627000 -1.855389000  8 -9.616056000 -8.662197000 -1.849703000  6 -10.271131000 -6.587876000 -0.779526000  7 -10.486789000 -6.864037000 0.574302000  6 -10.657304000 -5.681058000 1.133082000  1 -10.855606000 -5.482618000 2.180686000  6 -9.153289000 -2.554421000 0.000772000  1 -8.444575000 -3.306659000 -0.366344000  8 -8.585244000 -1.799287000 1.065875000  1 -7.823555000 -1.272076000 0.719305000  6 -9.741439000 -1.725166000 -1.159213000  1 -9.945888000 -2.441249000 -1.961354000  8 -8.940636000 -0.658538000 -1.665092000  15 -8.086395000 -0.987519000 -3.101927000  8 -8.932143000 -1.859149000 -4.010659000  8 -7.540563000 0.351472000 -3.519165000  8 -6.822276000 -1.865165000 -2.473483000  6 -6.857804000 -3.305694000 -2.549153000  1 -7.882508000 -3.663410000 -2.668997000  1 -6.461755000 -3.678279000 -1.600226000  6 -6.019649000 -3.800288000 -3.721114000  1 -6.451727000 -3.450945000 -4.661650000  8 -4.679511000 -3.238731000 -3.684822000  6 -3.797498000 -4.115816000 -3.004735000  1 -2.891745000 -4.230116000 -3.610203000  7 -3.326442000 -3.459488000 -1.757162000  6 -3.975062000 -3.235240000 -0.546819000  7 -5.033387000 -3.910083000 -0.034042000  6 -5.499913000 -3.393982000 1.098933000  7 -6.574955000 -3.964439000 1.739869000  1 -6.981996000 -4.719350000 1.196310000  1 -7.286174000 -3.292198000 2.037570000  7 -4.945388000 -2.293815000 1.710154000  1 -5.280526000 -2.003957000 2.642809000  6 -3.855968000 -1.549278000 1.215625000  8 -3.512203000 -0.497203000 1.807770000  6 -3.323790000 -2.120169000 0.022095000  7 -2.295978000 -1.667408000 -0.796756000  6 -2.334823000 -2.473160000 -1.838996000  1 -1.716089000 -2.411651000 -2.725144000  6 -4.521345000 -5.487326000 -2.838456000  1 -4.829105000 -5.614928000 -1.794770000  8 -3.719503000 -6.594219000 -3.170603000  1 -3.486862000 -6.540590000 -4.125371000  6 -5.802945000 -5.314849000 -3.715568000  1 -6.658300000 -5.863732000 -3.305105000  8 -5.517724000 -5.764947000 -5.048811000  15 -6.461681000 -6.913763000 -5.796743000  8 -5.732698000 -8.227038000 -5.929902000  8 -7.863138000 -6.886171000 -5.191463000  8 -6.562961000 -6.134095000 -7.263854000  6 -6.170353000 -6.809611000 -8.466006000  1 -7.025247000 -6.797770000 -9.156876000  1 -5.900572000 -7.851262000 -8.248781000  6 -4.975520000 -6.146934000 -9.124783000  1 -4.686862000 -6.760431000 -9.993949000  8 -5.316788000 -4.810884000 -9.598339000  6 -4.219599000 -3.936243000 -9.396446000  1 -4.020799000 -3.359937000 -10.308583000  7 -4.545471000 -2.988581000 -8.308403000  6 -3.706681000 -1.987995000 -7.840209000  7 -2.548151000 -1.554760000 -8.379300000  6 -1.997808000 -0.587646000 -7.624926000  1 -1.090261000 -0.124178000 -8.017277000  7 -2.406198000 -0.124953000 -6.426632000  6 -3.525733000 -0.654191000 -5.859136000  7 -3.826659000 -0.327819000 -4.572427000  1 -4.444859000 -0.990557000 -4.108168000  1 -3.000098000 -0.080552000 -4.025525000  6 -4.307210000 -1.535098000 -6.646416000  7 -5.503740000 -2.202022000 -6.415434000  6 -5.605871000 -3.065789000 -7.414478000  1 -6.387728000 -3.801936000 -7.544739000  6 -3.020567000 -4.800992000 -8.979007000  1 -2.312634000 -4.252124000 -8.347538000  8 -2.387820000 -5.300017000 -10.154894000  1 -2.147117000 -6.218741000 -9.935781000  6 -3.745533000 -5.938492000 -8.234190000  1 -4.039280000 -5.618677000 -7.228007000  8 -2.978061000 -7.142358000 -8.176921000  15 -1.794919000 -7.242768000 -6.977995000  8 -1.233232000 -8.636002000 -7.086035000  8 -2.308177000 -6.640152000 -5.684227000  8 -0.722928000 -6.105244000 -7.534293000  6 0.298207000 -6.428212000 -8.486858000  1 0.170262000 -5.758335000 -9.347513000  1 0.211029000 -7.470586000 -8.823881000  6 1.671637000 -6.204760000 -7.862934000  1 2.437952000 -6.388106000 -8.629937000  8 1.796362000 -4.827711000 -7.422672000  6 1.806071000 -4.767542000 -5.995180000  1 2.792002000 -4.439139000 -5.643502000  7 0.855083000 -3.763315000 -5.528062000  6 1.173604000 -2.476414000 -5.122889000  7 2.391330000 -1.895624000 -5.154852000  6 2.400864000 -0.673858000 -4.624489000  7 3.541388000 0.065363000 -4.650854000  1 4.390189000 -0.464466000 -4.803560000  1 3.607776000 0.869563000 -4.027781000  7 1.275987000 -0.070364000 -4.081267000  1 1.405726000 0.802147000 -3.566274000  6 -0.026315000 -0.627910000 -4.084844000  8 -0.986400000 -0.007555000 -3.569411000  6 -0.041555000 -1.914272000 -4.690638000  7 -1.087061000 -2.818794000 -4.852450000  6 -0.526505000 -3.904228000 -5.353006000  1 -1.053594000 -4.816122000 -5.610349000  6 1.490646000 -6.189021000 -5.462591000  1 0.399998000 -6.306236000 -5.404135000  8 2.069160000 -6.421931000 -4.199472000  1 2.535976000 -7.312444000 -4.228682000  6 1.969922000 -7.072407000 -6.633806000  1 1.428987000 -8.026704000 -6.676723000  8 3.393209000 -7.275551000 -6.572425000  15 3.869244000 -8.713964000 -5.862764000  8 3.275440000 -8.751059000 -4.452400000  8 3.644421000 -9.862573000 -6.813264000  8 5.487819000 -8.399139000 -5.815304000  6 6.100943000 -7.922308000 -4.603657000  1 6.949075000 -8.590658000 -4.393004000  1 5.397746000 -7.962983000 -3.764502000  6 6.664751000 -6.518859000 -4.728782000  1 7.173581000 -6.395744000 -5.699317000  8 5.633813000 -5.521209000 -4.594212000  6 6.223769000 -4.330579000 -4.101014000  1 6.558195000 -3.665999000 -4.915209000  7 5.174885000 -3.627956000 -3.351381000  6 5.286813000 -2.354398000 -2.824226000  7 6.335398000 -1.513288000 -2.989369000  6 6.174013000 -0.347456000 -2.365267000  7 7.174526000 0.567434000 -2.384837000  1 7.940101000 0.340475000 -3.005922000  1 6.977840000 1.562407000 -2.257259000  7 5.035045000 -0.036470000 -1.646665000  1 5.001448000 0.842102000 -1.140287000  6 3.880846000 -0.871478000 -1.479496000  8 2.912814000 -0.467183000 -0.825400000  6 4.075006000 -2.125410000 -2.147805000  7 3.227378000 -3.220501000 -2.281254000  6 3.911068000 -4.099446000 -3.002930000  1 3.553459000 -5.068000000 -3.332612000  6 7.466750000 -4.723703000 -3.250536000  1 7.222492000 -4.633800000 -2.180298000  8 8.562031000 -3.903475000 -3.607027000  1 9.338229000 -4.328392000 -3.177201000  6 7.681518000 -6.235220000 -3.611886000  1 7.472962000 -6.852558000 -2.729637000  8 8.976343000 -6.522674000 -4.135892000  15 10.196001000 -7.046328000 -3.125538000  8 11.412194000 -7.200669000 -4.008408000  8 9.724957000 -8.155831000 -2.212344000  8 10.273469000 -5.603466000 -2.253342000  6 11.227105000 -5.522223000 -1.177475000  1 11.302011000 -6.480943000 -0.653149000  1 10.845501000 -4.779354000 -0.470265000  6 12.604785000 -5.128342000 -1.695212000  1 12.955394000 -5.878456000 -2.408677000  8 12.520046000 -3.863086000 -2.427545000  6 13.007782000 -2.836973000 -1.604818000  1 13.387284000 -2.037094000 -2.250455000  7 11.897518000 -2.239467000 -0.792186000  6 11.886205000 -1.769758000 0.520267000  7 12.764161000 -2.007330000 1.517868000  6 12.432387000 -1.357964000 2.640961000  1 13.080470000 -1.532214000 3.494220000  7 11.407574000 -0.507243000 2.854897000  6 10.547171000 -0.251096000 1.827749000  7 9.632932000 0.725129000 1.968545000  1 8.814046000 0.807013000 1.361142000  1 9.541807000 1.195827000 2.857797000  6 10.728638000 -0.976133000 0.621377000  7 10.027501000 -0.974643000 -0.578417000  6 10.748872000 -1.736771000 -1.388705000  1 10.490520000 -1.987950000 -2.410274000  6 14.135043000 -3.451710000 -0.765351000  1 14.295986000 -2.937244000 0.188277000  8 15.304417000 -3.420448000 -1.579246000  1 15.676545000 -4.320511000 -1.486700000  6 13.637725000 -4.912381000 -0.560890000  1 13.172573000 -5.034748000 0.426127000  8 14.796459000 -5.747660000 -0.676195000  15 14.747448000 -7.364107000 -0.204231000  8 16.134518000 -7.876038000 -0.506007000  8 13.486727000 -8.017310000 -0.715133000  8 14.517795000 -7.199609000 1.436837000  6 15.460133000 -6.456635000 2.216579000  1 15.458290000 -5.393909000 1.926051000  1 16.479438000 -6.850708000 2.086440000  6 15.005511000 -6.602141000 3.665951000  1 14.991670000 -7.666055000 3.943584000  8 15.937418000 -5.911840000 4.544132000  6 15.233036000 -5.039582000 5.416100000  1 15.684776000 -5.107756000 6.413783000  7 15.386826000 -3.642443000 4.926050000  6 14.694389000 -2.491994000 5.296997000  7 13.551836000 -2.420536000 6.028907000  6 13.080180000 -1.174052000 6.146817000  7 11.926369000 -0.920263000 6.808325000  1 11.260057000 -1.681089000 7.012449000  1 11.473950000 -0.033734000 6.613028000  7 13.738788000 -0.073475000 5.633465000  1 13.295230000 0.843918000 5.772082000  6 14.923601000 -0.086212000 4.849325000  8 15.389648000 0.973767000 4.407173000  6 15.399657000 -1.434876000 4.683954000  7 16.492066000 -1.908741000 3.966361000  6 16.458677000 -3.217500000 4.140463000  1 17.156437000 -3.936828000 3.732441000  6 13.773853000 -5.489035000 5.406103000  1 13.091777000 -4.673028000 5.659880000  8 13.639729000 -6.583310000 6.309093000  1 12.984957000 -7.162730000 5.873483000  6 13.625112000 -5.991101000 3.954567000  1 13.428498000 -5.156792000 3.268705000  8 12.601713000 -6.981316000 3.866311000  15 11.102321000 -6.461621000 3.289488000  8 11.295824000 -5.786882000 1.946502000  8 10.171799000 -7.624867000 3.503773000  8 10.734789000 -5.312153000 4.434359000  6 10.919849000 -3.911572000 4.159784000  1 11.400561000 -3.768885000 3.186631000  1 11.564742000 -3.497089000 4.945151000  6 9.599458000 -3.162121000 4.183992000  1 9.816545000 -2.110661000 4.402468000  8 8.955707000 -3.211924000 2.868893000  6 7.653084000 -3.747454000 2.997785000  1 6.884243000 -2.962272000 3.061803000  7 7.346628000 -4.546099000 1.817426000  6 6.100870000 -4.670927000 1.232848000  7 5.025037000 -3.871888000 1.457190000  6 3.964347000 -4.174720000 0.702570000  7 2.831161000 -3.426825000 0.781355000  1 2.921279000 -2.578332000 1.353955000  1 2.359712000 -3.284763000 -0.111870000  7 3.964061000 -5.254282000 -0.149276000  1 3.132151000 -5.419035000 -0.707006000  6 5.087551000 -6.069008000 -0.498257000  8 4.970873000 -6.931585000 -1.376611000  6 6.227540000 -5.713458000 0.300923000  7 7.523180000 -6.218144000 0.307326000  6 8.166629000 -5.508883000 1.221178000  1 9.219526000 -5.594438000 1.482192000  6 7.666010000 -4.585192000 4.301209000  1 8.150791000 -5.546879000 4.092364000  8 6.386101000 -4.842450000 4.832683000  1 6.107242000 -4.009388000 5.256048000  6 8.591966000 -3.741484000 5.189546000  1 9.085913000 -4.325041000 5.969861000  8 7.756874000 -2.732595000 5.780038000  15 8.397579000 -1.813054000 7.017482000  8 7.258248000 -1.505830000 7.961374000  8 9.676222000 -2.461049000 7.521529000  8 8.887641000 -0.441466000 6.215865000  6 8.247073000 0.088073000 5.045697000  1 8.251147000 -0.648571000 4.231738000  1 8.883031000 0.930943000 4.756005000  6 6.840550000 0.643632000 5.201517000  1 6.689584000 1.328546000 4.357430000  8 5.852980000 -0.407896000 5.128280000  6 4.702899000 0.051387000 5.837225000  1 4.078181000 0.714504000 5.225524000  7 3.895610000 -1.131864000 6.143433000  6 4.515123000 -2.249417000 6.661643000  1 5.557591000 -2.102203000 6.947683000  6 2.528432000 -1.121960000 5.819012000  8 1.916764000 -0.119782000 5.458562000  7 1.918815000 -2.361540000 5.963745000  1 0.931596000 -2.381611000 5.726333000  6 2.474652000 -3.570818000 6.441379000  8 1.776563000 -4.588324000 6.513291000  6 3.867928000 -3.437770000 6.820565000  1 4.382334000 -4.297502000 7.237601000  6 5.263401000 0.813050000 7.062644000  1 5.526745000 0.089342000 7.841141000  8 4.377009000 1.774995000 7.599549000  1 4.604763000 2.600672000 7.123522000  6 6.566361000 1.439971000 6.490103000  1 7.390995000 1.383073000 7.203745000  8 6.308440000 2.806435000 6.146261000  15 7.063855000 4.049470000 7.001259000  8 8.059826000 3.455172000 7.971618000  8 5.973843000 5.010441000 7.404757000  8 7.860121000 4.789494000 5.733682000  6 9.062473000 4.203742000 5.214587000  1 9.217447000 3.186670000 5.595647000  1 9.923159000 4.812483000 5.533106000  6 9.021902000 4.195273000 3.689990000  1 10.040857000 4.051073000 3.303728000  8 8.187293000 3.096248000 3.228491000  6 7.251297000 3.572827000 2.252215000  1 7.655835000 3.469815000 1.234040000  7 6.049133000 2.756625000 2.335232000  6 5.921502000 1.449699000 1.869148000  7 6.832872000 0.752807000 1.142661000  6 6.493747000 -0.535461000 0.982313000  7 7.323084000 -1.386662000 0.344286000  1 8.165881000 -1.044690000 -0.133507000  1 6.981043000 -2.294303000 0.063686000  7 5.327066000 -1.068467000 1.472819000  1 5.206605000 -2.104236000 1.453926000  6 4.305782000 -0.366723000 2.146920000  8 3.275441000 -0.968663000 2.500670000  6 4.672285000 1.008521000 2.341686000  7 4.045332000 2.005786000 3.081738000  6 4.889321000 3.017098000 3.074609000  1 4.727578000 3.963251000 3.569756000  6 7.032166000 5.059298000 2.614949000  1 6.393892000 5.091399000 3.508818000  8 6.454562000 5.824393000 1.584173000  1 7.095765000 6.506281000 1.261298000  6 8.430401000 5.488711000 3.104101000  1 8.356859000 6.268526000 3.867578000  8 9.234222000 5.972507000 2.014843000  15 9.249124000 7.649416000 1.861046000  8 8.102848000 8.017934000 0.921954000  8 9.385844000 8.293924000 3.217989000  8 10.633226000 7.794291000 0.967892000  6 11.809887000 7.113296000 1.443340000  1 11.524748000 6.336821000 2.161006000  1 12.481851000 7.826559000 1.939516000  6 12.504838000 6.477188000 0.231142000  1 12.930467000 7.255764000 -0.411868000  8 13.616929000 5.642754000 0.674541000  6 13.240335000 4.287388000 0.687901000  1 14.015589000 3.678865000 0.211306000  7 13.110223000 3.799072000 2.107094000  6 13.184390000 4.619776000 3.198832000  1 13.527866000 5.627086000 2.988762000  6 12.829041000 2.429925000 2.240703000  8 12.915861000 1.653400000 1.292279000  7 12.417406000 2.053019000 3.507844000  1 12.076985000 1.071685000 3.563522000  6 12.441190000 2.830045000 4.666225000  8 12.127145000 2.321764000 5.766828000  6 12.869919000 4.192424000 4.458638000  1 12.956349000 4.856107000 5.311603000  6 11.867144000 4.136978000 -0.055996000  1 11.098631000 3.849500000 0.673569000  8 11.873608000 3.194301000 -1.101175000  1 11.990518000 3.740125000 -1.907315000  6 11.580031000 5.574034000 -0.577460000  1 10.540232000 5.868037000 -0.430882000  8 11.916334000 5.625000000 -1.973440000  15 10.685616000 6.095084000 -3.044718000  8 11.382845000 6.473627000 -4.320610000  8 9.758083000 7.006925000 -2.275727000  8 9.941083000 4.633746000 -3.322901000  6 8.917342000 4.169708000 -2.430397000  1 8.878614000 4.760016000 -1.506512000  1 9.153325000 3.129455000 -2.169257000  6 7.562219000 4.236525000 -3.129443000  1 7.645718000 3.841116000 -4.154734000  8 6.650367000 3.415030000 -2.350667000  6 5.312268000 3.734586000 -2.763824000  1 4.969126000 3.057407000 -3.551359000  7 4.432331000 3.545208000 -1.616047000  6 4.645758000 4.319774000 -0.486572000  1 5.615393000 4.803668000 -0.421746000  6 3.234662000 2.839210000 -1.792671000  8 2.988269000 2.204778000 -2.831453000  7 2.365435000 2.892890000 -0.734873000  1 1.403554000 2.583824000 -0.998379000  6 2.438629000 3.773289000 0.358732000  8 1.466624000 3.910208000 1.114589000  6 3.714470000 4.449920000 0.487617000  1 3.908975000 5.080449000 1.345710000  6 5.345522000 5.181465000 -3.311671000  1 4.693614000 5.838721000 -2.717795000  8 4.932612000 5.125631000 -4.662498000  1 4.919435000 6.050749000 -4.983623000  6 6.837243000 5.589518000 -3.137907000  1 6.977607000 6.070071000 -2.161271000  8 7.298497000 6.448366000 -4.172404000  15 6.891567000 8.063369000 -4.048625000  8 6.544380000 8.424922000 -2.615865000  8 7.915289000 8.831473000 -4.846530000  8 5.450824000 8.002099000 -4.933127000  6 4.326152000 8.724239000 -4.404390000  1 4.563019000 9.788795000 -4.257554000  1 4.048729000 8.312120000 -3.423995000  6 3.176308000 8.621296000 -5.391286000  1 3.415695000 9.176184000 -6.310591000  8 2.953046000 7.232160000 -5.742052000  6 1.564292000 6.920687000 -5.686739000  1 1.267675000 6.351796000 -6.575444000  7 1.309394000 6.046703000 -4.533132000  6 0.519525000 4.906942000 -4.530404000  7 -0.207535000 4.441686000 -5.568280000  6 -0.819988000 3.283277000 -5.290215000  7 -1.586953000 2.676081000 -6.225255000  1 -1.492392000 3.041244000 -7.163846000  1 -1.848887000 1.678475000 -6.139202000  7 -0.742012000 2.672835000 -4.058253000  1 -1.080462000 1.702577000 -3.925959000  6 -0.020958000 3.144270000 -2.948642000  8 -0.045135000 2.460598000 -1.895256000  6 0.667784000 4.359720000 -3.239954000  7 1.525388000 5.142255000 -2.471939000  6 1.887243000 6.127959000 -3.268428000  1 2.563920000 6.926526000 -3.002716000  6 0.817276000 8.255393000 -5.589839000  1 -0.144383000 8.158489000 -5.074943000  8 0.644463000 8.775099000 -6.906972000  1 0.772227000 9.737204000 -6.806057000  6 1.833663000 9.119510000 -4.821626000  1 1.771249000 8.922614000 -3.743730000  8 1.645704000 10.509099000 -5.071815000  15 0.975961000 11.422448000 -3.801294000  8 0.988641000 12.839801000 -4.310719000  8 1.595116000 10.972202000 -2.501674000  8 -0.584994000 10.819590000 -3.776135000  6 -1.460929000 11.206747000 -4.845778000  1 -0.957028000 11.088556000 -5.816138000  1 -1.755851000 12.260902000 -4.737690000  6 -2.709825000 10.346341000 -4.833349000  1 -3.381854000 10.709231000 -5.629581000  8 -2.343505000 8.971413000 -5.104580000  6 -3.214597000 8.080746000 -4.412442000  1 -3.699742000 7.390330000 -5.106968000  7 -2.450073000 7.234069000 -3.473608000  6 -1.470941000 7.771712000 -2.684711000  1 -1.147364000 8.778374000 -2.940114000  6 -2.921870000 5.891799000 -3.262699000  8 -3.817900000 5.452863000 -4.004914000  7 -2.353366000 5.170916000 -2.257267000  6 -1.419915000 5.716578000 -1.467342000  7 -0.955431000 4.966467000 -0.437812000  1 -0.037886000 5.165916000 -0.056436000  1 -1.178044000 3.976946000 -0.474764000  6 -0.923996000 7.050852000 -1.663542000  1 -0.128954000 7.463699000 -1.051210000  6 -4.246258000 8.935575000 -3.664031000  1 -4.490915000 8.492569000 -2.693649000  8 -5.407867000 9.060814000 -4.473605000  1 -6.015394000 9.579892000 -3.907657000  6 -3.507692000 10.287337000 -3.524227000  1 -2.832250000 10.263304000 -2.660411000  8 -4.380368000 11.412632000 -3.425802000  15 -5.197672000 11.643679000 -1.982482000  8 -4.340198000 11.225607000 -0.808993000  8 -5.824096000 13.011496000 -2.097293000  8 -6.400950000 10.470080000 -2.226110000  6 -6.883664000 9.748117000 -1.074489000  1 -7.711008000 10.302840000 -0.603260000  1 -6.072144000 9.629694000 -0.344268000  6 -7.409837000 8.389850000 -1.483747000  1 -8.105375000 8.485942000 -2.334158000  8 -6.310385000 7.523287000 -1.843301000  6 -6.702652000 6.163452000 -1.662554000  1 -6.850842000 5.650632000 -2.622148000  7 -5.625430000 5.461079000 -0.977925000  6 -5.279037000 4.135661000 -1.182615000  7 -5.754095000 3.287427000 -2.111523000  6 -5.163799000 2.080475000 -2.024999000  1 -5.515992000 1.329533000 -2.732590000  7 -4.223020000 1.666829000 -1.144193000  6 -3.782321000 2.542662000 -0.204048000  7 -2.815895000 2.136272000 0.674833000  1 -2.816097000 2.648009000 1.552422000  1 -2.757746000 1.124002000 0.804531000  6 -4.323670000 3.849000000 -0.187944000  7 -4.102070000 4.958536000 0.622090000  6 -4.895664000 5.893474000 0.123508000  1 -4.987127000 6.909723000 0.487435000  6 -8.006988000 6.163643000 -0.842428000  1 -7.924068000 5.462197000 -0.005469000  8 -9.085613000 5.826019000 -1.700674000  1 -9.869461000 5.912092000 -1.121151000  6 -8.106058000 7.626027000 -0.346225000  1 -7.535286000 7.735449000 0.586477000  8 -9.452402000 8.031443000 -0.163713000  15 -9.916230000 8.635097000 1.342206000  8 -10.100154000 10.127178000 1.193351000  8 -9.040253000 8.035224000 2.425506000  8 -11.453937000 7.981957000 1.402710000  6 -11.684291000 6.791587000 2.167979000  1 -12.669116000 6.893096000 2.648091000  1 -10.922328000 6.692683000 2.951705000  6 -11.725137000 5.533304000 1.315578000  1 -12.486534000 5.629766000 0.528121000  8 -10.442406000 5.309083000 0.676977000  6 -10.029332000 3.956489000 0.811307000  1 -9.718626000 3.566235000 -0.165276000  7 -8.842050000 3.908750000 1.684326000  6 -7.874753000 2.923436000 1.626691000  7 -7.895130000 1.847821000 0.801379000  6 -6.795578000 1.113834000 0.897639000  7 -6.662111000 0.003773000 0.080270000  1 -7.323319000 0.043873000 -0.700080000  1 -5.713381000 -0.162213000 -0.250340000  7 -5.787361000 1.363351000 1.793302000  1 -4.980193000 0.724298000 1.839913000  6 -5.673852000 2.528364000 2.615718000  8 -4.647845000 2.705517000 3.286501000  6 -6.854648000 3.342314000 2.498206000  7 -7.171967000 4.575447000 3.056957000  6 -8.357499000 4.889627000 2.554458000  1 -8.867854000 5.839471000 2.685219000  6 -11.231170000 3.174642000 1.354754000  1 -10.929646000 2.321354000 1.973723000  8 -12.013658000 2.748048000 0.240287000  1 -12.936325000 2.924380000 0.507645000  6 -11.995171000 4.262281000 2.139969000  1 -11.590869000 4.361873000 3.156404000  8 -13.396362000 4.001945000 2.177264000  15 -14.039382000 3.388354000 3.626732000  8 -13.429767000 4.135732000 4.785826000  8 -15.517527000 3.269273000 3.364366000  8 -13.319357000 1.886463000 3.656042000  6 -13.761777000 0.882353000 2.732869000  1 -13.692548000 1.243348000 1.697911000  1 -14.805302000 0.602485000 2.936985000  6 -12.890322000 -0.343976000 2.886584000  1 -13.276009000 -1.108775000 2.202040000  8 -11.518723000 -0.045586000 2.523335000  6 -10.622704000 -0.710976000 3.403775000  1 -9.868096000 -1.254878000 2.828035000  7 -9.880841000 0.303098000 4.178360000  6 -8.547164000 0.197319000 4.514339000  7 -7.769903000 -0.899056000 4.332224000  6 -6.524858000 -0.715626000 4.735977000  7 -5.621361000 -1.754926000 4.560654000  1 -6.070406000 -2.663360000 4.647560000  1 -4.765840000 -1.705801000 5.105156000  7 -6.065235000 0.462152000 5.268265000  1 -5.092630000 0.547978000 5.547105000  6 -6.852151000 1.633966000 5.531787000  8 -6.329775000 2.608771000 6.084467000  6 -8.202107000 1.431324000 5.090680000  7 -9.309674000 2.272649000 5.132387000  6 -10.291825000 1.572577000 4.580548000  1 -11.310766000 1.906487000 4.421273000  6 -11.465749000 -1.665867000 4.283403000  1 -11.018266000 -1.791822000 5.273797000  8 -11.562331000 -2.953124000 3.684260000  1 -12.413774000 -3.047109000 3.207782000  6 -12.804779000 -0.911282000 4.311364000  1 -12.761388000 -0.094401000 5.041018000  8 -13.931219000 -1.743798000 4.558533000  15 -14.804530000 -1.574102000 5.973708000  8 -14.678252000 -0.176276000 6.519109000  8 -16.137202000 -2.241979000 5.685649000  8 -13.919697000 -2.536781000 7.004676000  6 -13.669082000 -3.904417000 6.649345000  1 -13.441319000 -3.988253000 5.578192000  1 -14.552737000 -4.522564000 6.872524000  6 -12.476509000 -4.408359000 7.446626000  1 -12.399957000 -5.500584000 7.319207000  8 -11.269664000 -3.790438000 6.934094000  6 -10.404223000 -3.418846000 7.994622000  1 -9.392642000 -3.807694000 7.824118000  7 -10.270324000 -1.957332000 8.027545000  6 -9.082068000 -1.275511000 8.241172000  7 -7.860091000 -1.792668000 8.466645000  6 -6.942173000 -0.816863000 8.615961000  1 -5.921084000 -1.152588000 8.811306000  7 -7.107611000 0.518539000 8.547599000  6 -8.348327000 1.005040000 8.286282000  7 -8.512596000 2.359634000 8.181299000  1 -9.307216000 2.626213000 7.604791000  1 -7.660202000 2.830901000 7.881454000  6 -9.419192000 0.090229000 8.148404000  7 -10.776134000 0.248134000 7.879525000  6 -11.250338000 -0.988803000 7.819280000  1 -12.274918000 -1.268204000 7.600556000  6 -10.992917000 -4.013404000 9.287245000  1 -10.772195000 -3.380707000 10.161303000  8 -10.465578000 -5.326303000 9.440966000  1 -11.212210000 -5.854761000 9.781413000  1 -12.967286000 -3.103893000 9.122772000  6 -12.493581000 -4.081236000 8.956855000  8 -13.083616000 -5.088343000 9.774883000  1 -13.966085000 -5.289567000 9.427905000  19 8.853222000 9.276696000 -1.421608000 | 1 -17.004299000 -1.409277000 4.328818000  8 -17.392660000 -0.848256000 3.606730000  6 -17.272999000 -1.539941000 2.371287000  1 -17.210526000 -0.781114000 1.574903000  1 -18.161583000 -2.166774000 2.154831000  6 -16.027470000 -2.406188000 2.268203000  1 -15.229632000 -1.949731000 2.860771000  8 -16.276871000 -3.743237000 2.807691000  6 -15.428768000 -4.673906000 2.168826000  1 -14.756827000 -5.162733000 2.884083000  7 -16.261687000 -5.758939000 1.564241000  6 -17.598685000 -5.589406000 1.302646000  1 -17.996796000 -4.632212000 1.624288000  6 -15.583929000 -6.938411000 1.228032000  8 -14.379539000 -7.095598000 1.432722000  7 -16.391486000 -7.901414000 0.644015000  1 -15.919267000 -8.764422000 0.390836000  6 -17.770395000 -7.819680000 0.330372000  8 -18.339832000 -8.774150000 -0.209532000  6 -18.360597000 -6.551548000 0.710905000  1 -19.416197000 -6.387916000 0.522872000  6 -14.626886000 -3.923239000 1.090483000  1 -14.492031000 -4.514370000 0.176864000  8 -13.372766000 -3.567505000 1.652315000  1 -12.916559000 -2.983030000 0.999808000  6 -15.500785000 -2.670703000 0.859532000  1 -16.327250000 -2.913842000 0.172540000  8 -14.752984000 -1.550614000 0.412715000  15 -14.687905000 -1.309473000 -1.263464000  8 -15.850024000 -0.423676000 -1.651286000  8 -14.433341000 -2.641387000 -1.935415000  8 -13.324818000 -0.351211000 -1.256459000  6 -12.076456000 -0.885078000 -1.727214000  1 -11.606022000 -0.110633000 -2.350457000  1 -12.250210000 -1.781429000 -2.335903000  6 -11.102985000 -1.218689000 -0.612469000  1 -11.004466000 -0.386553000 0.098415000  8 -11.557151000 -2.405607000 0.095410000  6 -10.437120000 -3.166446000 0.587441000  1 -10.422035000 -3.123065000 1.682115000  7 -10.593622000 -4.569901000 0.241270000  6 -10.369710000 -5.159610000 -0.995011000  7 -10.197218000 -4.502239000 -2.157281000  6 -9.790941000 -5.286086000 -3.157174000  7 -9.519125000 -4.735038000 -4.366939000  1 -9.357969000 -3.720765000 -4.339217000  1 -8.912110000 -5.307283000 -4.966904000  7 -9.638538000 -6.649912000 -3.012949000  1 -9.130286000 -7.126555000 -3.770408000  6 -9.877800000 -7.401670000 -1.822483000  8 -9.671748000 -8.623492000 -1.811278000  6 -10.308249000 -6.539388000 -0.748896000  7 -10.517349000 -6.806681000 0.607675000  6 -10.679107000 -5.619631000 1.160473000  1 -10.870999000 -5.414147000 2.207908000  6 -9.165107000 -2.508599000 0.006311000  1 -8.461097000 -3.267770000 -0.355540000  8 -8.591140000 -1.747509000 1.063648000  1 -7.821856000 -1.235207000 0.711861000  6 -9.749725000 -1.684756000 -1.159177000  1 -9.958340000 -2.404602000 -1.956754000  8 -8.942984000 -0.625399000 -1.671255000  15 -8.094381000 -0.967238000 -3.107880000  8 -8.948518000 -1.835601000 -4.011888000  8 -7.537349000 0.365377000 -3.531005000  8 -6.835870000 -1.852727000 -2.478621000  6 -6.886465000 -3.293413000 -2.541409000  1 -7.915071000 -3.641804000 -2.655353000  1 -6.491709000 -3.661761000 -1.590259000  6 -6.056187000 -3.805953000 -3.711077000  1 -6.485189000 -3.457121000 -4.653227000  8 -4.709283000 -3.260224000 -3.679253000  6 -3.837586000 -4.144350000 -2.994949000  1 -2.934866000 -4.276202000 -3.601591000  7 -3.355288000 -3.485762000 -1.753111000  6 -3.998432000 -3.244752000 -0.542965000  7 -5.064751000 -3.901380000 -0.022988000  6 -5.519693000 -3.372443000 1.108811000  7 -6.599981000 -3.923720000 1.757379000  1 -7.021144000 -4.675492000 1.220416000  1 -7.299202000 -3.240033000 2.057033000  7 -4.947354000 -2.277298000 1.712393000  1 -5.276797000 -1.978640000 2.644638000  6 -3.849423000 -1.551423000 1.209955000  8 -3.487833000 -0.501329000 1.795113000  6 -3.330099000 -2.135584000 0.017463000  7 -2.298573000 -1.701630000 -0.806772000  6 -2.351057000 -2.513159000 -1.843833000  1 -1.734166000 -2.465690000 -2.732147000  6 -4.579429000 -5.504552000 -2.816617000  1 -4.891169000 -5.617322000 -1.772337000  8 -3.791932000 -6.625165000 -3.135717000  1 -3.548209000 -6.579231000 -4.088425000  6 -5.857005000 -5.322782000 -3.697046000  1 -6.718883000 -5.860284000 -3.285178000  8 -5.574430000 -5.781501000 -5.027908000  15 -6.522337000 -6.930949000 -5.769186000  8 -5.800540000 -8.249512000 -5.888651000  8 -7.926153000 -6.889923000 -5.170083000  8 -6.615216000 -6.162935000 -7.242926000  6 -6.220135000 -6.849326000 -8.437946000  1 -7.070666000 -6.835677000 -9.134129000  1 -5.959821000 -7.891505000 -8.211846000  6 -5.016252000 -6.200297000 -9.093691000  1 -4.727244000 -6.821982000 -9.956920000  8 -5.343886000 -4.864757000 -9.577936000  6 -4.240423000 -3.997834000 -9.376138000  1 -4.031032000 -3.430389000 -10.291456000  7 -4.565054000 -3.038848000 -8.297881000  6 -3.723275000 -2.037685000 -7.836063000  7 -2.561529000 -1.613662000 -8.375622000  6 -2.009535000 -0.642011000 -7.628162000  1 -1.099380000 -0.185108000 -8.022150000  7 -2.419228000 -0.167889000 -6.434850000  6 -3.542075000 -0.688096000 -5.865868000  7 -3.844489000 -0.349755000 -4.582627000  1 -4.467530000 -1.004771000 -4.114103000  1 -3.018391000 -0.101218000 -4.035796000  6 -4.325320000 -1.571962000 -6.647961000  7 -5.525368000 -2.231590000 -6.414637000  6 -5.628359000 -3.103723000 -7.406248000  1 -6.413135000 -3.837533000 -7.532374000  6 -3.051222000 -4.869766000 -8.945246000  1 -2.342259000 -4.322189000 -8.313644000  8 -2.415911000 -5.382832000 -10.113723000  1 -2.187118000 -6.302904000 -9.887537000  6 -3.790316000 -5.995744000 -8.196819000  1 -4.087467000 -5.666017000 -7.194883000  8 -3.033945000 -7.205917000 -8.126087000  15 -1.859866000 -7.307665000 -6.918466000  8 -1.316033000 -8.709348000 -7.005083000  8 -2.373318000 -6.681126000 -5.636132000  8 -0.769569000 -6.191707000 -7.481948000  6 0.248187000 -6.536191000 -8.430384000  1 0.122476000 -5.881831000 -9.303287000  1 0.156031000 -7.584302000 -8.747700000  6 1.622548000 -6.306916000 -7.810804000  1 2.388411000 -6.507691000 -8.573861000  8 1.752441000 -4.922267000 -7.396495000  6 1.764726000 -4.835364000 -5.970672000  1 2.752604000 -4.505334000 -5.625364000  7 0.819075000 -3.819100000 -5.519924000  6 1.147462000 -2.535656000 -5.112538000  7 2.370967000 -1.967979000 -5.136228000  6 2.390138000 -0.745354000 -4.607183000  7 3.538406000 -0.020749000 -4.629139000  1 4.381358000 -0.558223000 -4.785480000  1 3.613833000 0.793917000 -4.020634000  7 1.267612000 -0.130692000 -4.070648000  1 1.398753000 0.739989000 -3.553763000  6 -0.041308000 -0.674644000 -4.085259000  8 -0.999543000 -0.043513000 -3.579420000  6 -0.065298000 -1.960817000 -4.690277000  7 -1.118602000 -2.855218000 -4.858882000  6 -0.565076000 -3.946646000 -5.353981000  1 -1.097806000 -4.854964000 -5.611400000  6 1.444620000 -6.244225000 -5.410173000  1 0.353440000 -6.355512000 -5.344661000  8 2.030394000 -6.450892000 -4.146138000  1 2.475733000 -7.352818000 -4.154166000  6 1.915891000 -7.153264000 -6.565501000  1 1.367521000 -8.104050000 -6.589921000  8 3.337463000 -7.366344000 -6.501711000  15 3.804699000 -8.791412000 -5.759473000  8 3.190092000 -8.805986000 -4.357397000  8 3.596995000 -9.957745000 -6.692037000  8 5.420912000 -8.468017000 -5.693364000  6 6.015822000 -7.984034000 -4.475619000  1 6.844349000 -8.665781000 -4.232068000  1 5.292010000 -7.995235000 -3.653090000  6 6.611380000 -6.595250000 -4.615486000  1 7.137393000 -6.501151000 -5.580094000  8 5.601132000 -5.572789000 -4.514888000  6 6.204669000 -4.387377000 -4.024442000  1 6.542929000 -3.728368000 -4.841341000  7 5.163616000 -3.670383000 -3.275443000  6 5.289450000 -2.395483000 -2.753722000  7 6.350337000 -1.568964000 -2.916403000  6 6.193488000 -0.391021000 -2.313842000  7 7.201525000 0.515539000 -2.342730000  1 7.975244000 0.269149000 -2.945797000  1 7.020518000 1.514081000 -2.229108000  7 5.051447000 -0.059009000 -1.609710000  1 5.021621000 0.825884000 -1.114491000  6 3.887717000 -0.879961000 -1.440758000  8 2.918498000 -0.456209000 -0.800378000  6 4.073938000 -2.144620000 -2.091288000  7 3.210660000 -3.227143000 -2.228623000  6 3.887941000 -4.119405000 -2.940306000  1 3.518085000 -5.082920000 -3.270875000  6 7.447666000 -4.787312000 -3.176035000  1 7.225880000 -4.651852000 -2.106295000  8 8.560173000 -4.011295000 -3.580528000  1 9.328387000 -4.436113000 -3.137159000  6 7.617365000 -6.314485000 -3.489280000  1 7.371282000 -6.898087000 -2.593767000  8 8.908093000 -6.662651000 -3.985479000  15 10.105266000 -7.180777000 -2.945971000  8 11.321991000 -7.407231000 -3.812720000  8 9.596042000 -8.238204000 -1.992270000  8 10.221523000 -5.706605000 -2.135335000  6 11.177690000 -5.608895000 -1.063524000  1 11.249546000 -6.558045000 -0.521054000  1 10.803546000 -4.850208000 -0.369541000  6 12.555252000 -5.231923000 -1.593050000  1 12.898756000 -5.994468000 -2.297057000  8 12.472461000 -3.976446000 -2.343372000  6 12.979924000 -2.941861000 -1.543451000  1 13.362545000 -2.157751000 -2.206548000  7 11.883734000 -2.317318000 -0.730932000  6 11.902792000 -1.796839000 0.562425000  7 12.798182000 -2.004940000 1.551068000  6 12.490318000 -1.316530000 2.657508000  1 13.153532000 -1.464803000 3.504106000  7 11.473145000 -0.454258000 2.861377000  6 10.597785000 -0.224020000 1.840683000  7 9.693433000 0.763112000 1.966781000  1 8.870217000 0.843756000 1.365630000  1 9.624526000 1.264667000 2.840781000  6 10.753912000 -0.989667000 0.656061000  7 10.030324000 -1.026449000 -0.529607000  6 10.728498000 -1.825634000 -1.324330000  1 10.445718000 -2.114668000 -2.329339000  6 14.107019000 -3.554746000 -0.703286000  1 14.284272000 -3.023963000 0.238455000  8 15.268132000 -3.554619000 -1.530115000  1 15.636849000 -4.453154000 -1.413461000  6 13.593732000 -5.003880000 -0.466144000  1 13.127887000 -5.098864000 0.523432000  8 14.742628000 -5.854813000 -0.563892000  15 14.681693000 -7.452209000 -0.033108000  8 16.064387000 -7.985686000 -0.316928000  8 13.415055000 -8.112675000 -0.519749000  8 14.454643000 -7.227198000 1.601534000  6 15.408567000 -6.473895000 2.357178000  1 15.424698000 -5.421687000 2.030846000  1 16.421074000 -6.889433000 2.241811000  6 14.950728000 -6.563470000 3.810245000  1 14.913019000 -7.617977000 4.120033000  8 15.897914000 -5.867453000 4.667385000  6 15.212100000 -4.962753000 5.521590000  1 15.662714000 -5.019934000 6.520499000  7 15.394877000 -3.579539000 5.003041000  6 14.723697000 -2.407670000 5.345702000  7 13.580613000 -2.297190000 6.071974000  6 13.130486000 -1.040028000 6.156375000  7 11.979264000 -0.748537000 6.806384000  1 11.301315000 -1.490494000 7.038250000  1 11.544586000 0.141957000 6.590144000  7 13.809995000 0.035409000 5.617047000  1 13.381668000 0.963329000 5.729536000  6 14.997419000 -0.018047000 4.838649000  8 15.483014000 1.021960000 4.370813000  6 15.450411000 -1.378737000 4.709905000  7 16.535312000 -1.889948000 4.006633000  6 16.476333000 -3.193430000 4.210664000  1 17.160550000 -3.935275000 3.820383000  6 13.743774000 -5.381363000 5.520857000  1 13.079172000 -4.545412000 5.755237000  8 13.586100000 -6.451520000 6.448792000  1 12.917114000 -7.025272000 6.027621000  6 13.585007000 -5.913102000 4.081236000  1 13.409186000 -5.090098000 3.376088000  8 12.538162000 -6.880365000 4.013374000  15 11.051647000 -6.334612000 3.428681000  8 11.264546000 -5.673638000 2.081520000  8 10.096550000 -7.476603000 3.648983000  8 10.704786000 -5.167559000 4.562082000  6 10.908323000 -3.773561000 4.267506000  1 11.390947000 -3.652400000 3.292573000  1 11.558979000 -3.356110000 5.046398000  6 9.598413000 -3.006420000 4.280528000  1 9.831449000 -1.953608000 4.474854000  8 8.947374000 -3.076051000 2.970268000  6 7.638811000 -3.592774000 3.115514000  1 6.879395000 -2.797338000 3.163406000  7 7.322301000 -4.414007000 1.953161000  6 6.076616000 -4.545392000 1.369837000  7 4.998699000 -3.747433000 1.588377000  6 3.942839000 -4.051513000 0.826985000  7 2.813199000 -3.297908000 0.895331000  1 2.907420000 -2.438126000 1.449638000  1 2.327961000 -3.176235000 0.007762000  7 3.944295000 -5.136187000 -0.017785000  1 3.122558000 -5.287779000 -0.594209000  6 5.070343000 -5.950069000 -0.360370000  8 4.958731000 -6.814113000 -1.238252000  6 6.207088000 -5.590534000 0.441226000  7 7.505164000 -6.087475000 0.446375000  6 8.146530000 -5.372737000 1.357037000  1 9.199819000 -5.453539000 1.616572000  6 7.645872000 -4.400044000 4.438049000  1 8.115921000 -5.373124000 4.249275000  8 6.364824000 -4.625819000 4.981235000  1 6.102185000 -3.780026000 5.389567000  6 8.587848000 -3.549177000 5.302852000  1 9.077255000 -4.121715000 6.094180000  8 7.770150000 -2.515074000 5.873912000  15 8.434110000 -1.570964000 7.080618000  8 7.307182000 -1.224636000 8.025889000  8 9.709647000 -2.221096000 7.589575000  8 8.933555000 -0.226644000 6.239395000  6 8.289425000 0.280750000 5.061485000  1 8.275551000 -0.477175000 4.267424000  1 8.933597000 1.107058000 4.743442000  6 6.891883000 0.859434000 5.214932000  1 6.744181000 1.527786000 4.357182000  8 5.890049000 -0.180121000 5.171231000  6 4.749781000 0.311206000 5.874728000  1 4.130733000 0.967804000 5.250300000  7 3.929073000 -0.853809000 6.212784000  6 4.537609000 -1.963531000 6.760145000  1 5.582701000 -1.820242000 7.038394000  6 2.562119000 -0.839015000 5.887257000  8 1.961254000 0.158004000 5.495562000  7 1.939106000 -2.066875000 6.069934000  1 0.951602000 -2.083353000 5.833434000  6 2.482616000 -3.268159000 6.580918000  8 1.773489000 -4.275333000 6.683161000  6 3.878281000 -3.140053000 6.952924000  1 4.384670000 -3.993612000 7.391902000  6 5.326944000 1.093606000 7.079442000  1 5.583535000 0.384803000 7.873828000  8 4.457540000 2.081485000 7.596346000  1 4.702805000 2.892787000 7.104256000  6 6.636512000 1.687720000 6.487239000  1 7.464346000 1.633260000 7.197280000  8 6.395467000 3.051267000 6.118097000  15 7.216861000 4.297712000 6.906683000  8 8.225007000 3.703843000 7.864378000  8 6.170059000 5.305452000 7.309464000  8 7.995758000 4.972067000 5.592336000  6 9.188142000 4.362904000 5.078661000  1 9.335329000 3.352498000 5.479933000  1 10.057486000 4.968876000 5.378344000  6 9.135877000 4.323469000 3.554298000  1 10.150745000 4.165624000 3.163337000  8 8.293874000 3.216261000 3.118772000  6 7.324009000 3.686506000 2.172429000  1 7.696026000 3.575854000 1.142599000  7 6.120843000 2.878777000 2.298651000  6 5.975419000 1.561103000 1.870375000  7 6.878021000 0.830413000 1.166172000  6 6.516907000 -0.455835000 1.038722000  7 7.330775000 -1.338101000 0.424661000  1 8.183091000 -1.029055000 -0.059523000  1 6.974099000 -2.248410000 0.172825000  7 5.339459000 -0.955447000 1.539286000  1 5.201037000 -1.989364000 1.546516000  6 4.328624000 -0.220409000 2.192174000  8 3.286556000 -0.795676000 2.556105000  6 4.718590000 1.152633000 2.353102000  7 4.104453000 2.180166000 3.060902000  6 4.962139000 3.179184000 3.024633000  1 4.810170000 4.143865000 3.485805000  6 7.118240000 5.173540000 2.542166000  1 6.529176000 5.205726000 3.466989000  8 6.473527000 5.953503000 1.564307000  1 7.140450000 6.456008000 1.038668000  6 8.539346000 5.604884000 2.950565000  1 8.513623000 6.420413000 3.678469000  8 9.292897000 6.024697000 1.798244000  15 9.286170000 7.677869000 1.493712000  8 8.199077000 7.920572000 0.446562000  8 9.300142000 8.453007000 2.786221000  8 10.719409000 7.792418000 0.681064000  6 11.876035000 7.111929000 1.200829000  1 11.566943000 6.354504000 1.929007000  1 12.543587000 7.830742000 1.694559000  6 12.588482000 6.445514000 0.014393000  1 13.028531000 7.207182000 -0.638771000  8 13.686403000 5.612147000 0.489678000  6 13.290922000 4.262473000 0.542656000  1 14.052049000 3.629300000 0.075298000  7 13.170700000 3.816848000 1.975367000  6 13.248530000 4.671839000 3.040616000  1 13.583005000 5.674326000 2.796189000  6 12.898705000 2.450818000 2.153575000  8 12.980458000 1.646309000 1.228637000  7 12.504431000 2.110209000 3.436484000  1 12.165099000 1.130090000 3.523415000  6 12.530675000 2.923578000 4.569410000  8 12.227763000 2.448817000 5.687845000  6 12.948556000 4.281711000 4.315805000  1 13.037294000 4.972511000 5.146696000  6 11.907986000 4.115206000 -0.186025000  1 11.134967000 3.896656000 0.562570000  8 11.870105000 3.114107000 -1.174383000  1 12.037566000 3.599134000 -2.008907000  6 11.668144000 5.529533000 -0.783364000  1 10.631770000 5.851983000 -0.681140000  8 12.049969000 5.506575000 -2.168996000  15 10.874187000 5.950957000 -3.303816000  8 11.608022000 6.162925000 -4.595904000  8 10.002229000 7.001170000 -2.650851000  8 10.029385000 4.530427000 -3.459578000  6 8.998533000 4.178983000 -2.523440000  1 8.954534000 4.878871000 -1.679714000  1 9.228144000 3.178313000 -2.134642000  6 7.650862000 4.169431000 -3.239007000  1 7.752197000 3.697814000 -4.229720000  8 6.735959000 3.403793000 -2.412309000  6 5.401794000 3.689661000 -2.860397000  1 5.071228000 2.965207000 -3.610856000  7 4.514822000 3.558164000 -1.710621000  6 4.730576000 4.369518000 -0.608442000  1 5.695864000 4.864121000 -0.572927000  6 3.322063000 2.837452000 -1.855704000  8 3.070456000 2.174336000 -2.873877000  7 2.464045000 2.909603000 -0.788893000  1 1.504735000 2.582138000 -1.038862000  6 2.536527000 3.828374000 0.271492000  8 1.568443000 3.984914000 1.029621000  6 3.806482000 4.520904000 0.369599000  1 4.002033000 5.179515000 1.205906000  6 5.428798000 5.102498000 -3.495927000  1 4.786155000 5.792095000 -2.928442000  8 4.992114000 4.972847000 -4.833166000  1 4.977288000 5.879935000 -5.202236000  6 6.924511000 5.515930000 -3.364362000  1 7.062240000 6.076939000 -2.430633000  8 7.401369000 6.271145000 -4.468984000  15 7.060755000 7.902935000 -4.512249000  8 6.880757000 8.447601000 -3.103217000  8 8.018196000 8.508967000 -5.502391000  8 5.532826000 7.819546000 -5.221512000  6 4.460208000 8.567256000 -4.621816000  1 4.736869000 9.620887000 -4.474301000  1 4.222545000 8.146913000 -3.634176000  6 3.259085000 8.505315000 -5.548759000  1 3.472075000 9.055940000 -6.477099000  8 2.978786000 7.123908000 -5.889993000  6 1.583437000 6.856532000 -5.777398000  1 1.232772000 6.298702000 -6.653263000  7 1.348215000 5.989366000 -4.615421000  6 0.553809000 4.852939000 -4.591844000  7 -0.201206000 4.390264000 -5.610296000  6 -0.810905000 3.234282000 -5.315986000  7 -1.606579000 2.632196000 -6.230131000  1 -1.539676000 3.003046000 -7.168934000  1 -1.862761000 1.632830000 -6.144530000  7 -0.699326000 2.621264000 -4.088123000  1 -1.049067000 1.655964000 -3.943162000  6 0.057313000 3.086453000 -2.999974000  8 0.070889000 2.394855000 -1.951318000  6 0.733891000 4.304866000 -3.305896000  7 1.612318000 5.085171000 -2.560200000  6 1.958056000 6.068833000 -3.365453000  1 2.641482000 6.866308000 -3.114097000  6 0.885501000 8.213472000 -5.646967000  1 -0.058542000 8.146263000 -5.096811000  8 0.680253000 8.745460000 -6.954794000  1 0.844278000 9.702172000 -6.854676000  6 1.958915000 9.041236000 -4.916772000  1 1.937014000 8.840289000 -3.837989000  8 1.799241000 10.437386000 -5.149545000  15 1.152236000 11.346087000 -3.862409000  8 1.165860000 12.766459000 -4.363406000  8 1.789214000 10.882869000 -2.576298000  8 -0.412326000 10.753645000 -3.814444000  6 -1.312185000 11.171564000 -4.851963000  1 -0.839201000 11.058660000 -5.838370000  1 -1.584905000 12.229030000 -4.721025000  6 -2.575787000 10.333355000 -4.816917000  1 -3.257733000 10.714402000 -5.596067000  8 -2.239601000 8.954321000 -5.105915000  6 -3.117691000 8.073886000 -4.409258000  1 -3.627014000 7.400608000 -5.103237000  7 -2.357690000 7.201964000 -3.490359000  6 -1.360709000 7.711794000 -2.704784000  1 -1.018463000 8.713719000 -2.954064000  6 -2.853940000 5.866902000 -3.287969000  8 -3.764985000 5.452770000 -4.026468000  7 -2.292040000 5.125661000 -2.294203000  6 -1.344006000 5.646309000 -1.503985000  7 -0.892977000 4.880583000 -0.482126000  1 0.023441000 5.056830000 -0.084889000  1 -1.154172000 3.900483000 -0.507791000  6 -0.821069000 6.971414000 -1.693996000  1 -0.012606000 7.361739000 -1.084639000  6 -4.120351000 8.942708000 -3.638618000  1 -4.364348000 8.495196000 -2.670178000  8 -5.287263000 9.103517000 -4.434137000  1 -5.877941000 9.627586000 -3.854840000  6 -3.349934000 10.276001000 -3.493441000  1 -2.659741000 10.225788000 -2.642468000  8 -4.197015000 11.417740000 -3.362917000  15 -4.994886000 11.634275000 -1.906334000  8 -4.139812000 11.160070000 -0.752718000  8 -5.583458000 13.021599000 -1.978159000  8 -6.233689000 10.501975000 -2.170204000  6 -6.741347000 9.777557000 -1.030909000  1 -7.562069000 10.343681000 -0.561664000  1 -5.938813000 9.635382000 -0.294903000  6 -7.289544000 8.433721000 -1.459496000  1 -7.979553000 8.552636000 -2.311541000  8 -6.202881000 7.554516000 -1.826074000  6 -6.615780000 6.199015000 -1.663007000  1 -6.767915000 5.699524000 -2.628964000  7 -5.550227000 5.474674000 -0.982779000  6 -5.225553000 4.144857000 -1.193202000  7 -5.721057000 3.304859000 -2.119041000  6 -5.146299000 2.089790000 -2.040489000  1 -5.516435000 1.344314000 -2.744803000  7 -4.200359000 1.662472000 -1.171498000  6 -3.738191000 2.531066000 -0.235513000  7 -2.761025000 2.114284000 0.627671000  1 -2.739759000 2.629903000 1.502626000  1 -2.715746000 1.101917000 0.762281000  6 -4.264669000 3.842873000 -0.208416000  7 -4.019568000 4.947083000 0.601862000  6 -4.803963000 5.894502000 0.112208000  1 -4.877411000 6.911318000 0.478518000  6 -7.922640000 6.208148000 -0.847272000  1 -7.851341000 5.497249000 -0.017350000  8 -9.002623000 5.893119000 -1.712086000  1 -9.786663000 5.976075000 -1.132454000  6 -8.003618000 7.666579000 -0.335103000  1 -7.437665000 7.756922000 0.602535000  8 -9.345066000 8.089201000 -0.156414000  15 -9.812197000 8.683857000 1.351725000  8 -9.984025000 10.178416000 1.214289000  8 -8.946498000 8.068685000 2.434753000  8 -11.354896000 8.041106000 1.398611000  6 -11.598406000 6.848261000 2.155725000  1 -12.582542000 6.956224000 2.635915000  1 -10.838250000 6.736072000 2.939421000  6 -11.651014000 5.596030000 1.294883000  1 -12.410045000 5.705215000 0.506863000  8 -10.369879000 5.362276000 0.656482000  6 -9.967446000 4.006327000 0.788766000  1 -9.658429000 3.615531000 -0.188185000  7 -8.781555000 3.948100000 1.663351000  6 -7.823985000 2.953286000 1.607224000  7 -7.858045000 1.873933000 0.787407000  6 -6.766102000 1.128826000 0.884591000  7 -6.649373000 0.011774000 0.073917000  1 -7.310401000 0.056926000 -0.705955000  1 -5.703979000 -0.170909000 -0.257061000  7 -5.752597000 1.372494000 1.775517000  1 -4.950805000 0.726430000 1.823520000  6 -5.626483000 2.538111000 2.595644000  8 -4.599114000 2.704397000 3.266804000  6 -6.798133000 3.364865000 2.475415000  7 -7.102330000 4.602976000 3.030623000  6 -8.285616000 4.927188000 2.529254000  1 -8.786497000 5.882318000 2.659052000  6 -11.176127000 3.231659000 1.328476000  1 -10.881224000 2.377045000 1.949044000  8 -11.958101000 2.808552000 0.212407000  1 -12.880167000 2.993033000 0.476656000  6 -11.936176000 4.323654000 2.112016000  1 -11.538112000 4.416270000 3.131488000  8 -13.339658000 4.074343000 2.138541000  15 -14.000796000 3.475214000 3.585723000  8 -13.391822000 4.221410000 4.745920000  8 -15.478034000 3.371259000 3.312234000  8 -13.297713000 1.965595000 3.627362000  6 -13.744138000 0.962190000 2.705451000  1 -13.663980000 1.317939000 1.669350000  1 -14.792015000 0.694049000 2.903318000  6 -12.886697000 -0.272551000 2.871257000  1 -13.277421000 -1.038162000 2.190323000  8 -11.510477000 0.009748000 2.513323000  6 -10.625345000 -0.657955000 3.403139000  1 -9.874108000 -1.214568000 2.835471000  7 -9.875314000 0.354670000 4.170963000  6 -8.541607000 0.241916000 4.504704000  7 -7.772180000 -0.861184000 4.329109000  6 -6.524653000 -0.682640000 4.727033000  7 -5.627715000 -1.728506000 4.554676000  1 -6.084164000 -2.633228000 4.642468000  1 -4.774602000 -1.685336000 5.103635000  7 -6.055619000 0.495625000 5.249748000  1 -5.080404000 0.579046000 5.520193000  6 -6.834911000 1.673017000 5.510210000  8 -6.305619000 2.646940000 6.057915000  6 -8.186852000 1.477268000 5.072049000  7 -9.288409000 2.326651000 5.110162000  6 -10.276488000 1.629745000 4.565001000  1 -11.292936000 1.970291000 4.403782000  6 -11.482169000 -1.597397000 4.286157000  1 -11.041581000 -1.718935000 5.280236000  8 -11.586357000 -2.888501000 3.697095000  1 -12.434809000 -2.978828000 3.214321000  6 -12.814159000 -0.829950000 4.300643000  1 -12.766467000 -0.007768000 5.024113000  8 -13.950289000 -1.648922000 4.548231000  15 -14.823286000 -1.465874000 5.961923000  8 -14.689077000 -0.065622000 6.499130000  8 -16.159789000 -2.127626000 5.677429000  8 -13.944304000 -2.427863000 6.998256000  6 -13.706657000 -3.800265000 6.652409000  1 -13.474533000 -3.892833000 5.582918000  1 -14.597825000 -4.407718000 6.875077000  6 -12.523277000 -4.310760000 7.459017000  1 -12.455667000 -5.404336000 7.338216000  8 -11.308071000 -3.706395000 6.950431000  6 -10.446510000 -3.334413000 8.014093000  1 -9.437374000 -3.733688000 7.853485000  7 -10.298136000 -1.874123000 8.036488000  6 -9.101987000 -1.203523000 8.241953000  7 -7.885360000 -1.732407000 8.468959000  6 -6.956106000 -0.765825000 8.607392000  1 -5.938265000 -1.111369000 8.802566000  7 -7.106748000 0.570729000 8.528441000  6 -8.342733000 1.069083000 8.266925000  7 -8.491987000 2.424620000 8.153114000  1 -9.286594000 2.696698000 7.579368000  1 -7.635814000 2.885032000 7.846926000  6 -9.424073000 0.165062000 8.138463000  7 -10.779828000 0.335616000 7.871015000  6 -11.267821000 -0.896495000 7.822071000  1 -12.295449000 -1.166224000 7.605444000  6 -11.048656000 -3.914881000 9.307054000  1 -10.829225000 -3.277493000 10.178038000  8 -10.531627000 -5.230410000 9.473791000  1 -11.284594000 -5.751527000 9.811618000  1 -13.015909000 -2.993468000 9.124537000  6 -12.547489000 -3.974743000 8.967253000  8 -13.149621000 -4.973347000 9.787005000  1 -14.032263000 -5.168447000 9.436885000  11 8.696959000 8.555724000 -1.703875000 |
| **H2** | 1 17.786305000 8.680898000 4.271280000  8 18.395698000 8.547403000 5.042780000  6 18.793139000 9.863270000 5.405195000  1 19.196184000 9.828913000 6.427167000  1 17.931188000 10.551692000 5.405936000  6 19.846669000 10.470676000 4.478678000  1 19.906645000 11.555917000 4.657748000  8 21.161280000 9.890519000 4.727170000  6 21.859247000 9.734326000 3.506404000  1 22.858828000 10.177528000 3.571384000  7 22.061987000 8.274528000 3.235831000  6 21.311768000 7.305796000 3.866236000  1 20.508409000 7.681474000 4.494680000  6 23.082617000 7.951010000 2.336312000  8 23.740981000 8.806253000 1.740084000  7 23.284391000 6.588489000 2.179614000  1 24.026155000 6.332295000 1.535043000  6 22.614015000 5.522146000 2.828721000  8 22.955993000 4.354630000 2.610633000  6 21.555281000 5.975827000 3.704481000  1 20.938885000 5.237651000 4.203655000  6 21.018956000 10.396821000 2.401686000  1 21.141464000 9.896310000 1.433795000  8 21.374568000 11.777600000 2.336216000  1 20.569858000 12.233778000 2.033183000  6 19.618361000 10.213713000 2.987438000  1 19.313665000 9.171780000 2.847392000  8 18.664561000 11.112103000 2.425691000  15 17.179623000 10.449535000 1.989114000  8 16.301618000 11.618816000 1.642099000  8 16.786450000 9.383825000 2.999069000  8 17.604826000 9.574222000 0.637139000  6 18.320940000 10.227265000 -0.419994000  1 18.960806000 11.022116000 -0.014516000  1 17.619358000 10.676404000 -1.139770000  6 19.174880000 9.215407000 -1.159887000  1 19.724092000 9.753727000 -1.949376000  8 20.129858000 8.603410000 -0.252660000  6 20.288887000 7.227530000 -0.567003000  1 21.354538000 6.977367000 -0.638367000  7 19.722246000 6.403002000 0.518494000  6 20.026134000 5.072960000 0.737808000  7 20.833990000 4.301278000 -0.031686000  6 20.932665000 3.057552000 0.418985000  7 21.706860000 2.141691000 -0.251922000  1 21.994647000 2.473724000 -1.166221000  1 21.335574000 1.196812000 -0.279506000  7 20.319075000 2.620717000 1.571307000  1 20.496604000 1.675129000 1.896054000  6 19.470876000 3.393064000 2.422819000  8 19.027047000 2.897778000 3.466420000  6 19.290134000 4.709388000 1.879207000  7 18.533064000 5.778263000 2.337809000  6 18.805004000 6.770685000 1.502473000  1 18.367162000 7.759663000 1.512356000  6 19.556579000 6.982424000 -1.890323000  1 19.195568000 5.953386000 -1.979373000  8 20.438454000 7.306738000 -2.966236000  1 19.877800000 7.792231000 -3.601021000  6 18.435540000 8.033808000 -1.805945000  1 17.611904000 7.690955000 -1.168677000  8 17.961323000 8.403368000 -3.099514000  15 16.510918000 7.694865000 -3.629212000  8 16.269118000 8.304757000 -4.984900000  8 15.519539000 7.699112000 -2.493696000  8 16.976024000 6.095749000 -3.780469000  6 17.855875000 5.725741000 -4.847973000  1 18.745509000 6.370931000 -4.856932000  1 17.347433000 5.822795000 -5.818622000  6 18.295353000 4.285099000 -4.674917000  1 18.937996000 4.020929000 -5.530484000  8 19.068328000 4.145983000 -3.445910000  6 18.764713000 2.910698000 -2.819511000  1 19.685977000 2.394792000 -2.527780000  7 17.987016000 3.133968000 -1.585975000  6 17.865150000 2.200061000 -0.576548000  7 18.539202000 1.023512000 -0.495752000  6 18.230252000 0.332517000 0.596107000  7 18.910946000 -0.826823000 0.873207000  1 19.359832000 -1.233769000 0.060404000  1 18.439873000 -1.512899000 1.453511000  7 17.311558000 0.762180000 1.523191000  1 17.065056000 0.158992000 2.301950000  6 16.537808000 1.966351000 1.463147000  8 15.696436000 2.198766000 2.337411000  6 16.908609000 2.731189000 0.306348000  7 16.443013000 3.957712000 -0.152910000  6 17.099923000 4.168293000 -1.284152000  1 16.995664000 5.022667000 -1.942532000  6 17.952048000 2.089595000 -3.838788000  1 17.283857000 1.388410000 -3.333578000  8 18.836925000 1.425339000 -4.725859000  1 18.599558000 0.477712000 -4.680356000  6 17.189604000 3.231622000 -4.543898000  1 16.403919000 3.599711000 -3.872878000  8 16.630482000 2.917681000 -5.809490000  15 15.129101000 2.154910000 -5.824381000  8 14.633234000 2.299600000 -7.241454000  8 14.333686000 2.542380000 -4.601377000  8 15.577596000 0.560670000 -5.542357000  6 16.451397000 -0.052587000 -6.493127000  1 17.293021000 0.617160000 -6.716628000  1 15.919599000 -0.278057000 -7.430746000  6 16.980028000 -1.354197000 -5.927760000  1 17.685986000 -1.786067000 -6.653398000  8 17.697365000 -1.094808000 -4.681726000  6 17.461939000 -2.136383000 -3.742363000  1 18.411699000 -2.491809000 -3.326352000  7 16.662055000 -1.632176000 -2.610721000  6 16.636443000 -2.231745000 -1.362693000  7 17.295893000 -3.337051000 -0.964701000  6 17.031039000 -3.623741000 0.324747000  1 17.523121000 -4.511501000 0.726835000  7 16.242910000 -2.956102000 1.193891000  6 15.586691000 -1.849475000 0.755170000  7 14.842108000 -1.150463000 1.653732000  1 14.155436000 -0.497515000 1.296701000  1 14.598597000 -1.633768000 2.509184000  6 15.765521000 -1.437273000 -0.587735000  7 15.258127000 -0.385203000 -1.339945000  6 15.798793000 -0.539942000 -2.542107000  1 15.604936000 0.072126000 -3.416616000  6 16.719523000 -3.242071000 -4.496992000  1 16.079380000 -3.828456000 -3.831650000  8 17.678342000 -4.078597000 -5.145104000  1 17.302595000 -4.237975000 -6.032210000  6 15.947373000 -2.426132000 -5.550663000  1 15.046783000 -1.965873000 -5.127214000  8 15.628090000 -3.221853000 -6.689262000  15 14.035316000 -3.809700000 -6.804963000  8 14.018532000 -4.590258000 -8.092369000  8 13.080235000 -2.698044000 -6.454457000  8 13.980793000 -4.843217000 -5.489301000  6 14.758468000 -6.044669000 -5.513725000  1 15.800978000 -5.824830000 -5.781282000  1 14.353783000 -6.754929000 -6.250398000  6 14.730679000 -6.703978000 -4.147562000  1 15.348938000 -7.614252000 -4.205623000  8 15.303197000 -5.817671000 -3.147009000  6 14.567593000 -5.900338000 -1.937795000  1 15.254157000 -6.017340000 -1.090534000  7 13.820867000 -4.642443000 -1.718695000  6 13.297105000 -4.235398000 -0.506809000  7 13.300882000 -4.951952000 0.645696000  6 12.712914000 -4.314211000 1.649464000  7 12.645431000 -4.900295000 2.888261000  1 12.892537000 -5.883465000 2.879796000  1 11.822823000 -4.695174000 3.445081000  7 12.201051000 -3.039846000 1.535291000  1 11.817379000 -2.582784000 2.357192000  6 12.200784000 -2.229796000 0.355282000  8 11.788867000 -1.063363000 0.415972000  6 12.747900000 -2.962508000 -0.749061000  7 12.903681000 -2.606383000 -2.082526000  6 13.538478000 -3.628240000 -2.635476000  1 13.796036000 -3.740005000 -3.680499000  6 13.619801000 -7.093752000 -2.074697000  1 12.720603000 -6.979753000 -1.463903000  8 14.329796000 -8.280551000 -1.713907000  1 14.079405000 -8.930499000 -2.397970000  6 13.351109000 -7.092447000 -3.591017000  1 12.592827000 -6.350037000 -3.865675000  8 12.987686000 -8.392774000 -4.055511000  15 11.344190000 -8.705837000 -4.345910000  8 11.322259000 -10.114077000 -4.879026000  8 10.730849000 -7.512605000 -5.033010000  8 10.720180000 -8.659987000 -2.792626000  6 11.091993000 -9.674870000 -1.855587000  1 12.178999000 -9.831121000 -1.866063000  1 10.602565000 -10.629798000 -2.100671000  6 10.673868000 -9.266697000 -0.454567000  1 10.998573000 -10.066474000 0.230648000  8 11.336503000 -8.031614000 -0.072007000  6 10.445655000 -7.194424000 0.643560000  1 10.937270000 -6.804868000 1.542038000  7 10.077716000 -6.025293000 -0.181064000  6 9.610676000 -4.826581000 0.321658000  7 9.334553000 -4.562832000 1.625208000  6 8.918142000 -3.316589000 1.817371000  7 8.654344000 -2.876963000 3.091345000  1 8.589125000 -3.635639000 3.761315000  1 7.903343000 -2.201041000 3.186572000  7 8.795714000 -2.397858000 0.800486000  1 8.553244000 -1.438821000 1.026407000  6 9.068798000 -2.621617000 -0.589862000  8 8.947314000 -1.696956000 -1.397684000  6 9.475103000 -3.983401000 -0.794988000  7 9.832213000 -4.650237000 -1.960145000  6 10.182797000 -5.864777000 -1.563282000  1 10.498405000 -6.685630000 -2.194603000  6 9.226515000 -8.049254000 0.993638000  1 8.319128000 -7.452841000 1.130176000  8 9.532904000 -8.790398000 2.176985000  1 9.224068000 -9.696038000 1.982287000  6 9.177244000 -9.006949000 -0.213278000  1 8.708050000 -8.530775000 -1.082460000  8 8.523790000 -10.233054000 0.112763000  15 6.856000000 -10.334878000 -0.207478000  8 6.472250000 -11.719692000 0.243820000  8 6.584749000 -9.771193000 -1.579215000  8 6.265419000 -9.184657000 0.846683000  6 6.235510000 -9.454764000 2.251982000  1 7.244209000 -9.675716000 2.623367000  1 5.581464000 -10.311807000 2.464311000  6 5.686591000 -8.244977000 2.980609000  1 5.694480000 -8.460209000 4.060585000  8 6.516214000 -7.074737000 2.730791000  6 5.703961000 -5.920676000 2.579086000  1 6.057250000 -5.109814000 3.228190000  7 5.819445000 -5.420323000 1.192167000  6 5.771241000 -4.097835000 0.780139000  7 5.578027000 -2.981540000 1.516046000  6 5.573909000 -1.862113000 0.756403000  1 5.425935000 -0.928066000 1.300637000  7 5.697402000 -1.758424000 -0.573640000  6 5.856704000 -2.897281000 -1.299829000  7 5.915968000 -2.807610000 -2.656156000  1 5.520572000 -3.602472000 -3.155911000  1 5.649603000 -1.901977000 -3.026953000  6 5.954307000 -4.134646000 -0.617560000  7 6.123793000 -5.442525000 -1.056779000  6 6.034322000 -6.179291000 0.040199000  1 6.128061000 -7.258279000 0.097768000  6 4.269485000 -6.331165000 2.942929000  1 3.525619000 -5.733107000 2.413706000  8 4.136806000 -6.188370000 4.362161000  1 3.376709000 -6.752622000 4.594582000  6 4.259273000 -7.826176000 2.589814000  1 4.089261000 -7.982848000 1.519819000  8 3.295477000 -8.521739000 3.373679000  15 2.390478000 -9.783574000 2.705095000  8 2.272968000 -10.808125000 3.806700000  8 2.889519000 -10.086425000 1.313726000  8 0.911361000 -9.033348000 2.472961000  6 0.210667000 -8.518229000 3.613558000  1 0.840581000 -8.548545000 4.512223000  1 -0.684236000 -9.130775000 3.804034000  6 -0.229795000 -7.087112000 3.354081000  1 -0.775340000 -6.716581000 4.238275000  8 0.947041000 -6.271996000 3.137337000  6 0.700145000 -5.300690000 2.130244000  1 0.963700000 -4.310359000 2.499748000  7 1.542972000 -5.551281000 0.955566000  6 1.963073000 -4.540336000 0.113688000  7 1.776641000 -3.219591000 0.326760000  6 2.254615000 -2.468427000 -0.664179000  7 2.123669000 -1.120493000 -0.596213000  1 1.810304000 -0.729401000 0.303055000  1 2.720931000 -0.550524000 -1.178876000  7 2.853387000 -3.000330000 -1.786430000  1 2.944930000 -2.384442000 -2.601110000  6 3.057052000 -4.381729000 -2.057242000  8 3.589934000 -4.725958000 -3.127072000  6 2.580890000 -5.188584000 -0.970661000  7 2.533197000 -6.572195000 -0.801928000  6 1.895266000 -6.756190000 0.344551000  1 1.684698000 -7.706534000 0.818854000  6 -0.782649000 -5.385486000 1.759120000  1 -0.941073000 -5.173721000 0.693576000  8 -1.521704000 -4.459283000 2.550354000  1 -2.444808000 -4.593488000 2.226643000  6 -1.101165000 -6.859324000 2.110102000  1 -0.765349000 -7.504084000 1.288087000  8 -2.472274000 -7.121556000 2.414056000  15 -3.579971000 -7.004559000 1.162515000  8 -4.905386000 -7.505544000 1.676564000  8 -2.951990000 -7.490404000 -0.123851000  8 -3.596694000 -5.305959000 1.157808000  6 -4.738414000 -4.547546000 0.768354000  1 -4.782954000 -3.683822000 1.445907000  1 -5.661899000 -5.132049000 0.880721000  6 -4.652295000 -4.063462000 -0.664471000  1 -5.524087000 -3.414658000 -0.857354000  8 -3.430005000 -3.332575000 -0.902434000  6 -3.385132000 -3.111618000 -2.331934000  1 -3.895464000 -2.172031000 -2.582930000  7 -2.004002000 -2.954636000 -2.722002000  6 -1.353219000 -1.743484000 -2.903964000  7 -1.903040000 -0.513267000 -2.773233000  6 -1.037844000 0.475320000 -2.996907000  7 -1.437613000 1.764791000 -2.828109000  1 -2.438756000 1.914309000 -2.829825000  1 -0.891559000 2.512087000 -3.237249000  7 0.276572000 0.248943000 -3.338863000  1 0.901305000 1.038648000 -3.474133000  6 0.906579000 -1.020959000 -3.472315000  8 2.119359000 -1.064216000 -3.748846000  6 -0.029777000 -2.080358000 -3.237329000  7 0.139520000 -3.462508000 -3.241407000  6 -1.041387000 -3.949730000 -2.918980000  1 -1.285968000 -4.999413000 -2.805936000  6 -4.173052000 -4.276742000 -3.001382000  1 -3.527612000 -4.869920000 -3.668337000  8 -5.247031000 -3.698407000 -3.713940000  1 -5.788140000 -4.473377000 -4.047251000  6 -4.625039000 -5.117774000 -1.767202000  1 -3.879204000 -5.890264000 -1.532905000  8 -5.927003000 -5.672524000 -1.841520000  15 -6.368158000 -6.747767000 -3.038311000  8 -5.549151000 -8.010647000 -2.952370000  8 -6.500696000 -5.973426000 -4.345606000  8 -7.912259000 -6.957842000 -2.470775000  6 -8.085827000 -7.756985000 -1.283911000  1 -7.130372000 -7.911117000 -0.766100000  1 -8.479960000 -8.740977000 -1.577292000  6 -9.080120000 -7.135379000 -0.320897000  1 -9.335174000 -7.909415000 0.421274000  8 -8.501274000 -6.000616000 0.385745000  6 -9.502971000 -5.029199000 0.641519000  1 -9.503886000 -4.746360000 1.699555000  7 -9.196662000 -3.783028000 -0.128520000  6 -8.323857000 -3.802918000 -1.182594000  1 -7.935816000 -4.778152000 -1.449409000  6 -9.764354000 -2.584714000 0.337717000  8 -10.563386000 -2.564864000 1.279305000  7 -9.343693000 -1.449386000 -0.324292000  1 -9.679082000 -0.545946000 0.101533000  6 -8.375649000 -1.386305000 -1.324163000  8 -7.933860000 -0.283048000 -1.723252000  6 -7.918405000 -2.663077000 -1.810727000  1 -7.178679000 -2.710881000 -2.603931000  6 -10.840870000 -5.637916000 0.210089000  1 -11.561655000 -4.880029000 -0.108809000  8 -11.357560000 -6.404394000 1.299827000  1 -11.720403000 -7.208533000 0.884602000  6 -10.379311000 -6.577406000 -0.916626000  1 -10.171355000 -6.018568000 -1.836345000  8 -11.310435000 -7.626834000 -1.166855000  15 -12.385322000 -7.406117000 -2.463854000  8 -13.128922000 -8.712945000 -2.560052000  8 -11.661004000 -6.746099000 -3.609567000  8 -13.380561000 -6.213206000 -1.839993000  6 -14.245415000 -6.572849000 -0.758060000  1 -13.681032000 -7.077334000 0.038012000  1 -15.036199000 -7.256091000 -1.102790000  6 -14.900510000 -5.333263000 -0.186663000  1 -15.571087000 -5.653753000 0.628056000  8 -13.902025000 -4.431736000 0.347304000  6 -14.277237000 -3.083849000 0.099561000  1 -14.242299000 -2.499243000 1.027344000  7 -13.324811000 -2.459172000 -0.823216000  6 -13.062695000 -1.102305000 -0.840043000  7 -13.619536000 -0.184656000 -0.021433000  6 -13.186431000 1.056528000 -0.279209000  7 -13.623029000 2.082389000 0.480663000  1 -14.376646000 1.870331000 1.118444000  1 -13.455127000 3.065252000 0.218202000  7 -12.277172000 1.350000000 -1.269321000  1 -12.053229000 2.355426000 -1.437027000  6 -11.683130000 0.417434000 -2.139082000  8 -10.876757000 0.792836000 -3.020241000  6 -12.133238000 -0.916327000 -1.877562000  7 -11.828307000 -2.132452000 -2.486048000  6 -12.560954000 -3.030347000 -1.844931000  1 -12.601596000 -4.094740000 -2.044805000  6 -15.700222000 -3.110719000 -0.478779000  1 -15.878341000 -2.271167000 -1.163147000  8 -16.627493000 -3.103176000 0.605749000  1 -17.331904000 -3.728871000 0.345288000  6 -15.718450000 -4.485504000 -1.171147000  1 -15.242766000 -4.445035000 -2.157959000  8 -17.038575000 -5.018164000 -1.262405000  15 -17.921766000 -4.754244000 -2.666582000  8 -19.193790000 -5.550161000 -2.493930000  8 -17.028479000 -4.874503000 -3.877532000  8 -18.254865000 -3.129416000 -2.372915000  6 -19.258719000 -2.548856000 -3.224264000  1 -19.992165000 -3.312805000 -3.518361000  1 -18.792162000 -2.132662000 -4.131022000  6 -19.976362000 -1.424078000 -2.495842000  1 -20.636948000 -0.921727000 -3.212872000  8 -20.805765000 -1.948396000 -1.412308000  6 -20.352314000 -1.473080000 -0.160082000  1 -21.049635000 -0.749746000 0.279396000  7 -20.295324000 -2.612317000 0.789560000  6 -19.874899000 -3.848287000 0.356519000  1 -19.744943000 -3.942326000 -0.716617000  6 -20.549970000 -2.349827000 2.143923000  8 -20.939474000 -1.261595000 2.566186000  7 -20.322833000 -3.440380000 2.972724000  1 -20.496902000 -3.270588000 3.958811000  6 -19.862930000 -4.734089000 2.628449000  8 -19.693474000 -5.585758000 3.507435000  6 -19.651968000 -4.892066000 1.203284000  1 -19.324461000 -5.850118000 0.815271000  6 -18.953840000 -0.831924000 -0.390791000  1 -18.191633000 -1.609187000 -0.279688000  8 -18.632021000 0.221368000 0.493695000  1 -19.058833000 1.003917000 0.092979000  6 -19.046992000 -0.390886000 -1.857273000  1 -18.064450000 -0.361397000 -2.338367000  8 -19.644867000 0.917588000 -1.851201000  15 -19.235350000 1.884394000 -3.176993000  8 -19.847613000 3.233470000 -2.895254000  8 -19.455036000 1.109094000 -4.453215000  8 -17.582183000 1.918261000 -2.965983000  6 -17.026683000 2.706202000 -1.896462000  1 -16.827986000 2.055711000 -1.031426000  1 -17.728608000 3.497684000 -1.601111000  6 -15.745752000 3.349925000 -2.394871000  1 -15.376678000 4.055690000 -1.643267000  8 -14.716929000 2.331309000 -2.582317000  6 -14.334498000 2.229047000 -3.934996000  1 -13.326358000 2.626648000 -4.103698000  7 -14.257020000 0.784383000 -4.282093000  6 -15.163135000 -0.104367000 -3.746888000  1 -15.903860000 0.355318000 -3.101258000  6 -13.265830000 0.371184000 -5.182491000  8 -12.481768000 1.138799000 -5.737563000  7 -13.255039000 -0.999550000 -5.402271000  1 -12.518474000 -1.326072000 -6.019617000  6 -14.111388000 -1.989726000 -4.871329000  8 -13.946098000 -3.176873000 -5.176382000  6 -15.138719000 -1.441476000 -4.007989000  1 -15.877624000 -2.112047000 -3.580410000  6 -15.400473000 2.997418000 -4.762790000  1 -16.237712000 2.306766000 -4.928024000  8 -14.979503000 3.454828000 -6.015870000  1 -14.151275000 3.997141000 -5.911244000  6 -15.914275000 4.054760000 -3.751641000  1 -16.969048000 4.287335000 -3.951319000  8 -15.116909000 5.242069000 -3.829388000  15 -15.915718000 6.628884000 -3.289498000  8 -16.384411000 6.398176000 -1.868280000  8 -16.852873000 7.118478000 -4.372442000  8 -14.562294000 7.591240000 -3.205071000  6 -14.310170000 8.586392000 -4.205789000  1 -15.107661000 9.344865000 -4.212712000  1 -14.269197000 8.135188000 -5.208250000  6 -12.992369000 9.247592000 -3.812955000  1 -13.080076000 9.603824000 -2.787037000  8 -11.890781000 8.297317000 -3.798668000  6 -11.271511000 8.265626000 -5.058573000  1 -10.180229000 8.285619000 -4.942448000  7 -11.625153000 6.981022000 -5.710161000  6 -11.141143000 6.487197000 -6.908257000  7 -10.223522000 7.084145000 -7.707945000  6 -9.957333000 6.379000000 -8.802215000  7 -9.098555000 6.888328000 -9.736746000  1 -8.531894000 7.658319000 -9.402208000  1 -8.612617000 6.243467000 -10.347745000  7 -10.555978000 5.168899000 -9.085284000  1 -10.332568000 4.692615000 -9.954195000  6 -11.524592000 4.489393000 -8.275339000  8 -11.993499000 3.409539000 -8.651235000  6 -11.793894000 5.254045000 -7.090914000  7 -12.647476000 5.000544000 -6.023741000  6 -12.516000000 6.041539000 -5.214356000  1 -13.038257000 6.180788000 -4.279644000  6 -11.762297000 9.524723000 -5.859097000  1 -12.472557000 9.209120000 -6.633992000  8 -10.729058000 10.254143000 -6.481906000  1 -10.407319000 10.847478000 -5.773703000  6 -12.488188000 10.335183000 -4.753421000  1 -13.273963000 10.997020000 -5.137063000  8 -11.448175000 11.101458000 -4.120938000  15 -11.844218000 12.313975000 -3.009762000  8 -13.140785000 12.962300000 -3.427736000  8 -10.549901000 13.058565000 -2.797722000  8 -12.222254000 11.414845000 -1.651657000  6 -11.180225000 10.666384000 -1.001373000  1 -10.511227000 11.340302000 -0.444901000  1 -10.586275000 10.112212000 -1.742837000  6 -11.827364000 9.696852000 -0.029305000  1 -12.539718000 10.241921000 0.611427000  8 -12.537598000 8.677435000 -0.774371000  6 -12.475817000 7.429220000 -0.105416000  1 -13.478108000 7.036404000 0.082457000  7 -11.806508000 6.415982000 -0.970051000  6 -10.762298000 6.742667000 -1.781973000  1 -10.480831000 7.787120000 -1.795790000  6 -12.249696000 5.071239000 -0.877684000  8 -13.145764000 4.801727000 -0.043595000  7 -11.690055000 4.144004000 -1.696416000  6 -10.680785000 4.475225000 -2.530363000  7 -10.209216000 3.519931000 -3.350555000  1 -9.387373000 3.718632000 -3.903638000  1 -10.478537000 2.530165000 -3.209290000  6 -10.152222000 5.805199000 -2.564707000  1 -9.333048000 6.076602000 -3.222657000  6 -11.710915000 7.657710000 1.216287000  1 -11.088279000 6.791187000 1.443688000  8 -12.622049000 7.923370000 2.271219000  1 -12.405649000 7.279808000 2.973845000  6 -10.859889000 8.904938000 0.857962000  1 -9.989323000 8.580293000 0.268979000  8 -10.423556000 9.687196000 1.957016000  15 -8.943875000 9.305926000 2.661658000  8 -8.603710000 10.495816000 3.522816000  8 -8.017478000 8.707294000 1.631195000  8 -9.377435000 8.008361000 3.633295000  6 -10.312029000 8.240185000 4.690295000  1 -11.172352000 8.812707000 4.316947000  1 -9.841376000 8.804137000 5.510356000  6 -10.785689000 6.910291000 5.241839000  1 -11.473399000 7.105906000 6.078772000  8 -11.510277000 6.172179000 4.211300000  6 -11.190775000 4.780673000 4.273326000  1 -12.110199000 4.183650000 4.263498000  7 -10.419435000 4.396453000 3.081602000  6 -10.438108000 3.143271000 2.482107000  7 -11.016439000 2.008720000 2.931626000  6 -10.776653000 0.978443000 2.100795000  1 -11.182698000 0.007707000 2.387611000  7 -10.085744000 0.979972000 0.942098000  6 -9.533286000 2.143089000 0.493804000  7 -8.904264000 2.162016000 -0.693413000  1 -8.306895000 2.957206000 -0.875677000  1 -8.644948000 1.267859000 -1.132296000  6 -9.677312000 3.292694000 1.308790000  7 -9.175792000 4.581993000 1.196334000  6 -9.620015000 5.207418000 2.277633000  1 -9.399224000 6.230160000 2.562536000  6 -10.398373000 4.577549000 5.566828000  1 -9.712131000 3.728059000 5.494300000  8 -11.320428000 4.397323000 6.643287000  1 -10.953310000 4.934744000 7.370939000  6 -9.693313000 5.937444000 5.712579000  1 -8.811552000 6.004676000 5.065540000  8 -9.349564000 6.205583000 7.070401000  15 -7.729310000 5.963868000 7.525781000  8 -7.684338000 6.368912000 8.975447000  8 -6.829843000 6.512825000 6.447975000  8 -7.598607000 4.302348000 7.375339000  6 -8.320578000 3.461615000 8.281091000  1 -9.376819000 3.759679000 8.326732000  1 -7.897180000 3.527072000 9.294765000  6 -8.238306000 2.019265000 7.817741000  1 -8.807612000 1.407055000 8.535966000  8 -8.841456000 1.878089000 6.505718000  6 -8.081492000 0.989088000 5.702992000  1 -8.741044000 0.244673000 5.239874000  7 -7.452397000 1.734065000 4.596317000  6 -7.124560000 1.174568000 3.377753000  7 -7.171319000 -0.147032000 3.081141000  6 -6.810812000 -0.397541000 1.828083000  7 -6.792452000 -1.685007000 1.376871000  1 -6.861104000 -2.394034000 2.095008000  1 -6.187440000 -1.902722000 0.597935000  7 -6.480747000 0.586581000 0.925330000  1 -6.332756000 0.330631000 -0.048891000  6 -6.370622000 1.986080000 1.200902000  8 -6.037741000 2.761494000 0.293581000  6 -6.700475000 2.248255000 2.572766000  7 -6.738487000 3.438923000 3.292263000  6 -7.188380000 3.101830000 4.490955000  1 -7.338144000 3.762974000 5.335838000  6 -7.058321000 0.318135000 6.627131000  1 -6.146900000 0.011838000 6.100379000  8 -7.697436000 -0.803670000 7.241484000  1 -7.454182000 -0.732588000 8.184924000  6 -6.829913000 1.416810000 7.684880000  1 -6.104709000 2.162254000 7.337935000  8 -6.436459000 0.865129000 8.942061000  15 -4.772358000 0.767165000 9.273002000  8 -4.696701000 0.161822000 10.650556000  8 -4.114908000 2.062751000 8.868780000  8 -4.288613000 -0.330153000 8.118831000  6 -4.610176000 -1.719485000 8.245032000  1 -5.697837000 -1.866018000 8.244702000  1 -4.195592000 -2.128411000 9.178067000  6 -3.999598000 -2.466541000 7.074324000  1 -4.205938000 -3.541556000 7.203403000  8 -4.580609000 -2.019816000 5.823048000  6 -3.564126000 -1.875370000 4.834459000  1 -3.870369000 -2.373364000 3.906989000  7 -3.417385000 -0.450752000 4.482430000  6 -3.380926000 0.039795000 3.188171000  7 -3.438750000 -0.701575000 2.056517000  6 -3.437102000 0.040790000 0.950631000  7 -3.627174000 -0.559963000 -0.257881000  1 -3.496139000 -1.575105000 -0.235714000  1 -3.201306000 -0.145582000 -1.087600000  7 -3.322419000 1.413990000 0.970745000  1 -3.440921000 1.927840000 0.103299000  6 -3.267971000 2.241494000 2.131157000  8 -3.204016000 3.474196000 2.002421000  6 -3.298987000 1.438265000 3.317717000  7 -3.299346000 1.804009000 4.661069000  6 -3.368342000 0.661538000 5.327037000  1 -3.438959000 0.555484000 6.402398000  6 -2.278172000 -2.534590000 5.410278000  1 -1.369662000 -2.067149000 5.015883000  8 -2.293544000 -3.933315000 5.172369000  1 -1.842512000 -4.102292000 4.314871000  6 -2.488953000 -2.268769000 6.912069000  1 -2.230484000 -1.224874000 7.126600000  8 -1.804235000 -3.127690000 7.812047000  15 -0.406486000 -2.551442000 8.539226000  8 -0.066867000 -3.576934000 9.593497000  8 -0.526608000 -1.071513000 8.821603000  8 0.666674000 -2.653814000 7.261832000  6 0.844545000 -3.928675000 6.626518000  1 -0.132724000 -4.382899000 6.415934000  1 1.426583000 -4.602460000 7.275370000  6 1.608048000 -3.738895000 5.333953000  1 1.811278000 -4.730092000 4.903594000  8 0.794023000 -2.995289000 4.384025000  6 1.588633000 -2.054438000 3.666036000  1 1.435811000 -2.171066000 2.585839000  7 1.129515000 -0.700008000 4.005304000  6 0.840231000 0.304366000 3.095988000  7 1.003669000 0.308788000 1.756530000  6 0.546479000 1.450083000 1.199860000  1 0.645844000 1.515581000 0.113795000  7 -0.028145000 2.506324000 1.798629000  6 -0.200030000 2.469685000 3.146970000  7 -0.822648000 3.521443000 3.758275000  1 -1.257263000 3.265218000 4.641924000  1 -1.490337000 3.986269000 3.140944000  6 0.274498000 1.344928000 3.861336000  7 0.209092000 0.991940000 5.205315000  6 0.722017000 -0.229799000 5.252795000  1 0.781749000 -0.861767000 6.133117000  6 3.056645000 -2.369800000 4.025084000  1 3.696055000 -1.478200000 3.968133000  8 3.502140000 -3.412172000 3.181100000  1 4.301843000 -3.125792000 2.667252000  1 2.826199000 -2.153484000 6.189515000  6 2.933180000 -2.957281000 5.450564000  8 4.025442000 -3.754829000 5.849601000  1 4.103275000 -4.459602000 5.166826000 | 1 16.687940000 9.289903000 3.933771000  8 17.522760000 9.482735000 4.434862000  6 17.489991000 10.890051000 4.634037000  1 18.191475000 11.136765000 5.443458000  1 16.485395000 11.222851000 4.945669000  6 17.863238000 11.701310000 3.392966000  1 17.564803000 12.751292000 3.541187000  8 19.301686000 11.661985000 3.154845000  6 19.560032000 11.596861000 1.765462000  1 20.283052000 12.364426000 1.468902000  7 20.189957000 10.276010000 1.441467000  6 20.106915000 9.200722000 2.299204000  1 19.476654000 9.354259000 3.171546000  6 20.899165000 10.209317000 0.238033000  8 20.954149000 11.150984000 -0.556042000  7 21.531942000 8.994444000 0.023620000  1 22.058060000 8.927686000 -0.842504000  6 21.561679000 7.859465000 0.871365000  8 22.224136000 6.866476000 0.550746000  6 20.759884000 8.029192000 2.063864000  1 20.664742000 7.196924000 2.751224000  6 18.218272000 11.771819000 1.034517000  1 18.190879000 11.225458000 0.084277000  8 17.987724000 13.168707000 0.852508000  1 17.019558000 13.269534000 0.853822000  6 17.268261000 11.185777000 2.080123000  1 17.349125000 10.094386000 2.053811000  8 15.919087000 11.609070000 1.897543000  15 14.735888000 10.418519000 2.030463000  8 13.423422000 11.150937000 2.034641000  8 15.136994000 9.424992000 3.108958000  8 14.968850000 9.576182000 0.612404000  6 14.983035000 10.293129000 -0.629842000  1 15.361985000 11.312588000 -0.479412000  1 13.966964000 10.356101000 -1.048985000  6 15.862975000 9.566911000 -1.629450000  1 15.869466000 10.156223000 -2.560820000  8 17.221337000 9.466203000 -1.124136000  6 17.783520000 8.212625000 -1.483528000  1 18.775472000 8.357374000 -1.928881000  7 17.967135000 7.385819000 -0.273988000  6 18.804256000 6.289642000 -0.189636000  7 19.547826000 5.773877000 -1.200331000  6 20.250384000 4.712125000 -0.827401000  7 21.052909000 4.064201000 -1.735440000  1 20.888911000 4.372766000 -2.687767000  1 21.079606000 3.052336000 -1.651237000  7 20.253143000 4.222988000 0.459493000  1 20.874947000 3.455677000 0.694249000  6 19.495972000 4.730241000 1.559788000  8 19.627931000 4.228064000 2.683245000  6 18.671489000 5.824418000 1.130684000  7 17.759499000 6.597604000 1.835641000  6 17.350631000 7.514613000 0.970273000  1 16.601186000 8.275163000 1.143589000  6 16.809033000 7.548510000 -2.461591000  1 16.866256000 6.456608000 -2.423926000  8 17.086118000 8.026416000 -3.778764000  1 16.205838000 8.185782000 -4.169409000  6 15.466996000 8.123131000 -1.973664000  1 15.103816000 7.594072000 -1.084709000  8 14.484222000 8.118001000 -3.007433000  15 13.332363000 6.869740000 -2.979939000  8 12.430435000 7.166646000 -4.148833000  8 12.866257000 6.661874000 -1.561449000  8 14.297199000 5.541798000 -3.306913000  6 14.836558000 5.384941000 -4.623481000  1 15.342405000 6.305055000 -4.947977000  1 14.037697000 5.156371000 -5.344422000  6 15.837210000 4.245997000 -4.635275000  1 16.205166000 4.126917000 -5.667308000  8 16.964552000 4.564951000 -3.766691000  6 17.403652000 3.398118000 -3.092239000  1 18.493037000 3.305492000 -3.158209000  7 17.058601000 3.466956000 -1.658146000  6 17.629931000 2.663751000 -0.689969000  7 18.675627000 1.816212000 -0.872806000  6 19.011250000 1.169735000 0.238624000  7 20.104346000 0.341579000 0.233895000  1 20.390782000 0.053361000 -0.694652000  1 20.125088000 -0.409521000 0.914907000  7 18.356756000 1.343224000 1.435681000  1 18.637900000 0.797285000 2.244372000  6 17.208614000 2.168025000 1.666150000  8 16.657276000 2.154794000 2.772737000  6 16.879661000 2.898359000 0.475576000  7 15.859992000 3.810502000 0.229631000  6 15.989336000 4.127640000 -1.050216000  1 15.361465000 4.807619000 -1.613824000  6 16.688140000 2.208435000 -3.760976000  1 16.567443000 1.388270000 -3.052649000  8 17.400788000 1.798802000 -4.916803000  1 17.627006000 0.859527000 -4.771332000  6 15.349295000 2.885899000 -4.123829000  1 14.762364000 3.023039000 -3.208027000  8 14.556483000 2.226755000 -5.104865000  15 13.593910000 0.954540000 -4.606298000  8 12.620113000 0.697770000 -5.729603000  8 13.171163000 1.113658000 -3.161150000  8 14.703066000 -0.302565000 -4.539122000  6 15.407495000 -0.654290000 -5.736683000  1 15.812863000 0.248729000 -6.210088000  1 14.739562000 -1.166325000 -6.445603000  6 16.530839000 -1.603794000 -5.373608000  1 17.086652000 -1.866077000 -6.286064000  8 17.446335000 -0.962925000 -4.434784000  6 17.871708000 -1.888359000 -3.439736000  1 18.964305000 -1.870059000 -3.352272000  7 17.325905000 -1.507436000 -2.123967000  6 17.840030000 -1.966425000 -0.921206000  7 18.868661000 -2.815752000 -0.733485000  6 19.059123000 -3.063148000 0.577668000  1 19.872012000 -3.751116000 0.818962000  7 18.383918000 -2.574014000 1.639399000  6 17.357537000 -1.711178000 1.411836000  7 16.707498000 -1.189792000 2.477384000  1 15.925218000 -0.565851000 2.344678000  1 16.874787000 -1.574858000 3.396363000  6 17.042456000 -1.366468000 0.074256000  7 16.062949000 -0.564419000 -0.496244000  6 16.243632000 -0.689336000 -1.803796000  1 15.606843000 -0.277340000 -2.576096000  6 17.359920000 -3.270255000 -3.868759000  1 17.158495000 -3.906399000 -2.998735000  8 18.314411000 -3.874586000 -4.738280000  1 17.781405000 -4.308455000 -5.431182000  6 16.097506000 -2.886753000 -4.657335000  1 15.268412000 -2.661221000 -3.980158000  8 15.738960000 -3.891496000 -5.601515000  15 14.353737000 -4.802139000 -5.262201000  8 14.246560000 -5.794161000 -6.385362000  8 13.244645000 -3.857112000 -4.850367000  8 14.804775000 -5.531185000 -3.834735000  6 15.843347000 -6.518990000 -3.798557000  1 16.762013000 -6.134846000 -4.264172000  1 15.528087000 -7.424152000 -4.337649000  6 16.118560000 -6.892918000 -2.350872000  1 16.845911000 -7.719592000 -2.343592000  8 16.691294000 -5.766596000 -1.627252000  6 16.118314000 -5.669363000 -0.330584000  1 16.906341000 -5.530310000 0.419017000  7 15.237655000 -4.484214000 -0.275639000  6 14.733873000 -3.883919000 0.863384000  7 14.974173000 -4.269114000 2.140373000  6 14.350918000 -3.507195000 3.032362000  7 14.534802000 -3.746657000 4.366561000  1 14.928489000 -4.656647000 4.573957000  1 13.818805000 -3.440804000 5.014072000  7 13.560446000 -2.432307000 2.685217000  1 13.122042000 -1.878233000 3.414816000  6 13.296085000 -1.968435000 1.355353000  8 12.604891000 -0.956421000 1.186772000  6 13.933454000 -2.824648000 0.399202000  7 13.930510000 -2.788593000 -0.987235000  6 14.709742000 -3.791242000 -1.354642000  1 14.895461000 -4.124763000 -2.363698000  6 15.308586000 -6.949638000 -0.096092000  1 14.461859000 -6.771802000 0.576382000  8 16.169843000 -7.965322000 0.417998000  1 15.907237000 -8.773173000 -0.064160000  6 14.883761000 -7.301617000 -1.533773000  1 14.018554000 -6.708335000 -1.847555000  8 14.629925000 -8.697547000 -1.672828000  15 13.005540000 -9.165384000 -1.838932000  8 13.044595000 -10.670359000 -1.850231000  8 12.354851000 -8.313540000 -2.899042000  8 12.378370000 -8.595001000 -0.396674000  6 12.771291000 -9.216042000 0.832218000  1 13.866667000 -9.244769000 0.920913000  1 12.392583000 -10.247880000 0.881662000  6 12.191804000 -8.440412000 1.999796000  1 12.470574000 -8.967692000 2.926102000  8 12.746573000 -7.095752000 2.047458000  6 11.739505000 -6.161927000 2.401729000  1 12.105775000 -5.497955000 3.193895000  7 11.414870000 -5.314607000 1.233107000  6 10.743082000 -4.107331000 1.280214000  7 10.218356000 -3.519713000 2.385049000  6 9.610538000 -2.370611000 2.116023000  7 9.009018000 -1.655789000 3.122257000  1 8.945264000 -2.174420000 3.991375000  1 8.144787000 -1.181605000 2.877221000  7 9.575025000 -1.811445000 0.857477000  1 9.179716000 -0.883901000 0.742136000  6 10.141922000 -2.365878000 -0.320614000  8 10.118291000 -1.718210000 -1.381331000  6 10.692441000 -3.661489000 -0.052899000  7 11.290619000 -4.582193000 -0.902829000  6 11.705753000 -5.558241000 -0.109235000  1 12.181786000 -6.481479000 -0.411376000  6 10.518234000 -6.970926000 2.850679000  1 9.581904000 -6.429307000 2.685814000  8 10.674112000 -7.307218000 4.230316000  1 10.404945000 -8.243945000 4.284754000  6 10.670134000 -8.235376000 1.986267000  1 10.313620000 -8.064327000 0.964225000  8 10.018040000 -9.361234000 2.570948000  15 8.488620000 -9.766372000 1.950596000  8 8.069655000 -10.979005000 2.738830000  8 8.530924000 -9.689226000 0.445638000  8 7.605968000 -8.425924000 2.416743000  6 7.306920000 -8.237776000 3.802839000  1 8.224780000 -8.277183000 4.404998000  1 6.619197000 -9.017685000 4.161188000  6 6.644316000 -6.889226000 3.996656000  1 6.434462000 -6.761447000 5.070169000  8 7.535765000 -5.822884000 3.564521000  6 6.800829000 -4.811729000 2.902923000  1 7.069967000 -3.824367000 3.296194000  7 7.125437000 -4.797168000 1.465000000  6 6.913938000 -3.714711000 0.628664000  7 6.448283000 -2.490914000 0.947049000  6 6.371459000 -1.694663000 -0.141428000  1 5.986368000 -0.691163000 0.039298000  7 6.681199000 -1.985925000 -1.417121000  6 7.133278000 -3.231434000 -1.708860000  7 7.449708000 -3.523672000 -3.003276000  1 7.414479000 -4.502181000 -3.264372000  1 7.054464000 -2.892428000 -3.690965000  6 7.283401000 -4.164131000 -0.655910000  7 7.700652000 -5.489482000 -0.614042000  6 7.588872000 -5.837094000 0.661322000  1 7.806031000 -6.811043000 1.085150000  6 5.316908000 -5.123760000 3.112384000  1 4.690352000 -4.741272000 2.302450000  8 4.929557000 -4.561158000 4.373483000  1 4.177854000 -5.104082000 4.675888000  6 5.338131000 -6.656050000 3.218291000  1 5.397052000 -7.117975000 2.226611000  8 4.221704000 -7.148964000 3.947795000  15 3.251252000 -8.360217000 3.254362000  8 2.633833000 -9.067221000 4.433380000  8 4.017620000 -9.043294000 2.149484000  8 2.100967000 -7.437791000 2.467392000  6 1.267579000 -6.582188000 3.259508000  1 1.738507000 -6.359700000 4.226776000  1 0.299386000 -7.067765000 3.456293000  6 1.016114000 -5.280317000 2.524081000  1 0.408358000 -4.628663000 3.170658000  8 2.291563000 -4.643128000 2.261269000  6 2.242714000 -3.926964000 1.036978000  1 2.580069000 -2.897724000 1.185223000  7 3.170491000 -4.549820000 0.075047000  6 3.511391000 -3.948130000 -1.120464000  7 3.225861000 -2.670919000 -1.462418000  6 3.571931000 -2.393743000 -2.714504000  7 3.406585000 -1.125177000 -3.193636000  1 2.723866000 -0.572658000 -2.689726000  1 3.390981000 -1.022273000 -4.202740000  7 4.159171000 -3.307104000 -3.562062000  1 4.128071000 -3.088200000 -4.561298000  6 4.493589000 -4.656161000 -3.238988000  8 5.000790000 -5.389525000 -4.101950000  6 4.142324000 -4.950875000 -1.877546000  7 4.186131000 -6.147478000 -1.163354000  6 3.584553000 -5.881492000 -0.013854000  1 3.401725000 -6.576325000 0.796797000  6 0.805613000 -4.027721000 0.506164000  1 0.792505000 -4.067784000 -0.588664000  8 0.033369000 -2.917293000 0.963247000  1 -0.827874000 -3.027500000 0.488813000  6 0.357669000 -5.364770000 1.141395000  1 0.788460000 -6.194516000 0.565732000  8 -1.050931000 -5.530332000 1.273201000  15 -1.977641000 -5.549668000 -0.120673000  8 -3.316225000 -6.124226000 0.262210000  8 -1.144875000 -6.033866000 -1.286991000  8 -2.120321000 -3.867282000 -0.317968000  6 -3.406638000 -3.244839000 -0.413586000  1 -3.355618000 -2.309074000 0.158915000  1 -4.180910000 -3.900041000 0.006204000  6 -3.757472000 -2.934734000 -1.855732000  1 -4.749637000 -2.456779000 -1.893523000  8 -2.763634000 -2.029974000 -2.403142000  6 -2.630287000 -2.251834000 -3.814184000  1 -2.890508000 -1.347797000 -4.376552000  7 -1.226980000 -2.526632000 -4.108770000  6 -0.498963000 -1.900375000 -5.101556000  7 -0.969692000 -0.953706000 -5.942991000  6 -0.050040000 -0.514232000 -6.795936000  7 -0.362464000 0.485217000 -7.667644000  1 -1.353694000 0.665446000 -7.767823000  1 0.181027000 0.587717000 -8.515040000  7 1.246430000 -0.986431000 -6.803264000  1 1.923658000 -0.581983000 -7.443956000  6 1.790620000 -1.964669000 -5.925273000  8 3.009203000 -2.225830000 -6.014442000  6 0.790139000 -2.465768000 -5.031970000  7 0.847914000 -3.424221000 -4.022003000  6 -0.364438000 -3.439800000 -3.493607000  1 -0.692979000 -4.074967000 -2.674997000  6 -3.586781000 -3.402400000 -4.190659000  1 -3.124121000 -4.064570000 -4.939284000  8 -4.792337000 -2.834020000 -4.665226000  1 -5.366309000 -3.625367000 -4.870411000  6 -3.770785000 -4.115349000 -2.826918000  1 -2.921669000 -4.787351000 -2.638206000  8 -4.998684000 -4.804700000 -2.664065000  15 -5.446674000 -5.962463000 -3.786253000  8 -4.386455000 -7.030487000 -3.885925000  8 -5.970783000 -5.234961000 -5.019187000  8 -6.785190000 -6.475674000 -2.959558000  6 -6.586628000 -7.308395000 -1.799322000  1 -5.554294000 -7.240483000 -1.434008000  1 -6.790053000 -8.353275000 -2.075422000  6 -7.538907000 -6.929005000 -0.681395000  1 -7.510888000 -7.741451000 0.062845000  8 -7.117902000 -5.696907000 -0.028750000  6 -8.254036000 -4.952233000 0.376460000  1 -8.165990000 -4.658799000 1.427951000  7 -8.327792000 -3.683203000 -0.412687000  6 -7.612378000 -3.524428000 -1.569125000  1 -7.048974000 -4.390622000 -1.890294000  6 -9.085763000 -2.636358000 0.135529000  8 -9.730862000 -2.776699000 1.180563000  7 -9.038027000 -1.455214000 -0.572623000  1 -9.546136000 -0.652888000 -0.112400000  6 -8.256430000 -1.192826000 -1.695560000  8 -8.162821000 -0.030085000 -2.153632000  6 -7.568772000 -2.338309000 -2.238224000  1 -6.952623000 -2.231876000 -3.125188000  6 -9.482637000 -5.833782000 0.127520000  1 -10.384873000 -5.248794000 -0.071890000  8 -9.666591000 -6.679907000 1.264088000  1 -9.915235000 -7.544851000 0.889355000  6 -8.998802000 -6.663727000 -1.072478000  1 -9.045804000 -6.079086000 -1.998243000  8 -9.713929000 -7.888215000 -1.217031000  15 -10.972255000 -7.909553000 -2.358026000  8 -11.425537000 -9.346299000 -2.390694000  8 -10.564908000 -7.113063000 -3.571596000  8 -12.108768000 -6.958459000 -1.578899000  6 -12.723922000 -7.502966000 -0.407267000  1 -11.962756000 -7.874689000 0.292485000  1 -13.385514000 -8.342268000 -0.669400000  6 -13.550324000 -6.440818000 0.285145000  1 -14.017980000 -6.904556000 1.169788000  8 -12.707923000 -5.344381000 0.712970000  6 -13.403235000 -4.113680000 0.575881000  1 -13.371075000 -3.547468000 1.515204000  7 -12.754748000 -3.280144000 -0.440558000  6 -12.861855000 -1.903007000 -0.465910000  7 -13.536108000 -1.153194000 0.431214000  6 -13.498659000 0.153352000 0.133253000  7 -14.142927000 1.031350000 0.925560000  1 -14.539211000 0.666473000 1.778583000  1 -14.122557000 2.048549000 0.762893000  7 -12.835491000 0.658942000 -0.961618000  1 -12.874896000 1.690367000 -1.116778000  6 -12.118317000 -0.096836000 -1.907531000  8 -11.553453000 0.464284000 -2.874088000  6 -12.159024000 -1.496839000 -1.613070000  7 -11.632481000 -2.600993000 -2.281389000  6 -12.014075000 -3.648713000 -1.566961000  1 -11.811292000 -4.690603000 -1.785354000  6 -14.850804000 -4.450648000 0.186880000  1 -15.296419000 -3.664632000 -0.436357000  8 -15.604712000 -4.656323000 1.379956000  1 -16.183615000 -5.420962000 1.190598000  6 -14.660582000 -5.788509000 -0.551214000  1 -14.343671000 -5.633616000 -1.589362000  8 -15.832056000 -6.599853000 -0.488391000  15 -16.930103000 -6.544050000 -1.757701000  8 -17.982488000 -7.573306000 -1.417682000  8 -16.202549000 -6.514110000 -3.079895000  8 -17.540381000 -5.011282000 -1.412508000  6 -18.757051000 -4.671302000 -2.101521000  1 -19.336783000 -5.580592000 -2.313343000  1 -18.524841000 -4.165753000 -3.052040000  6 -19.602523000 -3.732331000 -1.255818000  1 -20.453157000 -3.403425000 -1.864961000  8 -20.131996000 -4.419238000 -0.078840000  6 -19.625683000 -3.839623000 1.107325000  1 -20.397864000 -3.282496000 1.651512000  7 -19.187898000 -4.926092000 2.018783000  6 -18.597699000 -6.058665000 1.508740000  1 -18.618239000 -6.146491000 0.427252000  6 -19.282878000 -4.698754000 3.399978000  8 -19.821201000 -3.707807000 3.892295000  7 -18.711260000 -5.703899000 4.168447000  1 -18.763942000 -5.556411000 5.171927000  6 -18.055647000 -6.882811000 3.739262000  8 -17.582030000 -7.665701000 4.569612000  6 -18.040150000 -7.020385000 2.296776000  1 -17.588731000 -7.898614000 1.848977000  6 -18.451142000 -2.905492000 0.695356000  1 -17.529891000 -3.495259000 0.673038000  8 -18.240372000 -1.803143000 1.552905000  1 -18.897522000 -1.143834000 1.256066000  6 -18.860252000 -2.504122000 -0.727339000  1 -17.997497000 -2.238539000 -1.346437000  8 -19.748848000 -1.381463000 -0.587251000  15 -19.826244000 -0.360880000 -1.932748000  8 -20.737204000 0.769365000 -1.524138000  8 -20.013401000 -1.186563000 -3.182241000  8 -18.235256000 0.133543000 -1.973288000  6 -17.757635000 1.048492000 -0.969354000  1 -17.300439000 0.480303000 -0.145407000  1 -18.588009000 1.650991000 -0.577151000  6 -16.742400000 1.976838000 -1.610834000  1 -16.473160000 2.762153000 -0.896911000  8 -15.522938000 1.244593000 -1.936069000  6 -15.303993000 1.195525000 -3.326180000  1 -14.442599000 1.806285000 -3.622085000  7 -14.942625000 -0.204395000 -3.684552000  6 -15.534305000 -1.258412000 -3.026028000  1 -16.261085000 -0.959332000 -2.279020000  6 -14.014640000 -0.408515000 -4.714458000  8 -13.512052000 0.500398000 -5.373084000  7 -13.718493000 -1.747626000 -4.934021000  1 -13.020795000 -1.917362000 -5.651443000  6 -14.250390000 -2.890599000 -4.295051000  8 -13.869871000 -4.019200000 -4.628270000  6 -15.241721000 -2.562368000 -3.290746000  1 -15.738926000 -3.367543000 -2.759229000  6 -16.613759000 1.679720000 -4.008309000  1 -17.278481000 0.809346000 -4.085084000  8 -16.465013000 2.211182000 -5.293976000  1 -15.781740000 2.934755000 -5.272611000  6 -17.236259000 2.600681000 -2.926562000  1 -18.332585000 2.578372000 -2.993956000  8 -16.750000000 3.938352000 -3.085496000  15 -17.751665000 5.116648000 -2.409254000  8 -18.010585000 4.760687000 -0.960353000  8 -18.877664000 5.428918000 -3.371052000  8 -16.619416000 6.335462000 -2.416670000  6 -16.684905000 7.390560000 -3.384364000  1 -17.612872000 7.970962000 -3.268847000  1 -16.667557000 6.987699000 -4.407807000  6 -15.489413000 8.294734000 -3.098616000  1 -15.525270000 8.594137000 -2.051374000  8 -14.225712000 7.591209000 -3.255561000  6 -13.773466000 7.715192000 -4.579471000  1 -12.700908000 7.948195000 -4.589824000  7 -13.949294000 6.401271000 -5.246166000  6 -13.545854000 6.044435000 -6.520471000  7 -12.905868000 6.841211000 -7.410826000  6 -12.652854000 6.228403000 -8.562448000  7 -12.075700000 6.932549000 -9.582562000  1 -11.650700000 7.805650000 -9.294676000  1 -11.557804000 6.425027000 -10.288973000  7 -13.002444000 4.917706000 -8.814013000  1 -12.801822000 4.512884000 -9.723947000  6 -13.672835000 4.028973000 -7.909901000  8 -13.938491000 2.874541000 -8.262320000  6 -13.934891000 4.700514000 -6.669068000  7 -14.553259000 4.248591000 -5.509504000  6 -14.536044000 5.280282000 -4.678064000  1 -14.942735000 5.290122000 -3.677916000  6 -14.592384000 8.875094000 -5.251677000  1 -15.314038000 8.449228000 -5.960396000  8 -13.801556000 9.816541000 -5.941670000  1 -13.523782000 10.440447000 -5.240967000  6 -15.325513000 9.489591000 -4.029384000  1 -16.265902000 9.991933000 -4.286674000  8 -14.387012000 10.428210000 -3.475862000  15 -14.874402000 11.505789000 -2.265795000  8 -16.311853000 11.897423000 -2.503553000  8 -13.731573000 12.484513000 -2.160382000  8 -14.908768000 10.511012000 -0.922135000  6 -13.671612000 9.975646000 -0.420889000  1 -13.089172000 10.760196000 0.085524000  1 -13.069513000 9.566428000 -1.245366000  6 -13.998606000 8.879624000 0.576942000  1 -14.725457000 9.260944000 1.312460000  8 -14.579655000 7.751407000 -0.125584000  6 -14.186546000 6.528896000 0.474171000  1 -15.059315000 5.926479000 0.739681000  7 -13.409455000 5.703820000 -0.496025000  6 -12.587121000 6.265297000 -1.425578000  1 -12.559052000 7.346278000 -1.443578000  6 -13.497186000 4.293039000 -0.375375000  8 -14.158538000 3.825822000 0.581666000  7 -12.872731000 3.521811000 -1.301397000  6 -12.098705000 4.079598000 -2.257077000  7 -11.555954000 3.263209000 -3.177201000  1 -10.888135000 3.646778000 -3.831017000  1 -11.577171000 2.236409000 -3.042951000  6 -11.890627000 5.494924000 -2.311571000  1 -11.252324000 5.949395000 -3.062318000  6 -13.350313000 6.883435000 1.719907000  1 -12.553056000 6.153141000 1.857612000  8 -14.177861000 6.952850000 2.870691000  1 -13.790000000 6.315323000 3.500645000  6 -12.800415000 8.276556000 1.320311000  1 -11.959265000 8.136867000 0.624979000  8 -12.392839000 9.104442000 2.396870000  15 -10.788660000 9.007184000 2.898379000  8 -10.592396000 10.196808000 3.803390000  8 -9.900023000 8.672433000 1.724976000  8 -10.817745000 7.597439000 3.809004000  6 -11.636509000 7.566867000 4.980917000  1 -12.629133000 7.983214000 4.760270000  1 -11.179185000 8.153683000 5.792637000  6 -11.782460000 6.135768000 5.458658000  1 -12.393876000 6.137137000 6.373983000  8 -12.470536000 5.340701000 4.444640000  6 -11.896625000 4.036680000 4.358573000  1 -12.687083000 3.277371000 4.376268000  7 -11.183746000 3.886033000 3.079045000  6 -10.963494000 2.679318000 2.428245000  7 -11.229431000 1.431164000 2.871498000  6 -10.818261000 0.504108000 1.988050000  1 -10.960149000 -0.541176000 2.262830000  7 -10.237575000 0.696404000 0.785973000  6 -10.002603000 1.964425000 0.345985000  7 -9.479767000 2.150135000 -0.877815000  1 -9.099460000 3.064579000 -1.079741000  1 -9.079177000 1.345616000 -1.379692000  6 -10.348018000 3.030235000 1.213221000  7 -10.161941000 4.402333000 1.123357000  6 -10.651672000 4.880452000 2.259238000  1 -10.636852000 5.916713000 2.577680000  6 -10.947260000 3.904089000 5.550216000  1 -10.134283000 3.201961000 5.344964000  8 -11.696899000 3.492351000 6.694097000  1 -11.337223000 4.028273000 7.426342000  6 -10.483695000 5.360230000 5.730738000  1 -9.713965000 5.631513000 4.999286000  8 -10.029030000 5.600916000 7.060425000  15 -8.347373000 5.642622000 7.312330000  8 -8.198811000 5.951822000 8.778695000  8 -7.705236000 6.424781000 6.195118000  8 -7.932532000 4.050045000 7.002890000  6 -8.357674000 3.034226000 7.917266000  1 -9.430510000 3.134482000 8.132889000  1 -7.806199000 3.108065000 8.866732000  6 -8.108152000 1.661398000 7.319593000  1 -8.476344000 0.914722000 8.041792000  8 -8.854446000 1.516504000 6.082176000  6 -8.082965000 0.825980000 5.117116000  1 -8.686124000 0.034131000 4.656175000  7 -7.687725000 1.745749000 4.031206000  6 -7.267459000 1.317667000 2.789915000  7 -7.011370000 0.031307000 2.449467000  6 -6.587467000 -0.088245000 1.199464000  7 -6.232716000 -1.320727000 0.720988000  1 -6.156314000 -2.026919000 1.443276000  1 -5.476904000 -1.335983000 0.049397000  7 -6.509658000 0.971934000 0.323503000  1 -6.352764000 0.783171000 -0.663745000  6 -6.746107000 2.349208000 0.639985000  8 -6.647180000 3.208147000 -0.246144000  6 -7.095282000 2.485230000 2.025835000  7 -7.368153000 3.611864000 2.794379000  6 -7.714461000 3.140408000 3.983521000  1 -7.957719000 3.720903000 4.863935000  6 -6.862104000 0.255332000 5.843985000  1 -5.996322000 0.138365000 5.182852000  8 -7.236786000 -0.993391000 6.428874000  1 -6.860756000 -0.956882000 7.330172000  6 -6.656366000 1.301123000 6.960403000  1 -6.104148000 2.174430000 6.593059000  8 -6.013263000 0.727383000 8.099429000  15 -4.313225000 0.799270000 8.150200000  8 -3.939461000 0.054995000 9.405444000  8 -3.878486000 2.206304000 7.825051000  8 -3.925338000 -0.086876000 6.796790000  6 -3.934804000 -1.517917000 6.806069000  1 -4.965313000 -1.891954000 6.874978000  1 -3.357367000 -1.903047000 7.658659000  6 -3.298332000 -2.016888000 5.521886000  1 -3.288083000 -3.118981000 5.547137000  8 -4.067300000 -1.576047000 4.372123000  6 -3.192041000 -1.167877000 3.325450000  1 -3.476163000 -1.652519000 2.385356000  7 -3.345075000 0.278674000 3.098471000  6 -3.412234000 0.900257000 1.862309000  7 -3.326438000 0.296731000 0.649292000  6 -3.457499000 1.151867000 -0.366443000  7 -3.520394000 0.683918000 -1.643433000  1 -3.172943000 -0.264114000 -1.812192000  1 -3.361254000 1.336393000 -2.400175000  7 -3.603209000 2.509971000 -0.185519000  1 -3.739989000 3.106765000 -0.994863000  6 -3.688492000 3.194052000 1.060780000  8 -3.805639000 4.429372000 1.079656000  6 -3.604017000 2.265158000 2.146520000  7 -3.658593000 2.479917000 3.519822000  6 -3.500311000 1.281440000 4.058193000  1 -3.523165000 1.046543000 5.115504000  6 -1.759170000 -1.604758000 3.734554000  1 -1.005674000 -0.924652000 3.328021000  8 -1.543474000 -2.942485000 3.315759000  1 -0.890249000 -2.925387000 2.582361000  6 -1.866440000 -1.521061000 5.270633000  1 -1.779195000 -0.474501000 5.583955000  8 -0.944284000 -2.325664000 5.988876000  15 0.036244000 -1.648799000 7.172328000  8 0.166603000 -2.711922000 8.237185000  8 -0.365917000 -0.221405000 7.463992000  8 1.484211000 -1.507610000 6.338503000  6 1.993113000 -2.663623000 5.665134000  1 1.212345000 -3.424828000 5.542460000  1 2.818136000 -3.107007000 6.246468000  6 2.521259000 -2.273084000 4.297330000  1 2.887057000 -3.178857000 3.796890000  8 1.438748000 -1.733399000 3.506198000  6 1.881593000 -0.642559000 2.719746000  1 1.644415000 -0.813619000 1.662825000  7 1.134069000 0.562640000 3.096693000  6 0.651111000 1.497102000 2.194309000  7 0.781375000 1.496737000 0.853933000  6 0.163580000 2.562956000 0.308297000  1 0.221723000 2.638813000 -0.780174000  7 -0.523535000 3.547590000 0.919444000  6 -0.658041000 3.502604000 2.270388000  7 -1.370940000 4.492862000 2.890295000  1 -1.764803000 4.201515000 3.780744000  1 -2.083042000 4.902474000 2.283884000  6 -0.035681000 2.446218000 2.978439000  7 0.013054000 2.100422000 4.326950000  6 0.720088000 0.977355000 4.359224000  1 0.932260000 0.383828000 5.241594000  6 3.406584000 -0.524925000 2.912543000  1 3.735058000 0.526995000 2.902706000  8 4.004937000 -1.267325000 1.865129000  1 4.859302000 -1.633800000 2.177820000  1 3.452412000 -0.472817000 5.083778000  6 3.628460000 -1.206370000 4.283064000  8 4.943400000 -1.732905000 4.416905000  1 4.886320000 -2.714076000 4.411492000  19 12.256386000 -1.908381000 -3.228004000 | 1 16.762753000 9.123245000 4.037795000  8 17.644687000 9.348786000 4.433161000  6 17.573632000 10.749920000 4.663095000  1 18.360086000 11.018375000 5.382464000  1 16.602716000 11.030266000 5.105310000  6 17.749337000 11.594858000 3.401253000  1 17.421728000 12.626564000 3.605322000  8 19.146734000 11.630358000 2.985476000  6 19.231752000 11.597603000 1.573729000  1 19.872705000 12.405475000 1.204427000  7 19.881864000 10.316238000 1.147744000  6 19.958522000 9.225267000 1.986049000  1 19.430086000 9.330662000 2.930249000  6 20.444322000 10.306423000 -0.132465000  8 20.353011000 11.260886000 -0.907629000  7 21.111566000 9.132190000 -0.445644000  1 21.536022000 9.108527000 -1.367950000  6 21.306337000 7.990098000 0.369813000  8 21.983636000 7.043350000 -0.045619000  6 20.640721000 8.095470000 1.650408000  1 20.673356000 7.249399000 2.326585000  6 17.802319000 11.712828000 1.018886000  1 17.683207000 11.177032000 0.069834000  8 17.482413000 13.098253000 0.892639000  1 16.518322000 13.149164000 1.016504000  6 17.020269000 11.066594000 2.163301000  1 17.149844000 9.981118000 2.106933000  8 15.640012000 11.423401000 2.158190000  15 14.541510000 10.172698000 2.410982000  8 13.205282000 10.837559000 2.587239000  8 15.118828000 9.187192000 3.414298000  8 14.640528000 9.360378000 0.960337000  6 14.467165000 10.090532000 -0.261803000  1 14.810588000 11.126552000 -0.141407000  1 13.405678000 10.105969000 -0.553273000  6 15.253986000 9.421221000 -1.372376000  1 15.118428000 10.020250000 -2.287430000  8 16.666653000 9.385619000 -1.035435000  6 17.243971000 8.167617000 -1.481532000  1 18.166487000 8.368798000 -2.039787000  7 17.613935000 7.336816000 -0.318501000  6 18.510492000 6.286489000 -0.358631000  7 19.157782000 5.832677000 -1.461050000  6 19.953252000 4.804191000 -1.199020000  7 20.678437000 4.218723000 -2.209187000  1 20.391453000 4.542476000 -3.126827000  1 20.749729000 3.206480000 -2.159201000  7 20.130359000 4.289948000 0.065603000  1 20.813522000 3.552103000 0.205829000  6 19.478961000 4.731370000 1.258267000  8 19.764733000 4.213704000 2.345286000  6 18.555413000 5.788239000 0.955431000  7 17.694073000 6.498141000 1.780412000  6 17.140741000 7.408496000 0.991438000  1 16.378987000 8.124409000 1.269962000  6 16.193272000 7.465155000 -2.347407000  1 16.309516000 6.377304000 -2.338163000  8 16.284209000 7.974670000 -3.678635000  1 15.356250000 8.098955000 -3.954813000  6 14.892591000 7.962303000 -1.691406000  1 14.666737000 7.404166000 -0.775234000  8 13.793459000 7.916852000 -2.599347000  15 12.736467000 6.592013000 -2.477071000  8 11.678238000 6.860167000 -3.514475000  8 12.466420000 6.311202000 -1.020796000  8 13.733893000 5.341959000 -2.967959000  6 14.119673000 5.254808000 -4.343111000  1 14.535215000 6.210646000 -4.691162000  1 13.254343000 5.002753000 -4.973810000  6 15.171659000 4.175142000 -4.508199000  1 15.430729000 4.111197000 -5.577568000  8 16.366424000 4.529024000 -3.753191000  6 16.935421000 3.370043000 -3.170652000  1 18.013236000 3.334414000 -3.362357000  7 16.757840000 3.383726000 -1.705935000  6 17.497784000 2.602655000 -0.840109000  7 18.575522000 1.846780000 -1.174448000  6 19.090079000 1.200679000 -0.133907000  7 20.241665000 0.473306000 -0.296958000  1 20.432801000 0.226771000 -1.261322000  1 20.414743000 -0.283454000 0.355030000  7 18.569018000 1.285144000 1.135410000  1 18.970817000 0.724996000 1.881098000  6 17.400947000 2.015492000 1.529876000  8 16.988760000 1.929343000 2.692049000  6 16.880314000 2.752059000 0.413757000  7 15.778296000 3.594337000 0.322143000  6 15.727542000 3.950644000 -0.952968000  1 14.992519000 4.600420000 -1.413527000  6 16.212563000 2.159005000 -3.793823000  1 16.202244000 1.321567000 -3.095656000  8 16.827648000 1.809299000 -5.022641000  1 17.133083000 0.886677000 -4.924107000  6 14.810249000 2.771435000 -4.007095000  1 14.303918000 2.840975000 -3.036394000  8 13.968235000 2.112041000 -4.944577000  15 13.187606000 0.710873000 -4.476789000  8 12.177790000 0.422019000 -5.558621000  8 12.840361000 0.732343000 -3.000960000  8 14.432180000 -0.401880000 -4.545852000  6 15.011345000 -0.703407000 -5.820330000  1 15.342782000 0.221214000 -6.309691000  1 14.283291000 -1.214745000 -6.466653000  6 16.180140000 -1.638068000 -5.595753000  1 16.631639000 -1.895076000 -6.565369000  8 17.191676000 -0.997703000 -4.759212000  6 17.748698000 -1.942537000 -3.846409000  1 18.843246000 -1.923421000 -3.903223000  7 17.384145000 -1.589006000 -2.463370000  6 18.082197000 -2.024618000 -1.346912000  7 19.186309000 -2.794829000 -1.310038000  6 19.556708000 -3.050576000 -0.039474000  1 20.443463000 -3.676297000 0.080859000  7 18.982014000 -2.638247000 1.110894000  6 17.873384000 -1.854898000 1.035108000  7 17.324763000 -1.398999000 2.187258000  1 16.458378000 -0.880789000 2.158232000  1 17.593465000 -1.831018000 3.060662000  6 17.371798000 -1.508763000 -0.243460000  7 16.266701000 -0.780965000 -0.664600000  6 16.287420000 -0.871987000 -1.987265000  1 15.528768000 -0.497948000 -2.663248000  6 17.183152000 -3.319568000 -4.227637000  1 17.092595000 -3.967430000 -3.346883000  8 18.016295000 -3.909924000 -5.223007000  1 17.403324000 -4.357450000 -5.835013000  6 15.831760000 -2.916912000 -4.830405000  1 15.118524000 -2.667521000 -4.040846000  8 15.310555000 -3.912846000 -5.705207000  15 13.893000000 -4.693358000 -5.224046000  8 13.540511000 -5.614836000 -6.355645000  8 12.939617000 -3.667598000 -4.640364000  8 14.431742000 -5.527424000 -3.887986000  6 15.475954000 -6.506309000 -3.965083000  1 16.325055000 -6.125423000 -4.549050000  1 15.104018000 -7.424473000 -4.443452000  6 15.929296000 -6.853152000 -2.553511000  1 16.667603000 -7.666962000 -2.620724000  8 16.562759000 -5.707809000 -1.914908000  6 16.118709000 -5.575811000 -0.571806000  1 16.975947000 -5.406544000 0.090703000  7 15.235171000 -4.395206000 -0.466678000  6 14.832550000 -3.760218000 0.695561000  7 15.200710000 -4.096216000 1.955455000  6 14.679913000 -3.291196000 2.875029000  7 15.014135000 -3.466761000 4.188757000  1 15.432224000 -4.366005000 4.394622000  1 14.379966000 -3.123382000 4.899433000  7 13.860514000 -2.224638000 2.565836000  1 13.503796000 -1.635720000 3.312664000  6 13.443643000 -1.826429000 1.252056000  8 12.727707000 -0.827920000 1.113140000  6 13.982855000 -2.721764000 0.272981000  7 13.856616000 -2.732043000 -1.108573000  6 14.611036000 -3.739907000 -1.514198000  1 14.705213000 -4.105656000 -2.523037000  6 15.354119000 -6.857495000 -0.219188000  1 14.567669000 -6.665917000 0.520339000  8 16.275347000 -7.840337000 0.250040000  1 15.981660000 -8.669244000 -0.175743000  6 14.802512000 -7.272713000 -1.597166000  1 13.888745000 -6.721528000 -1.840369000  8 14.592048000 -8.681433000 -1.660406000  15 12.982221000 -9.219652000 -1.716485000  8 13.082002000 -10.720035000 -1.640555000  8 12.246624000 -8.458895000 -2.789450000  8 12.405357000 -8.587078000 -0.278874000  6 12.873701000 -9.125526000 0.962666000  1 13.972714000 -9.123634000 0.998257000  1 12.524961000 -10.161282000 1.088434000  6 12.329835000 -8.296103000 2.110752000  1 12.644474000 -8.773846000 3.052171000  8 12.876839000 -6.947504000 2.071152000  6 11.874862000 -6.001232000 2.408894000  1 12.262175000 -5.292602000 3.150886000  7 11.509915000 -5.221601000 1.205547000  6 10.866904000 -3.998125000 1.197168000  7 10.374551000 -3.335949000 2.273075000  6 9.789829000 -2.189042000 1.948037000  7 9.214226000 -1.409104000 2.918994000  1 9.147231000 -1.879648000 3.814808000  1 8.358324000 -0.929361000 2.656794000  7 9.754780000 -1.696615000 0.660653000  1 9.400024000 -0.758794000 0.503388000  6 10.304642000 -2.325673000 -0.483251000  8 10.317866000 -1.725517000 -1.575937000  6 10.804006000 -3.625125000 -0.158012000  7 11.360694000 -4.607012000 -0.965295000  6 11.766563000 -5.547136000 -0.125940000  1 12.209654000 -6.499749000 -0.382603000  6 10.675112000 -6.791196000 2.939307000  1 9.730527000 -6.263513000 2.776283000  8 10.877204000 -7.054680000 4.328832000  1 10.612682000 -7.987848000 4.440138000  6 10.806826000 -8.098734000 2.138013000  1 10.416339000 -7.983146000 1.120596000  8 10.178062000 -9.193427000 2.801964000  15 8.624551000 -9.617955000 2.257176000  8 8.212799000 -10.765426000 3.141210000  8 8.623740000 -9.648415000 0.750071000  8 7.776068000 -8.233446000 2.653439000  6 7.498474000 -7.960698000 4.030158000  1 8.425926000 -7.961861000 4.619013000  1 6.818760000 -8.718359000 4.446066000  6 6.835896000 -6.604311000 4.153468000  1 6.635417000 -6.417507000 5.220148000  8 7.722456000 -5.562556000 3.655323000  6 6.978834000 -4.586474000 2.951360000  1 7.253140000 -3.579768000 3.288328000  7 7.285068000 -4.647302000 1.510946000  6 7.076156000 -3.604980000 0.624514000  7 6.625595000 -2.362472000 0.887020000  6 6.550263000 -1.618315000 -0.237755000  1 6.178413000 -0.602599000 -0.102802000  7 6.848651000 -1.973583000 -1.499996000  6 7.283067000 -3.237248000 -1.734836000  7 7.587767000 -3.594044000 -3.016166000  1 7.538359000 -4.583261000 -3.230812000  1 7.196254000 -2.990453000 -3.730329000  6 7.429424000 -4.120662000 -0.639723000  7 7.833763000 -5.446556000 -0.536824000  6 7.730351000 -5.730123000 0.754892000  1 7.942745000 -6.684301000 1.224040000  6 5.498041000 -4.887960000 3.195102000  1 4.861049000 -4.545623000 2.375562000  8 5.125907000 -4.264646000 4.432116000  1 4.375823000 -4.789896000 4.767632000  6 5.523178000 -6.413404000 3.374602000  1 5.576666000 -6.922206000 2.405747000  8 4.413036000 -6.874436000 4.133573000  15 3.442153000 -8.119374000 3.502827000  8 2.839197000 -8.776821000 4.717494000  8 4.200743000 -8.847314000 2.421430000  8 2.280845000 -7.238074000 2.685726000  6 1.450381000 -6.350001000 3.444240000  1 1.928042000 -6.081570000 4.396431000  1 0.485673000 -6.829538000 3.670762000  6 1.188201000 -5.084069000 2.652133000  1 0.581689000 -4.406785000 3.272829000  8 2.459460000 -4.453779000 2.352937000  6 2.400083000 -3.793718000 1.097910000  1 2.735134000 -2.757926000 1.197733000  7 3.321603000 -4.457782000 0.157636000  6 3.651848000 -3.911720000 -1.067463000  7 3.367165000 -2.650352000 -1.464479000  6 3.703173000 -2.430927000 -2.730747000  7 3.539463000 -1.184134000 -3.263979000  1 2.863749000 -0.605925000 -2.779968000  1 3.519772000 -1.123939000 -4.276388000  7 4.278602000 -3.384017000 -3.541873000  1 4.239324000 -3.209647000 -4.549537000  6 4.611100000 -4.718357000 -3.161287000  8 5.107415000 -5.492325000 -3.994650000  6 4.272513000 -4.949681000 -1.784546000  7 4.321064000 -6.112674000 -1.017366000  6 3.732348000 -5.793285000 0.124998000  1 3.556796000 -6.450222000 0.968259000  6 0.959537000 -3.922292000 0.582481000  1 0.938308000 -4.010789000 -0.509411000  8 0.186450000 -2.795771000 0.996173000  1 -0.678990000 -2.930471000 0.535408000  6 0.522296000 -5.232653000 1.278423000  1 0.954755000 -6.084300000 0.737058000  8 -0.884691000 -5.401098000 1.424850000  15 -1.818712000 -5.479296000 0.037908000  8 -3.150234000 -6.052377000 0.446525000  8 -0.987095000 -5.996888000 -1.115032000  8 -1.976792000 -3.806394000 -0.215330000  6 -3.267061000 -3.196890000 -0.334304000  1 -3.221382000 -2.238626000 0.200173000  1 -4.036685000 -3.839955000 0.112218000  6 -3.621689000 -2.947993000 -1.787205000  1 -4.614704000 -2.473337000 -1.842278000  8 -2.630562000 -2.067186000 -2.376314000  6 -2.507026000 -2.346602000 -3.778441000  1 -2.772553000 -1.466605000 -4.375410000  7 -1.105761000 -2.631974000 -4.071936000  6 -0.385457000 -2.044047000 -5.093478000  7 -0.861953000 -1.128020000 -5.965027000  6 0.050663000 -0.722571000 -6.842064000  7 -0.268022000 0.244905000 -7.746964000  1 -1.259812000 0.423380000 -7.844652000  1 0.267919000 0.315849000 -8.602318000  7 1.346004000 -1.198009000 -6.843965000  1 2.017760000 -0.820076000 -7.506301000  6 1.896018000 -2.145189000 -5.935867000  8 3.112769000 -2.413828000 -6.027422000  6 0.902995000 -2.609573000 -5.014917000  7 0.968088000 -3.530552000 -3.971199000  6 -0.239350000 -3.524101000 -3.431486000  1 -0.561047000 -4.128879000 -2.587272000  6 -3.464486000 -3.513111000 -4.100446000  1 -3.005994000 -4.204881000 -4.824506000  8 -4.674724000 -2.966581000 -4.589083000  1 -5.250580000 -3.765549000 -4.754990000  6 -3.637039000 -4.168540000 -2.707054000  1 -2.784360000 -4.829141000 -2.495928000  8 -4.861388000 -4.853611000 -2.506822000  15 -5.311829000 -6.059218000 -3.576256000  8 -4.242624000 -7.120530000 -3.646743000  8 -5.857152000 -5.385238000 -4.830256000  8 -6.634922000 -6.550439000 -2.712571000  6 -6.413055000 -7.326698000 -1.517695000  1 -5.378488000 -7.227527000 -1.165715000  1 -6.603972000 -8.385763000 -1.744172000  6 -7.360617000 -6.909860000 -0.409139000  1 -7.315422000 -7.687360000 0.370671000  8 -6.951046000 -5.644172000 0.183731000  6 -8.093547000 -4.895974000 0.562582000  1 -8.000814000 -4.551880000 1.598265000  7 -8.189148000 -3.665957000 -0.284900000  6 -7.478823000 -3.549251000 -1.449439000  1 -6.911658000 -4.424291000 -1.738294000  6 -8.952946000 -2.602787000 0.221982000  8 -9.594866000 -2.705382000 1.273197000  7 -8.913459000 -1.450148000 -0.532375000  1 -9.420752000 -0.631962000 -0.099587000  6 -8.134893000 -1.227103000 -1.665678000  8 -8.046418000 -0.081633000 -2.166731000  6 -7.444478000 -2.390460000 -2.165894000  1 -6.833491000 -2.317129000 -3.059767000  6 -9.312190000 -5.804790000 0.366876000  1 -10.223693000 -5.240623000 0.150219000  8 -9.473053000 -6.599814000 1.542768000  1 -9.720314000 -7.482474000 1.211203000  6 -8.827250000 -6.681897000 -0.798686000  1 -8.890168000 -6.140145000 -1.749248000  8 -9.525103000 -7.921894000 -0.882311000  15 -10.785021000 -8.018681000 -2.016969000  8 -11.197923000 -9.467697000 -1.998012000  8 -10.405034000 -7.253439000 -3.259175000  8 -11.945427000 -7.073521000 -1.266910000  6 -12.544635000 -7.592767000 -0.075932000  1 -11.773312000 -7.922531000 0.633989000  1 -13.186315000 -8.456128000 -0.307790000  6 -13.394892000 -6.526925000 0.581276000  1 -13.856541000 -6.971944000 1.478533000  8 -12.575250000 -5.401415000 0.977346000  6 -13.291131000 -4.188480000 0.793124000  1 -13.272553000 -3.588164000 1.711391000  7 -12.651696000 -3.381843000 -0.250353000  6 -12.766759000 -2.006707000 -0.321256000  7 -13.445330000 -1.230116000 0.549633000  6 -13.415587000 0.065654000 0.206640000  7 -14.065596000 0.966530000 0.968353000  1 -14.460390000 0.628321000 1.833059000  1 -14.042138000 1.978542000 0.775800000  7 -12.755571000 0.537474000 -0.905250000  1 -12.800962000 1.562507000 -1.097726000  6 -12.032425000 -0.246123000 -1.823553000  8 -11.469478000 0.283453000 -2.808884000  6 -12.064683000 -1.634972000 -1.480445000  7 -11.530731000 -2.757259000 -2.111124000  6 -11.907669000 -3.782896000 -1.363421000  1 -11.698693000 -4.830148000 -1.547632000  6 -14.730832000 -4.564833000 0.411940000  1 -15.188648000 -3.808349000 -0.238417000  8 -15.484470000 -4.744432000 1.609544000  1 -16.048928000 -5.525269000 1.444022000  6 -14.514232000 -5.923194000 -0.279731000  1 -14.196447000 -5.798059000 -1.321526000  8 -15.671939000 -6.752238000 -0.193597000  15 -16.766094000 -6.755832000 -1.467762000  8 -17.801758000 -7.791938000 -1.098753000  8 -16.034548000 -6.754727000 -2.788078000  8 -17.404152000 -5.223810000 -1.173229000  6 -18.621788000 -4.924748000 -1.879155000  1 -19.185592000 -5.849315000 -2.066591000  1 -18.391894000 -4.444321000 -2.843204000  6 -19.486525000 -3.974423000 -1.066163000  1 -20.338000000 -3.675006000 -1.689342000  8 -20.012771000 -4.635648000 0.126947000  6 -19.521777000 -4.015210000 1.298889000  1 -20.305073000 -3.454499000 1.823144000  7 -19.072409000 -5.068571000 2.242969000  6 -18.455556000 -6.202415000 1.768366000  1 -18.466797000 -6.320314000 0.689576000  6 -19.181210000 -4.804937000 3.616686000  8 -19.742956000 -3.812089000 4.077939000  7 -18.594464000 -5.776414000 4.416431000  1 -18.656799000 -5.602149000 5.415055000  6 -17.911684000 -6.952773000 4.024359000  8 -17.427146000 -7.701937000 4.879175000  6 -17.883969000 -7.130267000 2.586401000  1 -17.411613000 -8.011167000 2.166289000  6 -18.358635000 -3.076107000 0.867025000  1 -17.428767000 -3.652674000 0.866326000  8 -18.169304000 -1.946929000 1.694053000  1 -18.832622000 -1.305358000 1.372929000  6 -18.764302000 -2.721417000 -0.568888000  1 -17.900923000 -2.462624000 -1.189980000  8 -19.668018000 -1.606780000 -0.466526000  15 -19.747047000 -0.624615000 -1.840123000  8 -20.667899000 0.509905000 -1.467191000  8 -19.922258000 -1.486955000 -3.066334000  8 -18.159657000 -0.118919000 -1.887676000  6 -17.695410000 0.836212000 -0.915426000  1 -17.226627000 0.302587000 -0.075044000  1 -18.535208000 1.436662000 -0.540651000  6 -16.697475000 1.760280000 -1.589868000  1 -16.439118000 2.572574000 -0.902509000  8 -15.467323000 1.038020000 -1.895767000  6 -15.251668000 0.948179000 -3.284525000  1 -14.402418000 1.564591000 -3.603021000  7 -14.866325000 -0.455852000 -3.598520000  6 -15.442899000 -1.498961000 -2.909411000  1 -16.180080000 -1.189852000 -2.176721000  6 -13.931300000 -0.676640000 -4.618537000  8 -13.443555000 0.219249000 -5.305514000  7 -13.609562000 -2.016259000 -4.793436000  1 -12.904997000 -2.195979000 -5.501635000  6 -14.121987000 -3.147316000 -4.118520000  8 -13.717137000 -4.278604000 -4.411258000  6 -15.125423000 -2.805138000 -3.130898000  1 -15.611159000 -3.602036000 -2.576828000  6 -16.571134000 1.388700000 -3.977151000  1 -17.222028000 0.505902000 -4.023663000  8 -16.434592000 1.880093000 -5.280128000  1 -15.760088000 2.611905000 -5.287115000  6 -17.205843000 2.333556000 -2.923054000  1 -18.301813000 2.290386000 -2.985572000  8 -16.744068000 3.674113000 -3.125612000  15 -17.764085000 4.853874000 -2.478905000  8 -17.998960000 4.544033000 -1.015471000  8 -18.906522000 5.107539000 -3.438406000  8 -16.660337000 6.096547000 -2.540729000  6 -16.755322000 7.110282000 -3.549342000  1 -17.695208000 7.674233000 -3.449945000  1 -16.736184000 6.667230000 -4.556044000  6 -15.577418000 8.049854000 -3.308356000  1 -15.613603000 8.390785000 -2.273891000  8 -14.301325000 7.364913000 -3.444305000  6 -13.852666000 7.451656000 -4.772458000  1 -12.786849000 7.713459000 -4.793602000  7 -13.992558000 6.110189000 -5.388966000  6 -13.564382000 5.714215000 -6.643184000  7 -12.923250000 6.489214000 -7.551744000  6 -12.641450000 5.839439000 -8.675987000  7 -12.057761000 6.514905000 -9.711733000  1 -11.651411000 7.404627000 -9.448826000  1 -11.518404000 5.989801000 -10.388704000  7 -12.966639000 4.515252000 -8.885301000  1 -12.745367000 4.081816000 -9.777056000  6 -13.636328000 3.648244000 -7.959829000  8 -13.877272000 2.477793000 -8.274891000  6 -13.928986000 4.359350000 -6.748058000  7 -14.557040000 3.939736000 -5.581320000  6 -14.569250000 5.001386000 -4.788314000  1 -14.986968000 5.039314000 -3.793273000  6 -14.703466000 8.561448000 -5.487627000  1 -15.419049000 8.087935000 -6.171876000  8 -13.940586000 9.490760000 -6.223921000  1 -13.670632000 10.149427000 -5.552673000  6 -15.443445000 9.209294000 -4.287729000  1 -16.395971000 9.680029000 -4.559357000  8 -14.522660000 10.190354000 -3.780011000  15 -15.024509000 11.298180000 -2.603746000  8 -16.474325000 11.643645000 -2.837894000  8 -13.906341000 12.308604000 -2.545926000  8 -15.016963000 10.351511000 -1.225460000  6 -13.759190000 9.863653000 -0.726414000  1 -13.189214000 10.679197000 -0.256028000  1 -13.159404000 9.441366000 -1.545889000  6 -14.042898000 8.794716000 0.312520000  1 -14.771529000 9.181068000 1.043686000  8 -14.598576000 7.625665000 -0.342030000  6 -14.172351000 6.438923000 0.305150000  1 -15.028542000 5.830908000 0.609366000  7 -13.392353000 5.588148000 -0.639661000  6 -12.599989000 6.124462000 -1.609366000  1 -12.596319000 7.204088000 -1.675389000  6 -13.449275000 4.182721000 -0.455313000  8 -14.085517000 3.745354000 0.532392000  7 -12.823431000 3.383685000 -1.356185000  6 -12.080127000 3.913387000 -2.351529000  7 -11.537590000 3.066890000 -3.244060000  1 -10.889329000 3.433721000 -3.926613000  1 -11.530586000 2.047842000 -3.058586000  6 -11.904167000 5.329133000 -2.473803000  1 -11.289598000 5.762063000 -3.256447000  6 -13.328008000 6.863847000 1.523521000  1 -12.510473000 6.159605000 1.678634000  8 -14.142320000 6.958489000 2.681638000  1 -13.724787000 6.363207000 3.333865000  6 -12.819252000 8.253417000 1.061688000  1 -11.983806000 8.108388000 0.360523000  8 -12.421536000 9.134392000 2.099076000  15 -10.809626000 9.106616000 2.582968000  8 -10.641647000 10.334392000 3.441640000  8 -9.923944000 8.756614000 1.411790000  8 -10.783099000 7.732231000 3.546066000  6 -11.581518000 7.725313000 4.732124000  1 -12.587470000 8.110230000 4.514339000  1 -11.125278000 8.352446000 5.513686000  6 -11.686216000 6.309485000 5.263090000  1 -12.284124000 6.329526000 6.187050000  8 -12.370582000 5.464006000 4.288088000  6 -11.768992000 4.170833000 4.238021000  1 -12.541922000 3.395518000 4.292877000  7 -11.070200000 3.990166000 2.954718000  6 -10.843691000 2.765654000 2.340700000  7 -11.098610000 1.530578000 2.824796000  6 -10.688152000 0.578813000 1.967928000  1 -10.822823000 -0.458394000 2.275088000  7 -10.115117000 0.737333000 0.757390000  6 -9.889479000 1.991986000 0.276547000  7 -9.370623000 2.140878000 -0.954397000  1 -8.988506000 3.048357000 -1.183134000  1 -8.965482000 1.321765000 -1.427730000  6 -10.239165000 3.082662000 1.110862000  7 -10.068034000 4.453066000 0.975492000  6 -10.554763000 4.963021000 2.098948000  1 -10.552419000 6.009917000 2.381347000  6 -10.801525000 4.101177000 5.420252000  1 -9.976768000 3.408885000 5.229484000  8 -11.528037000 3.715817000 6.587946000  1 -11.170130000 4.284105000 7.296184000  6 -10.366570000 5.572228000 5.541811000  1 -9.613626000 5.833292000 4.789383000  8 -9.896769000 5.869596000 6.854637000  15 -8.212592000 5.947224000 7.079588000  8 -8.048161000 6.326589000 8.527654000  8 -7.598237000 6.685454000 5.917736000  8 -7.776688000 4.348899000 6.837398000  6 -8.177428000 3.366903000 7.798193000  1 -9.248801000 3.461941000 8.022901000  1 -7.615712000 3.486807000 8.736950000  6 -7.916494000 1.973958000 7.254570000  1 -8.266688000 1.253184000 8.011244000  8 -8.674341000 1.768512000 6.032754000  6 -7.903933000 1.048983000 5.088128000  1 -8.502013000 0.232444000 4.665168000  7 -7.529973000 1.928926000 3.962305000  6 -7.121439000 1.455814000 2.733329000  7 -6.862973000 0.158725000 2.438615000  6 -6.457731000 -0.006906000 1.187359000  7 -6.110958000 -1.256061000 0.750035000  1 -6.015583000 -1.935449000 1.495122000  1 -5.381002000 -1.301551000 0.052090000  7 -6.394535000 1.020089000 0.271618000  1 -6.250147000 0.795630000 -0.710045000  6 -6.634379000 2.407026000 0.538953000  8 -6.550611000 3.232169000 -0.380193000  6 -6.968296000 2.593979000 1.922374000  7 -7.242400000 3.747680000 2.649187000  6 -7.570561000 3.320597000 3.859907000  1 -7.809933000 3.933821000 4.719033000  6 -6.669637000 0.522614000 5.825428000  1 -5.809075000 0.385271000 5.161328000  8 -7.025744000 -0.703239000 6.467930000  1 -6.641332000 -0.624139000 7.362894000  6 -6.464230000 1.617848000 6.893269000  1 -5.928519000 2.481739000 6.481732000  8 -5.799219000 1.102681000 8.047543000  15 -4.099628000 1.196771000 8.070497000  8 -3.699530000 0.513721000 9.352134000  8 -3.686445000 2.592619000 7.676521000  8 -3.719602000 0.256178000 6.752037000  6 -3.719019000 -1.173320000 6.821510000  1 -4.746160000 -1.551196000 6.913874000  1 -3.132397000 -1.518430000 7.684918000  6 -3.087867000 -1.720874000 5.554761000  1 -3.066019000 -2.820713000 5.627175000  8 -3.868959000 -1.337456000 4.392736000  6 -3.004501000 -0.966491000 3.322982000  1 -3.289679000 -1.494758000 2.407058000  7 -3.174283000 0.467105000 3.033644000  6 -3.263762000 1.032511000 1.772051000  7 -3.187669000 0.376572000 0.586074000  6 -3.339431000 1.184338000 -0.464824000  7 -3.411430000 0.659768000 -1.719213000  1 -3.052958000 -0.290061000 -1.850729000  1 -3.267541000 1.280451000 -2.505195000  7 -3.497364000 2.547666000 -0.342470000  1 -3.645642000 3.107887000 -1.175625000  6 -3.571113000 3.285982000 0.872889000  8 -3.697321000 4.520305000 0.837689000  6 -3.464708000 2.407129000 1.997819000  7 -3.502446000 2.682426000 3.360924000  6 -3.326250000 1.510486000 3.949778000  1 -3.334849000 1.322716000 5.016706000  6 -1.564449000 -1.372145000 3.741482000  1 -0.819060000 -0.704489000 3.300577000  8 -1.339233000 -2.725370000 3.382329000  1 -0.704842000 -2.734889000 2.632089000  6 -1.663534000 -1.221595000 5.272440000  1 -1.585111000 -0.161052000 5.537237000  8 -0.730480000 -1.982461000 6.024240000  15 0.273210000 -1.230136000 7.141032000  8 0.438523000 -2.227001000 8.263453000  8 -0.134677000 0.208934000 7.358561000  8 1.699211000 -1.127075000 6.265563000  6 2.201709000 -2.314514000 5.644672000  1 1.418110000 -3.078279000 5.562834000  1 3.030099000 -2.731941000 6.240256000  6 2.719392000 -1.988366000 4.255939000  1 3.081240000 -2.916809000 3.795878000  8 1.629958000 -1.487059000 3.449551000  6 2.061218000 -0.427037000 2.616218000  1 1.815864000 -0.641598000 1.569102000  7 1.310586000 0.789101000 2.949961000  6 0.804217000 1.676860000 2.013611000  7 0.914213000 1.617277000 0.672749000  6 0.275318000 2.650488000 0.089143000  1 0.315384000 2.676843000 -1.002463000  7 -0.413386000 3.654804000 0.665633000  6 -0.525764000 3.670299000 2.019313000  7 -1.237581000 4.681447000 2.605781000  1 -1.610831000 4.428681000 3.516594000  1 -1.965457000 5.055571000 1.994994000  6 0.118061000 2.652989000 2.764245000  7 0.189083000 2.368738000 4.125907000  6 0.908554000 1.255597000 4.198553000  1 1.138050000 0.704328000 5.103634000  6 3.587827000 -0.297258000 2.789642000  1 3.912308000 0.754551000 2.735021000  8 4.180200000 -1.078655000 1.767096000  1 5.031950000 -1.438266000 2.094403000  1 3.661224000 -0.158151000 4.956991000  6 3.826120000 -0.923423000 4.183954000  8 5.142690000 -1.446430000 4.318965000  1 5.082915000 -2.425934000 4.373174000  11 12.284679000 -2.028377000 -2.995907000 |
| **H3** | 1 -0.227922000 -0.014567000 -4.842458000  8 -0.051840000 -0.907614000 -4.464787000  6 -0.126603000 -1.784859000 -5.581827000  1 0.357437000 -2.728149000 -5.296583000  1 0.423893000 -1.374425000 -6.446784000  6 -1.555761000 -2.075868000 -6.058062000  1 -1.524213000 -2.442486000 -7.095749000  8 -2.192157000 -3.113284000 -5.249053000  6 -3.514071000 -2.746072000 -4.922267000  1 -4.189920000 -3.594247000 -5.086724000  7 -3.587557000 -2.389846000 -3.463242000  6 -2.484962000 -2.510415000 -2.648347000  1 -1.586352000 -2.871002000 -3.134906000  6 -4.845941000 -2.039100000 -2.947299000  8 -5.870204000 -2.026342000 -3.642114000  7 -4.849075000 -1.720161000 -1.608687000  1 -5.692977000 -1.201410000 -1.257632000  6 -3.779594000 -1.824489000 -0.715731000  8 -3.957365000 -1.584135000 0.494660000  6 -2.536579000 -2.250263000 -1.310987000  1 -1.660504000 -2.370317000 -0.682703000  6 -3.881329000 -1.552455000 -5.818124000  1 -4.621952000 -0.898973000 -5.348102000  8 -4.348664000 -2.075199000 -7.062182000  1 -4.099807000 -1.405648000 -7.723702000  6 -2.516005000 -0.882033000 -5.975498000  1 -2.314033000 -0.277274000 -5.086034000  8 -2.486858000 -0.082142000 -7.160416000  15 -1.483560000 1.256351000 -7.255772000  8 -0.792151000 1.206662000 -8.591135000  8 -0.689826000 1.387537000 -5.952696000  8 -2.592790000 2.478246000 -7.203397000  6 -3.374722000 2.747238000 -8.383865000  1 -3.840057000 1.821927000 -8.754026000  1 -2.727839000 3.166951000 -9.164800000  6 -4.456610000 3.767550000 -8.056226000  1 -4.912415000 4.089018000 -9.004464000  8 -5.513681000 3.178240000 -7.247839000  6 -5.658442000 3.873226000 -6.027066000  1 -6.727384000 3.974208000 -5.796694000  7 -5.032428000 3.088455000 -4.944234000  6 -5.022810000 3.404324000 -3.593262000  7 -5.543776000 4.512219000 -3.016244000  6 -5.416771000 4.500572000 -1.684485000  7 -5.960149000 5.508026000 -0.950803000  1 -6.206658000 6.335855000 -1.476224000  1 -5.681902000 5.632827000 0.033237000  7 -4.800031000 3.492267000 -0.983150000  1 -4.758849000 3.551980000 0.064928000  6 -4.262230000 2.321437000 -1.550549000  8 -3.749417000 1.439213000 -0.833930000  6 -4.407026000 2.305062000 -2.970456000  7 -4.073959000 1.326159000 -3.902142000  6 -4.473058000 1.820922000 -5.062101000  1 -4.369994000 1.350169000 -6.025768000  6 -5.008747000 5.256254000 -6.192370000  1 -4.549712000 5.601786000 -5.256755000  8 -6.012280000 6.158704000 -6.641095000  1 -5.532796000 6.751822000 -7.251731000  6 -3.948503000 5.011307000 -7.294179000  1 -2.972475000 4.820979000 -6.847996000  8 -3.903492000 6.170771000 -8.119236000  15 -2.623831000 6.429600000 -9.179525000  8 -3.178203000 6.453567000 -10.580657000  8 -1.473067000 5.511210000 -8.801949000  8 -2.294128000 8.002227000 -8.784659000  6 -1.512838000 8.267173000 -7.602493000  1 -0.667612000 8.902695000 -7.905412000  1 -1.127471000 7.341820000 -7.162133000  6 -2.271895000 9.036891000 -6.534361000  1 -2.854358000 9.852650000 -6.990339000  8 -3.176734000 8.206624000 -5.760920000  6 -3.058692000 8.487542000 -4.380834000  1 -4.036003000 8.778103000 -3.969996000  7 -2.667400000 7.268646000 -3.637250000  6 -1.947098000 6.152634000 -4.052665000  7 -1.343742000 5.955909000 -5.241884000  6 -0.821183000 4.734643000 -5.374526000  7 -0.186953000 4.406556000 -6.530961000  1 -0.581701000 4.866935000 -7.369398000  1 -0.004555000 3.412215000 -6.638800000  7 -0.861542000 3.781951000 -4.375384000  1 -0.639945000 2.815936000 -4.649989000  6 -1.328851000 4.000861000 -3.062738000  8 -1.196102000 3.114940000 -2.191422000  6 -1.945426000 5.285883000 -2.942634000  7 -2.604799000 5.865525000 -1.870278000  6 -3.030432000 7.034706000 -2.316102000  1 -3.606288000 7.756555000 -1.749247000  6 -2.066193000 9.657164000 -4.200043000  1 -1.415661000 9.505569000 -3.325097000  8 -2.814141000 10.862231000 -4.092963000  1 -2.291009000 11.508182000 -4.608740000  6 -1.281652000 9.639023000 -5.525672000  1 -0.390325000 9.005201000 -5.439422000  8 -0.938545000 10.963799000 -5.933197000  15 0.697859000 11.353860000 -6.065190000  8 0.708391000 12.793923000 -6.511254000  8 1.420422000 10.251733000 -6.806857000  8 1.077452000 11.273371000 -4.435441000  6 2.233995000 10.510812000 -4.072338000  1 3.153054000 10.969132000 -4.473331000  1 2.166733000 9.489200000 -4.472493000  6 2.370708000 10.468869000 -2.566586000  1 2.359412000 11.487053000 -2.147954000  8 1.287976000 9.705014000 -1.976278000  6 1.771792000 8.924414000 -0.894723000  1 1.248125000 9.172389000 0.038202000  7 1.497177000 7.508986000 -1.184866000  6 1.581312000 6.493155000 -0.248205000  7 1.820790000 6.620978000 1.072990000  6 1.877062000 5.419115000 1.667714000  1 2.075124000 5.395058000 2.739699000  7 1.717997000 4.208889000 1.100554000  6 1.476444000 4.093812000 -0.232762000  7 1.364374000 2.859227000 -0.772966000  1 0.764547000 2.783830000 -1.592414000  1 1.351805000 2.066227000 -0.119376000  6 1.403698000 5.300422000 -0.978382000  7 1.228195000 5.575211000 -2.330533000  6 1.291382000 6.896979000 -2.414935000  1 1.177143000 7.481175000 -3.318392000  6 3.286693000 9.181406000 -0.742664000  1 3.814089000 8.238860000 -0.546842000  8 3.496038000 10.116423000 0.297927000  1 4.470950000 10.208516000 0.334545000  6 3.656589000 9.757210000 -2.132586000  1 3.892555000 8.930789000 -2.818880000  8 4.723159000 10.698979000 -2.111032000  15 6.288624000 10.119014000 -2.024182000  8 7.179237000 11.293897000 -2.341703000  8 6.402985000 8.784001000 -2.723510000  8 6.296816000 9.853637000 -0.346950000  6 7.219753000 8.838518000 0.084670000  1 8.249402000 9.085864000 -0.217079000  1 6.962259000 7.877135000 -0.380578000  6 7.218017000 8.710974000 1.592922000  1 7.462080000 9.673790000 2.063987000  8 5.920670000 8.283082000 2.099304000  6 6.072478000 7.143894000 2.928364000  1 5.428839000 7.245789000 3.811128000  7 5.644351000 5.943324000 2.176436000  6 5.607386000 4.643161000 2.649076000  7 5.947004000 4.222429000 3.893586000  6 5.834025000 2.904873000 4.035299000  7 6.202092000 2.311492000 5.215021000  1 6.323259000 2.957860000 5.985040000  1 5.761250000 1.433418000 5.468035000  7 5.416033000 2.060288000 3.033840000  1 5.451463000 1.034270000 3.197136000  6 5.018061000 2.465866000 1.741971000  8 4.629702000 1.621593000 0.908543000  6 5.131069000 3.876887000 1.569318000  7 4.873047000 4.677322000 0.465590000  6 5.184806000 5.897601000 0.860445000  1 5.104220000 6.799328000 0.272931000  6 7.557902000 7.043536000 3.304094000  1 7.867504000 6.006964000 3.492270000  8 7.801914000 7.878443000 4.431837000  1 8.685926000 8.257399000 4.267064000  6 8.220649000 7.635270000 2.049355000  1 8.308790000 6.864768000 1.275274000  8 9.490951000 8.196791000 2.363656000  15 10.820926000 7.673842000 1.449849000  8 11.985542000 8.456158000 1.997764000  8 10.431702000 7.621734000 -0.006168000  8 10.897762000 6.073088000 1.930079000  6 11.227228000 5.796011000 3.296691000  1 10.610825000 6.408172000 3.971738000  1 12.285358000 6.027202000 3.491934000  6 10.994002000 4.328408000 3.599210000  1 11.323912000 4.144822000 4.634966000  8 9.580516000 4.014483000 3.496946000  6 9.405019000 2.735836000 2.904726000  1 8.704228000 2.139869000 3.502875000  7 8.813623000 2.895423000 1.563951000  6 8.168425000 1.893427000 0.866107000  7 8.113202000 0.590073000 1.231469000  6 7.422391000 -0.148001000 0.367704000  7 7.309771000 -1.490528000 0.557457000  1 7.995508000 -1.925973000 1.169749000  1 7.149771000 -2.037020000 -0.278460000  7 6.769692000 0.379454000 -0.725513000  1 6.082618000 -0.201739000 -1.203050000  6 6.807668000 1.740440000 -1.154793000  8 6.157599000 2.093583000 -2.147439000  6 7.659119000 2.510997000 -0.289531000  7 8.022257000 3.854631000 -0.324314000  6 8.709506000 4.051529000 0.791292000  1 9.200638000 4.963070000 1.103926000  6 10.788256000 2.078686000 2.830630000  1 10.862874000 1.368563000 1.999485000  8 11.059440000 1.436233000 4.077337000  1 11.989415000 1.665685000 4.268140000  6 11.694827000 3.314942000 2.678995000  1 11.711643000 3.672615000 1.643209000  8 13.017309000 3.058582000 3.152725000  15 14.184642000 2.632541000 1.995425000  8 15.451041000 2.440631000 2.788743000  8 14.049053000 3.545614000 0.803404000  8 13.591196000 1.159057000 1.475018000  6 13.758729000 -0.003323000 2.297187000  1 13.215455000 0.115620000 3.245576000  1 14.823542000 -0.163247000 2.519324000  6 13.221395000 -1.220436000 1.566855000  1 13.459530000 -2.114201000 2.166664000  8 11.781183000 -1.117285000 1.425170000  6 11.374841000 -1.610402000 0.150872000  1 10.567236000 -2.343310000 0.266092000  7 10.836415000 -0.502116000 -0.649754000  6 9.871020000 -0.621601000 -1.636460000  7 9.107836000 -1.685055000 -1.945366000  6 8.292323000 -1.426367000 -2.987294000  1 7.644448000 -2.240846000 -3.315294000  7 8.157285000 -0.274814000 -3.682622000  6 8.909540000 0.789462000 -3.318814000  7 8.770727000 1.965117000 -4.011559000  1 9.070102000 2.790416000 -3.503822000  1 7.865207000 2.071893000 -4.456040000  6 9.834933000 0.641304000 -2.263202000  7 10.750952000 1.513841000 -1.684423000  6 11.336696000 0.793578000 -0.736243000  1 12.111295000 1.137807000 -0.059446000  6 12.612579000 -2.224593000 -0.522609000  1 12.570796000 -2.103456000 -1.611522000  8 12.685483000 -3.594350000 -0.144235000  1 13.632733000 -3.825859000 -0.155576000  6 13.754263000 -1.431948000 0.141814000  1 13.915640000 -0.470306000 -0.357600000  8 14.973668000 -2.179262000 0.182938000  15 16.066650000 -1.965809000 -1.070504000  8 17.231539000 -2.875009000 -0.750719000  8 16.242764000 -0.497558000 -1.384782000  8 15.118874000 -2.681855000 -2.255557000  6 15.621019000 -2.625942000 -3.593808000  1 16.721219000 -2.606799000 -3.604686000  1 15.254382000 -1.718798000 -4.100292000  6 15.143778000 -3.835113000 -4.380346000  1 15.417518000 -3.686118000 -5.437111000  8 15.800655000 -5.043619000 -3.908225000  6 14.858027000 -6.085882000 -3.753136000  1 15.259202000 -7.012979000 -4.183017000  7 14.628803000 -6.318627000 -2.308144000  6 13.829248000 -7.308534000 -1.768469000  7 13.068192000 -8.188297000 -2.468129000  6 12.391916000 -9.020397000 -1.686923000  7 11.600658000 -9.989397000 -2.252989000  1 11.460524000 -9.851085000 -3.247202000  1 10.752343000 -10.249631000 -1.759605000  7 12.489379000 -9.008754000 -0.313490000  1 11.979714000 -9.700962000 0.227197000  6 13.262709000 -8.094959000 0.476817000  8 13.258240000 -8.195757000 1.708360000  6 13.967354000 -7.178292000 -0.376041000  7 14.839647000 -6.140412000 -0.064022000  6 15.220414000 -5.652951000 -1.238022000  1 15.906782000 -4.829603000 -1.390686000  6 13.566989000 -5.648575000 -4.462968000  1 12.672941000 -6.096291000 -4.013416000  8 13.668521000 -5.993010000 -5.844506000  1 13.349360000 -5.196007000 -6.311411000  6 13.635346000 -4.119670000 -4.297191000  1 13.237920000 -3.812712000 -3.324199000  8 12.962266000 -3.455950000 -5.368202000  15 11.490329000 -2.694720000 -4.997579000  8 11.056429000 -2.044303000 -6.285452000  8 11.657727000 -1.964553000 -3.692349000  8 10.535799000 -4.040400000 -4.678183000  6 10.182982000 -4.826885000 -5.826121000  1 11.091266000 -5.189747000 -6.329184000  1 9.611824000 -4.220814000 -6.544019000  6 9.339775000 -6.024884000 -5.445779000  1 9.087730000 -6.548660000 -6.383280000  8 10.099892000 -6.925694000 -4.598173000  6 9.235676000 -7.562379000 -3.672790000  1 9.343923000 -8.653230000 -3.729187000  7 9.611447000 -7.159035000 -2.307337000  6 9.263751000 -7.846759000 -1.161234000  7 8.504593000 -8.972316000 -1.107718000  6 8.299647000 -9.392594000 0.138318000  7 7.542064000 -10.510497000 0.356488000  1 6.979764000 -10.783582000 -0.441139000  1 7.060402000 -10.584162000 1.244855000  7 8.862792000 -8.783608000 1.237316000  1 8.636276000 -9.128520000 2.164921000  6 9.665111000 -7.593614000 1.238671000  8 10.066844000 -7.131441000 2.310875000  6 9.845100000 -7.123834000 -0.105921000  7 10.524616000 -6.014899000 -0.589267000  6 10.364550000 -6.056520000 -1.902378000  1 10.738374000 -5.342336000 -2.625962000  6 7.799720000 -7.139197000 -4.008260000  1 7.169004000 -7.095205000 -3.115255000  8 7.261939000 -8.055927000 -4.961196000  1 6.802110000 -7.483949000 -5.607478000  6 8.027526000 -5.770233000 -4.686323000  1 8.145849000 -4.964465000 -3.951883000  8 6.980971000 -5.482403000 -5.614130000  15 5.681944000 -4.522138000 -5.082126000  8 4.677252000 -4.595749000 -6.200529000  8 6.232274000 -3.237680000 -4.517488000  8 5.164847000 -5.389792000 -3.756251000  6 4.269183000 -6.492863000 -3.955513000  1 4.745299000 -7.261946000 -4.580608000  1 3.347016000 -6.152580000 -4.444439000  6 3.911218000 -7.089273000 -2.610681000  1 3.234820000 -7.943249000 -2.778355000  8 5.121781000 -7.564189000 -1.956130000  6 5.022359000 -7.386661000 -0.558434000  1 5.194660000 -8.329591000 -0.033143000  7 6.090858000 -6.456336000 -0.081262000  6 6.618930000 -5.463553000 -0.871855000  1 6.249495000 -5.447940000 -1.894885000  6 6.492083000 -6.630456000 1.250006000  8 6.104669000 -7.566682000 1.951053000  7 7.358832000 -5.655453000 1.705137000  1 7.580053000 -5.705950000 2.695078000  6 7.942127000 -4.604726000 0.981799000  8 8.696651000 -3.809198000 1.570249000  6 7.544292000 -4.573731000 -0.408033000  1 8.004252000 -3.844062000 -1.068181000  6 3.603052000 -6.848129000 -0.251512000  1 3.623248000 -6.150501000 0.594540000  8 2.744033000 -7.930575000 0.010693000  1 2.250388000 -7.686834000 0.846790000  6 3.255846000 -6.146699000 -1.591278000  1 3.747384000 -5.167100000 -1.660483000  8 1.858725000 -6.033843000 -1.808721000  15 1.180877000 -4.542275000 -2.200336000  8 -0.226750000 -4.888986000 -2.629686000  8 2.130684000 -3.738837000 -3.058104000  8 1.167671000 -3.773367000 -0.717030000  6 0.528143000 -4.448697000 0.385766000  1 1.040267000 -5.399380000 0.586271000  1 -0.527175000 -4.657151000 0.159391000  6 0.614987000 -3.592693000 1.636755000  1 1.600217000 -3.103022000 1.666555000  8 -0.425155000 -2.586676000 1.664902000  6 -0.857929000 -2.361654000 3.006140000  1 -0.616945000 -1.340254000 3.328074000  7 -2.317373000 -2.458241000 3.079252000  6 -3.140296000 -3.492379000 2.677433000  7 -2.764931000 -4.627065000 2.042835000  6 -3.788737000 -5.413406000 1.725178000  7 -3.557646000 -6.550200000 1.001677000  1 -2.586269000 -6.836307000 0.977148000  1 -4.223025000 -7.309898000 1.070313000  7 -5.096790000 -5.112890000 2.034942000  1 -5.857795000 -5.755262000 1.779407000  6 -5.532977000 -3.962647000 2.743014000  8 -6.746272000 -3.829509000 3.000076000  6 -4.438261000 -3.101742000 3.065096000  7 -4.415432000 -1.853112000 3.683711000  6 -3.137367000 -1.503528000 3.671037000  1 -2.703586000 -0.582267000 4.051026000  6 -0.135843000 -3.373638000 3.933936000  1 -0.854578000 -3.851986000 4.616764000  8 0.858391000 -2.680265000 4.646766000  1 1.065220000 -3.218729000 5.464333000  6 0.442163000 -4.403709000 2.928262000  1 -0.282967000 -5.210695000 2.765844000  8 1.708733000 -4.903115000 3.352038000  15 1.964988000 -6.541272000 3.468780000  8 1.486060000 -7.196508000 2.177258000  8 1.506739000 -7.095619000 4.795703000  8 3.618822000 -6.430437000 3.404173000  6 4.424767000 -6.633935000 4.574147000  1 5.226934000 -7.320427000 4.280157000  1 3.826690000 -7.084133000 5.377683000  6 5.049046000 -5.353693000 5.110143000  1 5.700470000 -5.641199000 5.951880000  8 5.881057000 -4.730854000 4.088879000  6 5.762582000 -3.325725000 4.132718000  1 6.757257000 -2.866279000 4.124873000  7 5.061097000 -2.835221000 2.897392000  6 4.532986000 -3.705216000 1.970764000  1 4.472862000 -4.737031000 2.300475000  6 5.100932000 -1.463776000 2.657587000  8 5.585592000 -0.682000000 3.501556000  7 4.574356000 -1.053181000 1.464384000  1 4.618229000 -0.026637000 1.264294000  6 4.249953000 -1.885969000 0.381811000  8 4.116774000 -1.412027000 -0.758253000  6 4.115590000 -3.278910000 0.748307000  1 3.685348000 -3.961223000 0.029753000  6 4.982085000 -2.976210000 5.404097000  1 4.395424000 -2.061485000 5.288509000  8 5.914251000 -2.854123000 6.477057000  1 5.495972000 -3.337569000 7.215609000  6 4.113203000 -4.238339000 5.600245000  1 3.193837000 -4.181995000 5.006339000  8 3.831692000 -4.457814000 6.990700000  15 2.477493000 -3.712670000 7.666048000  8 2.556623000 -3.989997000 9.140861000  8 1.260650000 -4.074020000 6.832381000  8 2.780821000 -2.115231000 7.321461000  6 3.444407000 -1.297097000 8.301640000  1 4.533513000 -1.414706000 8.207864000  1 3.137396000 -1.603194000 9.309327000  6 3.069156000 0.158038000 8.101436000  1 3.399780000 0.722829000 8.989288000  8 3.736620000 0.694103000 6.923439000  6 2.928489000 1.711350000 6.341443000  1 3.455272000 2.669819000 6.320840000  7 2.649319000 1.355405000 4.932064000  6 2.474571000 0.048997000 4.536954000  1 2.621264000 -0.697255000 5.309996000  6 2.379497000 2.423229000 4.062222000  8 2.421339000 3.600739000 4.447484000  7 2.082568000 2.057121000 2.775023000  1 1.919726000 2.864290000 2.105741000  6 1.961116000 0.757845000 2.272360000  8 1.708507000 0.565622000 1.069682000  6 2.147822000 -0.278976000 3.260142000  1 2.005166000 -1.316267000 2.994701000  6 1.628886000 1.824269000 7.169491000  1 0.778573000 1.983870000 6.507014000  8 1.718340000 2.879029000 8.117915000  1 1.007791000 3.495122000 7.860335000  6 1.585871000 0.434722000 7.834024000  1 1.225418000 -0.294328000 7.097463000  8 0.814054000 0.336679000 9.020352000  15 -0.794185000 -0.133335000 8.849328000  8 -1.287635000 -0.337045000 10.259984000  8 -0.928977000 -1.159021000 7.748770000  8 -1.465048000 1.254357000 8.194433000  6 -1.486663000 2.443141000 8.990000000  1 -0.506212000 2.604516000 9.458738000  1 -2.249112000 2.367945000 9.781151000  6 -1.840210000 3.624111000 8.108830000  1 -1.913321000 4.525803000 8.736405000  8 -0.796589000 3.836721000 7.109613000  6 -1.381720000 4.218926000 5.865482000  1 -0.878665000 5.107150000 5.466668000  7 -1.219568000 3.146944000 4.869922000  6 -1.284895000 3.356082000 3.498185000  7 -1.440476000 4.552432000 2.889139000  6 -1.540177000 4.444131000 1.557950000  7 -1.685323000 5.567149000 0.818185000  1 -1.878396000 6.400424000 1.357640000  1 -2.067659000 5.542639000 -0.142298000  7 -1.467875000 3.241963000 0.886927000  1 -1.518324000 3.235593000 -0.138595000  6 -1.285655000 1.960023000 1.490043000  8 -1.213368000 0.944810000 0.791615000  6 -1.221209000 2.079451000 2.922740000  7 -1.151520000 1.104027000 3.912401000  6 -1.173584000 1.764171000 5.061863000  1 -1.177182000 1.327962000 6.053923000  6 -2.870289000 4.475188000 6.131444000  1 -3.447758000 4.231613000 5.235772000  8 -3.057698000 5.827523000 6.518525000  1 -4.028218000 5.935144000 6.572927000  6 -3.130896000 3.489871000 7.288822000  1 -3.228428000 2.470759000 6.899046000  8 -4.262165000 3.825427000 8.078703000  15 -5.521271000 2.718864000 8.254112000  8 -5.858949000 2.701602000 9.724934000  8 -5.250479000 1.477390000 7.438147000  8 -6.728950000 3.532365000 7.412187000  6 -7.038057000 4.877766000 7.794380000  1 -6.276341000 5.284069000 8.471942000  1 -8.008680000 4.904148000 8.313901000  6 -7.145904000 5.770369000 6.571105000  1 -7.402474000 6.786542000 6.910344000  8 -5.870553000 5.820991000 5.869112000  6 -6.077900000 5.871252000 4.459884000  1 -5.500508000 6.689789000 4.022213000  7 -5.593518000 4.617786000 3.824104000  6 -5.500280000 3.441866000 4.520036000  1 -5.792189000 3.481019000 5.563979000  6 -5.267528000 4.674249000 2.445785000  8 -5.421975000 5.765552000 1.846601000  7 -4.811495000 3.549480000 1.846215000  6 -4.683088000 2.389427000 2.534892000  7 -4.217775000 1.328696000 1.876465000  1 -4.163152000 0.427798000 2.330274000  1 -4.004852000 1.382534000 0.863088000  6 -5.035961000 2.309522000 3.922681000  1 -4.952385000 1.387787000 4.488824000  6 -7.585096000 6.046097000 4.241765000  1 -7.910281000 5.598656000 3.296467000  8 -7.908209000 7.436616000 4.281698000  1 -8.737458000 7.477881000 4.796034000  6 -8.154425000 5.347475000 5.491823000  1 -8.160553000 4.257056000 5.377957000  8 -9.456370000 5.831760000 5.813661000  15 -10.773893000 4.842821000 5.389947000  8 -11.985053000 5.623062000 5.828777000  8 -10.469587000 3.423647000 5.795648000  8 -10.643356000 4.847021000 3.722746000  6 -10.974468000 6.040551000 3.003223000  1 -10.357839000 6.882347000 3.349567000  1 -12.033138000 6.298977000 3.152168000  6 -10.731392000 5.825927000 1.521786000  1 -11.032035000 6.745334000 0.993531000  8 -9.318698000 5.586987000 1.276184000  6 -9.157750000 4.589285000 0.276648000  1 -8.429191000 4.923965000 -0.472223000  7 -8.608866000 3.361783000 0.886881000  6 -8.017019000 2.324140000 0.183339000  7 -7.844959000 2.212884000 -1.150876000  6 -7.198970000 1.075340000 -1.463355000  1 -7.015354000 0.894200000 -2.523114000  7 -6.737116000 0.123464000 -0.631346000  6 -6.924132000 0.251789000 0.710886000  7 -6.456280000 -0.723999000 1.517034000  1 -6.474731000 -0.564347000 2.515267000  1 -5.644402000 -1.248299000 1.166658000  6 -7.627958000 1.392285000 1.166603000  7 -7.988131000 1.828248000 2.435001000  6 -8.575471000 3.000594000 2.230318000  1 -9.031939000 3.630806000 2.982676000  6 -10.541134000 4.322554000 -0.322786000  1 -10.637806000 3.296834000 -0.690755000  8 -10.784831000 5.263170000 -1.370846000  1 -11.721049000 5.514539000 -1.254254000  6 -11.454987000 4.632851000 0.875703000  1 -11.501382000 3.791176000 1.575605000  8 -12.761672000 5.012656000 0.439774000  15 -14.025976000 3.903848000 0.661750000  8 -15.239846000 4.604200000 0.109554000  8 -13.925507000 3.317038000 2.046758000  8 -13.556843000 2.658294000 -0.356636000  6 -13.600216000 2.850750000 -1.773699000  1 -13.144613000 3.813902000 -2.046580000  1 -14.640681000 2.847739000 -2.132293000  6 -12.840364000 1.743654000 -2.484937000  1 -12.940830000 1.927048000 -3.567259000  8 -11.429167000 1.809489000 -2.134434000  6 -10.897363000 0.503392000 -1.980600000  1 -9.924797000 0.437076000 -2.480155000  7 -10.686774000 0.200851000 -0.553343000  6 -9.951684000 -0.869809000 -0.079211000  7 -9.220549000 -1.733060000 -0.826347000  6 -8.580134000 -2.629445000 -0.083334000  7 -7.706341000 -3.508624000 -0.681881000  1 -7.708441000 -3.427653000 -1.693212000  1 -7.759954000 -4.470448000 -0.355792000  7 -8.697602000 -2.715997000 1.282689000  1 -8.015922000 -3.283278000 1.807029000  6 -9.451391000 -1.832031000 2.109022000  8 -9.439385000 -1.957917000 3.341123000  6 -10.142003000 -0.859402000 1.311716000  7 -10.980232000 0.183557000 1.688593000  6 -11.294589000 0.791332000 0.554669000  1 -11.967471000 1.629944000 0.436768000  6 -11.935735000 -0.455495000 -2.561426000  1 -11.866632000 -1.459788000 -2.128734000  8 -11.781581000 -0.492206000 -3.982748000  1 -12.693135000 -0.441899000 -4.329009000  6 -13.240763000 0.283342000 -2.201222000  1 -13.496950000 0.144292000 -1.144594000  8 -14.319723000 -0.125718000 -3.040431000  15 -15.275739000 -1.416236000 -2.467555000  8 -16.266587000 -1.668010000 -3.575076000  8 -15.651939000 -1.146407000 -1.032294000  8 -14.148648000 -2.647759000 -2.394858000  6 -13.680688000 -3.215583000 -3.628149000  1 -13.096683000 -2.477248000 -4.191689000  1 -14.526855000 -3.546597000 -4.247563000  6 -12.814867000 -4.420999000 -3.336229000  1 -12.479998000 -4.842667000 -4.298697000  8 -11.657900000 -4.047807000 -2.546429000  6 -11.286431000 -5.132032000 -1.705830000  1 -10.257246000 -5.455429000 -1.905593000  7 -11.322265000 -4.696457000 -0.302155000  6 -10.508020000 -5.191317000 0.698692000  7 -9.388686000 -5.943100000 0.525972000  6 -8.781469000 -6.236925000 1.667619000  7 -7.587899000 -6.939356000 1.631475000  1 -7.469251000 -7.430719000 0.749775000  1 -7.413071000 -7.546785000 2.427120000  7 -9.241785000 -5.830317000 2.891629000  1 -8.714378000 -6.067827000 3.725939000  6 -10.453120000 -5.114981000 3.137962000  8 -10.828629000 -4.926910000 4.303528000  6 -11.078259000 -4.744295000 1.900453000  7 -12.219902000 -3.990933000 1.651445000  6 -12.341244000 -3.985380000 0.331668000  1 -13.101849000 -3.471359000 -0.245908000  6 -12.285855000 -6.285869000 -1.940386000  1 -12.524929000 -6.771964000 -0.986589000  8 -11.720195000 -7.200428000 -2.864629000  1 -12.455025000 -7.797887000 -3.115566000  6 -13.494715000 -5.540027000 -2.541193000  1 -14.129161000 -5.125392000 -1.749671000  8 -14.245878000 -6.382309000 -3.397498000  15 -15.925407000 -6.310318000 -3.412728000  8 -16.341991000 -5.834890000 -4.785103000  8 -16.445181000 -5.699974000 -2.133592000  8 -16.214961000 -7.959814000 -3.273721000  6 -15.603741000 -8.835763000 -4.228806000  1 -14.950983000 -8.287282000 -4.919354000  1 -16.389561000 -9.320039000 -4.831158000  6 -14.801927000 -9.923967000 -3.529920000  1 -14.429287000 -10.630900000 -4.288859000  8 -13.666994000 -9.340655000 -2.836530000  6 -13.469204000 -9.961248000 -1.569448000  1 -12.426447000 -10.280898000 -1.460300000  7 -13.707329000 -8.996310000 -0.493739000  6 -12.877068000 -8.811608000 0.605013000  7 -11.712118000 -9.426824000 0.878990000  6 -11.176246000 -8.960321000 2.024931000  1 -10.217414000 -9.397438000 2.312648000  7 -11.657361000 -8.023825000 2.866168000  6 -12.832286000 -7.416805000 2.557209000  7 -13.338501000 -6.487520000 3.416635000  1 -14.011046000 -5.847829000 3.009173000  1 -12.650747000 -6.035026000 4.018333000  6 -13.500175000 -7.804589000 1.369211000  7 -14.669888000 -7.366870000 0.754614000  6 -14.762867000 -8.098056000 -0.347339000  1 -15.521632000 -7.992062000 -1.112988000  6 -14.420413000 -11.169434000 -1.519659000  1 -14.756501000 -11.374449000 -0.491332000  8 -13.738479000 -12.283062000 -2.084246000  1 -14.422449000 -12.771821000 -2.579174000  1 -16.258744000 -10.059781000 -1.913087000  6 -15.562203000 -10.720958000 -2.447879000  8 -16.221697000 -11.886909000 -2.930230000  1 -16.846181000 -11.626188000 -3.624337000 | 1 -0.381682000 -0.327701000 -4.837562000  8 -0.240394000 -1.197607000 -4.395697000  6 -0.365208000 -2.148823000 -5.445327000  1 0.079092000 -3.088849000 -5.092777000  1 0.194388000 -1.826306000 -6.341349000  6 -1.812442000 -2.406468000 -5.889068000  1 -1.812673000 -2.825783000 -6.907043000  8 -2.473500000 -3.377779000 -5.019339000  6 -3.771147000 -2.942583000 -4.682640000  1 -4.484069000 -3.769122000 -4.791035000  7 -3.797801000 -2.514510000 -3.241432000  6 -2.680898000 -2.630008000 -2.444981000  1 -1.809137000 -3.049099000 -2.933123000  6 -5.031059000 -2.098833000 -2.716038000  8 -6.073261000 -2.097782000 -3.386555000  7 -4.990300000 -1.702340000 -1.400620000  1 -5.804374000 -1.129204000 -1.060979000  6 -3.905720000 -1.795318000 -0.525339000  8 -4.050651000 -1.480391000 0.672564000  6 -2.692708000 -2.299315000 -1.122117000  1 -1.808064000 -2.423543000 -0.506741000  6 -4.112377000 -1.778818000 -5.626726000  1 -4.812375000 -1.072180000 -5.170722000  8 -4.633745000 -2.340162000 -6.831851000  1 -4.369058000 -1.717542000 -7.531982000  6 -2.725673000 -1.173304000 -5.850805000  1 -2.478518000 -0.530718000 -5.000495000  8 -2.699736000 -0.437366000 -7.077578000  15 -1.627477000 0.832316000 -7.287883000  8 -0.990092000 0.663159000 -8.639964000  8 -0.781096000 0.996513000 -6.021455000  8 -2.657307000 2.121363000 -7.269489000  6 -3.445712000 2.399172000 -8.442564000  1 -3.974516000 1.493301000 -8.773492000  1 -2.791530000 2.754038000 -9.249002000  6 -4.457536000 3.491617000 -8.123095000  1 -4.914538000 3.815789000 -9.069844000  8 -5.528449000 2.984356000 -7.276809000  6 -5.618204000 3.731562000 -6.081365000  1 -6.677075000 3.885642000 -5.834799000  7 -4.999832000 2.965377000 -4.980657000  6 -4.939631000 3.333957000 -3.643661000  7 -5.387050000 4.489350000 -3.099157000  6 -5.239648000 4.517221000 -1.769083000  7 -5.720544000 5.574162000 -1.065039000  1 -5.938773000 6.396338000 -1.610832000  1 -5.457054000 5.703837000 -0.077720000  7 -4.661468000 3.503681000 -1.040957000  1 -4.623371000 3.587197000 0.005616000  6 -4.199180000 2.285567000 -1.574746000  8 -3.722515000 1.400598000 -0.837017000  6 -4.372254000 2.226706000 -2.990177000  7 -4.119557000 1.193298000 -3.887228000  6 -4.518296000 1.662683000 -5.057588000  1 -4.465793000 1.147437000 -6.002476000  6 -4.915689000 5.075971000 -6.321437000  1 -4.449251000 5.462163000 -5.404839000  8 -5.883095000 5.984925000 -6.834093000  1 -5.389258000 6.498344000 -7.502912000  6 -3.865288000 4.722807000 -7.400995000  1 -2.908204000 4.478175000 -6.938422000  8 -3.735118000 5.853408000 -8.256616000  15 -2.464898000 5.974379000 -9.354409000  8 -3.049891000 5.988745000 -10.743004000  8 -1.378705000 4.986171000 -8.963403000  8 -2.009263000 7.530759000 -9.022615000  6 -1.142515000 7.762271000 -7.894397000  1 -0.239429000 8.267893000 -8.268701000  1 -0.853152000 6.822518000 -7.414565000  6 -1.747044000 8.677640000 -6.843797000  1 -2.223302000 9.549774000 -7.318642000  8 -2.731465000 8.018549000 -6.006758000  6 -2.523841000 8.336588000 -4.644354000  1 -3.434223000 8.780145000 -4.216873000  7 -2.282585000 7.105644000 -3.859543000  6 -1.677479000 5.909156000 -4.232098000  7 -1.109366000 5.608921000 -5.417090000  6 -0.702758000 4.340365000 -5.503623000  7 -0.121865000 3.903879000 -6.651692000  1 -0.494149000 4.355058000 -7.504717000  1 -0.037987000 2.890985000 -6.710636000  7 -0.811147000 3.440466000 -4.460887000  1 -0.657356000 2.451318000 -4.688567000  6 -1.238538000 3.755397000 -3.152596000  8 -1.158610000 2.904563000 -2.242539000  6 -1.749960000 5.090661000 -3.088543000  7 -2.347440000 5.771911000 -2.040131000  6 -2.661275000 6.958711000 -2.530076000  1 -3.163168000 7.753477000 -1.990926000  6 -1.370673000 9.360436000 -4.550074000  1 -0.718645000 9.162339000 -3.686523000  8 -1.938773000 10.664003000 -4.486691000  1 -1.347645000 11.203065000 -5.049450000  6 -0.643887000 9.169241000 -5.894911000  1 0.148053000 8.415502000 -5.804680000  8 -0.123924000 10.405152000 -6.386091000  15 1.552555000 10.577614000 -6.473031000  8 1.762076000 11.947452000 -7.066707000  8 2.160652000 9.320412000 -7.052883000  8 1.850631000 10.637459000 -4.825507000  6 2.883759000 9.793397000 -4.307974000  1 3.877541000 10.130077000 -4.647757000  1 2.746415000 8.756640000 -4.645553000  6 2.873908000 9.856291000 -2.796713000  1 2.921585000 10.901238000 -2.454603000  8 1.665408000 9.247002000 -2.268202000  6 1.964372000 8.494769000 -1.104617000  1 1.345871000 8.819781000 -0.258061000  7 1.654776000 7.078262000 -1.362447000  6 1.665599000 6.094131000 -0.387636000  7 1.832541000 6.262536000 0.939830000  6 1.860660000 5.079739000 1.574041000  1 2.010253000 5.089722000 2.653575000  7 1.728427000 3.851801000 1.038212000  6 1.546460000 3.697811000 -0.300125000  7 1.446026000 2.448456000 -0.810258000  1 0.876992000 2.361363000 -1.648617000  1 1.392227000 1.674830000 -0.137659000  6 1.521356000 4.879072000 -1.087224000  7 1.438217000 5.108035000 -2.456844000  6 1.524340000 6.425092000 -2.582206000  1 1.476006000 6.977890000 -3.510821000  6 3.462193000 8.674672000 -0.785235000  1 3.889641000 7.727870000 -0.433725000  8 3.608992000 9.704512000 0.171946000  1 4.574751000 9.743138000 0.335301000  6 4.029874000 9.069008000 -2.170300000  1 4.243384000 8.160103000 -2.751159000  8 5.185350000 9.904083000 -2.120711000  15 6.649352000 9.240097000 -1.702914000  8 7.705604000 10.243534000 -2.084624000  8 6.728611000 7.783542000 -2.129289000  8 6.418003000 9.275932000 -0.019284000  6 7.270150000 8.332206000 0.652034000  1 8.327865000 8.547134000 0.439576000  1 7.043357000 7.325247000 0.280727000  6 7.126271000 8.330309000 2.157156000  1 7.358622000 9.311111000 2.593012000  8 5.786598000 7.953862000 2.589335000  6 5.849842000 6.788582000 3.394491000  1 5.108010000 6.868209000 4.198064000  7 5.522761000 5.607599000 2.558021000  6 5.541568000 4.275226000 2.937296000  7 5.853684000 3.786302000 4.162810000  6 5.822687000 2.458016000 4.204604000  7 6.185849000 1.802725000 5.353107000  1 6.242661000 2.400065000 6.168944000  1 5.770545000 0.894465000 5.531859000  7 5.503216000 1.664784000 3.128100000  1 5.614340000 0.635555000 3.226933000  6 5.137575000 2.133591000 1.846710000  8 4.856015000 1.327373000 0.936457000  6 5.161039000 3.560959000 1.784838000  7 4.906485000 4.424106000 0.728312000  6 5.124980000 5.628929000 1.221203000  1 5.010057000 6.564334000 0.697546000  6 7.288234000 6.692298000 3.925972000  1 7.571333000 5.659420000 4.171355000  8 7.411186000 7.559140000 5.047614000  1 8.333513000 7.871664000 5.023808000  6 8.062943000 7.240462000 2.722346000  1 8.158022000 6.446678000 1.975521000  8 9.341110000 7.742250000 3.096322000  15 10.675416000 7.259104000 2.174759000  8 11.826511000 8.068833000 2.703202000  8 10.281560000 7.199077000 0.711941000  8 10.801394000 5.655465000 2.609385000  6 11.080075000 5.268734000 3.961405000  1 10.440020000 5.825208000 4.661266000  1 12.131937000 5.478440000 4.207287000  6 10.838834000 3.774397000 4.123588000  1 11.192211000 3.486761000 5.126665000  8 9.418386000 3.468079000 4.031867000  6 9.218378000 2.288517000 3.263714000  1 8.498387000 1.634415000 3.768481000  7 8.659060000 2.664882000 1.949451000  6 8.126323000 1.821827000 0.989293000  7 8.014032000 0.476629000 1.075330000  6 7.514518000 -0.062270000 -0.035080000  7 7.395359000 -1.407445000 -0.162830000  1 7.798003000 -1.980221000 0.583176000  1 7.548040000 -1.760962000 -1.103856000  7 7.078826000 0.688538000 -1.110595000  1 6.638747000 0.209367000 -1.889624000  6 7.166608000 2.099665000 -1.244808000  8 6.728551000 2.666347000 -2.257288000  6 7.801731000 2.660824000 -0.090825000  7 8.156102000 3.973102000 0.182944000  6 8.665589000 3.937264000 1.404354000  1 9.128347000 4.753585000 1.933153000  6 10.590411000 1.628289000 3.090709000  1 10.650171000 1.032955000 2.171681000  8 10.867519000 0.828635000 4.240853000  1 11.798778000 1.029455000 4.456205000  6 11.510165000 2.864164000 3.080118000  1 11.514469000 3.342233000 2.094600000  8 12.836952000 2.535562000 3.489401000  15 13.969973000 2.274687000 2.249909000  8 15.246470000 1.928985000 2.971031000  8 13.835669000 3.372695000 1.224228000  8 13.323268000 0.924469000 1.510641000  6 13.406564000 -0.352266000 2.158526000  1 12.809451000 -0.352299000 3.081049000  1 14.450387000 -0.587415000 2.412357000  6 12.878987000 -1.419913000 1.217986000  1 12.994166000 -2.402000000 1.705171000  8 11.473252000 -1.185974000 0.949415000  6 11.175453000 -1.473949000 -0.410934000  1 10.337085000 -2.177107000 -0.479231000  7 10.746991000 -0.244802000 -1.096642000  6 9.895625000 -0.212641000 -2.189901000  7 9.095774000 -1.188452000 -2.656112000  6 8.400482000 -0.789065000 -3.739832000  1 7.712939000 -1.530176000 -4.150446000  7 8.418126000 0.417102000 -4.348090000  6 9.209586000 1.390132000 -3.832919000  7 9.248957000 2.602543000 -4.461828000  1 9.603528000 3.380227000 -3.918680000  1 8.451146000 2.815620000 -5.048891000  6 9.999280000 1.098068000 -2.696929000  7 10.882781000 1.857805000 -1.933647000  6 11.317277000 1.020488000 -0.998129000  1 12.018664000 1.253674000 -0.203742000  6 12.445208000 -2.050391000 -1.061899000  1 12.522958000 -1.744588000 -2.110900000  8 12.386670000 -3.468779000 -0.945142000  1 13.311272000 -3.771060000 -0.992523000  6 13.555002000 -1.473769000 -0.158600000  1 13.840768000 -0.468668000 -0.489814000  8 14.708877000 -2.315842000 -0.084819000  15 15.882176000 -2.163763000 -1.272400000  8 17.006966000 -3.082854000 -0.855044000  8 16.103645000 -0.713505000 -1.636408000  8 14.983070000 -2.912110000 -2.475884000  6 15.515537000 -2.890291000 -3.802590000  1 16.613339000 -2.966302000 -3.788428000  1 15.237390000 -1.953006000 -4.309754000  6 14.944510000 -4.052452000 -4.596432000  1 15.245936000 -3.935431000 -5.649503000  8 15.482059000 -5.310848000 -4.105544000  6 14.451663000 -6.270187000 -3.973208000  1 14.766880000 -7.220532000 -4.423678000  7 14.207470000 -6.519113000 -2.533832000  6 13.369979000 -7.489260000 -2.017540000  7 12.597095000 -8.343592000 -2.737976000  6 11.875202000 -9.151520000 -1.973428000  7 11.050494000 -10.090955000 -2.544667000  1 10.954648000 -9.969255000 -3.546820000  1 10.161566000 -10.249085000 -2.074623000  7 11.937455000 -9.141841000 -0.597327000  1 11.392526000 -9.819277000 -0.072862000  6 12.720852000 -8.257345000 0.213389000  8 12.675671000 -8.353956000 1.444499000  6 13.480243000 -7.367361000 -0.620824000  7 14.371222000 -6.355377000 -0.283800000  6 14.791060000 -5.873366000 -1.446554000  1 15.505081000 -5.071634000 -1.584139000  6 13.201043000 -5.712976000 -4.671965000  1 12.273553000 -6.061521000 -4.203393000  8 13.247594000 -6.085083000 -6.049649000  1 12.998148000 -5.266078000 -6.521876000  6 13.414245000 -4.192573000 -4.533452000  1 13.031443000 -3.831779000 -3.573287000  8 12.823065000 -3.496977000 -5.633944000  15 11.475592000 -2.514981000 -5.306739000  8 11.041715000 -1.995938000 -6.653850000  8 11.812153000 -1.626213000 -4.139196000  8 10.400290000 -3.683192000 -4.757344000  6 9.890008000 -4.538398000 -5.790833000  1 10.718444000 -4.952741000 -6.384056000  1 9.238000000 -3.971552000 -6.471506000  6 9.086051000 -5.698451000 -5.249529000  1 8.749588000 -6.269290000 -6.132106000  8 9.915096000 -6.558077000 -4.424074000  6 9.097014000 -7.245293000 -3.490875000  1 9.218429000 -8.330085000 -3.590060000  7 9.502712000 -6.884233000 -2.122577000  6 9.154567000 -7.604565000 -0.996897000  7 8.474522000 -8.781377000 -0.984240000  6 8.251560000 -9.231617000 0.249300000  7 7.576481000 -10.406899000 0.422038000  1 7.059239000 -10.710639000 -0.394404000  1 7.094744000 -10.553715000 1.300616000  7 8.725063000 -8.596412000 1.375050000  1 8.471443000 -8.956505000 2.289977000  6 9.442235000 -7.352851000 1.419329000  8 9.758642000 -6.871533000 2.511185000  6 9.645441000 -6.858771000 0.087161000  7 10.273668000 -5.704876000 -0.358089000  6 10.169020000 -5.740248000 -1.677584000  1 10.550647000 -5.002882000 -2.371532000  6 7.644980000 -6.824265000 -3.756541000  1 7.050141000 -6.829952000 -2.839412000  8 7.077991000 -7.702762000 -4.727905000  1 6.564004000 -7.114074000 -5.313996000  6 7.848867000 -5.426965000 -4.380359000  1 8.054963000 -4.665549000 -3.618672000  8 6.738336000 -5.052047000 -5.193344000  15 5.493720000 -4.158690000 -4.454496000  8 4.518866000 -3.880178000 -5.566287000  8 6.116815000 -3.106341000 -3.565881000  8 4.883323000 -5.273854000 -3.379934000  6 4.005479000 -6.343299000 -3.747425000  1 4.504183000 -7.023420000 -4.452296000  1 3.088718000 -5.945099000 -4.201272000  6 3.626090000 -7.102322000 -2.483520000  1 2.970538000 -7.942392000 -2.763183000  8 4.825475000 -7.636487000 -1.846572000  6 4.739926000 -7.511141000 -0.440858000  1 4.961401000 -8.464184000 0.047234000  7 5.771992000 -6.544260000 0.048692000  6 6.223231000 -5.521861000 -0.742131000  1 5.842173000 -5.518545000 -1.758010000  6 6.190891000 -6.680657000 1.376023000  8 5.876892000 -7.637365000 2.086009000  7 6.990798000 -5.638637000 1.817745000  1 7.203259000 -5.669749000 2.811060000  6 7.451900000 -4.526113000 1.095675000  8 8.090160000 -3.629247000 1.680528000  6 7.072952000 -4.559625000 -0.293186000  1 7.429731000 -3.811336000 -0.985246000  6 3.312403000 -7.028719000 -0.093202000  1 3.333216000 -6.361105000 0.776143000  8 2.483794000 -8.138730000 0.155366000  1 1.963046000 -7.893046000 0.971240000  6 2.924972000 -6.274987000 -1.394383000  1 3.358056000 -5.266369000 -1.395615000  8 1.520758000 -6.229851000 -1.594381000  15 0.785039000 -4.774810000 -2.013236000  8 -0.626829000 -5.177305000 -2.371664000  8 1.677280000 -3.977059000 -2.935880000  8 0.813751000 -3.956177000 -0.557272000  6 0.199179000 -4.602609000 0.577957000  1 0.693268000 -5.564326000 0.771473000  1 -0.868962000 -4.781488000 0.393364000  6 0.358187000 -3.734283000 1.810707000  1 1.364822000 -3.291843000 1.813101000  8 -0.626565000 -2.675603000 1.839318000  6 -1.027820000 -2.410633000 3.181541000  1 -0.742188000 -1.393215000 3.479574000  7 -2.490066000 -2.449918000 3.272881000  6 -3.353335000 -3.449040000 2.868239000  7 -3.020736000 -4.601313000 2.241638000  6 -4.073779000 -5.340859000 1.909333000  7 -3.882411000 -6.486654000 1.188043000  1 -2.924155000 -6.814683000 1.177401000  1 -4.580360000 -7.217323000 1.246212000  7 -5.371214000 -4.981437000 2.199668000  1 -6.155560000 -5.590255000 1.931118000  6 -5.765770000 -3.809877000 2.898054000  8 -6.976004000 -3.616905000 3.129400000  6 -4.637210000 -3.000401000 3.239113000  7 -4.566132000 -1.750436000 3.851593000  6 -3.273977000 -1.456802000 3.849686000  1 -2.808402000 -0.551410000 4.226803000  6 -0.333379000 -3.436628000 4.114249000  1 -1.057007000 -3.865037000 4.824520000  8 0.710708000 -2.777921000 4.786955000  1 0.901951000 -3.310183000 5.611333000  6 0.174252000 -4.515031000 3.119873000  1 -0.591180000 -5.289860000 2.988780000  8 1.424320000 -5.064667000 3.535591000  15 1.639498000 -6.711219000 3.619516000  8 1.185504000 -7.330286000 2.301706000  8 1.139708000 -7.282093000 4.924461000  8 3.298602000 -6.638433000 3.595037000  6 4.081237000 -6.870989000 4.775218000  1 4.845899000 -7.607712000 4.502393000  1 3.450784000 -7.274248000 5.578970000  6 4.776497000 -5.621695000 5.300553000  1 5.399901000 -5.936352000 6.153671000  8 5.652622000 -5.062116000 4.282459000  6 5.620620000 -3.650236000 4.304480000  1 6.639510000 -3.250345000 4.313413000  7 4.987783000 -3.141857000 3.045374000  6 4.246484000 -3.957069000 2.219968000  1 4.036603000 -4.945406000 2.616405000  6 5.235161000 -1.812694000 2.714477000  8 5.836799000 -1.061115000 3.511392000  7 4.779907000 -1.408675000 1.491213000  1 4.908729000 -0.403661000 1.250973000  6 4.142653000 -2.210257000 0.518568000  8 3.913878000 -1.766015000 -0.611038000  6 3.810218000 -3.533638000 1.004188000  1 3.195415000 -4.165776000 0.378753000  6 4.838121000 -3.234801000 5.555085000  1 4.301640000 -2.294030000 5.403405000  8 5.754396000 -3.131236000 6.642039000  1 5.301874000 -3.580922000 7.382087000  6 3.901534000 -4.447752000 5.767588000  1 2.985890000 -4.354597000 5.172103000  8 3.608221000 -4.632729000 7.160286000  15 2.277620000 -3.832000000 7.818206000  8 2.344656000 -4.081309000 9.298443000  8 1.052657000 -4.170970000 6.987002000  8 2.638021000 -2.253977000 7.440815000  6 3.303826000 -1.431840000 8.415823000  1 4.390638000 -1.586600000 8.352460000  1 2.963065000 -1.701342000 9.423003000  6 2.980034000 0.028189000 8.167597000  1 3.300522000 0.605940000 9.050537000  8 3.702012000 0.513685000 6.999551000  6 2.939155000 1.526587000 6.354875000  1 3.494717000 2.467774000 6.306889000  7 2.683921000 1.117231000 4.953365000  6 2.547711000 -0.203408000 4.595553000  1 2.675284000 -0.918220000 5.400904000  6 2.412832000 2.151915000 4.044770000  8 2.416641000 3.339882000 4.396733000  7 2.158838000 1.739886000 2.761671000  1 1.968784000 2.522632000 2.073233000  6 2.067196000 0.422403000 2.298642000  8 1.820182000 0.188467000 1.102649000  6 2.267687000 -0.580011000 3.320262000  1 2.137736000 -1.627593000 3.086450000  6 1.624783000 1.711170000 7.145021000  1 0.798273000 1.883552000 6.456426000  8 1.732910000 2.788625000 8.065567000  1 1.045295000 3.419013000 7.781260000  6 1.515239000 0.343251000 7.846788000  1 1.151365000 -0.393855000 7.119952000  8 0.709961000 0.303336000 9.013967000  15 -0.910480000 -0.110683000 8.817722000  8 -1.437252000 -0.278849000 10.221007000  8 -1.063715000 -1.143878000 7.726852000  8 -1.516493000 1.292741000 8.135157000  6 -1.495255000 2.492993000 8.913284000  1 -0.513265000 2.619815000 9.389370000  1 -2.267749000 2.462074000 9.697618000  6 -1.790018000 3.673648000 8.010951000  1 -1.828811000 4.587904000 8.623199000  8 -0.731120000 3.824347000 7.016698000  6 -1.291326000 4.211443000 5.762681000  1 -0.747042000 5.070430000 5.353634000  7 -1.169029000 3.116448000 4.786889000  6 -1.220745000 3.301705000 3.411560000  7 -1.369059000 4.487223000 2.780750000  6 -1.469128000 4.354893000 1.450961000  7 -1.612711000 5.464426000 0.694352000  1 -1.801964000 6.309823000 1.215085000  1 -1.941776000 5.435365000 -0.285673000  7 -1.399530000 3.140614000 0.802035000  1 -1.446265000 3.113090000 -0.224114000  6 -1.203640000 1.873236000 1.428217000  8 -1.109061000 0.847439000 0.746521000  6 -1.153945000 2.015000000 2.859277000  7 -1.100022000 1.056580000 3.866589000  6 -1.130563000 1.737793000 5.004105000  1 -1.154854000 1.320091000 6.003702000  6 -2.768243000 4.537169000 6.013556000  1 -3.348990000 4.306323000 5.116902000  8 -2.898827000 5.901960000 6.380325000  1 -3.864067000 6.046229000 6.445570000  6 -3.079731000 3.582408000 7.183605000  1 -3.219887000 2.562631000 6.808543000  8 -4.199492000 3.982473000 7.959604000  15 -5.493793000 2.924997000 8.177246000  8 -5.805433000 2.941598000 9.653959000  8 -5.284023000 1.661198000 7.377689000  8 -6.685044000 3.770779000 7.343674000  6 -6.929593000 5.136173000 7.701876000  1 -6.152030000 5.516484000 8.376368000  1 -7.899673000 5.216841000 8.216794000  6 -6.992239000 6.013269000 6.463954000  1 -7.197373000 7.046522000 6.786287000  8 -5.717278000 5.988250000 5.760632000  6 -5.923367000 6.023233000 4.350872000  1 -5.309223000 6.806735000 3.898969000  7 -5.494136000 4.738651000 3.737145000  6 -5.443670000 3.573317000 4.454015000  1 -5.745826000 3.638971000 5.493397000  6 -5.153933000 4.759894000 2.361092000  8 -5.272605000 5.844570000 1.742015000  7 -4.721680000 3.612691000 1.787086000  6 -4.625207000 2.464062000 2.500285000  7 -4.166501000 1.382497000 1.871421000  1 -4.141796000 0.488347000 2.340692000  1 -3.956652000 1.404685000 0.856207000  6 -5.003198000 2.417955000 3.882942000  1 -4.953378000 1.505509000 4.467619000  6 -7.420256000 6.266413000 4.129192000  1 -7.767562000 5.816705000 3.193346000  8 -7.673757000 7.671924000 4.139640000  1 -8.500802000 7.765033000 4.650483000  6 -8.022804000 5.623404000 5.393071000  1 -8.086490000 4.532820000 5.301044000  8 -9.297084000 6.182300000 5.706402000  15 -10.666813000 5.246139000 5.333542000  8 -11.831991000 6.090133000 5.778441000  8 -10.418384000 3.823852000 5.765552000  8 -10.573303000 5.204434000 3.664093000  6 -10.849595000 6.395260000 2.918249000  1 -10.209221000 7.221196000 3.259759000  1 -11.900735000 6.694266000 3.043952000  6 -10.588932000 6.145201000 1.444756000  1 -10.849107000 7.064308000 0.895106000  8 -9.180274000 5.856406000 1.229719000  6 -9.032717000 4.830501000 0.258041000  1 -8.275030000 5.121213000 -0.480597000  7 -8.546266000 3.597258000 0.909252000  6 -7.976307000 2.522172000 0.245480000  7 -7.780250000 2.371650000 -1.081301000  6 -7.170459000 1.203992000 -1.351387000  1 -6.974728000 0.987146000 -2.402045000  7 -6.757730000 0.261071000 -0.484846000  6 -6.965542000 0.429438000 0.849177000  7 -6.542654000 -0.540336000 1.687719000  1 -6.568594000 -0.354780000 2.681210000  1 -5.744318000 -1.098104000 1.361812000  6 -7.642668000 1.602163000 1.260379000  7 -8.018509000 2.081087000 2.508478000  6 -8.559231000 3.267433000 2.261297000  1 -9.014448000 3.929517000 2.986204000  6 -10.410718000 4.601648000 -0.368332000  1 -10.535424000 3.573763000 -0.721422000  8 -10.597754000 5.532018000 -1.436650000  1 -11.527062000 5.817435000 -1.346080000  6 -11.338918000 4.964633000 0.804572000  1 -11.429707000 4.137730000 1.517638000  8 -12.622163000 5.379531000 0.332898000  15 -13.926831000 4.314718000 0.538874000  8 -15.105741000 5.051460000 -0.041010000  8 -13.870843000 3.735588000 1.929603000  8 -13.482648000 3.045210000 -0.460494000  6 -13.489640000 3.224610000 -1.879761000  1 -12.996641000 4.169368000 -2.151800000  1 -14.521694000 3.252005000 -2.261010000  6 -12.751338000 2.085881000 -2.563370000  1 -12.818625000 2.263350000 -3.649252000  8 -11.347786000 2.107027000 -2.178028000  6 -10.864736000 0.785131000 -2.000901000  1 -9.883105000 0.681098000 -2.475564000  7 -10.699271000 0.487167000 -0.566924000  6 -10.010014000 -0.603233000 -0.069462000  7 -9.286412000 -1.491203000 -0.794309000  6 -8.691791000 -2.403032000 -0.031671000  7 -7.826125000 -3.307535000 -0.600442000  1 -7.756395000 -3.207077000 -1.608195000  1 -7.923380000 -4.269940000 -0.287813000  7 -8.849792000 -2.480524000 1.331474000  1 -8.198891000 -3.063646000 1.876497000  6 -9.602300000 -1.574003000 2.134197000  8 -9.632306000 -1.699024000 3.366053000  6 -10.237652000 -0.581445000 1.315742000  7 -11.052536000 0.488785000 1.666465000  6 -11.317540000 1.101265000 0.522351000  1 -11.958976000 1.961229000 0.383838000  6 -11.920190000 -0.142774000 -2.601047000  1 -11.896537000 -1.145749000 -2.160625000  8 -11.730906000 -0.194750000 -4.017686000  1 -12.630830000 -0.116294000 -4.388020000  6 -13.208012000 0.642075000 -2.279077000  1 -13.495135000 0.519504000 -1.228385000  8 -14.279094000 0.262181000 -3.142000000  15 -15.290389000 -0.992946000 -2.585103000  8 -16.266537000 -1.214345000 -3.712013000  8 -15.685792000 -0.705288000 -1.158496000  8 -14.209247000 -2.263322000 -2.485574000  6 -13.732771000 -2.848734000 -3.707198000  1 -13.121982000 -2.127344000 -4.264037000  1 -14.575116000 -3.163573000 -4.340239000  6 -12.900293000 -4.072477000 -3.394001000  1 -12.550889000 -4.501374000 -4.348032000  8 -11.755396000 -3.724309000 -2.575824000  6 -11.431807000 -4.813365000 -1.722142000  1 -10.403840000 -5.157767000 -1.890397000  7 -11.500764000 -4.373826000 -0.320714000  6 -10.731686000 -4.888929000 0.704994000  7 -9.631500000 -5.675329000 0.567119000  6 -9.067377000 -5.983231000 1.726651000  7 -7.896647000 -6.725047000 1.727212000  1 -7.769974000 -7.221758000 0.849503000  1 -7.768158000 -7.338715000 2.527008000  7 -9.548374000 -5.555623000 2.935404000  1 -9.055827000 -5.809855000 3.786013000  6 -10.746165000 -4.806070000 3.144620000  8 -11.149799000 -4.604589000 4.298414000  6 -11.324466000 -4.422471000 1.888397000  7 -12.436946000 -3.638807000 1.603693000  6 -12.518461000 -3.633509000 0.280719000  1 -13.247056000 -3.100661000 -0.320653000  6 -12.446971000 -5.947191000 -1.984206000  1 -12.722219000 -6.429079000 -1.037906000  8 -11.873813000 -6.872083000 -2.893377000  1 -12.611350000 -7.459135000 -3.160230000  6 -13.623702000 -5.176802000 -2.617070000  1 -14.270331000 -4.748744000 -1.842687000  8 -14.369599000 -6.002328000 -3.494294000  15 -16.047322000 -5.905912000 -3.540254000  8 -16.431399000 -5.430261000 -4.921911000  8 -16.579844000 -5.282083000 -2.273041000  8 -16.365760000 -7.550463000 -3.400570000  6 -15.749936000 -8.438467000 -4.341217000  1 -15.072828000 -7.902658000 -5.018088000  1 -16.530937000 -8.909852000 -4.959940000  6 -14.982438000 -9.539585000 -3.624115000  1 -14.600398000 -10.250313000 -4.374904000  8 -13.857704000 -8.974302000 -2.900354000  6 -13.702747000 -9.598841000 -1.629177000  1 -12.668035000 -9.933685000 -1.492622000  7 -13.955985000 -8.631008000 -0.559650000  6 -13.156660000 -8.461818000 0.564236000  7 -12.013330000 -9.099813000 0.874835000  6 -11.504934000 -8.643471000 2.037247000  1 -10.565294000 -9.100424000 2.355848000  7 -11.992998000 -7.696157000 2.862352000  6 -13.144810000 -7.065569000 2.515807000  7 -13.657953000 -6.123162000 3.356577000  1 -14.307953000 -5.473951000 2.928265000  1 -12.980432000 -5.681306000 3.977336000  6 -13.783687000 -7.442586000 1.308659000  7 -14.925357000 -6.983046000 0.658529000  6 -14.998402000 -7.712948000 -0.445735000  1 -15.730939000 -7.593420000 -1.234613000  6 -14.672101000 -10.793444000 -1.606697000  1 -15.038114000 -10.994927000 -0.587923000  8 -13.992544000 -11.916086000 -2.155386000  1 -14.670159000 -12.388563000 -2.674850000  1 -16.484571000 -9.657776000 -2.047718000  6 -15.782427000 -10.327365000 -2.564593000  8 -16.444828000 -11.483418000 -3.067440000  1 -17.009541000 -11.219536000 -3.809959000  19 8.905398000 6.110149000 -1.556324000 | 1 -0.453741000 -0.376057000 -4.912573000  8 -0.324279000 -1.233345000 -4.441946000  6 -0.468608000 -2.211352000 -5.462940000  1 -0.033014000 -3.146840000 -5.089380000  1 0.087239000 -1.919866000 -6.371836000  6 -1.923397000 -2.462472000 -5.888398000  1 -1.939018000 -2.898187000 -6.899352000  8 -2.583773000 -3.413520000 -4.995975000  6 -3.874476000 -2.963352000 -4.652620000  1 -4.596583000 -3.783489000 -4.750421000  7 -3.885890000 -2.528767000 -3.213184000  6 -2.768829000 -2.668212000 -2.420340000  1 -1.902863000 -3.095857000 -2.912115000  6 -5.110915000 -2.099479000 -2.680025000  8 -6.156713000 -2.081215000 -3.345369000  7 -5.059473000 -1.711034000 -1.362904000  1 -5.861511000 -1.125015000 -1.016340000  6 -3.977191000 -1.835183000 -0.489081000  8 -4.117090000 -1.533918000 0.713187000  6 -2.773211000 -2.351712000 -1.093812000  1 -1.887620000 -2.492606000 -0.483830000  6 -4.214162000 -1.804790000 -5.603893000  1 -4.896802000 -1.082714000 -5.145516000  8 -4.762796000 -2.373510000 -6.793504000  1 -4.497995000 -1.764372000 -7.505482000  6 -2.824185000 -1.219730000 -5.859944000  1 -2.556280000 -0.563271000 -5.026662000  8 -2.813726000 -0.508052000 -7.102012000  15 -1.718918000 0.730485000 -7.367002000  8 -1.134589000 0.527976000 -8.737935000  8 -0.825432000 0.885171000 -6.131695000  8 -2.710764000 2.047342000 -7.325936000  6 -3.515360000 2.356478000 -8.480011000  1 -4.088194000 1.472834000 -8.797536000  1 -2.867205000 2.685553000 -9.302002000  6 -4.473577000 3.488061000 -8.132698000  1 -4.940548000 3.836172000 -9.066065000  8 -5.541187000 3.019639000 -7.260820000  6 -5.579663000 3.772819000 -6.066418000  1 -6.626395000 3.974281000 -5.802345000  7 -4.979677000 2.982341000 -4.973054000  6 -4.912726000 3.343905000 -3.634831000  7 -5.333401000 4.508320000 -3.088579000  6 -5.187366000 4.529647000 -1.758174000  7 -5.641054000 5.595935000 -1.052578000  1 -5.855174000 6.421512000 -1.594340000  1 -5.397801000 5.710684000 -0.058309000  7 -4.634008000 3.500304000 -1.031835000  1 -4.604010000 3.577605000 0.014582000  6 -4.201928000 2.271888000 -1.568224000  8 -3.748915000 1.374285000 -0.830638000  6 -4.374539000 2.220730000 -2.984124000  7 -4.146599000 1.184488000 -3.884992000  6 -4.531264000 1.667952000 -5.054502000  1 -4.491068000 1.154333000 -6.000903000  6 -4.823073000 5.085643000 -6.319339000  1 -4.321260000 5.448381000 -5.411992000  8 -5.759043000 6.037992000 -6.811179000  1 -5.251171000 6.540514000 -7.477626000  6 -3.811920000 4.688768000 -7.420175000  1 -2.858414000 4.397242000 -6.977883000  8 -3.644755000 5.812989000 -8.277098000  15 -2.388861000 5.869570000 -9.396497000  8 -2.992973000 5.895112000 -10.776917000  8 -1.339380000 4.839870000 -9.012011000  8 -1.865094000 7.408241000 -9.086203000  6 -0.992960000 7.613817000 -7.956576000  1 -0.073735000 8.088439000 -8.331283000  1 -0.734344000 6.666213000 -7.474550000  6 -1.566855000 8.550220000 -6.907460000  1 -2.020852000 9.433044000 -7.384423000  8 -2.565083000 7.924379000 -6.060613000  6 -2.339652000 8.245440000 -4.701242000  1 -3.234029000 8.718159000 -4.271229000  7 -2.129957000 7.013308000 -3.909113000  6 -1.557801000 5.799493000 -4.277619000  7 -1.003576000 5.480065000 -5.464184000  6 -0.648851000 4.196514000 -5.554049000  7 -0.095403000 3.736454000 -6.706639000  1 -0.460027000 4.201232000 -7.555263000  1 -0.062777000 2.719413000 -6.765211000  7 -0.787934000 3.301640000 -4.510798000  1 -0.667472000 2.309281000 -4.737880000  6 -1.192786000 3.630138000 -3.196966000  8 -1.140845000 2.777004000 -2.289472000  6 -1.653026000 4.985844000 -3.132275000  7 -2.235530000 5.685633000 -2.087303000  6 -2.516729000 6.879371000 -2.580608000  1 -2.999128000 7.688137000 -2.044426000  6 -1.157196000 9.236730000 -4.620927000  1 -0.504784000 9.024749000 -3.761255000  8 -1.688762000 10.556373000 -4.561286000  1 -1.085059000 11.076402000 -5.128385000  6 -0.444377000 9.017205000 -5.968228000  1 0.328112000 8.243790000 -5.876674000  8 0.105537000 10.235947000 -6.470103000  15 1.780766000 10.343320000 -6.639720000  8 2.010173000 11.685270000 -7.287419000  8 2.315094000 9.047302000 -7.207397000  8 2.158414000 10.444166000 -5.011689000  6 3.191661000 9.591462000 -4.507354000  1 4.188050000 9.974574000 -4.785356000  1 3.089815000 8.576483000 -4.914803000  6 3.116101000 9.554628000 -2.997488000  1 3.127666000 10.574661000 -2.582376000  8 1.899864000 8.883046000 -2.580786000  6 2.129425000 8.166384000 -1.377984000  1 1.514952000 8.556126000 -0.555400000  7 1.747756000 6.765396000 -1.588020000  6 1.680676000 5.834843000 -0.563261000  7 1.776478000 6.071197000 0.760695000  6 1.774112000 4.922531000 1.454956000  1 1.877859000 4.989864000 2.537750000  7 1.664966000 3.668095000 0.978731000  6 1.540349000 3.448370000 -0.357634000  7 1.448075000 2.178439000 -0.810975000  1 0.934948000 2.057758000 -1.678451000  1 1.364002000 1.433823000 -0.108112000  6 1.566238000 4.586887000 -1.205408000  7 1.579088000 4.742004000 -2.588840000  6 1.697734000 6.048613000 -2.778038000  1 1.729257000 6.552042000 -3.735561000  6 3.627043000 8.288484000 -1.017816000  1 4.025871000 7.309384000 -0.720924000  8 3.761003000 9.251934000 0.007473000  1 4.726943000 9.304603000 0.179582000  6 4.248142000 8.740475000 -2.361864000  1 4.475722000 7.854958000 -2.972660000  8 5.408115000 9.562892000 -2.236357000  15 6.841440000 8.903647000 -1.721957000  8 7.925145000 9.881608000 -2.086893000  8 6.925713000 7.430007000 -2.097828000  8 6.531957000 8.992910000 -0.055211000  6 7.292028000 8.022049000 0.690209000  1 8.362900000 8.109284000 0.458270000  1 6.958396000 7.015251000 0.409805000  6 7.181610000 8.171111000 2.190781000  1 7.503292000 9.166704000 2.522968000  8 5.831606000 7.953509000 2.697572000  6 5.823254000 6.817099000 3.544149000  1 5.082463000 6.971905000 4.336926000  7 5.443408000 5.627496000 2.738757000  6 5.523679000 4.287323000 3.083273000  7 5.853202000 3.780090000 4.296067000  6 5.854404000 2.451183000 4.310222000  7 6.218465000 1.778529000 5.446807000  1 6.252886000 2.356190000 6.277708000  1 5.823849000 0.856209000 5.596595000  7 5.556727000 1.674603000 3.214945000  1 5.662882000 0.643759000 3.304285000  6 5.160017000 2.159257000 1.948951000  8 4.871036000 1.363706000 1.031471000  6 5.154091000 3.588059000 1.916423000  7 4.831877000 4.467640000 0.891450000  6 5.004905000 5.666705000 1.415505000  1 4.802455000 6.613061000 0.940976000  6 7.255085000 6.663978000 4.081014000  1 7.469924000 5.634648000 4.398032000  8 7.438850000 7.597898000 5.137798000  1 8.374392000 7.863188000 5.078649000  6 8.056920000 7.073325000 2.838313000  1 8.117920000 6.212649000 2.163185000  8 9.354989000 7.543771000 3.182955000  15 10.661171000 7.018467000 2.244920000  8 11.845859000 7.776125000 2.777005000  8 10.264891000 6.984992000 0.781786000  8 10.716083000 5.409692000 2.663800000  6 10.864359000 4.994827000 4.026495000  1 10.165091000 5.542668000 4.673862000  1 11.889943000 5.187382000 4.376499000  6 10.600328000 3.500507000 4.112319000  1 10.810740000 3.173841000 5.143320000  8 9.213646000 3.206681000 3.802248000  6 9.126998000 2.054044000 2.974168000  1 8.372008000 1.363557000 3.368909000  7 8.679864000 2.467054000 1.628883000  6 8.070069000 1.656134000 0.683661000  7 7.897720000 0.322616000 0.777500000  6 7.285352000 -0.192931000 -0.295197000  7 7.081707000 -1.516713000 -0.365383000  1 7.445265000 -2.073402000 0.400728000  1 6.718526000 -1.965556000 -1.208542000  7 6.837579000 0.578311000 -1.350632000  1 6.376486000 0.110820000 -2.125616000  6 6.969906000 1.990820000 -1.474300000  8 6.477938000 2.584418000 -2.446787000  6 7.708051000 2.518980000 -0.365699000  7 8.120479000 3.818154000 -0.085676000  6 8.695677000 3.752079000 1.107338000  1 9.193207000 4.556866000 1.626967000  6 10.519603000 1.408239000 2.949816000  1 10.707654000 0.864584000 2.016298000  8 10.640189000 0.541814000 4.078592000  1 11.537347000 0.715835000 4.422016000  6 11.421643000 2.640059000 3.139597000  1 11.569006000 3.167550000 2.191282000  8 12.674288000 2.302807000 3.734298000  15 14.020966000 2.252378000 2.704309000  8 15.184093000 1.943419000 3.610636000  8 13.963165000 3.423913000 1.756145000  8 13.632734000 0.924097000 1.769982000  6 13.717314000 -0.383944000 2.350813000  1 13.073979000 -0.453091000 3.239785000  1 14.752566000 -0.605258000 2.648761000  6 13.266912000 -1.407908000 1.325392000  1 13.437485000 -2.417195000 1.734071000  8 11.850872000 -1.235581000 1.064227000  6 11.575904000 -1.414899000 -0.319149000  1 10.780314000 -2.155953000 -0.461624000  7 11.076787000 -0.157135000 -0.891970000  6 10.203984000 -0.094731000 -1.966340000  7 9.494602000 -1.092079000 -2.512025000  6 8.779241000 -0.670099000 -3.568793000  1 8.163993000 -1.427355000 -4.052381000  7 8.696793000 0.578222000 -4.082349000  6 9.395250000 1.571047000 -3.480122000  7 9.318075000 2.837654000 -4.002760000  1 9.594069000 3.579924000 -3.370701000  1 8.457256000 3.026481000 -4.504443000  6 10.208527000 1.256631000 -2.368259000  7 11.059027000 2.009538000 -1.560017000  6 11.568409000 1.130215000 -0.703965000  1 12.278161000 1.345183000 0.087864000  6 12.877511000 -1.858822000 -1.006351000  1 12.942890000 -1.446763000 -2.018748000  8 12.900726000 -3.282583000 -1.036733000  1 13.779600000 -3.492696000 -1.409076000  6 13.949712000 -1.305210000 -0.045240000  1 14.167323000 -0.258773000 -0.287493000  8 15.161976000 -2.065209000 -0.030052000  15 16.211945000 -1.907695000 -1.316863000  8 17.525445000 -2.493420000 -0.860238000  8 16.094972000 -0.542466000 -1.954284000  8 15.434470000 -3.040310000 -2.325692000  6 15.808006000 -2.973143000 -3.711252000  1 16.897367000 -3.099687000 -3.808446000  1 15.533512000 -1.993823000 -4.130213000  6 15.103056000 -4.044154000 -4.521193000  1 15.420010000 -3.924195000 -5.570209000  8 15.492484000 -5.367775000 -4.069921000  6 14.372607000 -6.234589000 -4.062233000  1 14.608304000 -7.161326000 -4.602117000  7 14.071522000 -6.607745000 -2.662502000  6 13.205494000 -7.609097000 -2.267443000  7 12.411110000 -8.347519000 -3.086140000  6 11.665823000 -9.221902000 -2.423941000  7 10.812506000 -10.059767000 -3.102382000  1 10.721550000 -9.815601000 -4.082589000  1 9.917244000 -10.231368000 -2.652472000  7 11.726312000 -9.383280000 -1.057065000  1 11.160526000 -10.104946000 -0.621211000  6 12.531523000 -8.626286000 -0.145453000  8 12.485922000 -8.873635000 1.064641000  6 13.311850000 -7.657812000 -0.865868000  7 14.221713000 -6.712862000 -0.407711000  6 14.657184000 -6.107194000 -1.503016000  1 15.377229000 -5.300281000 -1.554569000  6 13.199851000 -5.498663000 -4.727811000  1 12.234812000 -5.778124000 -4.291725000  8 13.228664000 -5.778710000 -6.126099000  1 13.028811000 -4.915552000 -6.540087000  6 13.566722000 -4.019147000 -4.486933000  1 13.196024000 -3.672514000 -3.516690000  8 13.078581000 -3.203164000 -5.554731000  15 11.827403000 -2.108807000 -5.200228000  8 11.458533000 -1.500474000 -6.528955000  8 12.240113000 -1.299212000 -4.000466000  8 10.641728000 -3.190574000 -4.705832000  6 9.951428000 -3.837350000 -5.782549000  1 10.671409000 -4.219469000 -6.521671000  1 9.289514000 -3.126083000 -6.298100000  6 9.106414000 -5.007573000 -5.329226000  1 8.666219000 -5.426211000 -6.250154000  8 9.942013000 -6.024500000 -4.710557000  6 9.180695000 -6.755485000 -3.767016000  1 9.277839000 -7.832152000 -3.943390000  7 9.675118000 -6.484596000 -2.403228000  6 9.337955000 -7.237294000 -1.295812000  7 8.568568000 -8.357874000 -1.300231000  6 8.329795000 -8.819601000 -0.076561000  7 7.556498000 -9.938463000 0.088819000  1 7.033870000 -10.192566000 -0.741941000  1 7.014177000 -9.994681000 0.944316000  7 8.864960000 -8.252409000 1.058175000  1 8.630897000 -8.645170000 1.965059000  6 9.681340000 -7.072832000 1.121213000  8 10.055528000 -6.645790000 2.216579000  6 9.898020000 -6.557972000 -0.201452000  7 10.546517000 -5.405321000 -0.620205000  6 10.394196000 -5.381227000 -1.935703000  1 10.765971000 -4.622866000 -2.613152000  6 7.731188000 -6.278253000 -3.899262000  1 7.181410000 -6.403996000 -2.964111000  8 7.097345000 -6.999694000 -4.954587000  1 6.573125000 -6.329071000 -5.432259000  6 7.953225000 -4.810542000 -4.327495000  1 8.253499000 -4.181573000 -3.481772000  8 6.803265000 -4.273370000 -4.978523000  15 5.613624000 -3.519985000 -4.029532000  8 4.624129000 -2.985144000 -5.025620000  8 6.303637000 -2.676360000 -2.976011000  8 4.995293000 -4.796874000 -3.162970000  6 4.121336000 -5.808139000 -3.687909000  1 4.635308000 -6.385046000 -4.468134000  1 3.209885000 -5.348954000 -4.091508000  6 3.715783000 -6.736280000 -2.546524000  1 3.075440000 -7.530513000 -2.961774000  8 4.895540000 -7.357981000 -1.952956000  6 4.800251000 -7.378919000 -0.543953000  1 4.998460000 -8.382710000 -0.158176000  7 5.848734000 -6.484955000 0.044784000  6 6.404139000 -5.472549000 -0.687621000  1 6.074805000 -5.417425000 -1.716551000  6 6.193052000 -6.688029000 1.384266000  8 5.825897000 -7.673745000 2.029065000  7 6.976655000 -5.676583000 1.919181000  1 7.055177000 -5.701895000 2.934124000  6 7.498726000 -4.548211000 1.259595000  8 8.054412000 -3.637838000 1.900487000  6 7.271306000 -4.570554000 -0.161013000  1 7.732112000 -3.832178000 -0.801102000  6 3.380885000 -6.910520000 -0.143626000  1 3.425524000 -6.295831000 0.763287000  8 2.549763000 -8.026807000 0.063337000  1 2.033867000 -7.811768000 0.888816000  6 2.969233000 -6.063755000 -1.383172000  1 3.346165000 -5.038424000 -1.281077000  8 1.564582000 -6.081649000 -1.588234000  15 0.755007000 -4.658001000 -1.969674000  8 -0.634871000 -5.122907000 -2.338738000  8 1.604805000 -3.792791000 -2.870029000  8 0.745314000 -3.877162000 -0.494593000  6 0.169912000 -4.582793000 0.624685000  1 0.708742000 -5.525438000 0.790416000  1 -0.890702000 -4.805588000 0.442272000  6 0.299324000 -3.735876000 1.873587000  1 1.291238000 -3.261402000 1.885380000  8 -0.720080000 -2.714576000 1.914896000  6 -1.103456000 -2.459421000 3.264294000  1 -0.808687000 -1.446795000 3.570208000  7 -2.564064000 -2.489987000 3.361460000  6 -3.432360000 -3.481000000 2.947023000  7 -3.102456000 -4.638548000 2.328336000  6 -4.158979000 -5.366494000 1.981412000  7 -3.970448000 -6.514765000 1.263331000  1 -3.015679000 -6.852884000 1.265305000  1 -4.676922000 -7.237867000 1.312440000  7 -5.456722000 -4.992624000 2.252394000  1 -6.243396000 -5.593625000 1.973659000  6 -5.848714000 -3.814880000 2.941810000  8 -7.059851000 -3.607773000 3.154631000  6 -4.716284000 -3.016807000 3.297081000  7 -4.640843000 -1.764009000 3.903412000  6 -3.345919000 -1.483775000 3.918501000  1 -2.877137000 -0.580433000 4.296026000  6 -0.399906000 -3.495712000 4.180943000  1 -1.122303000 -3.954006000 4.874034000  8 0.623869000 -2.828574000 4.875511000  1 0.837262000 -3.374271000 5.685478000  6 0.138427000 -4.543380000 3.168356000  1 -0.605417000 -5.337206000 3.025221000  8 1.404823000 -5.064690000 3.573802000  15 1.661976000 -6.707059000 3.614078000  8 1.259961000 -7.301960000 2.269218000  8 1.146757000 -7.320740000 4.893181000  8 3.319654000 -6.594449000 3.630586000  6 4.084271000 -6.850138000 4.816607000  1 4.846104000 -7.591437000 4.546258000  1 3.440760000 -7.257979000 5.607664000  6 4.781868000 -5.611090000 5.362188000  1 5.399888000 -5.936707000 6.214638000  8 5.665299000 -5.043086000 4.354541000  6 5.633841000 -3.629860000 4.383969000  1 6.653011000 -3.232837000 4.392532000  7 4.997904000 -3.113582000 3.130218000  6 4.235383000 -3.916865000 2.313883000  1 4.031602000 -4.909676000 2.701883000  6 5.247170000 -1.783801000 2.803612000  8 5.869101000 -1.041826000 3.594593000  7 4.772654000 -1.368339000 1.591575000  1 4.906343000 -0.363334000 1.351436000  6 4.089780000 -2.152412000 0.634223000  8 3.825620000 -1.693051000 -0.480903000  6 3.767656000 -3.478601000 1.115256000  1 3.131104000 -4.101492000 0.503348000  6 4.849628000 -3.225799000 5.637007000  1 4.316694000 -2.281731000 5.493024000  8 5.762271000 -3.135826000 6.727883000  1 5.306481000 -3.592478000 7.461831000  6 3.908563000 -4.438434000 5.835695000  1 2.994104000 -4.339536000 5.238638000  8 3.612783000 -4.634032000 7.225698000  15 2.266629000 -3.858384000 7.882694000  8 2.339344000 -4.104907000 9.362970000  8 1.049585000 -4.228500000 7.052887000  8 2.597330000 -2.272814000 7.505510000  6 3.272860000 -1.450464000 8.475887000  1 4.358153000 -1.614916000 8.411634000  1 2.931584000 -1.713805000 9.484472000  6 2.962968000 0.013029000 8.225805000  1 3.285587000 0.585842000 9.111197000  8 3.694577000 0.495943000 7.062475000  6 2.935268000 1.501644000 6.402269000  1 3.496013000 2.438694000 6.337102000  7 2.672987000 1.068584000 5.008515000  6 2.531472000 -0.259269000 4.682299000  1 2.663077000 -0.956660000 5.502133000  6 2.397191000 2.084176000 4.078536000  8 2.403163000 3.279719000 4.403073000  7 2.134848000 1.642413000 2.806376000  1 1.931421000 2.401928000 2.096010000  6 2.029582000 0.314858000 2.378985000  8 1.762804000 0.050608000 1.193061000  6 2.239124000 -0.664832000 3.419392000  1 2.103093000 -1.716907000 3.211713000  6 1.623062000 1.703354000 7.191288000  1 0.797477000 1.877373000 6.502112000  8 1.739978000 2.787424000 8.103186000  1 1.061179000 3.422793000 7.809493000  6 1.501573000 0.341602000 7.903759000  1 1.127587000 -0.396919000 7.183617000  8 0.698511000 0.318221000 9.072934000  15 -0.921054000 -0.102938000 8.884629000  8 -1.449918000 -0.236217000 10.290866000  8 -1.070490000 -1.164987000 7.821292000  8 -1.525592000 1.284436000 8.166654000  6 -1.501659000 2.499707000 8.922305000  1 -0.521810000 2.629188000 9.401943000  1 -2.278713000 2.487292000 9.702601000  6 -1.784335000 3.667926000 8.000600000  1 -1.819131000 4.590569000 8.600485000  8 -0.721351000 3.799069000 7.008012000  6 -1.275426000 4.179625000 5.749201000  1 -0.722081000 5.029451000 5.333308000  7 -1.162783000 3.073382000 4.784916000  6 -1.225976000 3.241141000 3.407683000  7 -1.372748000 4.418617000 2.761826000  6 -1.485339000 4.269277000 1.434183000  7 -1.635477000 5.367104000 0.666148000  1 -1.790648000 6.227743000 1.171943000  1 -1.925827000 5.337550000 -0.325874000  7 -1.427848000 3.045344000 0.801381000  1 -1.475839000 3.004290000 -0.222976000  6 -1.233620000 1.786277000 1.443919000  8 -1.150955000 0.750635000 0.776122000  6 -1.171676000 1.946756000 2.872204000  7 -1.115167000 1.001135000 3.891493000  6 -1.130519000 1.697441000 5.020003000  1 -1.148174000 1.293385000 6.025314000  6 -2.750055000 4.519868000 5.993698000  1 -3.331116000 4.287805000 5.097431000  8 -2.869508000 5.888081000 6.351620000  1 -3.833010000 6.035557000 6.435228000  6 -3.071913000 3.576739000 7.170337000  1 -3.218656000 2.554796000 6.803669000  8 -4.191426000 3.993239000 7.937539000  15 -5.483862000 2.939234000 8.181410000  8 -5.771399000 2.959679000 9.663067000  8 -5.290921000 1.672537000 7.382220000  8 -6.685302000 3.787640000 7.365349000  6 -6.914413000 5.156675000 7.720567000  1 -6.135894000 5.528225000 8.398707000  1 -7.885716000 5.248469000 8.231268000  6 -6.963442000 6.034427000 6.482210000  1 -7.153435000 7.070604000 6.804612000  8 -5.689963000 5.990639000 5.777452000  6 -5.896924000 6.029406000 4.367998000  1 -5.271497000 6.803714000 3.915711000  7 -5.487349000 4.738521000 3.753509000  6 -5.450208000 3.573446000 4.471279000  1 -5.749573000 3.643833000 5.511052000  6 -5.147881000 4.754539000 2.376952000  8 -5.253159000 5.840282000 1.757470000  7 -4.731975000 3.601063000 1.802768000  6 -4.647852000 2.452844000 2.517658000  7 -4.201656000 1.365120000 1.889171000  1 -4.188873000 0.470679000 2.358538000  1 -3.995794000 1.383951000 0.873709000  6 -5.024297000 2.412321000 3.900715000  1 -4.985285000 1.500202000 4.486530000  6 -7.390006000 6.294762000 4.148102000  1 -7.745167000 5.851012000 3.212539000  8 -7.622695000 7.703924000 4.159807000  1 -8.448448000 7.808781000 4.670376000  6 -8.000415000 5.659813000 5.412148000  1 -8.079488000 4.570218000 5.319887000  8 -9.266376000 6.236704000 5.726346000  15 -10.649481000 5.318367000 5.360192000  8 -11.801830000 6.179642000 5.805317000  8 -10.419324000 3.894255000 5.796310000  8 -10.560842000 5.269406000 3.690432000  6 -10.820190000 6.461829000 2.941186000  1 -10.173330000 7.281692000 3.285114000  1 -11.868881000 6.772088000 3.060216000  6 -10.554034000 6.207551000 1.469356000  1 -10.802676000 7.128504000 0.917524000  8 -9.146778000 5.905723000 1.261950000  6 -9.003385000 4.878281000 0.291748000  1 -8.238334000 5.161148000 -0.442411000  7 -8.535366000 3.639390000 0.945733000  6 -7.975333000 2.557833000 0.284213000  7 -7.769847000 2.409199000 -1.041263000  6 -7.177657000 1.232264000 -1.310070000  1 -6.976536000 1.015314000 -2.359693000  7 -6.788267000 0.280318000 -0.443099000  6 -7.002570000 0.447988000 0.889866000  7 -6.598698000 -0.530605000 1.727535000  1 -6.625662000 -0.350489000 2.721860000  1 -5.814010000 -1.106507000 1.400639000  6 -7.664779000 1.629892000 1.299417000  7 -8.044944000 2.111242000 2.545332000  6 -8.565923000 3.306010000 2.296705000  1 -9.018812000 3.972250000 3.019251000  6 -10.379749000 4.663295000 -0.343128000  1 -10.511778000 3.637134000 -0.698322000  8 -10.552378000 5.596738000 -1.411051000  1 -11.479922000 5.889474000 -1.326176000  6 -11.310664000 5.033220000 0.825511000  1 -11.411241000 4.206864000 1.537894000  8 -12.588509000 5.457770000 0.348195000  15 -13.900849000 4.400462000 0.545976000  8 -15.072495000 5.145631000 -0.037837000  8 -13.854777000 3.818243000 1.935784000  8 -13.459686000 3.130385000 -0.453934000  6 -13.458466000 3.313708000 -1.872707000  1 -12.959303000 4.256735000 -2.139441000  1 -14.488347000 3.347536000 -2.259262000  6 -12.722325000 2.173465000 -2.556020000  1 -12.780843000 2.356310000 -3.641509000  8 -11.321296000 2.184481000 -2.160684000  6 -10.847352000 0.858911000 -1.987157000  1 -9.862407000 0.752206000 -2.454197000  7 -10.696725000 0.550800000 -0.553533000  6 -10.028633000 -0.553629000 -0.058029000  7 -9.311986000 -1.446962000 -0.782965000  6 -8.737721000 -2.373206000 -0.022006000  7 -7.881797000 -3.286647000 -0.589501000  1 -7.796809000 -3.179289000 -1.595486000  1 -7.990078000 -4.249178000 -0.281016000  7 -8.909179000 -2.458195000 1.339380000  1 -8.270243000 -3.052477000 1.886131000  6 -9.657825000 -1.548080000 2.141580000  8 -9.703969000 -1.683824000 3.371810000  6 -10.269433000 -0.539139000 1.325039000  7 -11.071301000 0.540704000 1.676393000  6 -11.316000000 1.165939000 0.534651000  1 -11.943589000 2.036088000 0.396721000  6 -11.903463000 -0.059133000 -2.600656000  1 -11.888484000 -1.064919000 -2.166530000  8 -11.705064000 -0.102956000 -4.016331000  1 -12.602349000 -0.019591000 -4.391961000  6 -13.188958000 0.730921000 -2.281952000  1 -13.482613000 0.604464000 -1.233499000  8 -14.257170000 0.360933000 -3.152694000  15 -15.273610000 -0.896128000 -2.608804000  8 -16.243755000 -1.109679000 -3.742402000  8 -15.676734000 -0.615980000 -1.182828000  8 -14.194767000 -2.168177000 -2.510119000  6 -13.715823000 -2.752118000 -3.731737000  1 -13.089480000 -2.035609000 -4.277609000  1 -14.556721000 -3.050943000 -4.374182000  6 -12.903395000 -3.989192000 -3.418815000  1 -12.555263000 -4.419948000 -4.372427000  8 -11.757252000 -3.660107000 -2.593857000  6 -11.455601000 -4.754354000 -1.739338000  1 -10.430404000 -5.111024000 -1.898316000  7 -11.533458000 -4.317819000 -0.337024000  6 -10.785431000 -4.850672000 0.695236000  7 -9.690895000 -5.646370000 0.566502000  6 -9.147371000 -5.971217000 1.731344000  7 -7.982629000 -6.721897000 1.742258000  1 -7.846138000 -7.211171000 0.861849000  1 -7.869417000 -7.343396000 2.538261000  7 -9.644203000 -5.552384000 2.937007000  1 -9.170614000 -5.823940000 3.793059000  6 -10.836697000 -4.791599000 3.134665000  8 -11.257489000 -4.598494000 4.284033000  6 -11.390179000 -4.387833000 1.873928000  7 -12.488708000 -3.588033000 1.580112000  6 -12.550963000 -3.570450000 0.256266000  1 -13.264850000 -3.024281000 -0.350805000  6 -12.483791000 -5.873648000 -2.011391000  1 -12.770726000 -6.354924000 -1.068234000  8 -11.920201000 -6.804153000 -2.921116000  1 -12.670136000 -7.363139000 -3.212465000  6 -13.646100000 -5.086004000 -2.648878000  1 -14.293114000 -4.651717000 -1.878242000  8 -14.393271000 -5.907277000 -3.527730000  15 -16.061865000 -5.742703000 -3.642131000  8 -16.370750000 -5.232116000 -5.030431000  8 -16.622016000 -5.116917000 -2.387911000  8 -16.442819000 -7.376273000 -3.540487000  6 -15.826059000 -8.273249000 -4.472297000  1 -15.127238000 -7.748113000 -5.135378000  1 -16.605756000 -8.726201000 -5.106368000  6 -15.095410000 -9.395602000 -3.748465000  1 -14.721968000 -10.112373000 -4.497986000  8 -13.965868000 -8.865517000 -3.006183000  6 -13.847151000 -9.499461000 -1.736140000  1 -12.823853000 -9.862335000 -1.585816000  7 -14.089738000 -8.530755000 -0.664539000  6 -13.297295000 -8.384347000 0.467513000  7 -12.168490000 -9.045437000 0.782902000  6 -11.660375000 -8.605440000 1.951751000  1 -10.731246000 -9.080974000 2.274016000  7 -12.137522000 -7.654948000 2.779491000  6 -13.275341000 -7.001469000 2.428651000  7 -13.778979000 -6.057333000 3.272955000  1 -14.413691000 -5.392916000 2.845123000  1 -13.100200000 -5.633406000 3.904937000  6 -13.910754000 -7.357754000 1.213187000  7 -15.037257000 -6.871509000 0.555671000  6 -15.114386000 -7.592702000 -0.554040000  1 -15.837479000 -7.453199000 -1.348201000  6 -14.848210000 -10.667941000 -1.733562000  1 -15.234457000 -10.864407000 -0.721270000  8 -14.191145000 -11.805936000 -2.277999000  1 -14.873365000 -12.256704000 -2.810606000  1 -16.623096000 -9.482271000 -2.194936000  6 -15.931336000 -10.167581000 -2.705127000  8 -16.617375000 -11.302466000 -3.224464000  1 -17.158322000 -11.019696000 -3.977663000  11 8.563048000 5.869001000 -1.413217000 |
| **H4** | 1 -29.763755000 0.812986000 -2.499082000  8 -30.426522000 1.531340000 -2.668619000  6 -30.907415000 1.264395000 -3.979736000  1 -31.852843000 1.808511000 -4.113721000  1 -31.115495000 0.189405000 -4.114259000  6 -29.938586000 1.671794000 -5.091151000  1 -30.244025000 1.190592000 -6.033884000  8 -29.938811000 3.114321000 -5.291961000  6 -28.627127000 3.564061000 -5.572682000  1 -28.611075000 4.170613000 -6.484885000  7 -28.171039000 4.461281000 -4.463976000  6 -28.792416000 4.470216000 -3.234644000  1 -29.540962000 3.695541000 -3.090921000  6 -27.132267000 5.344288000 -4.775649000  8 -26.544852000 5.324525000 -5.859225000  7 -26.832668000 6.241524000 -3.762607000  1 -26.089439000 6.900736000 -3.973435000  6 -27.465865000 6.383930000 -2.505169000  8 -27.122496000 7.292938000 -1.740494000  6 -28.484202000 5.382027000 -2.271290000  1 -28.987973000 5.363029000 -1.312128000  6 -27.724229000 2.325539000 -5.710401000  1 -26.701623000 2.522918000 -5.364762000  8 -27.752584000 1.897666000 -7.071159000  1 -27.656405000 0.929334000 -7.035254000  6 -28.471929000 1.342841000 -4.808074000  1 -28.240258000 1.578051000 -3.764011000  8 -28.176597000 -0.018348000 -5.111536000  8 -28.707186000 -0.609716000 -2.620281000  15 -27.834990000 -1.015689000 -3.798875000  8 -27.804848000 -2.414639000 -4.348893000  8 -26.320926000 -0.478254000 -3.382107000  6 -25.306136000 -0.356501000 -4.391756000  1 -25.753559000 -0.061945000 -5.349630000  1 -24.779680000 -1.313771000 -4.525830000  6 -24.301886000 0.686195000 -3.930744000  1 -23.530753000 0.807209000 -4.709251000  8 -24.983022000 1.944364000 -3.726040000  6 -24.488530000 2.604175000 -2.574370000  1 -24.054169000 3.578774000 -2.832343000  7 -25.609215000 2.888540000 -1.668054000  6 -25.660285000 3.979998000 -0.825927000  7 -24.760232000 4.989535000 -0.785215000  6 -25.075994000 5.926038000 0.101803000  7 -24.232592000 6.984335000 0.292904000  1 -23.557058000 7.105816000 -0.452362000  1 -24.627975000 7.851260000 0.636203000  7 -26.189566000 5.847054000 0.909025000  1 -26.387941000 6.603191000 1.556962000  6 -27.167731000 4.809910000 0.901180000  8 -28.122954000 4.869595000 1.697324000  6 -26.831353000 3.806793000 -0.062826000  7 -27.481848000 2.633470000 -0.427090000  6 -26.724943000 2.102718000 -1.376520000  1 -26.904270000 1.168240000 -1.895005000  6 -23.403828000 1.717902000 -1.912041000  1 -23.602390000 1.638723000 -0.838764000  8 -22.127706000 2.291805000 -2.136756000  1 -21.632583000 2.102924000 -1.315705000  6 -23.607867000 0.341104000 -2.604905000  1 -24.272847000 -0.275916000 -1.989273000  8 -22.401843000 -0.356512000 -2.887162000  15 -22.066805000 -1.796147000 -2.089103000  8 -20.992317000 -2.456475000 -2.917542000  8 -23.351156000 -2.475714000 -1.677446000  8 -21.418675000 -1.212654000 -0.661440000  6 -20.218981000 -0.434059000 -0.706058000  1 -20.184473000 0.170814000 -1.623190000  1 -19.337146000 -1.091773000 -0.674024000  6 -20.187964000 0.484526000 0.502204000  1 -19.242371000 1.047967000 0.498939000  8 -21.288434000 1.434470000 0.418064000  6 -21.939760000 1.590095000 1.678790000  1 -22.052970000 2.654716000 1.911702000  7 -23.300449000 1.042307000 1.621649000  6 -24.425009000 1.656421000 2.149951000  7 -24.461272000 2.872694000 2.749923000  6 -25.685380000 3.215076000 3.135165000  7 -25.891680000 4.401472000 3.793830000  1 -25.109485000 5.040519000 3.704078000  1 -26.792336000 4.841863000 3.618994000  7 -26.784650000 2.402724000 2.968938000  1 -27.701404000 2.775325000 3.197654000  6 -26.805667000 1.117652000 2.341412000  8 -27.866541000 0.490192000 2.255868000  6 -25.481435000 0.762010000 1.909732000  7 -25.026793000 -0.371742000 1.245834000  6 -23.725515000 -0.181499000 1.095901000  1 -23.038161000 -0.831986000 0.568376000  6 -21.054263000 0.884081000 2.716784000  1 -21.645602000 0.453096000 3.534876000  8 -20.095295000 1.817910000 3.196664000  1 -19.267319000 1.298424000 3.245090000  6 -20.335954000 -0.199108000 1.873115000  1 -20.944312000 -1.108730000 1.794773000  8 -19.034247000 -0.475749000 2.371766000  15 -18.883386000 -1.545899000 3.702595000  8 -18.556213000 -2.919957000 3.167190000  8 -20.032657000 -1.309453000 4.651943000  8 -17.546498000 -0.805569000 4.346675000  6 -16.421948000 -0.519133000 3.492524000  1 -16.459169000 0.546135000 3.218899000  1 -16.435032000 -1.123350000 2.576921000  6 -15.115652000 -0.824207000 4.209363000  1 -14.287037000 -0.495899000 3.564901000  8 -15.020340000 -0.081343000 5.457996000  6 -14.960116000 -0.985109000 6.548907000  1 -14.332341000 -0.528067000 7.322156000  7 -16.303529000 -1.174861000 7.143105000  6 -17.190108000 -2.248604000 7.119230000  7 -16.990121000 -3.517052000 6.711644000  6 -18.100403000 -4.266393000 6.835233000  1 -18.005188000 -5.306554000 6.514944000  7 -19.317362000 -3.911382000 7.293154000  6 -19.488425000 -2.631483000 7.705820000  7 -20.701924000 -2.264356000 8.207295000  1 -20.903943000 -1.273345000 8.241076000  1 -21.481671000 -2.870280000 7.983082000  6 -18.394166000 -1.738074000 7.644938000  7 -18.272737000 -0.392810000 7.974384000  6 -17.023596000 -0.101540000 7.653132000  1 -16.558590000 0.873253000 7.754328000  6 -14.339232000 -2.274273000 6.003664000  1 -14.622172000 -3.164620000 6.579388000  8 -12.926825000 -2.082115000 5.980081000  1 -12.644601000 -2.519293000 5.153356000  6 -14.903796000 -2.313788000 4.566359000  1 -15.853391000 -2.860671000 4.557218000  8 -13.933106000 -2.947354000 3.728351000  15 -14.545040000 -3.752852000 2.366731000  8 -15.108297000 -2.709139000 1.422825000  8 -15.347686000 -4.948548000 2.824068000  8 -13.029048000 -4.188246000 1.840639000  6 -12.809349000 -5.472295000 1.247903000  1 -13.260301000 -6.268114000 1.857619000  1 -11.724049000 -5.602159000 1.240459000  6 -13.304607000 -5.614074000 -0.190882000  1 -12.691441000 -6.392030000 -0.680155000  8 -14.690546000 -6.016134000 -0.212863000  6 -15.295975000 -5.597302000 -1.445871000  1 -15.540172000 -6.477519000 -2.053520000  7 -16.564848000 -4.943267000 -1.145396000  6 -17.732137000 -5.632862000 -0.855584000  7 -17.900588000 -6.973419000 -0.957777000  6 -19.114314000 -7.368871000 -0.590192000  7 -19.462117000 -8.685618000 -0.721328000  1 -18.675025000 -9.311736000 -0.841395000  1 -20.201074000 -9.049290000 -0.132174000  7 -20.078430000 -6.499651000 -0.128514000  1 -20.981549000 -6.862824000 0.161281000  6 -19.943542000 -5.078912000 0.023178000  8 -20.877465000 -4.430491000 0.508909000  6 -18.649841000 -4.656037000 -0.436166000  7 -18.063528000 -3.392363000 -0.462209000  6 -16.826143000 -3.600570000 -0.871037000  1 -16.056854000 -2.846118000 -0.956180000  6 -14.274162000 -4.707639000 -2.179563000  1 -14.731175000 -3.816247000 -2.628678000  8 -13.624251000 -5.494820000 -3.176174000  1 -12.682553000 -5.237897000 -3.111019000  6 -13.271149000 -4.349405000 -1.057712000  1 -13.622001000 -3.487486000 -0.480225000  8 -11.957516000 -4.168002000 -1.569472000  15 -11.554193000 -2.749436000 -2.384504000  8 -12.779759000 -1.890895000 -2.561365000  8 -10.719755000 -3.253326000 -3.555636000  8 -10.578538000 -1.970432000 -1.290285000  6 -9.212692000 -2.424760000 -1.189779000  1 -8.554788000 -1.714342000 -1.715289000  1 -9.106639000 -3.412967000 -1.641123000  6 -8.747288000 -2.516684000 0.253809000  1 -8.717631000 -1.528232000 0.738444000  8 -9.636667000 -3.373051000 1.014366000  6 -8.907434000 -4.279021000 1.817515000  1 -9.200367000 -4.173763000 2.871030000  7 -9.228920000 -5.670922000 1.462428000  6 -9.365361000 -6.261904000 0.210211000  7 -9.317750000 -5.643471000 -0.987320000  6 -9.641952000 -6.429405000 -2.010387000  7 -9.711412000 -5.903547000 -3.263521000  1 -9.983236000 -4.902389000 -3.319516000  1 -10.132260000 -6.483536000 -3.979013000  7 -9.885795000 -7.782873000 -1.851733000  1 -10.119621000 -8.345555000 -2.664659000  6 -9.874846000 -8.510962000 -0.618752000  8 -10.096613000 -9.728175000 -0.616250000  6 -9.610541000 -7.622171000 0.478051000  7 -9.607291000 -7.865728000 1.850705000  6 -9.378637000 -6.684256000 2.397895000  1 -9.279401000 -6.485450000 3.457022000  6 -7.415141000 -3.957369000 1.669997000  1 -6.816709000 -4.875346000 1.618093000  8 -7.045876000 -3.150823000 2.780541000  1 -6.115737000 -2.898420000 2.584700000  6 -7.362228000 -3.186714000 0.332965000  1 -7.253030000 -3.912101000 -0.483612000  8 -6.315707000 -2.213735000 0.266827000  15 -4.737189000 -2.773609000 0.150152000  8 -3.876146000 -1.596152000 -0.228172000  8 -4.726466000 -4.094034000 -0.585047000  8 -4.501481000 -3.123247000 1.798147000  6 -3.398646000 -2.569260000 2.534024000  1 -3.799746000 -2.174941000 3.477761000  1 -2.937913000 -1.747696000 1.969961000  6 -2.343662000 -3.623810000 2.848444000  1 -1.549914000 -3.142997000 3.435753000  8 -2.919905000 -4.694901000 3.662266000  6 -2.875999000 -5.922551000 2.954348000  1 -2.090209000 -6.588533000 3.343810000  7 -4.146874000 -6.623284000 3.145004000  6 -4.351684000 -7.986721000 2.982153000  7 -3.426997000 -8.949816000 2.801500000  6 -4.006515000 -10.159728000 2.687202000  1 -3.322122000 -11.001643000 2.555245000  7 -5.315647000 -10.480022000 2.697042000  6 -6.228240000 -9.484812000 2.870881000  7 -7.544924000 -9.820592000 2.881858000  1 -8.255622000 -9.116308000 2.657155000  1 -7.747067000 -10.762946000 2.568665000  6 -5.751428000 -8.160299000 3.062404000  7 -6.384133000 -6.942751000 3.292697000  6 -5.397874000 -6.055250000 3.325723000  1 -5.511178000 -4.991375000 3.487036000  6 -2.592827000 -5.572709000 1.467010000  1 -3.529533000 -5.308030000 0.964928000  8 -1.979112000 -6.606650000 0.727897000  1 -1.060152000 -6.630228000 1.051158000  6 -1.726991000 -4.317143000 1.630776000  1 -1.707299000 -3.681189000 0.738592000  8 -0.408390000 -4.801386000 1.960661000  15 0.913347000 -3.860235000 1.570283000  8 2.072661000 -4.470109000 2.312450000  8 0.536882000 -2.390425000 1.672982000  8 1.019072000 -4.125877000 -0.068821000  6 1.325132000 -5.437310000 -0.578826000  1 0.606198000 -5.628542000 -1.386454000  1 1.188100000 -6.208630000 0.192438000  6 2.744421000 -5.561139000 -1.136731000  1 2.824095000 -6.548078000 -1.609483000  8 2.991563000 -4.542395000 -2.151650000  6 3.988788000 -3.632836000 -1.685268000  1 4.937558000 -3.792570000 -2.208759000  7 3.569311000 -2.270839000 -1.984793000  6 4.305428000 -1.295091000 -2.642727000  7 5.546000000 -1.438158000 -3.161998000  6 6.011444000 -0.311122000 -3.702823000  7 7.228896000 -0.315409000 -4.319304000  1 7.912689000 -0.972821000 -3.920227000  1 7.611264000 0.591100000 -4.561108000  7 5.291484000 0.862233000 -3.739572000  1 5.770836000 1.723647000 -4.040869000  6 3.997588000 1.070404000 -3.189843000  8 3.486100000 2.201311000 -3.227819000  6 3.485980000 -0.150997000 -2.636147000  7 2.278888000 -0.430385000 -2.000634000  6 2.364153000 -1.693261000 -1.618361000  1 1.615499000 -2.253711000 -1.072268000  6 4.143988000 -3.898869000 -0.166703000  1 3.345874000 -3.383026000 0.371326000  8 5.359960000 -3.457853000 0.389297000  1 6.058944000 -4.074734000 0.099869000  6 3.879089000 -5.408663000 -0.116151000  1 3.606842000 -5.758706000 0.885286000  8 5.096349000 -6.002312000 -0.590110000  15 5.363335000 -7.658685000 -0.617350000  8 4.070356000 -8.373700000 -0.945761000  8 6.230851000 -8.045404000 0.554775000  8 6.394049000 -7.640175000 -1.942903000  6 5.818395000 -7.481443000 -3.248626000  1 4.757768000 -7.207437000 -3.178617000  1 5.883705000 -8.441652000 -3.783677000  6 6.537146000 -6.440041000 -4.091140000  1 6.123821000 -6.514559000 -5.111222000  8 6.276933000 -5.103238000 -3.585155000  6 7.435000000 -4.290583000 -3.717881000  1 7.158489000 -3.320633000 -4.143104000  7 7.995186000 -4.031848000 -2.375112000  6 8.746894000 -2.940363000 -1.980355000  7 9.211879000 -1.947023000 -2.774746000  6 9.958527000 -1.054442000 -2.106691000  7 10.476428000 0.007552000 -2.770248000  1 10.491457000 -0.082705000 -3.777803000  1 11.246959000 0.546368000 -2.336957000  7 10.211902000 -1.134512000 -0.758324000  1 10.829541000 -0.408645000 -0.310885000  6 9.727863000 -2.139409000 0.107309000  8 10.000437000 -2.119220000 1.324651000  6 8.955723000 -3.115416000 -0.600655000  7 8.368982000 -4.298629000 -0.160655000  6 7.817551000 -4.823929000 -1.245638000  1 7.266160000 -5.749622000 -1.304425000  6 8.424691000 -5.074583000 -4.578231000  1 9.459086000 -4.794999000 -4.364154000  8 8.122473000 -4.859533000 -5.958138000  1 8.221206000 -5.739581000 -6.368685000  6 8.070371000 -6.521945000 -4.187068000  1 8.504287000 -6.794870000 -3.218569000  8 8.472951000 -7.445867000 -5.198128000  15 9.911178000 -8.305727000 -4.925376000  8 10.072532000 -9.169170000 -6.148500000  8 9.921913000 -8.810274000 -3.504798000  8 11.039953000 -7.065114000 -4.935048000  6 11.351503000 -6.404539000 -6.166026000  1 10.434040000 -6.065920000 -6.667625000  1 11.888937000 -7.082414000 -6.845610000  6 12.231004000 -5.198299000 -5.892545000  1 12.466220000 -4.726749000 -6.860045000  8 11.513639000 -4.235353000 -5.070734000  6 12.387860000 -3.621099000 -4.148537000  1 12.270467000 -2.534469000 -4.180128000  7 12.060732000 -4.045615000 -2.754689000  6 11.387541000 -5.212581000 -2.471937000  1 11.053918000 -5.775672000 -3.337706000  6 12.600580000 -3.254574000 -1.747568000  8 13.232927000 -2.215583000 -2.024908000  7 12.395564000 -3.703245000 -0.471378000  1 12.799790000 -3.089000000 0.275292000  6 11.761888000 -4.901747000 -0.090686000  8 11.761474000 -5.250205000 1.102004000  6 11.195501000 -5.640239000 -1.196365000  1 10.672152000 -6.569157000 -1.001144000  6 13.812279000 -4.058329000 -4.515112000  1 14.470541000 -4.105336000 -3.637514000  8 14.311924000 -3.154480000 -5.492053000  1 14.793391000 -3.705202000 -6.140699000  6 13.550702000 -5.441615000 -5.143382000  1 13.442868000 -6.211862000 -4.370682000  8 14.567207000 -5.795819000 -6.074770000  15 15.882072000 -6.706293000 -5.555818000  8 16.596773000 -7.118786000 -6.821419000  8 15.439434000 -7.685497000 -4.494886000  8 16.747382000 -5.449023000 -4.834547000  6 18.154579000 -5.705078000 -4.667205000  1 18.514330000 -6.338348000 -5.489234000  1 18.336344000 -6.226340000 -3.713111000  6 18.955828000 -4.411639000 -4.660499000  1 19.999333000 -4.671630000 -4.449937000  8 18.913274000 -3.752781000 -5.961629000  6 18.156632000 -2.560219000 -5.888934000  1 18.789851000 -1.672908000 -6.039743000  7 17.179148000 -2.570372000 -6.983478000  6 16.543300000 -1.462214000 -7.509409000  7 16.781403000 -0.170370000 -7.174502000  6 16.009091000 0.684392000 -7.834772000  7 16.186372000 2.028710000 -7.651151000  1 16.717335000 2.264422000 -6.821257000  1 15.405060000 2.645680000 -7.835895000  7 15.060724000 0.289068000 -8.755271000  1 14.506441000 0.992710000 -9.234679000  6 14.768146000 -1.058881000 -9.147652000  8 13.888449000 -1.275944000 -9.988831000  6 15.615147000 -1.974791000 -8.434852000  7 15.678805000 -3.362687000 -8.479326000  6 16.619192000 -3.686949000 -7.600707000  1 16.945607000 -4.691648000 -7.362608000  6 17.501529000 -2.512358000 -4.474124000  1 16.522084000 -2.993247000 -4.522976000  8 17.323826000 -1.204217000 -3.960940000  1 18.208285000 -0.955738000 -3.628295000  6 18.488348000 -3.357436000 -3.657211000  1 18.032738000 -3.798278000 -2.766545000  8 19.561076000 -2.468425000 -3.301288000  15 20.612424000 -2.971676000 -2.075572000  8 21.771804000 -2.010993000 -2.136781000  8 20.746432000 -4.473502000 -2.110628000  8 19.660971000 -2.654412000 -0.735020000  6 19.416550000 -1.282195000 -0.386548000  1 18.714753000 -0.831924000 -1.104119000  1 20.356980000 -0.713386000 -0.406856000  6 18.811400000 -1.220754000 1.005744000  1 18.748476000 -0.166491000 1.306121000  8 17.457722000 -1.745026000 0.968412000  6 17.318876000 -2.873553000 1.810557000  1 16.744193000 -2.640881000 2.713390000  7 16.533873000 -3.882877000 1.070618000  6 16.845173000 -4.137605000 -0.246645000  1 17.708258000 -3.586497000 -0.612660000  6 15.440978000 -4.494285000 1.703851000  8 15.135739000 -4.288249000 2.876219000  7 14.742869000 -5.372703000 0.888534000  1 13.858141000 -5.700821000 1.278463000  6 14.985740000 -5.689991000 -0.465425000  8 14.248386000 -6.483842000 -1.063667000  6 16.134604000 -5.004028000 -1.019084000  1 16.391704000 -5.186299000 -2.057763000  6 18.753644000 -3.339204000 2.166063000  1 19.106199000 -4.010563000 1.372356000  8 18.844119000 -4.038359000 3.384370000  1 18.819031000 -3.374347000 4.103709000  6 19.565104000 -2.023314000 2.076209000  1 20.610423000 -2.210808000 1.810159000  8 19.495505000 -1.298760000 3.312229000  15 20.740712000 -1.436332000 4.436346000  8 20.897654000 -0.070857000 5.062433000  8 21.882926000 -2.220371000 3.838649000  8 19.988230000 -2.467912000 5.544750000  6 19.266105000 -1.874103000 6.633997000  1 19.907759000 -1.162916000 7.173282000  1 19.004744000 -2.700385000 7.308886000  6 18.014950000 -1.133924000 6.171694000  1 18.304418000 -0.310279000 5.512299000  8 17.150454000 -2.025223000 5.399478000  6 15.893764000 -2.170543000 6.050149000  1 15.121396000 -1.565643000 5.562771000  7 15.462073000 -3.575140000 5.920926000  6 16.384873000 -4.592325000 5.976073000  1 17.409541000 -4.262489000 6.110745000  6 14.098701000 -3.829221000 5.703948000  8 13.242805000 -2.949192000 5.632666000  7 13.794136000 -5.178250000 5.603039000  1 12.816669000 -5.380110000 5.416909000  6 14.674276000 -6.281157000 5.596052000  8 14.238006000 -7.423608000 5.411256000  6 16.053347000 -5.903872000 5.831911000  1 16.812771000 -6.677729000 5.861899000  6 16.129104000 -1.732357000 7.516321000  1 16.652759000 -2.557220000 8.026752000  8 14.975092000 -1.460075000 8.261335000  1 14.583958000 -0.594928000 7.953168000  6 17.153302000 -0.591132000 7.317899000  1 17.720866000 -0.369318000 8.232463000  8 16.543977000 0.596110000 6.808253000  15 15.594229000 1.625975000 7.713410000  8 16.311458000 2.014141000 8.982928000  8 14.172596000 1.076762000 7.719933000  8 15.583440000 2.847991000 6.588255000  6 16.796653000 3.602179000 6.405574000  1 17.631432000 3.137813000 6.945510000  1 16.645952000 4.611917000 6.815974000  6 17.171683000 3.763922000 4.942053000  1 18.002177000 4.489430000 4.908576000  8 17.629853000 2.512306000 4.363331000  6 17.207659000 2.423643000 3.009245000  1 18.051999000 2.145230000 2.365847000  7 16.187724000 1.362854000 2.893003000  6 15.788688000 0.720203000 1.734129000  7 16.224909000 0.966284000 0.477866000  6 15.672721000 0.154788000 -0.424599000  7 15.990096000 0.318807000 -1.746374000  1 16.765400000 0.956207000 -1.885492000  1 16.032454000 -0.495613000 -2.356775000  7 14.743012000 -0.812144000 -0.117923000  1 14.266196000 -1.333555000 -0.874312000  6 14.279643000 -1.106471000 1.181747000  8 13.456619000 -2.026557000 1.383613000  6 14.855444000 -0.243023000 2.156962000  7 14.664322000 -0.178905000 3.532145000  6 15.469998000 0.788285000 3.939021000  1 15.627858000 1.105436000 4.956739000  6 16.619713000 3.786092000 2.629166000  1 15.861835000 3.695950000 1.845345000  8 17.674197000 4.654505000 2.211101000  1 17.464492000 5.511630000 2.628585000  6 16.069263000 4.250974000 3.989710000  1 15.116887000 3.762313000 4.224412000  8 15.944523000 5.670714000 4.050132000  15 14.383798000 6.314062000 3.867689000  8 14.574546000 7.804187000 3.969841000  8 13.417194000 5.509920000 4.699022000  8 14.048717000 5.874495000 2.286337000  6 14.756222000 6.514638000 1.219092000  1 15.841737000 6.439692000 1.373905000  1 14.485501000 7.579127000 1.160007000  6 14.398618000 5.854200000 -0.098286000  1 14.924795000 6.396996000 -0.899768000  8 14.841739000 4.467163000 -0.108308000  6 13.897750000 3.653806000 -0.782643000  1 14.402636000 3.010501000 -1.508781000  7 13.204208000 2.748036000 0.181413000  6 13.182674000 2.990501000 1.525997000  1 13.699409000 3.884422000 1.859488000  6 12.493148000 1.661376000 -0.371488000  8 12.509825000 1.520142000 -1.617345000  7 11.837949000 0.821267000 0.467167000  6 11.861920000 1.017938000 1.802140000  7 11.218917000 0.125405000 2.573319000  1 11.417122000 0.115415000 3.563956000  1 10.840447000 -0.727927000 2.134828000  6 12.525597000 2.149809000 2.374748000  1 12.521442000 2.334067000 3.443692000  6 12.878631000 4.596336000 -1.433151000  1 11.892923000 4.129472000 -1.531453000  8 13.372465000 5.015885000 -2.706886000  1 13.176236000 5.971714000 -2.742466000  6 12.906490000 5.776939000 -0.447098000  1 12.316407000 5.559293000 0.450528000  8 12.465898000 6.986893000 -1.059460000  15 10.866768000 7.450651000 -0.717298000  8 10.663624000 8.715747000 -1.510901000  8 10.626818000 7.313483000 0.763770000  8 10.025171000 6.177755000 -1.407315000  6 10.033092000 6.049507000 -2.835748000  1 11.046020000 5.825388000 -3.197185000  1 9.695825000 6.981388000 -3.307718000  6 9.105822000 4.927466000 -3.251409000  1 9.050857000 4.920461000 -4.351853000  8 9.637702000 3.652221000 -2.800202000  6 8.579288000 2.790648000 -2.411521000  1 8.679556000 1.819448000 -2.909146000  7 8.663757000 2.522739000 -0.970239000  6 8.046009000 1.451346000 -0.346490000  7 7.290316000 0.492250000 -0.910860000  6 6.841582000 -0.384429000 0.009664000  1 6.227733000 -1.204938000 -0.361867000  7 7.068414000 -0.389107000 1.340723000  6 7.841081000 0.588918000 1.872938000  7 8.047605000 0.595554000 3.223590000  1 8.882648000 1.077535000 3.535212000  1 7.864567000 -0.286012000 3.689402000  6 8.359959000 1.593310000 1.019767000  7 9.133967000 2.731554000 1.234739000  6 9.290808000 3.261146000 0.027736000  1 9.822912000 4.174948000 -0.210937000  6 7.264443000 3.499383000 -2.760546000  1 6.449609000 3.237057000 -2.072763000  8 6.918220000 3.172465000 -4.114514000  1 6.475033000 3.974890000 -4.457391000  6 7.680352000 4.976880000 -2.682881000  1 7.683748000 5.306516000 -1.639030000  8 6.847037000 5.801218000 -3.486448000  15 5.597058000 6.689593000 -2.736126000  8 5.553058000 6.309070000 -1.270913000  8 4.402878000 6.604358000 -3.648849000  8 6.208682000 8.221404000 -2.930984000  6 7.485678000 8.546832000 -2.350178000  1 8.193353000 8.744465000 -3.165417000  1 7.884353000 7.726524000 -1.742154000  6 7.368807000 9.770785000 -1.457185000  1 8.372645000 10.022339000 -1.086510000  8 6.849004000 10.886181000 -2.223725000  6 5.761207000 11.496797000 -1.543493000  1 5.894841000 12.583860000 -1.549283000  7 4.499856000 11.271336000 -2.265772000  6 3.893283000 12.222581000 -3.066223000  7 4.326522000 13.489746000 -3.256748000  6 3.520732000 14.202936000 -4.034284000  7 3.800601000 15.515851000 -4.275015000  1 4.741425000 15.797691000 -4.029102000  1 3.429496000 15.948665000 -5.111237000  7 2.360786000 13.692010000 -4.581206000  1 1.744686000 14.305910000 -5.106092000  6 1.853353000 12.375202000 -4.391077000  8 0.735538000 12.085654000 -4.861752000  6 2.755256000 11.592263000 -3.603859000  7 2.666058000 10.277087000 -3.153776000  6 3.718267000 10.119514000 -2.370109000  1 3.995460000 9.201268000 -1.871760000  6 5.766922000 10.977364000 -0.094952000  1 4.750883000 10.889558000 0.311386000  8 6.562416000 11.869664000 0.657577000  1 6.497505000 11.482091000 1.582068000  6 6.416824000 9.583483000 -0.265219000  1 5.642834000 8.858824000 -0.549767000  8 7.173586000 9.047007000 0.826894000  15 6.877346000 9.147041000 2.468449000  8 6.168949000 10.460404000 2.796258000  8 8.181801000 8.821002000 3.148246000  8 5.882847000 7.843820000 2.769827000  6 4.906830000 7.382180000 1.823869000  1 4.895558000 6.284042000 1.879391000  1 5.192035000 7.637283000 0.801146000  6 3.496992000 7.861010000 2.130357000  1 3.115645000 7.385375000 3.050816000  8 3.469811000 9.292392000 2.271394000  6 2.120923000 9.736843000 2.081936000  1 1.655571000 9.939164000 3.058574000  7 2.204138000 11.015521000 1.359808000  6 1.515786000 11.508925000 0.258546000  7 0.625469000 10.848338000 -0.521342000  6 0.175776000 11.565763000 -1.540910000  7 -0.670798000 11.008961000 -2.465281000  1 -0.706182000 9.998915000 -2.367684000  1 -0.460381000 11.295745000 -3.427180000  7 0.495083000 12.894306000 -1.719770000  1 0.095411000 13.387210000 -2.512692000  6 1.459491000 13.635091000 -0.970374000  8 1.714949000 14.805716000 -1.279314000  6 1.991843000 12.825192000 0.091114000  7 2.951831000 13.136143000 1.047425000  6 3.049564000 12.044044000 1.783662000  1 3.743521000 11.879061000 2.599375000  6 1.343530000 8.595876000 1.392506000  1 0.791728000 8.939906000 0.512618000  8 0.481346000 8.015991000 2.359816000  1 0.000050000 7.320919000 1.833935000  1 2.942998000 7.976588000 0.046608000  6 2.497747000 7.634203000 0.994480000  8 2.122835000 6.267830000 0.896689000  15 0.873049000 5.911212000 -0.174745000  8 1.237722000 6.388876000 -1.560110000  8 -0.443071000 6.313305000 0.482416000  8 0.963768000 4.278236000 -0.012990000  6 2.055438000 3.560550000 -0.625039000  1 2.966617000 4.174382000 -0.645781000  1 1.796303000 3.294924000 -1.657427000  6 2.313125000 2.277817000 0.143582000  1 2.931778000 1.624807000 -0.487400000  8 3.062695000 2.548029000 1.367716000  6 2.545777000 1.767027000 2.429218000  1 3.368136000 1.292228000 2.980690000  7 1.832205000 2.667051000 3.368935000  6 1.285573000 2.305402000 4.588961000  7 1.272038000 1.089163000 5.172034000  6 0.646591000 1.128711000 6.364220000  1 0.595027000 0.179941000 6.903854000  7 0.074443000 2.178177000 6.991138000  6 0.105460000 3.387032000 6.373871000  7 -0.510832000 4.441977000 6.975385000  1 -0.288541000 5.371278000 6.644253000  1 -0.740567000 4.346390000 7.956393000  6 0.736818000 3.495730000 5.111122000  7 0.945014000 4.563016000 4.243830000  6 1.603349000 4.028440000 3.219943000  1 1.932737000 4.561605000 2.337923000  6 1.597344000 0.717332000 1.815237000  1 0.801633000 0.441195000 2.521667000  8 2.358636000 -0.411621000 1.429466000  1 1.726067000 -1.178252000 1.431934000  1 0.336511000 2.257711000 0.995971000  6 1.051682000 1.519992000 0.605308000  8 0.394907000 0.790726000 -0.402078000  1 1.089639000 0.383368000 -0.987048000 | 1 -29.285097000 0.679765000 -2.777882000  8 -29.991819000 1.350440000 -2.965760000  6 -30.366501000 1.098748000 -4.314018000  1 -31.342549000 1.571904000 -4.490994000  1 -30.478074000 0.016907000 -4.498789000  6 -29.365909000 1.624664000 -5.344596000  1 -29.571380000 1.158446000 -6.321337000  8 -29.470529000 3.069691000 -5.495630000  6 -28.184288000 3.632764000 -5.668312000  1 -28.158879000 4.278583000 -6.552868000  7 -27.874944000 4.513177000 -4.496747000  6 -28.574231000 4.419656000 -3.313620000  1 -29.265163000 3.583085000 -3.250716000  6 -26.892494000 5.487307000 -4.701375000  8 -26.234934000 5.561069000 -5.741463000  7 -26.734090000 6.360165000 -3.636331000  1 -26.033302000 7.083041000 -3.769262000  6 -27.456761000 6.396454000 -2.420257000  8 -27.237742000 7.293590000 -1.598085000  6 -28.404278000 5.309171000 -2.296645000  1 -28.966717000 5.208731000 -1.375961000  6 -27.177438000 2.475818000 -5.791940000  1 -26.197623000 2.738131000 -5.373425000  8 -27.087053000 2.103791000 -7.166320000  1 -26.911871000 1.146004000 -7.159310000  6 -27.898225000 1.401901000 -4.976160000  1 -27.753809000 1.614818000 -3.911914000  8 -27.473267000 0.081186000 -5.303202000  8 -28.114512000 -0.647833000 -2.874714000  15 -27.132452000 -0.931224000 -4.001530000  8 -26.938786000 -2.300364000 -4.591381000  8 -25.704999000 -0.277376000 -3.463801000  6 -24.634326000 -0.051668000 -4.395348000  1 -25.035516000 0.230439000 -5.377266000  1 -24.025746000 -0.961577000 -4.506607000  6 -23.750361000 1.053612000 -3.843220000  1 -22.940509000 1.255681000 -4.563061000  8 -24.539384000 2.250725000 -3.657638000  6 -24.182457000 2.909802000 -2.456329000  1 -23.813095000 3.924315000 -2.655308000  7 -25.382921000 3.070603000 -1.624765000  6 -25.573336000 4.110217000 -0.737993000  7 -24.770018000 5.190009000 -0.599701000  6 -25.206460000 6.041985000 0.320915000  7 -24.474044000 7.158819000 0.607822000  1 -23.786153000 7.390599000 -0.098795000  1 -24.959067000 7.959841000 0.993379000  7 -26.338783000 5.816497000 1.072930000  1 -26.619217000 6.504024000 1.765314000  6 -27.211767000 4.694691000 0.970058000  8 -28.182375000 4.605334000 1.745323000  6 -26.758021000 3.794089000 -0.044558000  7 -27.281673000 2.588315000 -0.495810000  6 -26.435045000 2.174892000 -1.427190000  1 -26.501219000 1.254634000 -1.995365000  6 -23.084314000 2.089176000 -1.738286000  1 -23.354906000 1.960725000 -0.684732000  8 -21.848992000 2.773365000 -1.855387000  1 -21.310035000 2.419981000 -1.120784000  6 -23.119412000 0.724601000 -2.481099000  1 -23.760483000 0.024741000 -1.933759000  8 -21.829270000 0.163739000 -2.690754000  15 -21.476085000 -1.382826000 -2.141142000  8 -20.377270000 -1.877562000 -3.048961000  8 -22.746519000 -2.150714000 -1.864510000  8 -20.852179000 -1.029572000 -0.626095000  6 -19.705148000 -0.179111000 -0.529393000  1 -19.621749000 0.469651000 -1.412170000  1 -18.792980000 -0.790126000 -0.452052000  6 -19.826195000 0.682322000 0.715517000  1 -18.917612000 1.296237000 0.816056000  8 -20.972589000 1.568502000 0.581423000  6 -21.714794000 1.643776000 1.799520000  1 -21.892146000 2.691559000 2.066631000  7 -23.039895000 1.038202000 1.624474000  6 -24.224527000 1.561530000 2.118256000  7 -24.362264000 2.737466000 2.780417000  6 -25.624328000 2.993304000 3.108041000  7 -25.930898000 4.125375000 3.819109000  1 -25.189724000 4.816885000 3.803241000  1 -26.854458000 4.515122000 3.645418000  7 -26.666357000 2.136924000 2.829606000  1 -27.614867000 2.456541000 3.004639000  6 -26.577828000 0.887165000 2.139179000  8 -27.593519000 0.207899000 1.958090000  6 -25.213590000 0.626970000 1.768893000  7 -24.659474000 -0.442343000 1.074038000  6 -23.364680000 -0.174901000 1.011403000  1 -22.612834000 -0.758902000 0.494512000  6 -20.878429000 0.926965000 2.869781000  1 -21.512270000 0.427793000 3.614840000  8 -20.002710000 1.871081000 3.471147000  1 -19.164840000 1.374632000 3.573192000  6 -20.048361000 -0.076956000 2.033426000  1 -20.607732000 -1.004153000 1.856039000  8 -18.787171000 -0.332315000 2.639602000  15 -18.750189000 -1.604720000 3.789177000  8 -18.535583000 -2.898509000 3.038090000  8 -19.892780000 -1.417077000 4.756803000  8 -17.374865000 -1.089795000 4.551986000  6 -16.181311000 -0.863592000 3.781145000  1 -16.048092000 0.222604000 3.664184000  1 -16.236352000 -1.324217000 2.787944000  6 -14.972438000 -1.455007000 4.491778000  1 -14.070244000 -1.164518000 3.935676000  8 -14.851385000 -0.904067000 5.833635000  6 -14.960526000 -1.942024000 6.794274000  1 -14.312682000 -1.679651000 7.638094000  7 -16.339238000 -2.028400000 7.323187000  6 -17.352435000 -2.965843000 7.136966000  7 -17.305004000 -4.171660000 6.537991000  6 -18.500850000 -4.788134000 6.552868000  1 -18.530235000 -5.771136000 6.077181000  7 -19.669734000 -4.360072000 7.068875000  6 -19.688086000 -3.145306000 7.670753000  7 -20.852511000 -2.711374000 8.228171000  1 -20.931692000 -1.723688000 8.431915000  1 -21.699705000 -3.181663000 7.935137000  6 -18.491658000 -2.393545000 7.737385000  7 -18.209024000 -1.139576000 8.267283000  6 -16.927507000 -0.962698000 7.993359000  1 -16.344899000 -0.082797000 8.244625000  6 -14.488798000 -3.218483000 6.094923000  1 -14.903262000 -4.133031000 6.537255000  8 -13.062912000 -3.205967000 6.130245000  1 -12.797934000 -3.601921000 5.278479000  6 -14.995395000 -2.994247000 4.653638000  1 -16.012779000 -3.386658000 4.546314000  8 -14.086697000 -3.676364000 3.779174000  15 -14.602381000 -4.022115000 2.213453000  8 -14.829933000 -2.694381000 1.506507000  8 -15.615191000 -5.136651000 2.218391000  8 -13.091042000 -4.555504000 1.749203000  6 -12.928477000 -5.756351000 0.977275000  1 -13.618045000 -6.533094000 1.332167000  1 -11.903012000 -6.080013000 1.167397000  6 -13.129972000 -5.575237000 -0.535810000  1 -12.327472000 -6.106658000 -1.068509000  8 -14.403682000 -6.143764000 -0.937913000  6 -14.947826000 -5.397070000 -2.033776000  1 -15.172916000 -6.083720000 -2.857274000  7 -16.220401000 -4.791109000 -1.620368000  6 -17.365838000 -5.532282000 -1.373729000  7 -17.521382000 -6.852176000 -1.635676000  6 -18.718015000 -7.309442000 -1.282647000  7 -19.049082000 -8.607812000 -1.555843000  1 -18.257664000 -9.200481000 -1.775441000  1 -19.763949000 -9.052479000 -0.993149000  7 -19.681961000 -6.517856000 -0.696262000  1 -20.577411000 -6.925159000 -0.443351000  6 -19.563061000 -5.124035000 -0.380077000  8 -20.500131000 -4.545817000 0.181795000  6 -18.279519000 -4.633071000 -0.799145000  7 -17.714157000 -3.366053000 -0.688254000  6 -16.493249000 -3.496205000 -1.171213000  1 -15.757230000 -2.705693000 -1.224451000  6 -13.888971000 -4.357850000 -2.431936000  1 -14.334101000 -3.431537000 -2.820309000  8 -13.027286000 -4.956110000 -3.388723000  1 -12.314006000 -4.290553000 -3.547001000  6 -13.204253000 -4.140375000 -1.063843000  1 -13.880153000 -3.559662000 -0.423349000  8 -11.914564000 -3.532771000 -1.060748000  15 -11.628893000 -2.260428000 -2.109273000  8 -12.805660000 -1.316861000 -2.103201000  8 -11.102643000 -2.882094000 -3.406956000  8 -10.418585000 -1.511820000 -1.270790000  6 -9.096793000 -2.101648000 -1.257649000  1 -8.413619000 -1.445752000 -1.816085000  1 -9.117226000 -3.092518000 -1.714628000  6 -8.579248000 -2.239317000 0.163810000  1 -8.461137000 -1.257712000 0.647031000  8 -9.518290000 -3.021663000 0.952467000  6 -8.850029000 -4.000037000 1.730491000  1 -9.122236000 -3.886636000 2.787575000  7 -9.275359000 -5.355666000 1.354680000  6 -9.409455000 -5.923696000 0.092443000  7 -9.364880000 -5.282947000 -1.092969000  6 -9.673910000 -6.051938000 -2.131341000  7 -9.733469000 -5.494044000 -3.374725000  1 -10.020788000 -4.505060000 -3.370777000  1 -10.203781000 -6.037310000 -4.088966000  7 -9.909030000 -7.407900000 -2.004666000  1 -10.130555000 -7.955719000 -2.831251000  6 -9.910797000 -8.158397000 -0.783098000  8 -10.129439000 -9.374536000 -0.802955000  6 -9.658554000 -7.287590000 0.331765000  7 -9.662605000 -7.556353000 1.699018000  6 -9.427603000 -6.387517000 2.271198000  1 -9.326903000 -6.211767000 3.334392000  6 -7.342803000 -3.782951000 1.556028000  1 -6.812395000 -4.741004000 1.497233000  8 -6.900657000 -3.002660000 2.657009000  1 -5.956178000 -2.823511000 2.448392000  6 -7.253701000 -3.017558000 0.214305000  1 -7.212930000 -3.746861000 -0.604970000  8 -6.142918000 -2.122627000 0.133762000  15 -4.609060000 -2.798298000 0.001024000  8 -3.669414000 -1.687597000 -0.389806000  8 -4.710822000 -4.117221000 -0.728889000  8 -4.379275000 -3.164553000 1.645028000  6 -3.262742000 -2.643970000 2.386875000  1 -3.654066000 -2.260290000 3.338923000  1 -2.787181000 -1.822030000 1.836209000  6 -2.228031000 -3.724879000 2.674505000  1 -1.426090000 -3.272829000 3.272159000  8 -2.819364000 -4.807109000 3.460657000  6 -2.821526000 -6.012377000 2.715366000  1 -2.072344000 -6.724782000 3.094274000  7 -4.128384000 -6.657426000 2.872476000  6 -4.405061000 -8.000861000 2.657943000  7 -3.533343000 -9.003088000 2.432333000  6 -4.176262000 -10.175173000 2.272149000  1 -3.537700000 -11.044832000 2.097494000  7 -5.499968000 -10.428100000 2.283300000  6 -6.358016000 -9.395254000 2.508541000  7 -7.688730000 -9.663311000 2.529135000  1 -8.369443000 -8.912221000 2.374208000  1 -7.950586000 -10.578632000 2.182879000  6 -5.811552000 -8.104379000 2.740870000  7 -6.379197000 -6.865057000 3.020077000  6 -5.346997000 -6.032811000 3.082452000  1 -5.401924000 -4.971641000 3.283518000  6 -2.506177000 -5.635208000 1.239682000  1 -3.432167000 -5.350022000 0.728714000  8 -1.893266000 -6.661707000 0.489357000  1 -0.973611000 -6.687996000 0.811153000  6 -1.625371000 -4.394075000 1.437130000  1 -1.603194000 -3.734093000 0.562424000  8 -0.309684000 -4.900372000 1.744138000  15 1.002321000 -3.905708000 1.463697000  8 2.155924000 -4.542474000 2.192683000  8 0.582600000 -2.457890000 1.667783000  8 1.159844000 -4.039083000 -0.185315000  6 1.455091000 -5.306816000 -0.801412000  1 0.751109000 -5.409744000 -1.637911000  1 1.282446000 -6.140457000 -0.106250000  6 2.882924000 -5.412319000 -1.343174000  1 2.939850000 -6.344004000 -1.919706000  8 3.179537000 -4.297975000 -2.236710000  6 4.194714000 -3.475514000 -1.658264000  1 5.145227000 -3.604096000 -2.187826000  7 3.822519000 -2.076268000 -1.810961000  6 4.521672000 -1.089571000 -2.495768000  7 5.705301000 -1.237989000 -3.131995000  6 6.152295000 -0.097085000 -3.659382000  7 7.320642000 -0.094581000 -4.365981000  1 8.016988000 -0.779315000 -4.040687000  1 7.704016000 0.816358000 -4.590892000  7 5.459401000 1.090178000 -3.590270000  1 5.931033000 1.951232000 -3.905055000  6 4.237917000 1.307957000 -2.900598000  8 3.763423000 2.453902000 -2.835091000  6 3.745384000 0.075564000 -2.354840000  7 2.599277000 -0.201769000 -1.616347000  6 2.675909000 -1.483721000 -1.305389000  1 1.949794000 -2.045439000 -0.732234000  6 4.310700000 -3.912235000 -0.176230000  1 3.499725000 -3.454625000 0.394645000  8 5.505242000 -3.542980000 0.468160000  1 6.243994000 -4.061972000 0.092217000  6 4.005702000 -5.411453000 -0.296643000  1 3.702797000 -5.858185000 0.656813000  8 5.208371000 -5.999442000 -0.810628000  15 5.406716000 -7.645328000 -1.069601000  8 4.079177000 -8.266271000 -1.447519000  8 6.290324000 -8.210276000 0.014847000  8 6.402755000 -7.495577000 -2.415016000  6 5.800918000 -7.250592000 -3.694999000  1 4.752091000 -6.945862000 -3.584689000  1 5.819703000 -8.183303000 -4.280141000  6 6.535673000 -6.190680000 -4.501975000  1 6.122489000 -6.223995000 -5.524040000  8 6.292083000 -4.865630000 -3.953233000  6 7.467001000 -4.070801000 -4.045609000  1 7.212942000 -3.077524000 -4.428601000  7 8.022448000 -3.885924000 -2.688197000  6 8.777904000 -2.826107000 -2.218012000  7 9.288447000 -1.806289000 -2.947568000  6 10.022519000 -0.957285000 -2.211034000  7 10.572204000 0.130519000 -2.802815000  1 10.609830000 0.096717000 -3.813256000  1 11.337096000 0.634923000 -2.320315000  7 10.231495000 -1.108501000 -0.861701000  1 10.839176000 -0.410886000 -0.356998000  6 9.695248000 -2.140086000 -0.061899000  8 9.920246000 -2.182248000 1.164815000  6 8.931841000 -3.066019000 -0.841356000  7 8.304168000 -4.254929000 -0.477084000  6 7.786587000 -4.720515000 -1.604893000  1 7.207301000 -5.622589000 -1.718938000  6 8.447326000 -4.836471000 -4.931635000  1 9.484999000 -4.582665000 -4.701247000  8 8.156633000 -4.564827000 -6.304139000  1 8.247504000 -5.429822000 -6.747336000  6 8.068233000 -6.291282000 -4.596449000  1 8.491983000 -6.605288000 -3.636152000  8 8.465085000 -7.182833000 -5.638242000  15 9.884273000 -8.078906000 -5.380201000  8 10.057578000 -8.883129000 -6.641372000  8 9.857379000 -8.653907000 -3.986836000  8 11.032436000 -6.858147000 -5.304086000  6 11.383442000 -6.141418000 -6.492426000  1 10.483610000 -5.758006000 -6.994055000  1 11.921569000 -6.794396000 -7.195427000  6 12.280497000 -4.969159000 -6.138300000  1 12.548433000 -4.455244000 -7.075236000  8 11.561797000 -4.033168000 -5.286940000  6 12.423046000 -3.484566000 -4.312640000  1 12.328564000 -2.395471000 -4.292608000  7 12.050351000 -3.970512000 -2.951118000  6 11.344159000 -5.133653000 -2.744566000  1 11.023288000 -5.645897000 -3.645896000  6 12.577628000 -3.240706000 -1.892283000  8 13.239922000 -2.204606000 -2.101226000  7 12.324761000 -3.744648000 -0.645597000  1 12.718403000 -3.173959000 0.140124000  6 11.651488000 -4.943403000 -0.341254000  8 11.603699000 -5.345735000 0.833480000  6 11.105730000 -5.617383000 -1.497178000  1 10.556626000 -6.541963000 -1.361341000  6 13.847334000 -3.933212000 -4.663943000  1 14.480049000 -4.039886000 -3.772888000  8 14.391512000 -2.991379000 -5.578948000  1 14.879557000 -3.518874000 -6.241853000  6 13.576224000 -5.276376000 -5.370597000  1 13.434922000 -6.084017000 -4.642862000  8 14.610238000 -5.599906000 -6.294004000  15 15.898407000 -6.555564000 -5.790299000  8 16.643617000 -6.906841000 -7.056908000  8 15.413457000 -7.586362000 -4.799030000  8 16.758869000 -5.353441000 -4.975033000  6 18.155414000 -5.644919000 -4.778233000  1 18.529360000 -6.242296000 -5.620589000  1 18.297149000 -6.218241000 -3.847553000  6 18.980040000 -4.369999000 -4.680127000  1 20.011761000 -4.662163000 -4.454383000  8 18.988803000 -3.642589000 -5.944971000  6 18.249170000 -2.442737000 -5.831951000  1 18.900513000 -1.560276000 -5.921655000  7 17.300460000 -2.381476000 -6.950342000  6 16.682519000 -1.240192000 -7.424462000  7 16.914791000 0.027951000 -7.004940000  6 16.161102000 0.924178000 -7.631025000  7 16.336653000 2.254310000 -7.362505000  1 16.846319000 2.438656000 -6.506703000  1 15.563922000 2.885992000 -7.533432000  7 15.234776000 0.589013000 -8.596644000  1 14.691438000 1.322260000 -9.042615000  6 14.948752000 -0.731130000 -9.077605000  8 14.087932000 -0.893480000 -9.950188000  6 15.777082000 -1.691544000 -8.402872000  7 15.836120000 -3.074550000 -8.528662000  6 16.752066000 -3.456026000 -7.647171000  1 17.064925000 -4.475509000 -7.458395000  6 17.557007000 -2.453680000 -4.434183000  1 16.569836000 -2.910505000 -4.533320000  8 17.391626000 -1.169570000 -3.859957000  1 18.270904000 -0.957409000 -3.490423000  6 18.503991000 -3.360187000 -3.635924000  1 18.015154000 -3.837888000 -2.782649000  8 19.582791000 -2.513473000 -3.203939000  15 20.586893000 -3.100125000 -1.975833000  8 21.763366000 -2.158871000 -1.951868000  8 20.696695000 -4.600182000 -2.086755000  8 19.600309000 -2.835294000 -0.648845000  6 19.369787000 -1.476917000 -0.240881000  1 18.703580000 -0.977398000 -0.959844000  1 20.321092000 -0.927637000 -0.200144000  6 18.715926000 -1.468579000 1.130449000  1 18.662357000 -0.428210000 1.477360000  8 17.354332000 -1.961476000 1.020817000  6 17.162334000 -3.123623000 1.804551000  1 16.559460000 -2.919252000 2.695744000  7 16.386381000 -4.082415000 0.991035000  6 16.736436000 -4.277244000 -0.326673000  1 17.618246000 -3.722251000 -0.637624000  6 15.266516000 -4.708900000 1.558986000  8 14.924182000 -4.554646000 2.729003000  7 14.586386000 -5.539826000 0.680887000  1 13.685967000 -5.873788000 1.027305000  6 14.864987000 -5.789705000 -0.680218000  8 14.134643000 -6.539607000 -1.340565000  6 16.039338000 -5.093738000 -1.163188000  1 16.326422000 -5.227416000 -2.201412000  6 18.573639000 -3.635419000 2.189734000  1 18.941486000 -4.278020000 1.379420000  8 18.605833000 -4.390103000 3.377458000  1 18.564482000 -3.758048000 4.124220000  6 19.413460000 -2.334534000 2.189423000  1 20.464198000 -2.531840000 1.953725000  8 19.310551000 -1.665990000 3.454288000  15 20.515876000 -1.866960000 4.611728000  8 20.661859000 -0.533123000 5.305106000  8 21.671710000 -2.632626000 4.016072000  8 19.719318000 -2.941782000 5.645389000  6 18.970164000 -2.393114000 6.739929000  1 19.602000000 -1.716472000 7.332592000  1 18.679026000 -3.247826000 7.365489000  6 17.742223000 -1.616049000 6.276633000  1 18.061249000 -0.764263000 5.668646000  8 16.894015000 -2.458314000 5.434535000  6 15.609210000 -2.603270000 6.028168000  1 14.870948000 -1.955189000 5.543504000  7 15.149837000 -3.987328000 5.810571000  6 16.044574000 -5.029932000 5.851135000  1 17.070664000 -4.734097000 6.042305000  6 13.790586000 -4.193679000 5.527035000  8 12.960846000 -3.288197000 5.465685000  7 13.457067000 -5.527436000 5.347013000  1 12.483642000 -5.693842000 5.111342000  6 14.309502000 -6.651540000 5.319300000  8 13.853089000 -7.771452000 5.060084000  6 15.686778000 -6.323454000 5.628243000  1 16.424813000 -7.118029000 5.649146000  6 15.798013000 -2.245804000 7.522202000  1 16.285962000 -3.106894000 8.007549000  8 14.621800000 -1.986934000 8.236951000  1 14.261903000 -1.097417000 7.962795000  6 16.851198000 -1.118363000 7.421343000  1 17.390935000 -0.959138000 8.365337000  8 16.283976000 0.108062000 6.958339000  15 15.327054000 1.111432000 7.885538000  8 16.008652000 1.415128000 9.196991000  8 13.894246000 0.597032000 7.816000000  8 15.384212000 2.390739000 6.827579000  6 16.620830000 3.122101000 6.726782000  1 17.424198000 2.610206000 7.271437000  1 16.478846000 4.113112000 7.183394000  6 17.052518000 3.350318000 5.287749000  1 17.899219000 4.056864000 5.322330000  8 17.504312000 2.120782000 4.658712000  6 17.130989000 2.115090000 3.286939000  1 17.993195000 1.853798000 2.660216000  7 16.095293000 1.084696000 3.076941000  6 15.719945000 0.514804000 1.872524000  7 16.202241000 0.814966000 0.645018000  6 15.665112000 0.061885000 -0.315614000  7 16.032464000 0.284288000 -1.615606000  1 16.825657000 0.909644000 -1.695825000  1 16.077399000 -0.498780000 -2.265611000  7 14.706244000 -0.898874000 -0.089694000  1 14.248851000 -1.376530000 -0.885336000  6 14.198666000 -1.252760000 1.178070000  8 13.359646000 -2.171236000 1.307777000  6 14.755265000 -0.449695000 2.214006000  7 14.523796000 -0.457790000 3.584212000  6 15.335008000 0.468417000 4.067523000  1 15.469162000 0.724535000 5.105514000  6 16.584592000 3.508391000 2.961284000  1 15.852593000 3.476678000 2.148955000  8 17.670718000 4.373871000 2.625910000  1 17.464110000 5.212250000 3.081241000  6 15.996416000 3.913554000 4.325128000  1 15.025309000 3.436969000 4.499930000  8 15.902585000 5.330858000 4.457591000  15 14.365369000 6.024534000 4.264627000  8 14.590810000 7.501096000 4.455013000  8 13.353174000 5.202668000 5.021052000  8 14.068507000 5.681733000 2.651802000  6 14.830601000 6.354444000 1.643824000  1 15.907341000 6.247648000 1.836318000  1 14.584785000 7.426493000 1.624497000  6 14.511011000 5.763288000 0.284256000  1 15.082115000 6.327424000 -0.470447000  8 14.920748000 4.367530000 0.229437000  6 13.985331000 3.607831000 -0.515683000  1 14.502805000 2.986684000 -1.252040000  7 13.236896000 2.675471000 0.378609000  6 13.163583000 2.859166000 1.731039000  1 13.683178000 3.726742000 2.124769000  6 12.529986000 1.628234000 -0.250636000  8 12.596620000 1.539816000 -1.499937000  7 11.822396000 0.768572000 0.521937000  6 11.794954000 0.903994000 1.865636000  7 11.109582000 -0.011699000 2.564672000  1 11.206180000 -0.035326000 3.569345000  1 10.720653000 -0.832589000 2.075589000  6 12.455929000 1.996477000 2.514403000  1 12.411318000 2.134879000 3.589325000  6 13.012669000 4.601756000 -1.159956000  1 12.020413000 4.163481000 -1.310308000  8 13.560471000 5.060355000 -2.397652000  1 13.390052000 6.021952000 -2.398498000  6 13.032624000 5.742088000 -0.126666000  1 12.400288000 5.507964000 0.737482000  8 12.653048000 6.986726000 -0.710568000  15 11.056206000 7.491196000 -0.420381000  8 10.919046000 8.770910000 -1.204416000  8 10.757504000 7.344351000 1.049048000  8 10.204750000 6.251442000 -1.157763000  6 10.249435000 6.145850000 -2.587677000  1 11.266911000 5.906838000 -2.925754000  1 9.944521000 7.092166000 -3.052613000  6 9.309938000 5.050998000 -3.047242000  1 9.282592000 5.065882000 -4.148634000  8 9.803312000 3.756247000 -2.608341000  6 8.718271000 2.909601000 -2.259387000  1 8.809817000 1.946002000 -2.773750000  7 8.766164000 2.616773000 -0.820721000  6 8.125603000 1.543843000 -0.221111000  7 7.398013000 0.578389000 -0.811525000  6 6.919288000 -0.298321000 0.093390000  1 6.332011000 -1.127253000 -0.301430000  7 7.084440000 -0.294980000 1.433248000  6 7.824621000 0.692838000 1.993495000  7 7.958815000 0.717212000 3.351192000  1 8.741470000 1.244321000 3.717240000  1 7.751115000 -0.150403000 3.831126000  6 8.383124000 1.689340000 1.156453000  7 9.149147000 2.826118000 1.401608000  6 9.353015000 3.355471000 0.201514000  1 9.896799000 4.267717000 -0.016378000  6 7.427920000 3.654540000 -2.625907000  1 6.589905000 3.398832000 -1.964113000  8 7.107426000 3.360706000 -3.994421000  1 6.695206000 4.181375000 -4.333379000  6 7.871469000 5.121578000 -2.514090000  1 7.853441000 5.436110000 -1.465833000  8 7.075520000 5.973676000 -3.327195000  15 5.831679000 6.888125000 -2.596785000  8 5.729810000 6.477727000 -1.142665000  8 4.665571000 6.862203000 -3.548601000  8 6.497623000 8.402456000 -2.740784000  6 7.762017000 8.677502000 -2.109890000  1 8.506861000 8.858244000 -2.895298000  1 8.108303000 7.837893000 -1.496114000  6 7.655501000 9.895743000 -1.209037000  1 8.652215000 10.101129000 -0.793543000  8 7.217742000 11.040098000 -1.984085000  6 6.135849000 11.696759000 -1.337671000  1 6.322017000 12.776018000 -1.323007000  7 4.896725000 11.540360000 -2.114904000  6 4.372784000 12.523109000 -2.935881000  7 4.857408000 13.778286000 -3.078297000  6 4.129716000 14.526175000 -3.899302000  7 4.465360000 15.833621000 -4.098159000  1 5.395345000 16.081013000 -3.783437000  1 4.168060000 16.290746000 -4.950932000  7 2.996716000 14.058248000 -4.533551000  1 2.439915000 14.695227000 -5.095509000  6 2.437520000 12.754988000 -4.400995000  8 1.354613000 12.503035000 -4.964621000  6 3.253083000 11.937694000 -3.556463000  7 3.094344000 10.619583000 -3.135933000  6 4.087020000 10.416610000 -2.288134000  1 4.300193000 9.483628000 -1.785920000  6 6.057686000 11.160146000 0.102245000  1 5.023384000 11.112312000 0.463207000  8 6.860302000 12.006314000 0.900522000  1 6.739553000 11.610616000 1.815727000  6 6.648350000 9.738463000 -0.058726000  1 5.852147000 9.052474000 -0.375503000  8 7.341881000 9.157416000 1.052821000  15 6.992459000 9.248656000 2.682297000  8 6.321635000 10.582368000 3.008316000  8 8.260249000 8.864515000 3.400719000  8 5.935594000 7.985886000 2.935716000  6 5.009786000 7.524248000 1.940203000  1 4.919361000 6.435808000 2.067712000  1 5.390296000 7.692303000 0.929121000  6 3.618078000 8.107651000 2.105256000  1 3.184082000 7.797496000 3.072924000  8 3.660483000 9.541611000 2.023026000  6 2.306839000 9.982130000 1.866561000  1 1.847720000 10.128616000 2.857656000  7 2.375224000 11.290289000 1.212509000  6 1.738481000 11.808399000 0.091892000  7 0.893483000 11.163316000 -0.748255000  6 0.495413000 11.905620000 -1.771490000  7 -0.295247000 11.371180000 -2.757831000  1 -0.333198000 10.359008000 -2.686534000  1 -0.016645000 11.676978000 -3.695678000  7 0.815187000 13.239787000 -1.900394000  1 0.461389000 13.747389000 -2.705812000  6 1.730947000 13.967001000 -1.081605000  8 1.998424000 15.145216000 -1.348861000  6 2.207451000 13.133884000 -0.011763000  7 3.110307000 13.427659000 1.003357000  6 3.181849000 12.316307000 1.712099000  1 3.826018000 12.138809000 2.565196000  6 1.528552000 8.847766000 1.156002000  1 1.132459000 9.160555000 0.184097000  8 0.498467000 8.430244000 2.035800000  1 0.049530000 7.688906000 1.544406000  1 3.145779000 7.918525000 0.010261000  6 2.642169000 7.765255000 0.977046000  8 2.177503000 6.425292000 1.079483000  15 1.040529000 5.952813000 -0.068093000  8 1.554383000 6.253496000 -1.456061000  8 -0.326297000 6.447600000 0.394760000  8 1.082019000 4.346854000 0.287404000  6 2.218670000 3.579507000 -0.163232000  1 3.143083000 4.167790000 -0.074519000  1 2.093688000 3.297837000 -1.215696000  6 2.332054000 2.311737000 0.655798000  1 3.041803000 1.643598000 0.146973000  8 2.863645000 2.596366000 1.986549000  6 2.178416000 1.815459000 2.948257000  1 2.895334000 1.357104000 3.641832000  7 1.297340000 2.710169000 3.738266000  6 0.565344000 2.350839000 4.857038000  7 0.491589000 1.143967000 5.455404000  6 -0.323577000 1.182547000 6.526539000  1 -0.434507000 0.241970000 7.071661000  7 -1.023228000 2.222677000 7.026940000  6 -0.927946000 3.421724000 6.396830000  7 -1.667741000 4.464334000 6.865050000  1 -1.426753000 5.395416000 6.553156000  1 -2.056444000 4.376542000 7.794943000  6 -0.099121000 3.530440000 5.254057000  7 0.218514000 4.589635000 4.410461000  6 1.054081000 4.060629000 3.523161000  1 1.509870000 4.581007000 2.691443000  6 1.370371000 0.749604000 2.180630000  1 0.479940000 0.438648000 2.746789000  8 2.228621000 -0.341534000 1.908967000  1 1.649623000 -1.132836000 1.746933000  1 0.246887000 2.301407000 1.195931000  6 1.009191000 1.561714000 0.909536000  8 0.510994000 0.840397000 -0.189303000  1 1.288821000 0.512415000 -0.718739000  19 -12.061287000 -1.904074000 1.369264000 | 1 -29.322028000 0.434117000 -2.779684000  8 -30.036358000 1.084757000 -3.004985000  6 -30.439341000 0.725531000 -4.320364000  1 -31.420638000 1.181424000 -4.512872000  1 -30.551498000 -0.367792000 -4.415990000  6 -29.463285000 1.169181000 -5.411603000  1 -29.687495000 0.624768000 -6.342682000  8 -29.577160000 2.596897000 -5.676905000  6 -28.297226000 3.147396000 -5.922313000  1 -28.292617000 3.718587000 -6.857241000  7 -27.968087000 4.121948000 -4.833635000  6 -28.642665000 4.123867000 -3.632653000  1 -29.326283000 3.291844000 -3.485944000  6 -26.995933000 5.079915000 -5.138684000  8 -26.359616000 5.069220000 -6.194380000  7 -26.822255000 6.039771000 -4.153955000  1 -26.129653000 6.752889000 -4.361529000  6 -27.521997000 6.174915000 -2.931568000  8 -27.294574000 7.140376000 -2.193311000  6 -28.458536000 5.096332000 -2.697372000  1 -29.001626000 5.070345000 -1.760086000  6 -27.287813000 1.987114000 -5.971890000  1 -26.301020000 2.285487000 -5.596021000  8 -27.222625000 1.505036000 -7.313113000  1 -27.049517000 0.550250000 -7.232221000  6 -27.987363000 0.980712000 -5.057477000  1 -27.822249000 1.278263000 -4.016789000  8 -27.564292000 -0.361240000 -5.286288000  8 -28.138800000 -0.887193000 -2.789990000  15 -27.186443000 -1.262703000 -3.915428000  8 -27.004591000 -2.675689000 -4.395420000  8 -25.746873000 -0.566029000 -3.471008000  6 -24.703616000 -0.405094000 -4.445677000  1 -25.132895000 -0.192833000 -5.433265000  1 -24.095168000 -1.319658000 -4.511462000  6 -23.809142000 0.738124000 -3.997171000  1 -23.017113000 0.887075000 -4.749138000  8 -24.597677000 1.944282000 -3.882839000  6 -24.216669000 2.691792000 -2.742128000  1 -23.851745000 3.688065000 -3.024258000  7 -25.400524000 2.916978000 -1.901957000  6 -25.583343000 4.029032000 -1.105870000  7 -24.785168000 5.121174000 -1.074581000  6 -25.215629000 6.049897000 -0.228320000  7 -24.486815000 7.192199000 -0.052285000  1 -23.807788000 7.360346000 -0.785071000  1 -24.974631000 8.023795000 0.258065000  7 -26.337370000 5.886899000 0.555124000  1 -26.617392000 6.634635000 1.182192000  6 -27.208104000 4.758801000 0.559094000  8 -28.176347000 4.739328000 1.342188000  6 -26.757357000 3.770575000 -0.371820000  7 -27.281912000 2.528714000 -0.709646000  6 -26.446544000 2.038884000 -1.613627000  1 -26.517717000 1.072977000 -2.099615000  6 -23.101582000 1.927392000 -1.987805000  1 -23.353376000 1.872829000 -0.923487000  8 -21.871103000 2.604703000 -2.173196000  1 -21.341244000 2.355249000 -1.390706000  6 -23.145149000 0.511754000 -2.629866000  1 -23.770053000 -0.147943000 -2.017289000  8 -21.859344000 -0.062299000 -2.831634000  15 -21.460145000 -1.521014000 -2.104185000  8 -20.330005000 -2.072754000 -2.937547000  8 -22.706552000 -2.298805000 -1.755336000  8 -20.873957000 -0.978563000 -0.631159000  6 -19.722576000 -0.131452000 -0.612043000  1 -19.643819000 0.443405000 -1.545385000  1 -18.811711000 -0.737290000 -0.490067000  6 -19.832661000 0.829073000 0.558957000  1 -18.930885000 1.460187000 0.589800000  8 -20.991926000 1.687090000 0.366799000  6 -21.720201000 1.856026000 1.583143000  1 -21.914879000 2.920003000 1.758086000  7 -23.036257000 1.213997000 1.483320000  6 -24.221447000 1.763229000 1.946510000  7 -24.364865000 2.989228000 2.509228000  6 -25.625278000 3.257613000 2.832090000  7 -25.937253000 4.442327000 3.449597000  1 -25.202094000 5.135678000 3.367046000  1 -26.863734000 4.809167000 3.243135000  7 -26.659994000 2.369116000 2.641677000  1 -27.609466000 2.690420000 2.807233000  6 -26.566334000 1.068067000 2.054213000  8 -27.577007000 0.366643000 1.942959000  6 -25.203385000 0.792032000 1.689716000  7 -24.644439000 -0.325559000 1.079855000  6 -23.353697000 -0.050184000 0.978426000  1 -22.600932000 -0.667396000 0.503059000  6 -20.851295000 1.258579000 2.698525000  1 -21.457843000 0.826696000 3.505117000  8 -19.981796000 2.275549000 3.178766000  1 -19.133654000 1.807947000 3.319006000  6 -20.019560000 0.189852000 1.946057000  1 -20.564480000 -0.760104000 1.869402000  8 -18.739810000 0.013769000 2.540928000  15 -18.646249000 -1.026968000 3.902075000  8 -18.445695000 -2.439364000 3.403748000  8 -19.750138000 -0.665864000 4.865061000  8 -17.245037000 -0.371462000 4.493473000  6 -16.099499000 -0.262829000 3.629204000  1 -16.015823000 0.782246000 3.294490000  1 -16.187042000 -0.914813000 2.751647000  6 -14.836361000 -0.672070000 4.372190000  1 -13.971052000 -0.471558000 3.722534000  8 -14.668986000 0.121589000 5.580054000  6 -14.699493000 -0.722918000 6.719850000  1 -14.021106000 -0.294242000 7.465916000  7 -16.048972000 -0.739090000 7.325660000  6 -17.044047000 -1.713227000 7.356720000  7 -16.986264000 -3.008969000 6.992711000  6 -18.165834000 -3.632777000 7.164675000  1 -18.186543000 -4.687861000 6.882094000  7 -19.328019000 -3.136070000 7.632114000  6 -19.356288000 -1.830925000 7.997620000  7 -20.513648000 -1.321039000 8.504776000  1 -20.611062000 -0.314032000 8.513838000  1 -21.357132000 -1.851649000 8.325598000  6 -18.176429000 -1.059601000 7.882266000  7 -17.907795000 0.276622000 8.157145000  6 -16.641455000 0.422561000 7.805928000  1 -16.073636000 1.344977000 7.864236000  6 -14.221278000 -2.097463000 6.245706000  1 -14.593161000 -2.922001000 6.866948000  8 -12.796816000 -2.050946000 6.215664000  1 -12.554950000 -2.604013000 5.448950000  6 -14.794978000 -2.153389000 4.813092000  1 -15.799773000 -2.590013000 4.831766000  8 -13.901373000 -2.942399000 4.018145000  15 -14.547587000 -3.727772000 2.678525000  8 -15.018806000 -2.658380000 1.699471000  8 -15.464065000 -4.843096000 3.109574000  8 -13.071215000 -4.269169000 2.165955000  6 -12.885493000 -5.599126000 1.663092000  1 -13.412761000 -6.331334000 2.289652000  1 -11.808969000 -5.779620000 1.731739000  6 -13.312605000 -5.790426000 0.205483000  1 -12.635407000 -6.526260000 -0.259102000  8 -14.671040000 -6.288665000 0.131779000  6 -15.294152000 -5.836539000 -1.082113000  1 -15.598662000 -6.707600000 -1.674118000  7 -16.514661000 -5.098219000 -0.753492000  6 -17.677272000 -5.702592000 -0.302054000  7 -17.884421000 -7.037811000 -0.208180000  6 -19.088733000 -7.339262000 0.265504000  7 -19.470802000 -8.649973000 0.345704000  1 -18.704740000 -9.309208000 0.278467000  1 -20.185399000 -8.904640000 1.016495000  7 -20.011387000 -6.384030000 0.633492000  1 -20.917285000 -6.675071000 0.989065000  6 -19.839489000 -4.961421000 0.566072000  8 -20.750609000 -4.221335000 0.954131000  6 -18.545033000 -4.647833000 0.026840000  7 -17.932811000 -3.419457000 -0.210620000  6 -16.729280000 -3.721217000 -0.658526000  1 -15.962444000 -3.006175000 -0.923404000  6 -14.257367000 -4.978402000 -1.820438000  1 -14.706862000 -4.132787000 -2.359335000  8 -13.524607000 -5.815343000 -2.711313000  1 -12.632930000 -5.419430000 -2.740605000  6 -13.362693000 -4.526035000 -0.654042000  1 -13.857586000 -3.733538000 -0.086923000  8 -12.067340000 -4.144777000 -1.090601000  15 -11.824217000 -2.585951000 -1.655653000  8 -13.120767000 -1.802923000 -1.532749000  8 -11.096929000 -2.761673000 -2.975696000  8 -10.815578000 -1.928342000 -0.519498000  6 -9.420254000 -2.304661000 -0.583435000  1 -8.842776000 -1.471340000 -1.011262000  1 -9.299540000 -3.189206000 -1.211637000  6 -8.860778000 -2.623610000 0.792908000  1 -8.812932000 -1.728373000 1.432175000  8 -9.695871000 -3.610088000 1.447264000  6 -8.915572000 -4.629211000 2.039647000  1 -9.148780000 -4.710453000 3.109697000  7 -9.248974000 -5.943658000 1.468451000  6 -9.424580000 -6.320875000 0.140789000  7 -9.424866000 -5.513373000 -0.940286000  6 -9.766508000 -6.128818000 -2.068236000  7 -9.899862000 -5.401621000 -3.212404000  1 -10.199191000 -4.418730000 -3.099355000  1 -10.272006000 -5.867925000 -4.030081000  7 -9.976030000 -7.494489000 -2.130601000  1 -10.238905000 -7.920945000 -3.014192000  6 -9.911219000 -8.415663000 -1.035380000  8 -10.105881000 -9.621583000 -1.228978000  6 -9.634084000 -7.712197000 0.185707000  7 -9.571653000 -8.181297000 1.496551000  6 -9.341445000 -7.103211000 2.225449000  1 -9.201303000 -7.082495000 3.298365000  6 -7.438193000 -4.257661000 1.868637000  1 -6.832308000 -5.140661000 1.630931000  8 -7.019947000 -3.641603000 3.078907000  1 -6.103624000 -3.343460000 2.881457000  6 -7.466149000 -3.270362000 0.680646000  1 -7.394976000 -3.845085000 -0.251812000  8 -6.434575000 -2.281094000 0.727024000  15 -4.857664000 -2.786201000 0.440637000  8 -4.028597000 -1.546350000 0.227038000  8 -4.873673000 -3.964184000 -0.505653000  8 -4.533102000 -3.405562000 1.991208000  6 -3.415468000 -2.944192000 2.767549000  1 -3.788439000 -2.707670000 3.773576000  1 -2.990219000 -2.035926000 2.320882000  6 -2.333176000 -4.011839000 2.880947000  1 -1.531894000 -3.613778000 3.518562000  8 -2.866081000 -5.211510000 3.527472000  6 -2.826088000 -6.307945000 2.628850000  1 -2.017952000 -7.011522000 2.882723000  7 -4.078837000 -7.056447000 2.740881000  6 -4.268845000 -8.378322000 2.360868000  7 -3.337463000 -9.277971000 1.988946000  6 -3.902524000 -10.464229000 1.695116000  1 -3.211165000 -11.257357000 1.399230000  7 -5.205166000 -10.810152000 1.699526000  6 -6.125085000 -9.878011000 2.071371000  7 -7.434633000 -10.240041000 2.076714000  1 -8.165754000 -9.524465000 2.007798000  1 -7.636484000 -11.120762000 1.617961000  6 -5.661364000 -8.593289000 2.462505000  7 -6.303039000 -7.444985000 2.915798000  6 -5.329940000 -6.554065000 3.060670000  1 -5.453339000 -5.534854000 3.402216000  6 -2.593502000 -5.716322000 1.210415000  1 -3.548830000 -5.389980000 0.785422000  8 -1.989765000 -6.605040000 0.295340000  1 -1.062416000 -6.669057000 0.587056000  6 -1.738631000 -4.489935000 1.553496000  1 -1.746768000 -3.716581000 0.777769000  8 -0.407312000 -4.997777000 1.778808000  15 0.905546000 -4.018827000 1.452757000  8 2.058278000 -4.623483000 2.209698000  8 0.492177000 -2.561610000 1.583452000  8 1.067759000 -4.242905000 -0.188339000  6 1.403940000 -5.541748000 -0.711676000  1 0.705167000 -5.732512000 -1.536996000  1 1.265285000 -6.326498000 0.046130000  6 2.836298000 -5.638174000 -1.239534000  1 2.942389000 -6.621477000 -1.714567000  8 3.087195000 -4.610908000 -2.244027000  6 4.058289000 -3.686808000 -1.752590000  1 5.020224000 -3.827720000 -2.256984000  7 3.621236000 -2.331532000 -2.055828000  6 4.348352000 -1.344703000 -2.706923000  7 5.596943000 -1.468799000 -3.210952000  6 6.051168000 -0.335309000 -3.747717000  7 7.274492000 -0.321516000 -4.351674000  1 7.963015000 -0.971502000 -3.948888000  1 7.646546000 0.590285000 -4.589656000  7 5.313795000 0.826949000 -3.792338000  1 5.783982000 1.694667000 -4.089413000  6 4.008365000 1.014297000 -3.262281000  8 3.478777000 2.136356000 -3.310665000  6 3.509370000 -0.214803000 -2.713447000  7 2.298514000 -0.514404000 -2.093801000  6 2.401157000 -1.774893000 -1.707643000  1 1.654403000 -2.347580000 -1.171797000  6 4.187202000 -3.957283000 -0.232164000  1 3.370542000 -3.456033000 0.292145000  8 5.385482000 -3.499416000 0.348767000  1 6.100535000 -4.100640000 0.065519000  6 3.944858000 -5.471536000 -0.192661000  1 3.653999000 -5.828635000 0.800899000  8 5.181785000 -6.045305000 -0.638599000  15 5.472396000 -7.697584000 -0.671661000  8 4.190643000 -8.429362000 -1.006597000  8 6.341543000 -8.075030000 0.502091000  8 6.507939000 -7.661329000 -1.993136000  6 5.936115000 -7.507412000 -3.300933000  1 4.872648000 -7.243522000 -3.235637000  1 6.012868000 -8.466492000 -3.836417000  6 6.648158000 -6.458117000 -4.139418000  1 6.242842000 -6.538156000 -5.162259000  8 6.368394000 -5.124089000 -3.636466000  6 7.517780000 -4.297995000 -3.761360000  1 7.233446000 -3.331472000 -4.189255000  7 8.064766000 -4.032383000 -2.414661000  6 8.803043000 -2.933611000 -2.015072000  7 9.263958000 -1.936168000 -2.806683000  6 9.999461000 -1.037722000 -2.134057000  7 10.511488000 0.029145000 -2.794415000  1 10.530757000 -0.059771000 -3.802023000  1 11.276276000 0.573358000 -2.357608000  7 10.247120000 -1.117009000 -0.784693000  1 10.855908000 -0.385666000 -0.333329000  6 9.764557000 -2.124758000 0.078264000  8 10.030228000 -2.103130000 1.297165000  6 9.003368000 -3.106128000 -0.633795000  7 8.422174000 -4.293374000 -0.196911000  6 7.884125000 -4.824329000 -1.285775000  1 7.341425000 -5.754693000 -1.348358000  6 8.523139000 -5.070746000 -4.613706000  1 9.552449000 -4.778795000 -4.391631000  8 8.229223000 -4.859686000 -5.996020000  1 8.341312000 -5.738679000 -6.405397000  6 8.182838000 -6.522014000 -4.224495000  1 8.613081000 -6.789521000 -3.252833000  8 8.602810000 -7.441693000 -5.232329000  15 10.046870000 -8.287807000 -4.947687000  8 10.227670000 -9.147969000 -6.170384000  8 10.049733000 -8.794202000 -3.527765000  8 11.163138000 -7.035952000 -4.945825000  6 11.478788000 -6.371638000 -6.173783000  1 10.562295000 -6.042070000 -6.683129000  1 12.028880000 -7.043758000 -6.848943000  6 12.343637000 -5.156807000 -5.892131000  1 12.582794000 -4.682508000 -6.857310000  8 11.609524000 -4.201248000 -5.076404000  6 12.469719000 -3.578272000 -4.146901000  1 12.341638000 -2.492869000 -4.179511000  7 12.135155000 -4.006140000 -2.755754000  6 11.468739000 -5.178298000 -2.478627000  1 11.147743000 -5.744425000 -3.347174000  6 12.659604000 -3.210535000 -1.744065000  8 13.286324000 -2.166780000 -2.016215000  7 12.446875000 -3.660224000 -0.469540000  1 12.838403000 -3.041958000 0.280703000  6 11.818239000 -4.863016000 -0.093987000  8 11.808537000 -5.210334000 1.098969000  6 11.268554000 -5.606847000 -1.204567000  1 10.750862000 -6.539789000 -1.013526000  6 13.901442000 -4.001360000 -4.501282000  1 14.552670000 -4.041253000 -3.618130000  8 14.400320000 -3.093109000 -5.474532000  1 14.893291000 -3.639573000 -6.118102000  6 13.658860000 -5.387286000 -5.131237000  1 13.551515000 -6.158235000 -4.359154000  8 14.686765000 -5.732131000 -6.053534000  15 16.006767000 -6.627703000 -5.521746000  8 16.735008000 -7.037906000 -6.780317000  8 15.566078000 -7.607196000 -4.460305000  8 16.853901000 -5.359266000 -4.798785000  6 18.262220000 -5.600588000 -4.619834000  1 18.635357000 -6.230113000 -5.438773000  1 18.441610000 -6.119984000 -3.664268000  6 19.049406000 -4.298684000 -4.606551000  1 20.093865000 -4.547390000 -4.387421000  8 19.010799000 -3.639845000 -5.907800000  6 18.238103000 -2.457202000 -5.842066000  1 18.860956000 -1.562012000 -5.989392000  7 17.269108000 -2.481205000 -6.944140000  6 16.622230000 -1.382200000 -7.475812000  7 16.837683000 -0.087200000 -7.137804000  6 16.058786000 0.756374000 -7.804629000  7 16.212956000 2.103102000 -7.617506000  1 16.732125000 2.345669000 -6.782135000  1 15.422947000 2.707295000 -7.807607000  7 15.125929000 0.347714000 -8.735060000  1 14.566131000 1.043475000 -9.219564000  6 14.856922000 -1.004144000 -9.130652000  8 13.988693000 -1.233758000 -9.980345000  6 15.710390000 -1.907825000 -8.410083000  7 15.794361000 -3.294603000 -8.454126000  6 16.731029000 -3.605509000 -7.566775000  1 17.069038000 -4.605480000 -7.325308000  6 17.572080000 -2.416031000 -4.432005000  1 16.598413000 -2.907701000 -4.487796000  8 17.376218000 -1.109801000 -3.920554000  1 18.255344000 -0.851768000 -3.581126000  6 18.562227000 -3.249758000 -3.607259000  1 18.104776000 -3.695506000 -2.719895000  8 19.622249000 -2.348698000 -3.243617000  15 20.669477000 -2.839728000 -2.009441000  8 21.817098000 -1.864420000 -2.059971000  8 20.823167000 -4.339650000 -2.044675000  8 19.702656000 -2.535508000 -0.676827000  6 19.440144000 -1.166204000 -0.329809000  1 18.739336000 -0.723377000 -1.052951000  1 20.374377000 -0.587066000 -0.342213000  6 18.823059000 -1.111786000 1.057391000  1 18.746615000 -0.058332000 1.357501000  8 17.475315000 -1.650239000 1.008603000  6 17.340705000 -2.779084000 1.850892000  1 16.756489000 -2.551169000 2.748792000  7 16.571946000 -3.797078000 1.105880000  6 16.896734000 -4.050468000 -0.208426000  1 17.756282000 -3.490019000 -0.568557000  6 15.480547000 -4.419316000 1.731240000  8 15.162925000 -4.214226000 2.900495000  7 14.799527000 -5.307444000 0.911985000  1 13.915109000 -5.644490000 1.294881000  6 15.057537000 -5.625186000 -0.439106000  8 14.334846000 -6.429633000 -1.041067000  6 16.202574000 -4.926509000 -0.985004000  1 16.470444000 -5.107475000 -2.021217000  6 18.776953000 -3.230248000 2.218706000  1 19.142526000 -3.899335000 1.428964000  8 18.863836000 -3.926754000 3.438795000  1 18.827470000 -3.261728000 4.156716000  6 19.576574000 -1.906744000 2.133715000  1 20.625421000 -2.084641000 1.875083000  8 19.491759000 -1.182185000 3.368718000  15 20.730353000 -1.307873000 4.501562000  8 20.871085000 0.059434000 5.127443000  8 21.883456000 -2.082527000 3.912581000  8 19.978761000 -2.344732000 5.605694000  6 19.243673000 -1.755594000 6.688792000  1 19.875414000 -1.038530000 7.231949000  1 18.984063000 -2.583153000 7.362769000  6 17.989894000 -1.026160000 6.216453000  1 18.277388000 -0.200863000 5.558289000  8 17.138196000 -1.925137000 5.438759000  6 15.879197000 -2.082393000 6.082112000  1 15.103993000 -1.485074000 5.589938000  7 15.461579000 -3.491061000 5.950840000  6 16.393610000 -4.499522000 6.010643000  1 17.414446000 -4.160146000 6.150461000  6 14.101804000 -3.757949000 5.726786000  8 13.238060000 -2.885990000 5.650848000  7 13.810408000 -5.109776000 5.624480000  1 12.835840000 -5.320760000 5.433324000  6 14.700947000 -6.204352000 5.621927000  8 14.276422000 -7.350839000 5.434847000  6 16.075159000 -5.814114000 5.864847000  1 16.841671000 -6.580789000 5.898625000  6 16.101575000 -1.641206000 7.549445000  1 16.630403000 -2.460486000 8.063495000  8 14.940674000 -1.379880000 8.287600000  1 14.543120000 -0.518589000 7.976971000  6 17.115522000 -0.490038000 7.356003000  1 17.674815000 -0.261085000 8.273856000  8 16.497291000 0.690095000 6.840568000  15 15.533973000 1.712755000 7.739568000  8 16.242394000 2.110424000 9.011098000  8 14.117785000 1.149687000 7.741691000  8 15.515549000 2.932387000 6.611921000  6 16.720812000 3.700461000 6.434734000  1 17.557606000 3.247444000 6.981143000  1 16.555490000 4.709301000 6.841718000  6 17.103106000 3.863306000 4.973239000  1 17.925782000 4.597849000 4.943579000  8 17.578674000 2.615551000 4.400238000  6 17.167059000 2.519780000 3.043417000  1 18.019034000 2.249698000 2.406559000  7 16.159605000 1.447892000 2.921226000  6 15.775763000 0.800871000 1.759722000  7 16.220048000 1.050238000 0.506965000  6 15.682516000 0.233440000 -0.399524000  7 16.009239000 0.399822000 -1.718724000  1 16.779837000 1.044182000 -1.851699000  1 16.063781000 -0.414318000 -2.328465000  7 14.759050000 -0.741532000 -0.099881000  1 14.293725000 -1.267958000 -0.859986000  6 14.287751000 -1.039521000 1.196029000  8 13.471470000 -1.967099000 1.391275000  6 14.847652000 -0.170371000 2.175535000  7 14.645054000 -0.107158000 3.549144000  6 15.438846000 0.867400000 3.961853000  1 15.585950000 1.186443000 4.980613000  6 16.566752000 3.874900000 2.656743000  1 15.815398000 3.775208000 1.867827000  8 17.614719000 4.754052000 2.244602000  1 17.393262000 5.609366000 2.659671000  6 16.001656000 4.336076000 4.012511000  1 15.053214000 3.837189000 4.241573000  8 15.860166000 5.754503000 4.069058000  15 14.294315000 6.379622000 3.867792000  8 14.465961000 7.871919000 3.972016000  8 13.327390000 5.563975000 4.687513000  8 13.982568000 5.936480000 2.282774000  6 14.695113000 6.581809000 1.222141000  1 15.779508000 6.518088000 1.388780000  1 14.414165000 7.643334000 1.157693000  6 14.358161000 5.914482000 -0.097357000  1 14.888683000 6.459207000 -0.894674000  8 14.812652000 4.531403000 -0.097599000  6 13.882267000 3.707514000 -0.777896000  1 14.399358000 3.065573000 -1.496583000  7 13.187176000 2.799733000 0.182767000  6 13.149837000 3.046953000 1.526190000  1 13.655395000 3.946492000 1.861797000  6 12.490370000 1.705722000 -0.373392000  8 12.519094000 1.561230000 -1.618714000  7 11.833863000 0.863259000 0.461598000  6 11.843254000 1.064145000 1.796315000  7 11.201596000 0.168009000 2.563497000  1 11.382071000 0.167707000 3.557458000  1 10.832712000 -0.689420000 2.124689000  6 12.491305000 2.203832000 2.371350000  1 12.474186000 2.392445000 3.439425000  6 12.861299000 4.638402000 -1.442287000  1 11.880279000 4.162785000 -1.546159000  8 13.362285000 5.055326000 -2.713932000  1 13.158912000 6.009474000 -2.755579000  6 12.870632000 5.824723000 -0.462559000  1 12.271674000 5.608516000 0.429522000  8 12.429853000 7.028229000 -1.087571000  15 10.827438000 7.490375000 -0.760615000  8 10.624015000 8.743217000 -1.573218000  8 10.580755000 7.375604000 0.721100000  8 9.995100000 6.203242000 -1.437879000  6 10.009159000 6.063092000 -2.865590000  1 11.025821000 5.847174000 -3.221578000  1 9.664123000 6.987680000 -3.346240000  6 9.095805000 4.928800000 -3.278877000  1 9.046816000 4.916563000 -4.379669000  8 9.638967000 3.661457000 -2.819426000  6 8.587139000 2.787779000 -2.439230000  1 8.702228000 1.817706000 -2.935750000  7 8.664060000 2.521920000 -0.997327000  6 8.052297000 1.445375000 -0.376552000  7 7.315136000 0.474805000 -0.945886000  6 6.866323000 -0.403533000 -0.027114000  1 6.266557000 -1.232536000 -0.402966000  7 7.076563000 -0.399791000 1.306680000  6 7.829922000 0.590490000 1.844127000  7 8.017658000 0.606727000 3.197009000  1 8.836639000 1.107527000 3.520620000  1 7.838746000 -0.273045000 3.667470000  6 8.347548000 1.597147000 0.992741000  7 9.104494000 2.745926000 1.212317000  6 9.269030000 3.272664000 0.005072000  1 9.793604000 4.191521000 -0.231210000  6 7.266834000 3.481855000 -2.798203000  1 6.450619000 3.212608000 -2.114886000  8 6.931570000 3.150429000 -4.153825000  1 6.486669000 3.949906000 -4.501504000  6 7.666340000 4.963662000 -2.719402000  1 7.659779000 5.291519000 -1.674996000  8 6.829147000 5.778610000 -3.528742000  15 5.560704000 6.648009000 -2.786199000  8 5.479749000 6.225761000 -1.334085000  8 4.390020000 6.585781000 -3.730722000  8 6.175874000 8.185141000 -2.922566000  6 7.442300000 8.492002000 -2.308537000  1 8.163181000 8.722500000 -3.102900000  1 7.833639000 7.649956000 -1.726198000  6 7.312206000 9.679161000 -1.369358000  1 8.310202000 9.905854000 -0.968258000  8 6.821757000 10.831731000 -2.099727000  6 5.714893000 11.415485000 -1.426331000  1 5.847745000 12.501883000 -1.388115000  7 4.475430000 11.215864000 -2.192407000  6 3.910692000 12.183589000 -3.003936000  7 4.354602000 13.453189000 -3.149417000  6 3.588179000 14.182837000 -3.951231000  7 3.880871000 15.499878000 -4.152340000  1 4.809426000 15.774386000 -3.856595000  1 3.551187000 15.950175000 -4.996674000  7 2.455234000 13.684827000 -4.562814000  1 1.865967000 14.309047000 -5.105893000  6 1.937908000 12.365632000 -4.423317000  8 0.844315000 12.086995000 -4.953623000  6 2.799332000 11.566348000 -3.608102000  7 2.686439000 10.243070000 -3.188291000  6 3.698412000 10.068295000 -2.357325000  1 3.947683000 9.141036000 -1.861502000  6 5.685062000 10.841997000 0.001101000  1 4.660552000 10.743974000 0.383336000  8 6.470957000 11.701864000 0.800232000  1 6.394482000 11.277885000 1.708517000  6 6.330640000 9.451338000 -0.208480000  1 5.559571000 8.743952000 -0.539832000  8 7.056492000 8.862584000 0.878072000  15 6.734370000 8.900681000 2.516419000  8 6.055099000 10.216971000 2.890787000  8 8.017431000 8.507716000 3.201322000  8 5.694798000 7.619979000 2.750280000  6 4.738933000 7.206023000 1.763506000  1 4.689765000 6.108125000 1.802220000  1 5.067156000 7.466450000 0.754938000  6 3.335273000 7.721018000 2.029641000  1 2.928520000 7.275358000 2.954473000  8 3.325206000 9.156246000 2.130753000  6 1.970510000 9.594876000 1.956059000  1 1.505123000 9.762212000 2.939525000  7 2.039478000 10.893911000 1.275007000  6 1.391056000 11.394654000 0.152483000  7 0.550300000 10.732454000 -0.678751000  6 0.129500000 11.462257000 -1.701928000  7 -0.662010000 10.908400000 -2.675584000  1 -0.677892000 9.895853000 -2.601878000  1 -0.411025000 11.220720000 -3.619571000  7 0.426323000 12.800790000 -1.839211000  1 0.055114000 13.299854000 -2.642124000  6 1.342620000 13.544510000 -1.035308000  8 1.588137000 14.725475000 -1.310778000  6 1.844663000 12.723916000 0.032837000  7 2.752902000 13.036095000 1.037758000  6 2.840820000 11.932043000 1.756394000  1 3.493452000 11.769884000 2.605922000  6 1.200496000 8.463419000 1.239132000  1 0.686767000 8.814675000 0.339362000  8 0.294770000 7.897728000 2.174188000  1 -0.173221000 7.203178000 1.636087000  1 2.820724000 7.803563000 -0.064163000  6 2.354178000 7.483816000 0.880871000  8 1.966533000 6.120306000 0.799781000  15 0.759153000 5.757701000 -0.316655000  8 1.180252000 6.223490000 -1.689960000  8 -0.579584000 6.169625000 0.285721000  8 0.842942000 4.125684000 -0.138965000  6 1.958093000 3.412186000 -0.713105000  1 2.862055000 4.036723000 -0.720094000  1 1.728623000 3.130270000 -1.747990000  6 2.212590000 2.143231000 0.077563000  1 2.859763000 1.493083000 -0.527349000  8 2.919660000 2.437965000 1.320274000  6 2.391794000 1.649483000 2.370750000  1 3.208080000 1.188554000 2.942472000  7 1.644032000 2.539391000 3.292182000  6 1.096904000 2.174886000 4.510669000  7 1.105924000 0.962635000 5.101820000  6 0.473619000 0.997291000 6.290445000  1 0.439139000 0.051470000 6.836494000  7 -0.123366000 2.038922000 6.907077000  6 -0.114897000 3.243869000 6.281567000  7 -0.755082000 4.289876000 6.873714000  1 -0.549573000 5.221352000 6.537880000  1 -0.985106000 4.196065000 7.854752000  6 0.519477000 3.356834000 5.020819000  7 0.711261000 4.422254000 4.147481000  6 1.387879000 3.894803000 3.132021000  1 1.714790000 4.428267000 2.249249000  6 1.476742000 0.581446000 1.736910000  1 0.667201000 0.296042000 2.423638000  8 2.266708000 -0.536196000 1.377072000  1 1.649984000 -1.315211000 1.366134000  1 0.214482000 2.098278000 0.876436000  6 0.950995000 1.369463000 0.508811000  8 0.334707000 0.624474000 -0.512867000  1 1.052353000 0.251774000 -1.092241000  11 -13.766368000 -1.094067000 0.608257000 |
[truncated: 490,511 more chars]
